# Supplementary material for: Identifying phenotype-genotype-function coupling in 3D organoid imaging using Shape, Appearance and Motion Phenotype Observation Tool (SPOT)
Source: Nat Commun. 2026 Aug 17;17:8410. doi: 10.1038/s41467-026-75506-7 (PMC13482367; doi:10.1038/s41467-026-75506-7)
Supplement: Supplementary file 1 [file 41467_2026_75506_MOESM1_ESM.pdf]

Identifying phenotype-genotype-function coupling in 3D organoid imaging using Shape, Appearance and Motion Phenotype Observation Tool (SPOT)

Authors

Felix Y. Zhou[1,2,5,6,7^*], Brittany-Amber Jacobs[1^], Adam Norton-Steele[1^], Xiaoyue Han[1,8^], Linna Zhou[1,3,8], Thomas M. Carroll[1], Carlos Ruiz Puig[1], Joseph Chadwick[1], Xiao Qin[1,9], Richard Lisle[1], Lewis Marsh[1,4,10], Helen M. Byrne[1,4], Heather A. Harrington[4,11], and Xin Lu[1,*]

[1]Ludwig Institute for Cancer Research, Nuffield Department of Medicine, University of Oxford, Oxford, OX3 7DQ, UK

[2]Lyda Hill Department of Bioinformatics, University of Texas Southwestern Medical Center, Dallas, TX, USA

[3]Department of Chemistry, University of Oxford, Oxford, OX1 3TA, UK

[4]Mathematical Institute, University of Oxford, Woodstock Road, Oxford, OX2 6GG, UK

[5]Present address: Department of Cell & Developmental Biology, Vanderbilt University, Nashville, TN, USA

[6]Present address: Center for Computational Systems Biology, Vanderbilt University, Nashville, TN, USA

[7]Present address: Vanderbilt Ingram Cancer Center, Vanderbilt University Medical Center, Nashville, TN, USA

[8]Present address: Ellison Institute of Technology, The Oxford Science Park, Heatley Road, Littlemore, Oxford, OX4 4GE, UK

[9]Present address: MRC Translational Discovery Unit (TIDU), MRC Weatherall Institute of Molecular Medicine, University of Oxford, Oxford, OX3 9DS, UK

[10]Present address: Novo Nordisk Research Centre Oxford (NNRCO), Innovation Building, Roosevelt Dr, Headington, Oxford, OX3 7FZ, UK

[11]Present address: Max Planck Institute of Molecular Cell Biology and Genetics, Dresden, Germany

^These authors contributed equally

*Corresponding authors

Correspondence

Correspondence to Felix Zhou, felix.y.zhou@vanderbilt.edu or Xin Lu, xin.lu@ludwig.ox.ac.uk

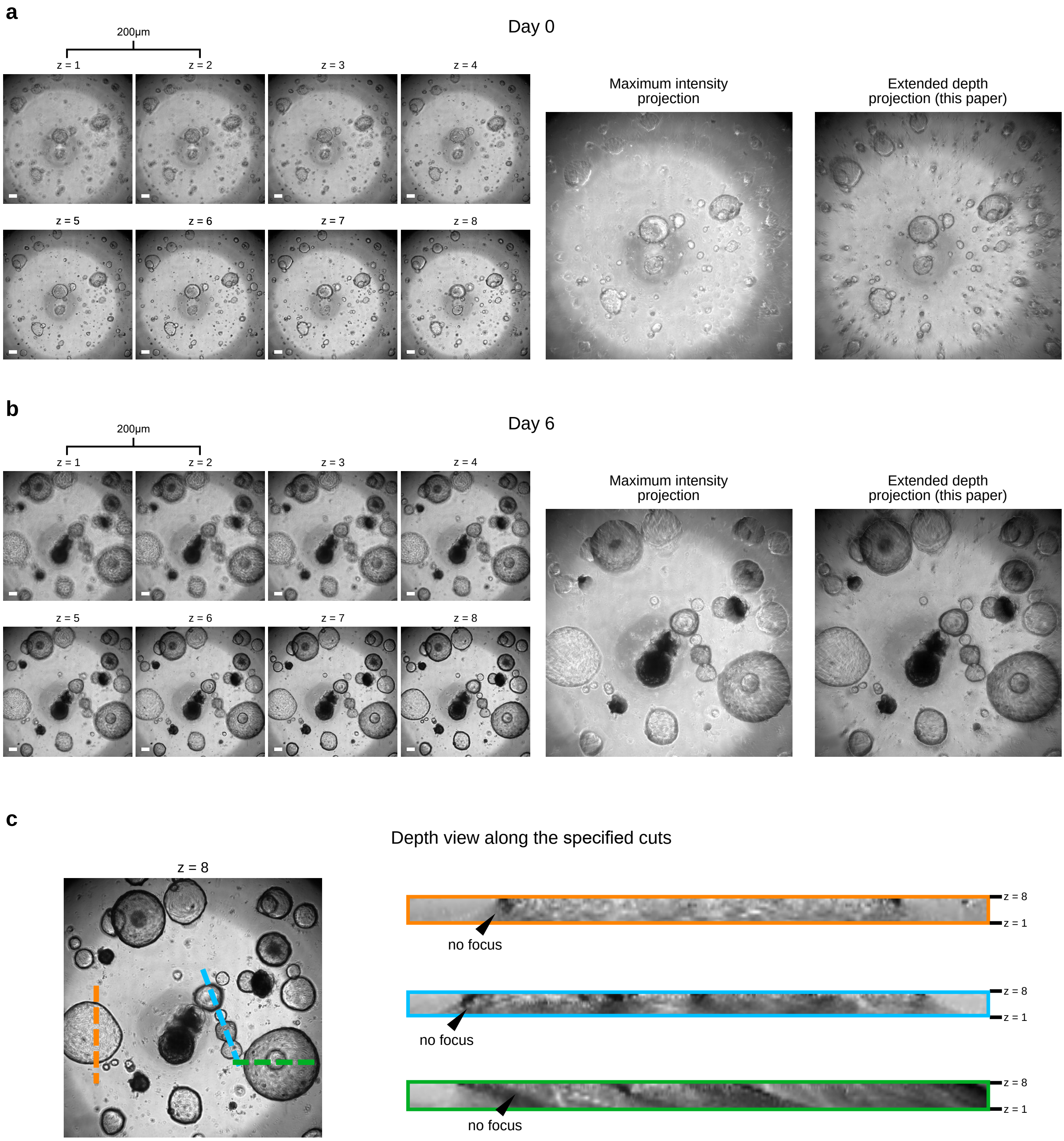

Supplementary Figure 1 . Brightfield phase contrast imaging has limited depth resolution and extended focus projection of a z-stack to a 2D image. a) Individual z-slices of the acquired 8-slice z-stack at day 0 (left), its 2D maximum intensity projection (middle), and 2D extended focus projection used in this paper (right). Each z-slice is separated by a depth of 200 µm. b) Same as a) of the same organoids acquired at day 6. c) Reslice of the z-stack image along the indicated color line indicated in the corresponding z=8 xy-slice on the left. Black triangle mark the edge of the organoid. All scale bars: 250 µm.

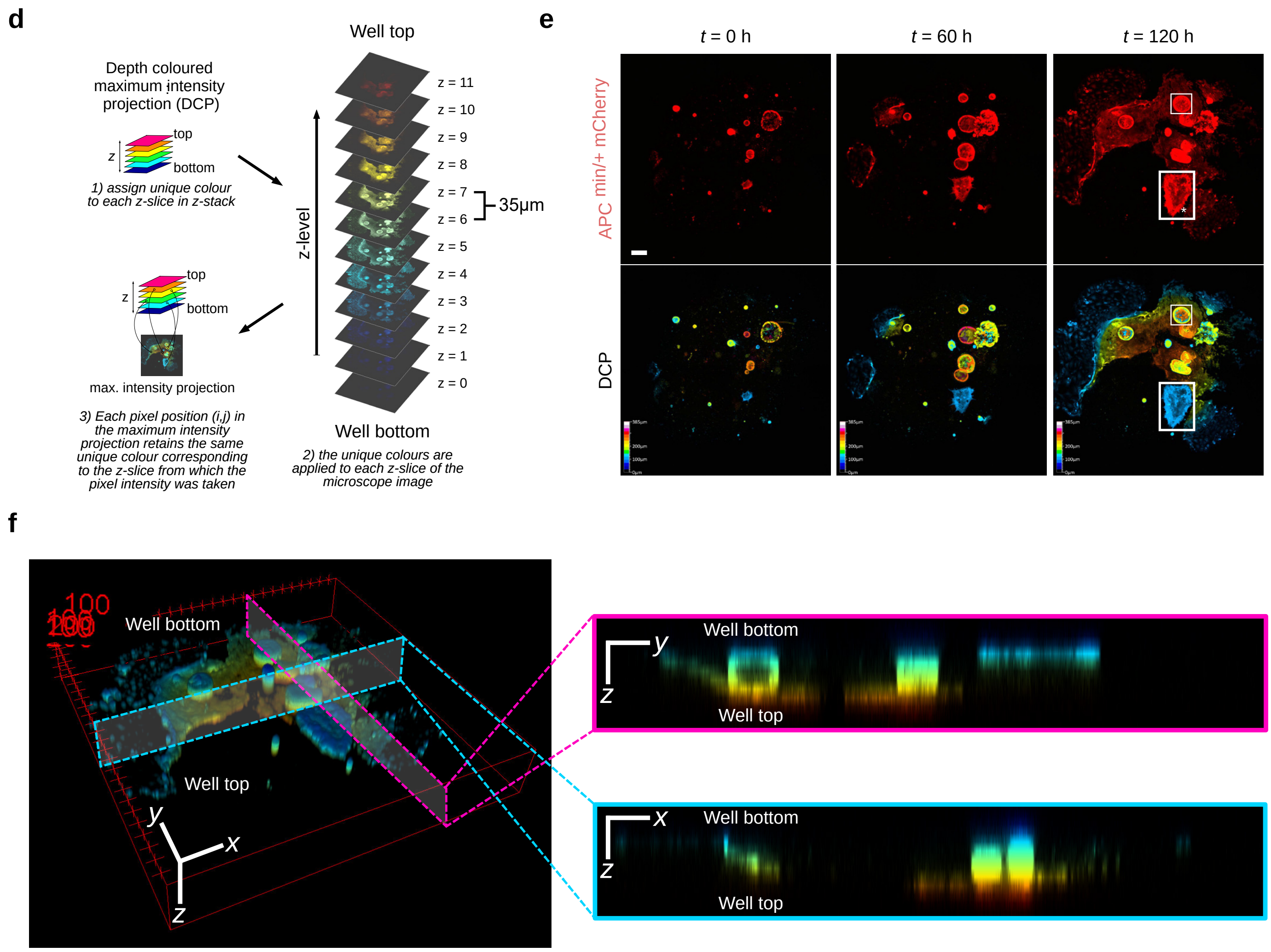

Supplementary Figure 1 cont. Acquired confocal z-stacks have depth-limited resolution. d) Schematic of computing a depth colored z-stack and its maximum intensity projection 2D image (DCP). e) DCP of initial, intermediate and final timepoint of APC mutant colon organoid undergoing large morphological transformation illustrating the effective capture of in-focussed organoids from different z-slices using 2D projection, here by maximum intensity. Scale bar: 200 μm. f) 3D render of the z-stack of the final timepoint 120 of b) (left) and its reslice in the two orthogonal colored planes (right). The coarse depth resolution offers limited 3D shape, and appearance information, whilst the available space in the ECM dome constrains organoid movement to be largely planar in xy.

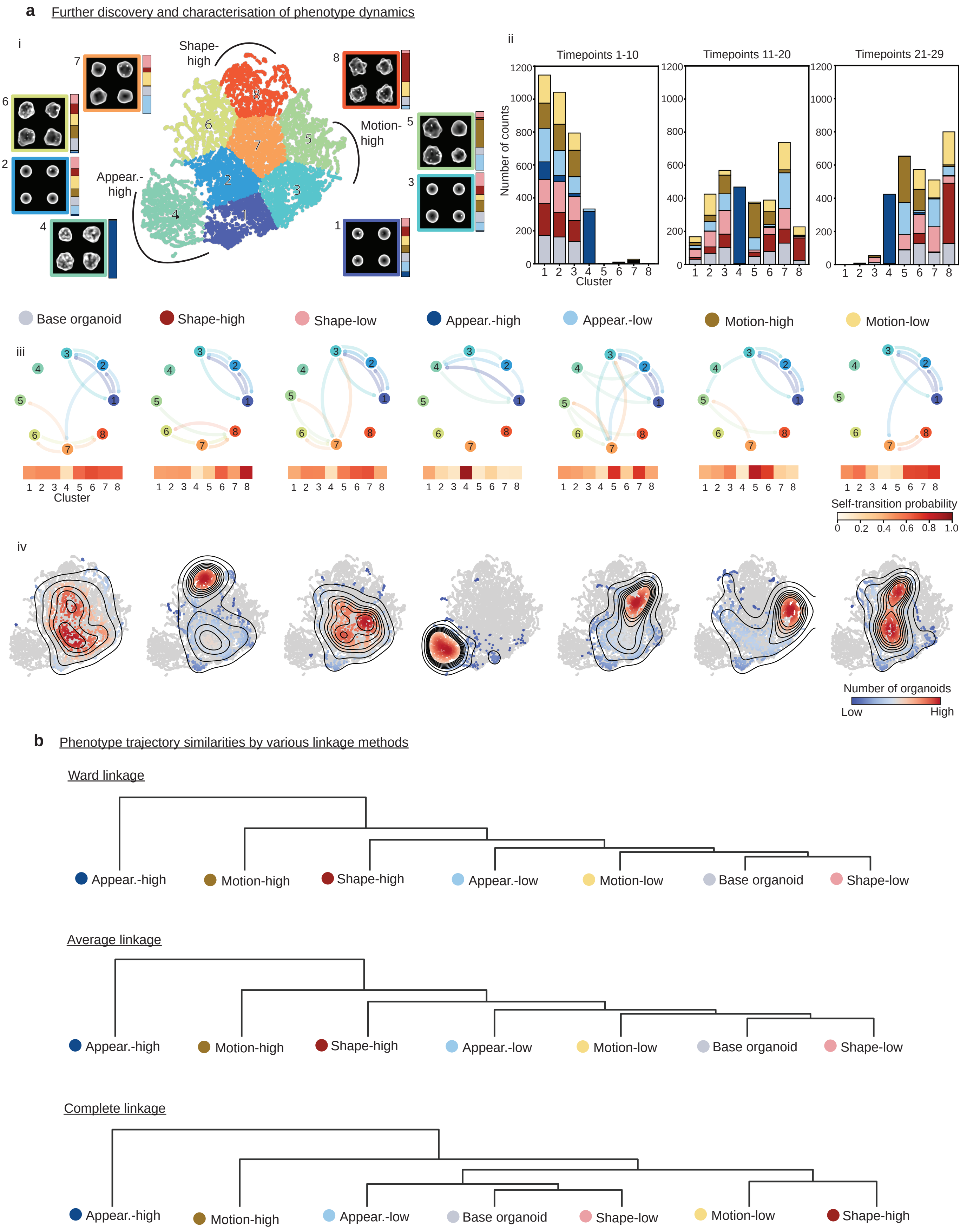

Supplementary Figure 2. SPOT phenotype cluster transition and density maps for Simulated Dataset A. a) (i) UMAP phenomic landscape of all organoids and conditions, same as Fig. 2d. (ii) Stacked barplots showing the total number of organoid instances and the behavior composition in each SPOT phenotype from early (timepoints 1-10, left); mid (timepoints 11-20, middle); and late (timepoints 21-30) timepoints of simulated videos. (iii) Graphs depicting the next timepoint relative transition probabilities of changing to a different phenotype cluster (top, inter-cluster), and heatmap

showing the next timepoint probability of staying in the same phenotype cluster (bottom, self-cluster). Arrow color represents the cluster of origin and its transparency reflects the probability magnitude – the more solid the color, the higher the probability. Pseudocounts were added to compute cluster transitions to prevent division by zero and the inter-cluster transitions used a lower cut-off of 0.2 to better visualize the most dominant transitions. (iv) UMAP point density heatmaps displaying how high and low perturbations of organoid behaviour in shape, appearance and motion map within the phenomic landscape. b) Hierarchical clustering of phenotype trajectories per condition, using Ward, average and complete linkage, top-to-bottom.

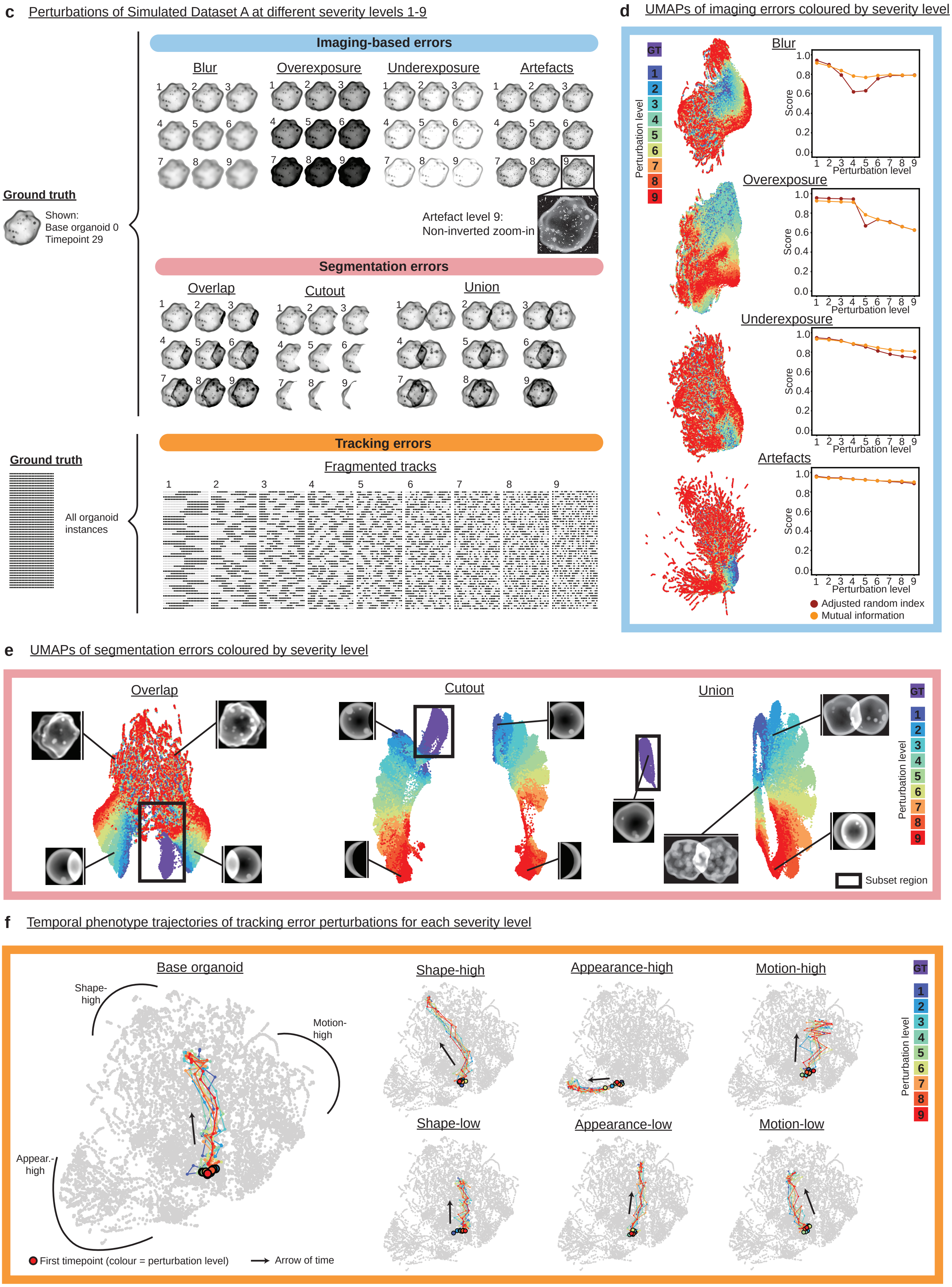

Supplementary Figure 2 cont. Robustness of SPOT analysis to imaging artefact, segmentation and tracking errors. c) Depiction of simulated perturbations grouped into imaging, segmentation and tracking error categories across 9 severity levels (Methods). Imaging and segmentation errors are illustrated using frame 29 of 'base organoid' 1 as a reference image. Fragmented tracks are

represented by consecutive black squares in the horizontal direction. The ground truth dataset has 100% complete organoid tracks, 100% black squares. Each increasing severity level includes an additional track break, but overall temporal coverage (total black squares) is fixed at 50% (Methods). Organoid exemplar images are depicted with inverted intensity such that underexposure appears bright and overexposure appears dark. A non-inverted artefact level 9 exemplar image is shown for clarity. d) For each of blur, overexposure, underexposure and artefact, a single UMAP plot (n=97, 440) was generated to jointly analyze ground-truth and its perturbations, colored by perturbation level. After k-means clustering as in Fig. 2c, adjusted random index and mutual information was computed to assess phenotype clustering agreement between the ground truth and each perturbation level (Methods). e) UMAP for each segmentation perturbation are colored by perturbation severity level, showing that the ground truth can easily be isolated (black crop boxes), thereby enabling exclusion of both severe and subtle segmentation error manifestations. Reference images are depicted alongside each UMAP, with black lines locating each instance on the plot. f) Temporal phenotype trajectories of each condition, for ground-truth and all track fragmentation levels.

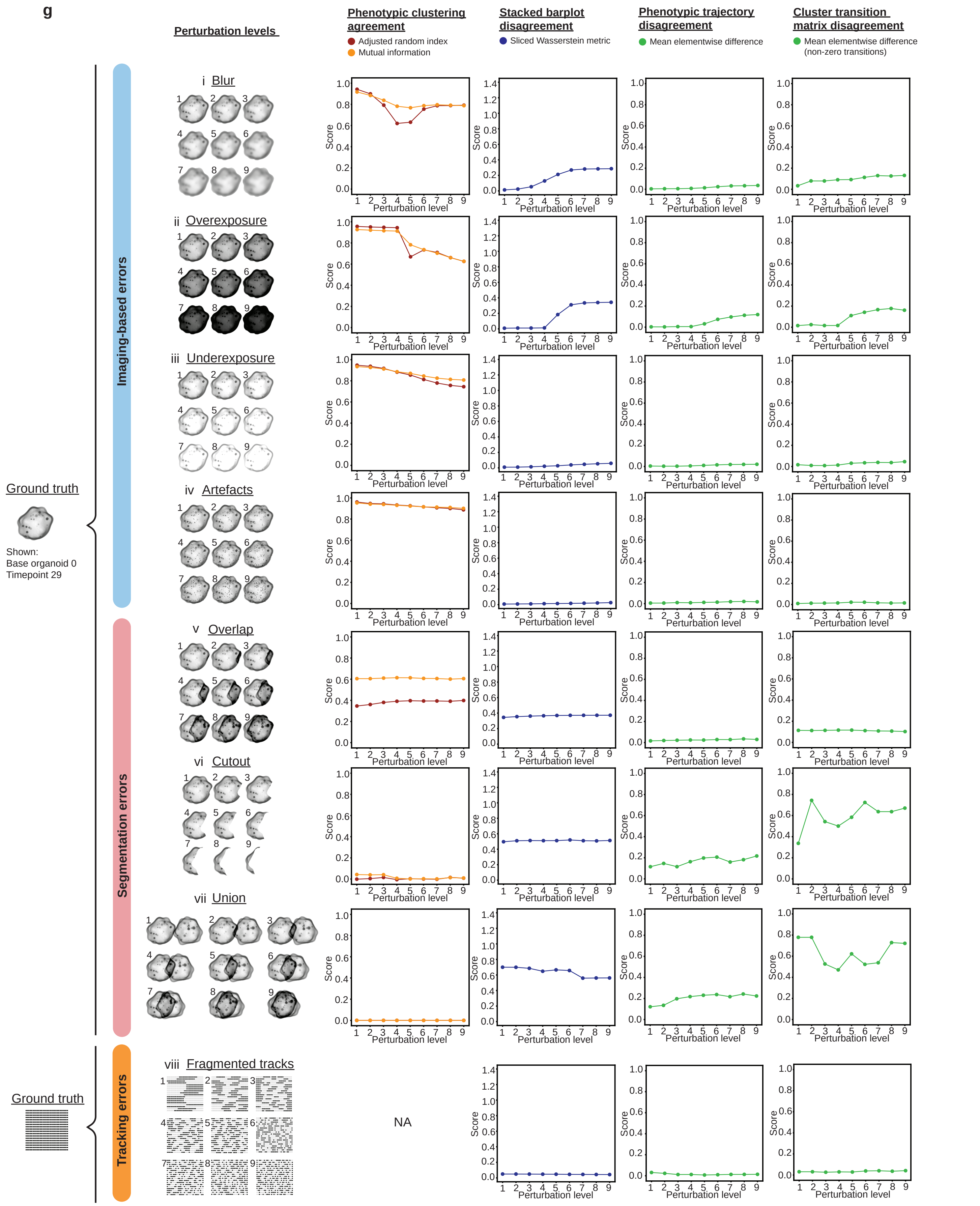

Supplementary Fig. 2 cont. Quantitative assessment of SPOT analysis robustness to imaging artefact, segmentation and tracking errors. g) Schematic of increasing severity level of perturbation, and phenotype clustering agreement, stacked barplot, phenotype trajectory and next timepoint cluster transition matrix disagreement metrics of ground-truth with each perturbation level

(left to right). For agreement metrics, the higher the value, the greater the concordance with ground-truth. For disagreement metrics, the lower the value, the greater the concordance with ground-truth. Multiple independent evaluation measures were used to evaluate phenotype clustering agreement. Sliced Wasserstein metric was used to evaluate stacked barplot disagreement. Mean elementwise difference was used to compute phenotype trajectory and next timepoint cluster transition matrix disagreements (Methods).

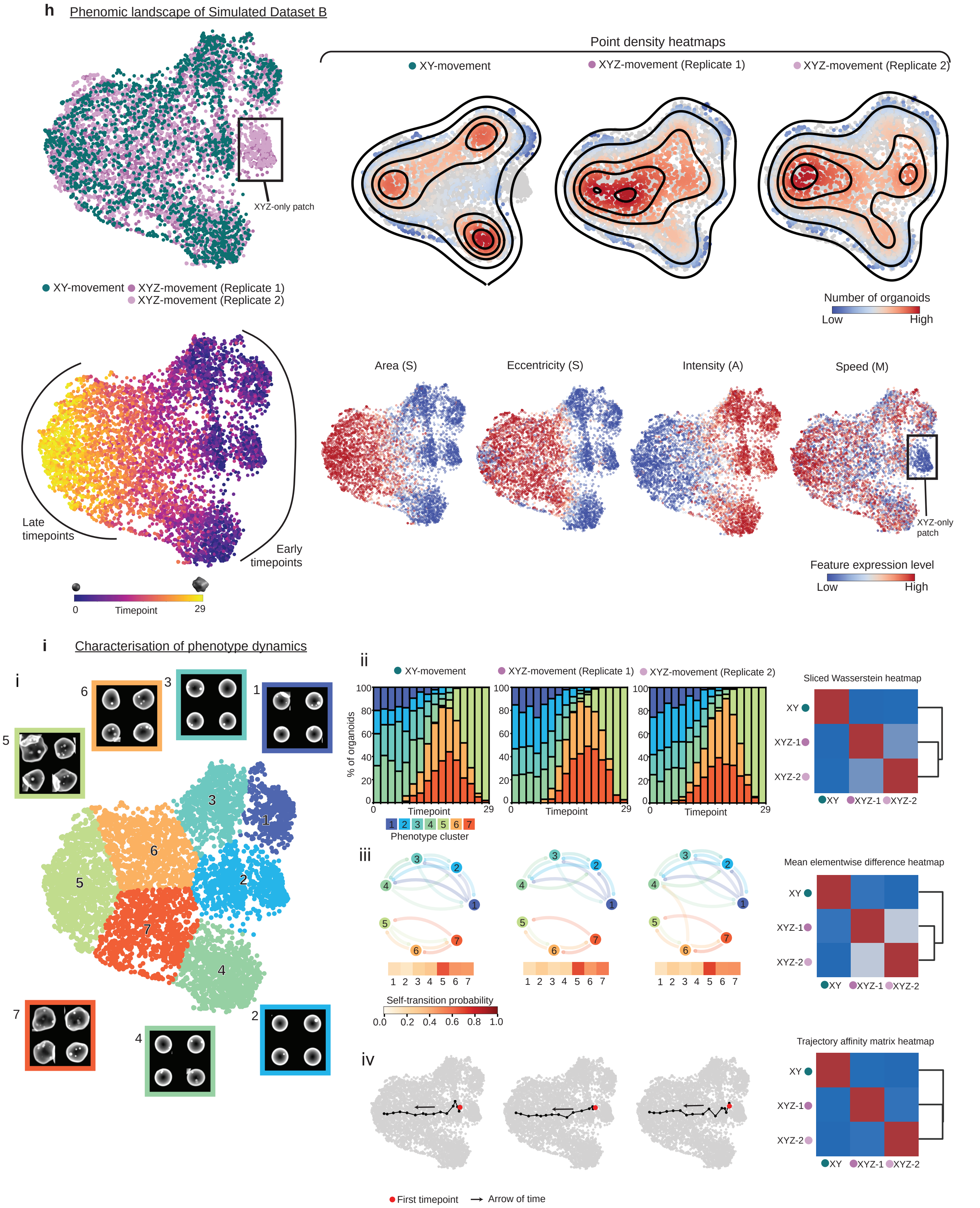

Supplementary Fig. 2 cont. SPOT analysis of 2D projections discriminates z-component motion. h) Phenomic landscape of Simulated Dataset B, colored by ground truth conditions (top left), density heatmaps (top right), timepoint in synthetic organoid evolution (bottom left) and selected individual features (area, eccentricity, entropy and mean speed; bottom right). Black boxes denote UMAP regions with no instances from the XY-movement condition. i) Partitioning of phenomic landscape into 7 clusters using k-means clustering (i), along with representative images for

each cluster. (ii) Stacked barplots show temporal changes in phenotype cluster distribution for each simulated organoid 3D movement condition, and pairwise computation of the max-sliced Wasserstein distance allow average-linkage hierarchical clustering to quantify stacked barplot similarity between conditions. (iii) Graphs depicting the next timepoint relative transition probabilities of changing to a different phenotype cluster (top, inter-cluster), and heatmap showing the next timepoint probability of staying in the same phenotype cluster (bottom, self-cluster). Arrow color represents the cluster of origin and its transparency reflect the probability magnitude – the more solid the color, the higher the probability. The mean elementwise difference between pairs of cluster transition matrices is computed for all condition permutations, and hierarchical clustering is performed using average linkage. (iv) Temporal phenotype trajectories show the progression of each simulated organoid 3D movement condition across the phenomic landscape over time. Red point indicates the origin of the trajectory at t=0. Trajectories are hierarchically clustered using average linkage.

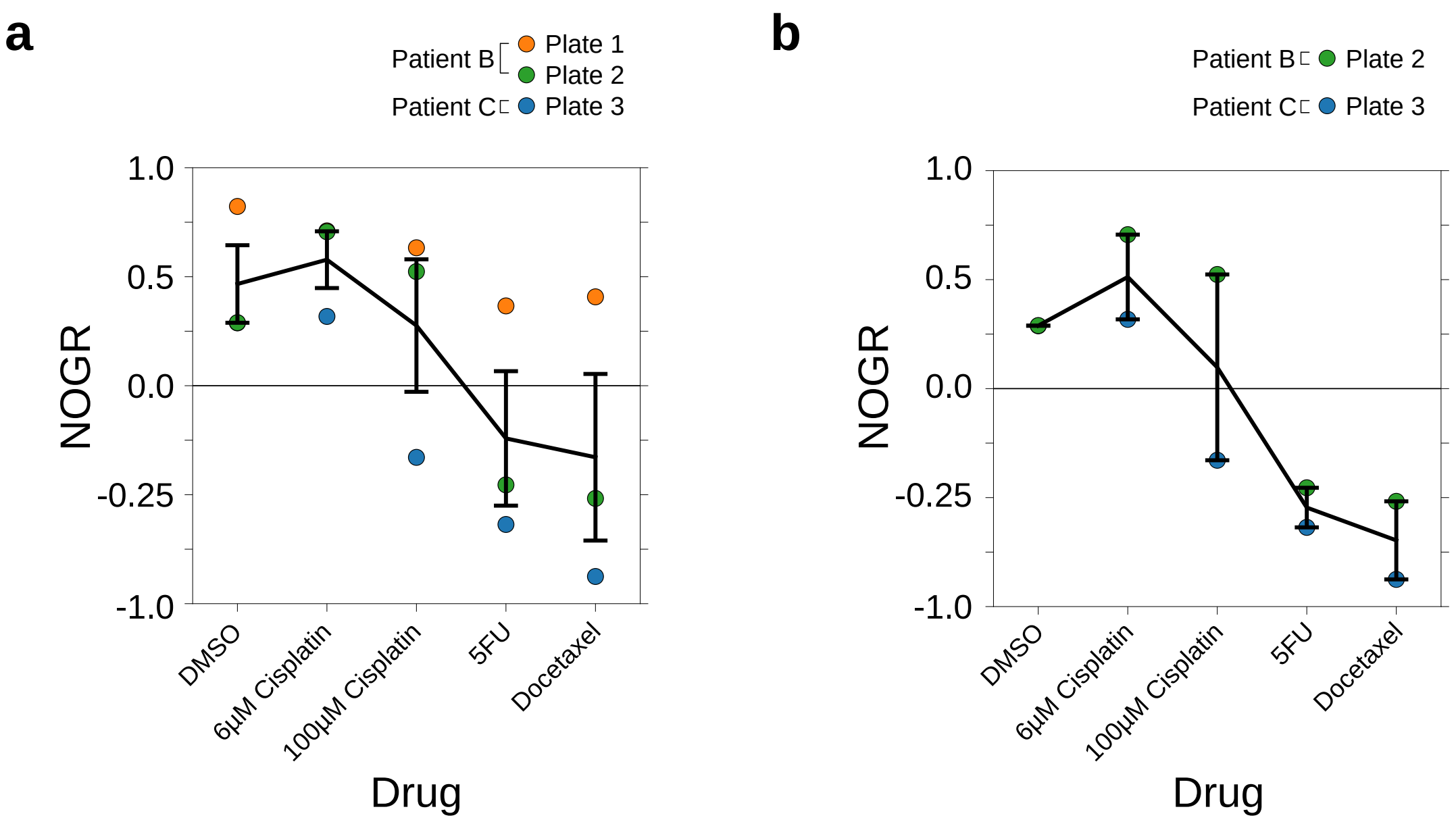

Supplementary Figure 3. Normalized Organoid Growth Rate (NOGR) validation of SPOT analysis. a) Median NOGR of all individual organoids per condition in each plate (n = 3 plates, m= 2 patients) (see Supplementary Table 3). b) Same as in a) but removing the outlier plate 2 organoids (n = 2 plates, m = 2 patients). Error bars represent mean±standard error of mean.

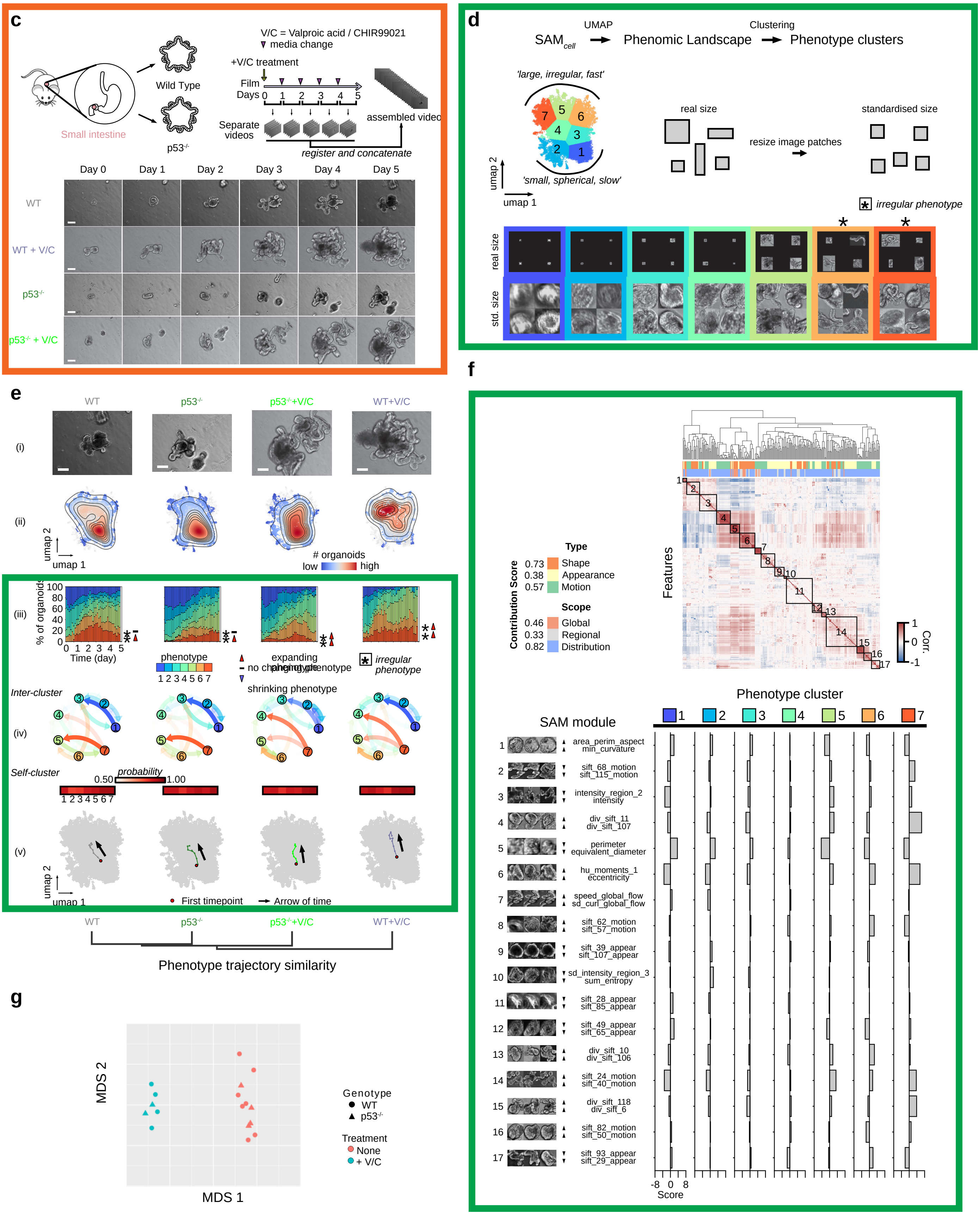

Supplementary Figure 3 cont. SPOT characterizes changes in murine small intestine organoid branching dynamics when treated with HDAC inhibitors. c) Small intestine organoids derived from WT p53+/+ and knockout p53-/- mice were grown in 24-well plates. The organoids were filmed

every 15 minutes for 5 days. Media was changed every day, resulting in five separate video stacks per well, which were then registered frame-by-frame and concatenated into one video for analysis (top right). Snapshots of an example video from each condition on days 0–5 (bottom). Scalebar: 50 μm. d) UMAP phenomic landscape, as in Fig. 3b, of filtered SAM phenomes from a total of 80 videos (20 per condition), 1-5 organoids in the field-of-view and carried out in 24-well plates, n=34,121 points. An asterisk indicates the 'irregular' branching-relevant clusters. e) Hierarchically clustered treatment conditions according to UMAP SAM temporal phenotype trajectories with complete linkage (bottom). Row (i): final frame of example videos in c) for each condition. Scalebar: 50 μm. Row (ii): smoothed UMAP local point density heatmaps. Row (iii): stacked barplots showing the temporal changes in the phenotype cluster distribution of segmented organoid instances in consecutive 3 hour time intervals for each indicated condition. An asterisk indicates the 'irregular', branching-relevant clusters. Row (iv): graph showing the next timepoint relative probability of transitioning to a different phenotype cluster (top) and heatmap showing the probability of staying in the same phenotype cluster, constructed as in Fig. 3c.Row (v): UMAP SAM temporal phenotype trajectories, constructed as in Fig. 3c. Each colored point on a trajectory denotes half-day increments from the starting point (red point) f) Contribution score of shape, appearance and motion, and global, regional and distributional features (left), automated hierarchical clustering to identify SAM feature modules (right) and SAM module expression (labeled 'score' in barplot) in each phenotype cluster (bottom) constructed as in Fig. 2f,g. g) Multidimensional scaling (MDS) plot of the log2(CPM) expression of bulk RNA-seq counts from WT p53+/+ (circles) and knockout p53-/- (triangles) organoids with no treatment harvested at day 0 or harvested at day 5 after V/C treatment (+V/C), where CPM stands for transcript counts per million.

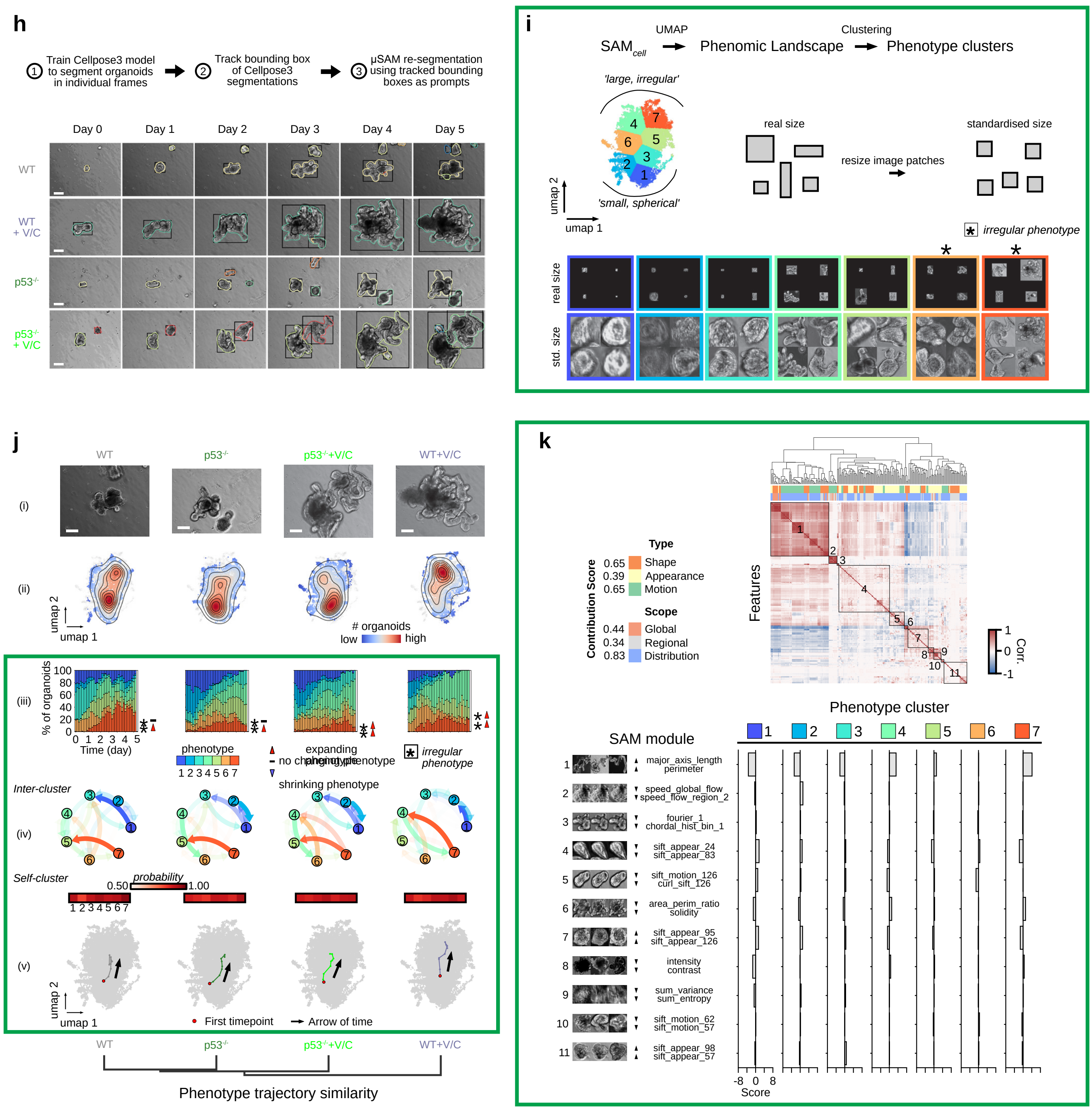

Supplementary Figure 3 cont. SPOT reanalysis of murine small intestine organoid branching dynamics with improved segmentation masks. h) Schematic of the improved segmentation workflow of the small intestine organoids derived from WT p53+/+ and knockout p53-/- mice treated with a combination of valproic acid (V) and CHIR99021 (C) (V/C treatment) analyzed in Supplementary Figure 3b (top). Resultant tracked bounding boxes (black) and outlines of μSAM re-segmented segmentations (uniquely colored) for same exemplar videos in Supplementary Figure 3b. Scalebar: 50 μm. i) UMAP phenomic landscape of filtered SAM phenomes based on the new segmentation masks. n=34,121 points. j) Hierarchically clustered treatment conditions according to UMAP SAM temporal phenotype trajectories with complete linkage (bottom). Row (i): final frame of example videos in a) for each condition. Scalebar: 50 μm. Row (ii): smoothed UMAP local point density heatmaps. Row (iii): stacked barplots showing the temporal changes in the phenotype cluster distribution of segmented organoid instances. Row (iv): graph showing the next timepoint relative probability of transitioning to a different phenotype cluster (top) and heatmap showing the probability of staying in the same phenotype cluster. Row (v): UMAP SAM temporal phenotype trajectories. k) Contribution score of shape, appearance and motion, and global, regional and distributional features

(left), automated hierarchical clustering to identify SAM feature modules (right) and SAM module expression (labeled ‘score’ in barplot) in each phenotype cluster (bottom). Panels i-k constructed as in Supplementary Figure 3d-f.

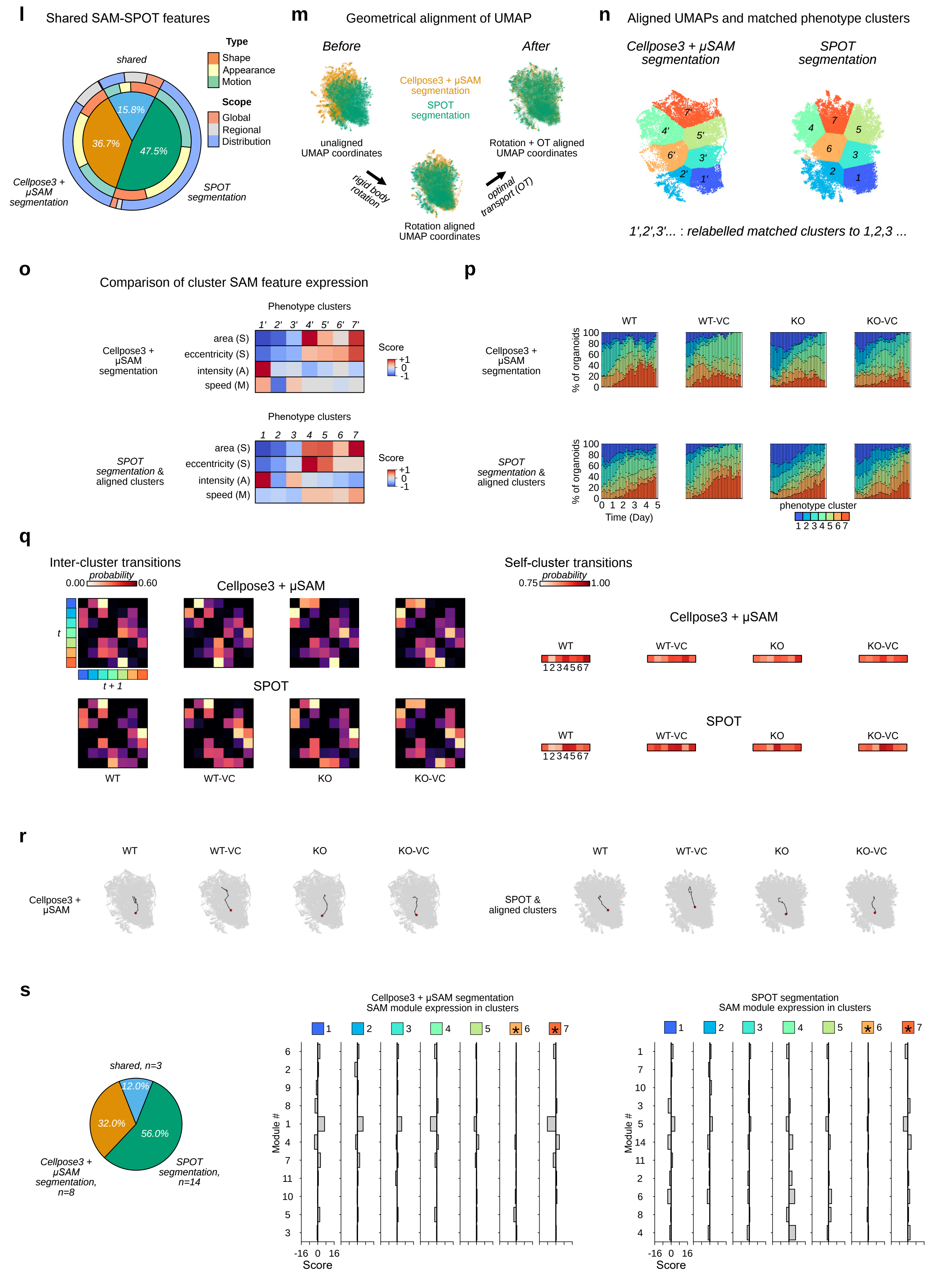

Supplementary Figure 3 contd. Reproducibility analysis of SPOT analyzed murine small intestine organoid branching in Supplementary Figure 3c-k with default and improved segmentation masks. l) Pie chart of the proportion of features that are unique or shared in the

two sets of filtered SPOT-SAM phenomes. Outer two rings illustrate the composition of shape, appearance and motion features, and global, regional and distribution features in each of the unique and shared fractions. m) Geometrical alignment by rigid body rotation and optimal transport of the two independent UMAP phenomic landscapes. n) Relabeling of the phenotype clusters of the SPOT analyzed Supplementary Figure 3h-k (Cellpose3 + μSAM segmentation) after matching the clusters to those of Supplementary Figure 3c-f (SPOT segmentation) by their median UMAP coordinates. The relabeled integer cluster IDs are denoted by an additional dash ('). o) Heatmap of the normalized mean expression score of selected SAM characteristics in each matched phenotype cluster for the Cellpose3 + μSAM segmentation (top) and SPOT segmentation (bottom) analysis. p) Stacked barplots of matched phenotype clusters. q) Inter-cluster (left) and self-cluster (right) transition probabilities depicted as a heatmap with matched phenotype clusters for each drug condition. r) Phenotypic trajectory for each drug condition after UMAP alignment for Cellpose3 + μSAM segmentation (left) and SPOT (right) segmentation. s) SAM module analysis with phenotype cluster alignment. Pie chart of the proportion of SAM modules found that are unique or shared (left). Shared is defined as the matched modules sharing more than a quarter of the same features. Mean expression of matched SAM modules in each of the matched phenotype clusters between the two analyses. SAM modules are ordered top-to-bottom by the extent of shared features in the Cellpose3 + μSAM segmentation (middle), and SPOT segmentation (right) based SPOT analyses. SAM module numbers correspond to those in the respective original SPOT analyses.

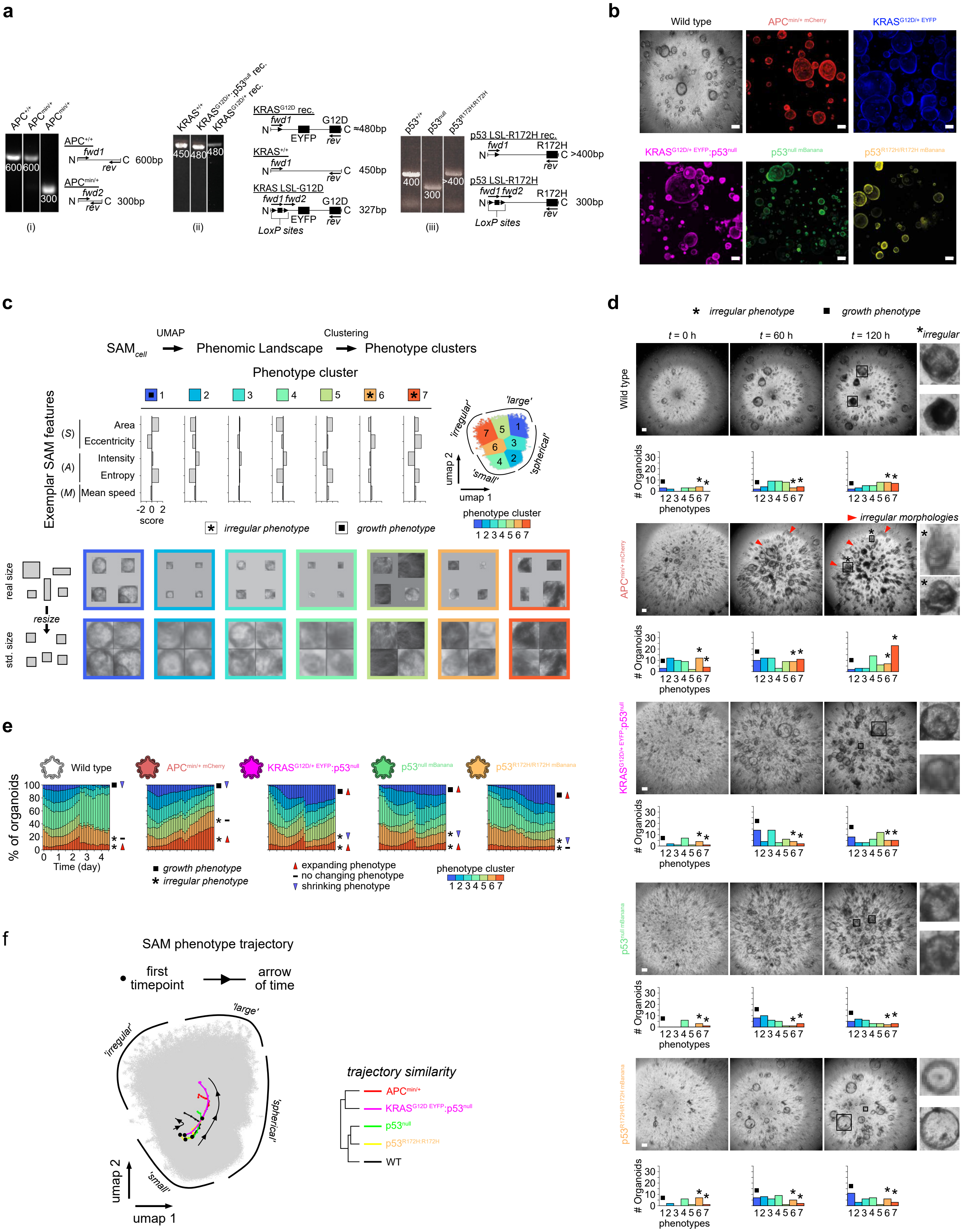

Supplementary Figure 4. SPOT draws the same conclusions with label-free imaging of genotype defined murine colon organoids. a) PCR validation of mutant colon organoid construction for (i) APCmin/+, (ii) KRASG12D/+ and KRASG12D/+:p53null and (iii) p53null and p53R172H:R172H. For each, the PCR gel with band base pair lengths labelled (left) and schematic of the primer design (right). See Supplementary Table 4 for primer sequences. b) Brightfield and confocal microscopy snapshots confirming fluorescent protein expression and correct organoid phenotype at day 7. Scalebars: 100 μm. c) K-means clustering of UMAP phenomic landscape of filtered SAM phenomes

from 98 phase contrast videos, two independent experiments with organoids embedded in 10 µL Matrigel droplets in 96-well plates, total n=626,083 points, constructed as in Figure 3b. Barplots of mean SPOT score of representative, global SAM features for each k-means phenotype cluster (top left) and top four image exemplars below arranged as a 2x2 grid in both real and standardized (std.) sizes to illustrate the difference in scale and appearance of organoids across clusters (bottom). Irregular phenotypes are indicated by an asterisk and growth phenotypes are indicated by a black square. d) Example video snapshots of the phase contrast timelapse of wild type and mutant organoids. For each genotype, upper: the extended focus projection used for analysis, scalebar: 200µm; lower: number (#) of organoids classified as each of the seven k-means phenotype clusters of c). More Organoids outlined in images by a black square are digitally enlarged and displayed on the right. Growth and irregular phenotypes are indicated as in c). e) Stacked barplot per genotype of the fraction of organoid instances in each phenotype cluster within consecutive 3 hour time intervals covering the 4.5 day acquisition. Beyond 2.5 days there is a discontinuous shift in cluster frequencies across most genotypes due to excess organoid death. f) UMAP SAM temporal phenotype trajectory of organoid growth for 0–2.5 days of e). Each colored point denotes a 6 hour increment from the starting point (black point) on each trajectory. Average linkage hierarchical clustering of the depicted phenotype trajectories (right).

g

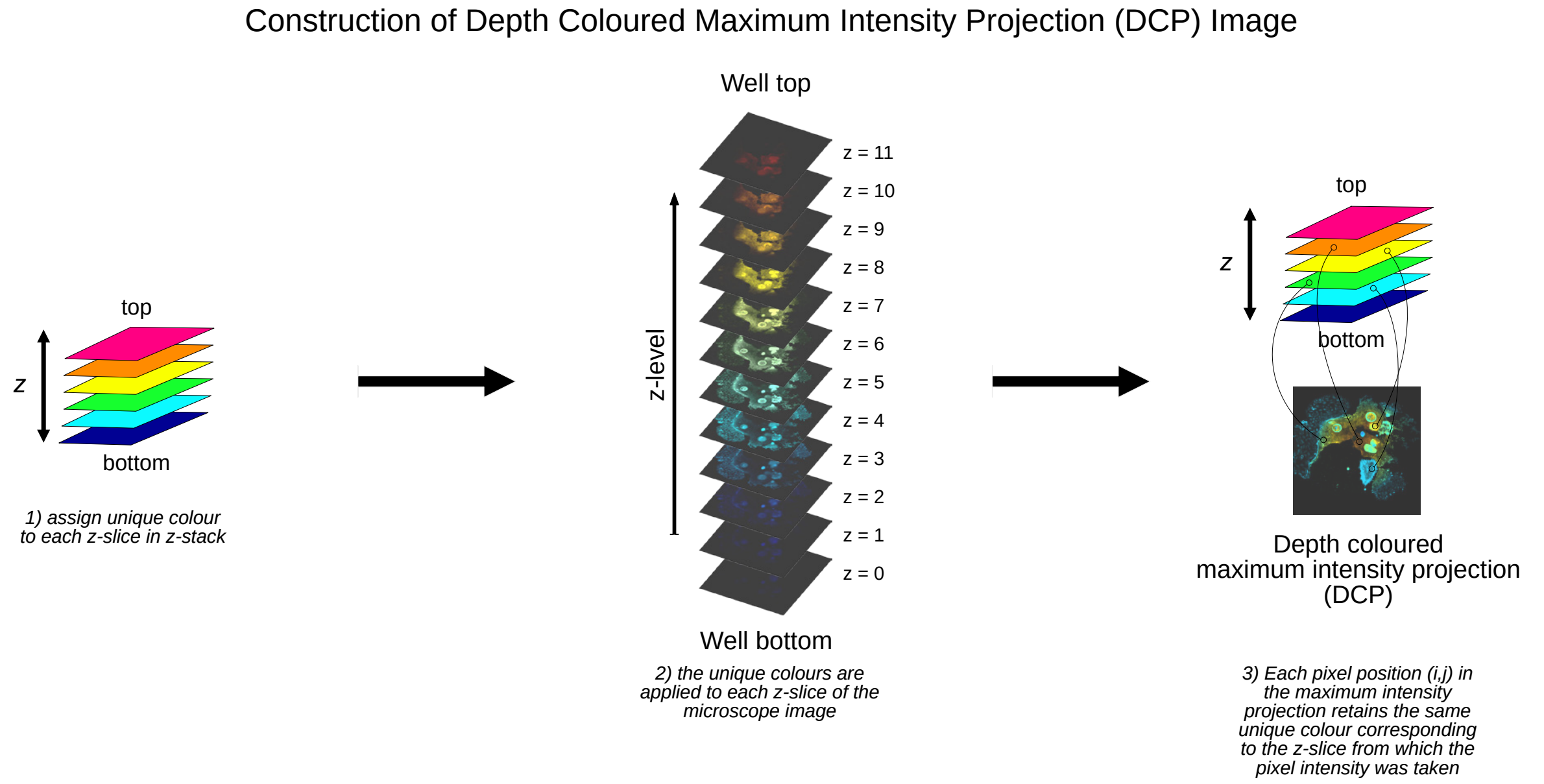

h

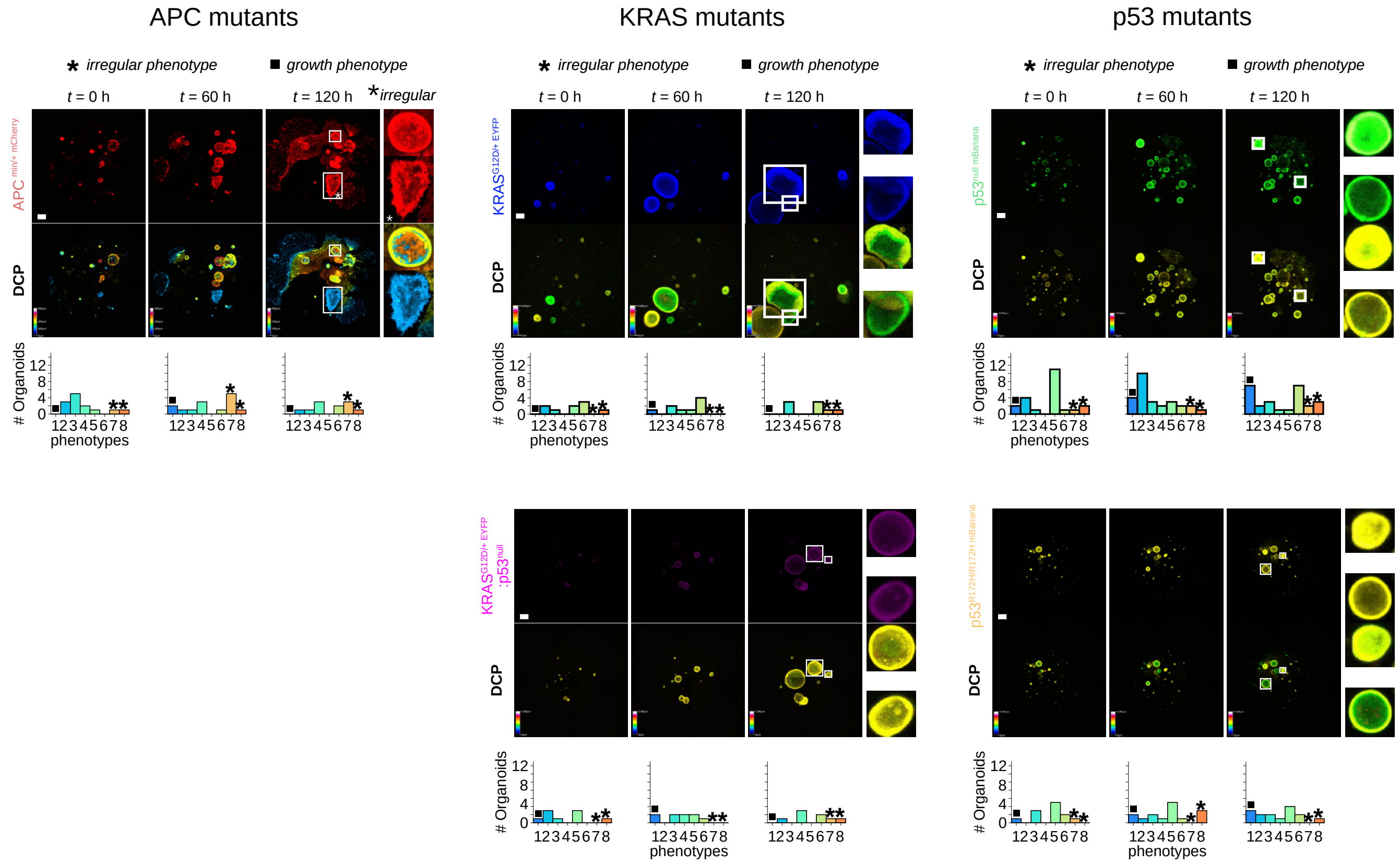

Supplementary Figure 4 cont. Murine colon organoids exhibit flattened morphologies preferentially at boundary of ECM dome. g) Schematic of computing a depth colored z-stack and its maximum intensity projection 2D image (DCP) to depict depth position of organoids in well. h) Representative DCP of mutant murine colon organoids at initial (t=0h), intermediate (t=60h) and final (t=120h) timepoints. For each mutant, top row: 2D projection image, middle row: corresponding DCP image, bottom row: phenotype cluster distribution of organoids in image. All scale bars: 200 µm.

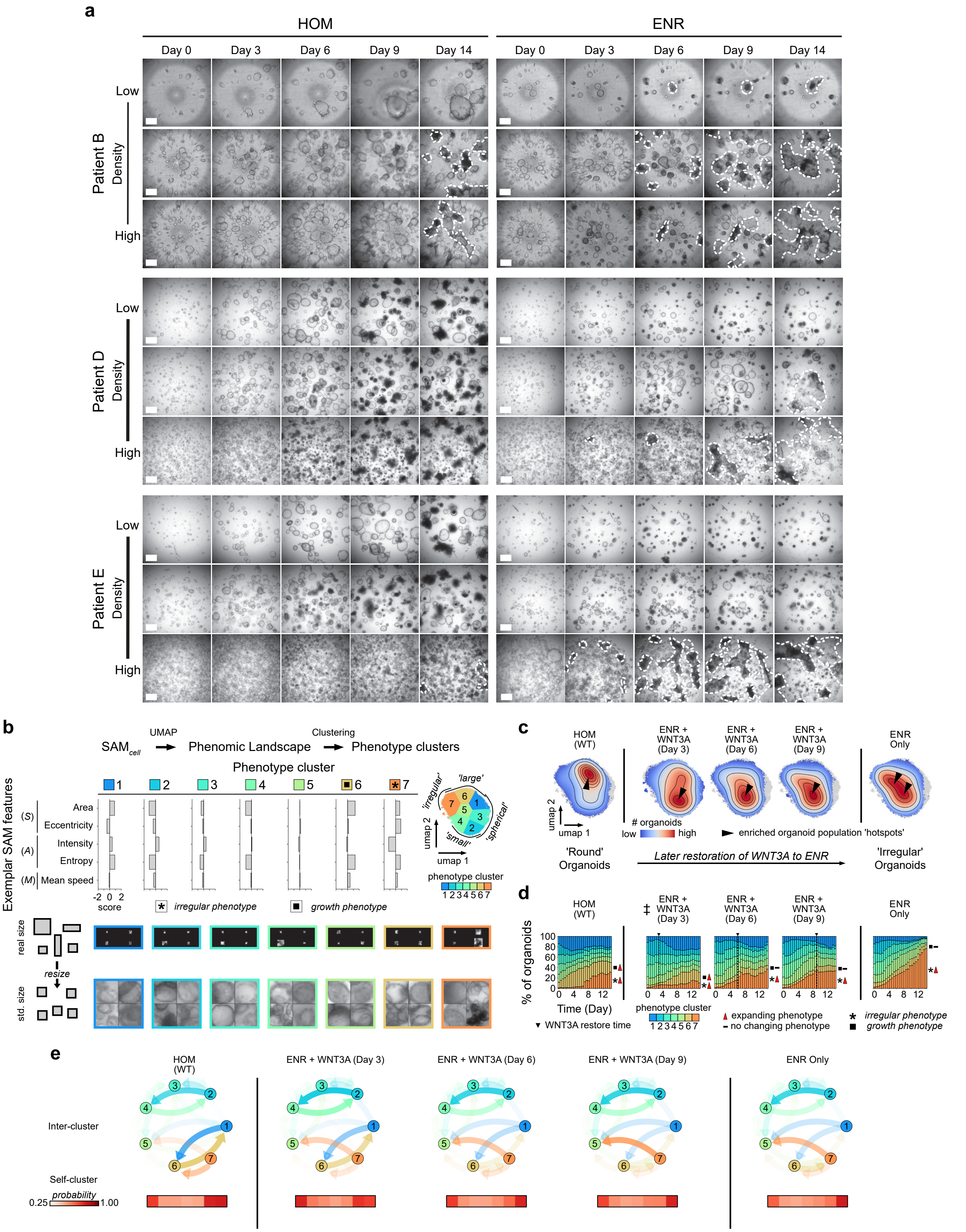

Supplementary Figure 5. SPOT detects changes in WNT signaling-induced phenotypic dynamics in patient-derived duodenum organoids. a) Brightfield microscopy snapshots of patient-derived duodenum organoids for three patients; patient B (top), patient D (middle) and patient E (bottom). For each patient, snapshots for low (top panel), medium (middle panel) and high (bottom panel) organoid densities and cultured in HOM (left panels, day 0–14) and ENR (right panels, day 0–14) with different confluencies. Scale bar: 200 μm. White dashed lines outline the morphologically

flattened organoids. The phase contrast filter was used for patient B and the brightfield filter was used for patient D and E's image collection. b) K-means clustering of UMAP phenomic landscape of filtered SAM phenomes from a total of 43 videos of organoids derived from three different patients, cultured and filmed in a 96-well plate, n=451,216 points (subsampled, every five organoid instances in the full compiled SAM table). Each point corresponds to a segmented organoid instance at a single timepoint. Corresponding barplots, representative image patches and UMAP phenomic landscape representations are constructed as in Fig. 4b. c) Smoothed UMAP local point density heatmaps, computed using all organoid instances in each respective culture condition. d) Stacked barplots showing the proportion of organoid instances in each phenotype cluster in b) over consecutive 12 hour intervals over the whole 0–14 day acquisition for each treatment condition. e) Graph showing the next timepoint relative transition probability to a different phenotype cluster (top), and heatmap showing the next timepoint transition probability to the same phenotype cluster (bottom) (Methods). Arrows are colored by the source cluster. The more transparent the arrow, the lower the probability of transition.

SUPPLEMENTARY TABLES

Supplementary Table 1. Cell lines and media requirements used for producing organoid conditioned media.

Cell line	Medium and Supplements	Selectable Antibiotic
HEK293T Rspo1-Fc Cells (provided by the laboratory of Prof. Gijs van den Brink)	Advanced DMEM/F-12<br>5% FBS<br>1% 2mM L-Glutamine<br>2 µL/mL 1M HEPES	0.3 mg/mL Zeocin (ThermoFisher Scientific/R25001)
HEK293T Nog-Fc Cells (provided by the laboratory of Prof. Calvin Kuo, Stanford University)	Advanced DMEM/F-12<br>5% FBS<br>1% 2mM L-Glutamine<br>2 µL/mL 1M HEPES	10 µg/mL Puromycin (ThermoFisher Scientific/A1113803)
HEK293T Wnt3A-Luciferase Reporter Cells	DMEM<br>10% FBS<br>1% 2mM L-Glutamine<br>2 µL/mL 1M HEPES	0.4 mg/mL G418 (Sigma-Aldrich/A1720)
L Wnt3A Cells (CRL-2647™)	DMEM<br>5% FBS<br>1% 2mM L-Glutamine<br>2 µL/mL 1M HEPES	0.4 mg/mL G418 (Sigma-Aldrich/A1720)

Supplementary Table 2. Constituents of the human and mouse organoid media and reagent concentrations. * denotes those constituents used in mouse organoid medium only, # denotes those constituents excluded from $APC^{min/+}$ mouse organoid medium, ^ denotes constituents included in $p53^{R172H/R172H}$ mouse organoid medium only.

Constituent	Company/Catalogue Number	Final Concentration
A83-01	StemCell Technologies/72024	500 nM
Antibiotic Antimycotic Solution (*)	Sigma-Aldrich/A5955	1X
B27 (*)	ThermoFisher Scientific/17504001	1X
CHIR99021 (#)	StemCell Technologies/72054	3 µM

EGF (*)	ThermoFisher Scientific/PHG0311	50 ng/mL
FGF10	Peprotech/100-26-25	100 ng/mL
Gastrin	Sigma-Aldrich/G9145	10 nM
N2 (*)	ThermoFisher Scientific/17502001	1X
N-Acetyl-Cysteine	Sigma-Aldrich/A9165-5G	1 mM
Nicotinamide	Sigma-Aldrich/N3376	10 mM
Noggin (*)	Conditioned Medium	20% of final volume
Nutlin-3 (^)	Merck Lifesciences/ N6287	5 μM
Penicillin/Streptomycin	Thermo Fisher Scientific/15140122	200 units/mL
R-Spondin (*,#)	Conditioned Medium	30% of final volume
SB202190	StemCell Technologies/72632	10 μM
Wnt3A (*, #)	Conditioned Medium	50% of final volume
Y-27632	StemCell Technologies/72308	10 μM

Supplementary Table 3. Plate types and live cell imaging acquisition details for organoid experiments

Figure	Plate	Patient ID	Plate number and IDs in figure (if applicable)	Z-stack	Wells per condition	Organoid statistics from SPOT analysis	Organoid statistics from scRNAseq analysis
Figure 2a-g, Supplementary Figure 2a,b	n/a	n/a	n/a	n/a	n/a	n=9,744 instances, m=336 tracks, number of individual organoids= 336	n/a
Figure 2h, Supplementary Figure 2c-g	n/a	n/a	n/a	n/a	n/a	Blur, overexposure, underexposure, artefact, overlap, and cutout perturbation conditions: n=97,440 instances, m=3,360 tracks, number of individual organoids= 3,360<br><br>Union perturbation condition: n=53,592 instances, m=1,848 tracks, number of overlapped organoids= 1,848<br><br>Track fragmentation perturbation condition (per	n/a

						fragmentation level 0-9): 0: n=9,744 instances, m=336 tracks, number of individual organoids= 336 1: n=4,900 instances, m=336 tracks, number of individual organoids= 336 2: n=4,816 instances, m=672 tracks, number of individual organoids= 336 3: n=4,893 instances, m=672 tracks, number of individual organoids= 336 4: n=4,781 instances, m=1,323 tracks, number of individual organoids= 336 5: n=4,781 instances, m=1,624 tracks, number of individual organoids= 336 6: n=4844 instances, m=1,918 tracks, number of	

						individual organoids= 336 7: n=4,872 instances, m=2,240 tracks, number of individual organoids= 336 8: n=4,802 instances, m=2,527 tracks, number of individual organoids= 336 9: n=4,816 instances, m=2,863 tracks, number of individual organoids= 336	
Supplementary Figure 2h,i	n/a	n/a	n/a	n/a	Scenes per condition=6 Organoids per scene=16	Pre-sub-setting: n=8,325 instances, m=288 tracks, number of individual organoids= 288 Post-sub-setting: n=6,182, m=n/a, number of individual organoids= 288	n/a
Figure 3	Corning® Costar® TC-Treated Multiple Well Plates	Patient A	1 plate (96-well)	Z-stack bright-field images covering 1000 µm total depth were acquired at 100	5-Fluorouacil: 9 Irinotecan: 9 Oxaliplatin: 9 Cisplatin: 9 Docetaxel: 9 Epirubicin: 9	n=516,503 instances; m=7,898 tracklets	n/a

				µm intervals (11 steps per acquisition) every hour for 5 days.	Mitomycin C: 9<br>Gefitinib: 9<br>DMSO: 24		
Figure 4	384-well plate, Flat bottom, rounded square wells	Mouse	5 plates (384-well)	Z-stack images 1225 µm in depth were acquired at 35 µm intervals (35 steps per acquisition) every 2 hours for 5 days at each of the chosen positions.	**Plate 1:**<br>$APC^{min/+}$: 5;<br>$KRAS^{G12D/+}$: 0 ;<br>$KRAS^{G12D/+}$: $p53^{null}$: 4 ;<br>$p53^{null}$: 5;<br>$p53^{R172H/R172H}$: 5<br>**Plate 2:**<br>$APC^{min/+}$: 6;<br>$KRAS^{G12D/+}$: 0 ;<br>$KRAS^{G12D/+}$: $p53^{null}$: 7 ;<br>$p53^{null}$: 7;<br>$p53^{R172H/R172H}$: 0<br>**Plate 3:**<br>$APC^{min/+}$: 7;<br>$KRAS^{G12D/+}$: 0 ;<br>$KRAS^{G12D/+}$: $p53^{null}$: 5 ;<br>$p53^{null}$: 7;<br>$p53^{R172H/R172H}$: 6<br>**Plate 4:**<br>$APC^{min/+}$: 5;<br>$KRAS^{G12D/+}$: 12 ;<br>$KRAS^{G12D/+}$: $p53^{null}$: 3 ;<br>$p53^{null}$: 4;<br>$p53^{R172H/R172H}$: 4<br>**Plate 5:**<br>$APC^{min/+}$: 0;<br>$KRAS^{G12D/+}$: 9 ;<br>$KRAS^{G12D/+}$: $p53^{null}$: 0 ;<br>$p53^{null}$: 0;<br>$p53^{R172H/R172H}$: 0	n=79,662 instances; m=1,850 tracklets<br><br>For panel a) errorbar plots, n number of instances for each time interval are:<br><br>$APC^{min/+}$: 615, 632, 634, 637, 637, 633, 630, 633, 633, 632, 629, 629, 622, 614, 613, 601, 590, 577, 570, 565, 565, 555, 549, 541, 535, 514, 509, 503, 498, 496, 501, 504, 504, 510, 509, 505<br><br>$KRAS^{G12D/+}$: 308, 318, 321, 321, 321, 316, 316, 317, 316, 314, 310, 300, 294, 296, 294, 289, 287, 281, 277, 274, 272, 264,	n/a

						264,<br>252, 251,<br>245, 244,<br>242, 242,<br>241, 244,<br>245, 250,<br>253,<br>250, 253<br><br>KRAS$^{G12D/+}$: p53^{null}: 463,<br>473, 477,<br>481, 480,<br>476, 470,<br>464, 458,<br>455, 455,<br>443,<br>440, 438,<br>435, 429,<br>424, 421,<br>419, 419,<br>417, 413,<br>412,<br>413, 414,<br>410, 403,<br>397, 391,<br>388, 389,<br>390, 390,<br>396,<br>396, 392<br><br>p53^{null}:<br>1066, 1118,<br>1145, 1187,<br>1202, 1234,<br>1242, 1262,<br>1267, 1277,<br>1280,<br>1294,<br>1302, 1307,<br>1306, 1301,<br>1293, 1289,<br>1288, 1279,<br>1277, 1279,<br>1278,<br>1276, 1272,<br>1265, 1263,<br>1258, 1248,<br>1242, 1243,<br>1241, 1240,<br>1246,<br>1248, 1248<br><br>p53$^{R172H/R172H}$: 356, 362,<br>364, 367,	

						369, 372, 373, 376, 375, 373, 372, 375, 377, 376, 374, 370, 370, 371, 372, 374, 378, 380, 380, 381, 380, 380, 380, 375, 374, 376, 376, 376, 376, 376, 376, 376	
Figure 5	Nunc Micro Well 96-Well Optical-Bottom Plates with Polymer Base, Cell culture, black	Patient B, Patient D, Patient E	1 plate	Z-stack images 1000 µm in depth were acquired at 100 µm intervals (11 steps per acquisition) approximately every hour for 14 days.	For each patient: HOM: 3 wells ENR+WNT 3: 3 wells ENR+WNT 6: 3 wells ENR+WNT 9: 3 wells ENR only: 3 wells	n=451,216 instances; m=22,856 tracklets	n/a
Figure 6a-e	384-well plate, Flat bottom, rounded square wells	Mouse	-	n/a	An appropriate number of wells were pooled to produce a cell suspension of $1x10^6$ cells/mL	n/a	n/a
Figure 6f-g	Corning™ Costar™ 24-well Clear TC-treated Multiple Well Plates, Individually Wrapped, Sterile	Patient D	-	n/a	An appropriate number of wells were pooled to produce a cell suspension of $1x10^6$ cells/mL	n/a	n/a

Supplementary Figure 1a-c	Nunc Micro Well 96-Well Optical-Bottom Plates with Polymer Base, Cell culture, black	Patient B	n/a	Z-stack images 1000 µm in depth were acquired at 100 µm intervals (11 steps per acquisition) approximately every 60 min for 14 days.	1 well	n/a	n/a
Supplementary Figure 1d-f	384-well plate, Flat bottom, rounded square wells	Mouse	n/a	Z-stack images 1225 µm in depth were acquired at 35 µm intervals (35 steps per acquisition) every 2 hours for 5 days at each of the chosen positions.	1 well	n/a	n/a
Reviewer Supplementary Figure 3a	Corning® Costar® TC-Treated Multiple Well Plates	Patient A	1 plate (96-well) (subset of Fig.3)	Z-stack bright-field images covering 1000 µm total depth were acquired at 100 µm intervals (11 steps per acquisition) every 4 hours	5-Fluorouacil: 9<br>Cisplatin: 9<br>Docetaxel: 9<br>DMSO: 24	n=281,600 instances; m=4,514 tracklets	n/a

				for 5 days.			
Reviewer Supplementary Figure 3b	Nunc Micro Well 96-Well Optical-Bottom Plates with Polymer Base, Cell culture, black	Patient B	2 plates (96-well): G_pB_wo/bg_r1, G_pB_wo/bg_r2	Z-stack bright-field images covering 1000 µm total depth were acquired at 100 µm intervals (11 steps per acquisition) every 4 hours for 5 days.	5-Fluorouacil: 3<br>6µM Cisplatin: 3<br>100µM Cisplatin: 3<br>Docetaxel: 3<br>DMSO: 3<br>for each of caspase and cytotox stains	n=70,757 instances; m= 2,574 tracklets	n/a
Reviewer Supplementary Figure 3c (identical dataset to Supplementary Fig 3c-g)	Nunc Micro Well 96-Well Optical-Bottom Plates with Polymer Base, Cell culture, black	Patient B	2 plates (96-well): G_pB_wo/bg_r1, G_pB_wo/bg_r2	Z-stack bright-field images covering 1000 µm total depth were acquired at 100 µm intervals (11 steps per acquisition) every 4 hours for 5 days.	5-Fluorouacil: 3<br>6µM Cisplatin: 3<br>100µM Cisplatin: 3<br>Docetaxel: 3<br>DMSO: 3<br>for each of caspase and cytotox stains	n=67,703 instances; m= 2,410 tracklets	n/a
Reviewer Supplementary Figure 3d-e	Comparative analysis of results in Reviewer Supplementary Figure 3b and Reviewer Supplementary Figure 3c						
Reviewer Supplementary Figure 3f-h	Nunc Micro Well 96-Well Optical-Bottom Plates with Polymer Base, Cell	Patient C	1 plate (96-well plate) (G_pC_w/bg_r1)	Z-stack bright-field images covering 1000 µm total depth	5-Fluorouacil: 3<br>6µM Cisplatin: 3<br>100µM Cisplatin: 3	n= 40,416 instances; n= 39,057 instances (after artefact removal);	n/a

	culture, black			were acquired at 100 µm intervals (11 steps per acquisition) every 4 hours for 5 days.	Docetaxel: 3 DMSO: 3 for each of caspase and cytotox stains	m=1,592 tracklets	
Reviewer Supplementary Figure 3i-k	Comparative analysis of results in Reviewer Supplementary Figure 3b and Reviewer Supplementary Figure 3h						
Reviewer Supplementary Figure 3l-o	Corning® Costar® TC-Treated Multiple Well Plates (plastic-bottom) Nunc MicroWell 96-Well Optical-Bottom Plates with Polymer Base, Cell culture, black (glass-bottom)	Patient B	Plastic-bottom: 2 plates (P_pB_wo/bg_r1, P_pB_w/bg_r2) Glass-bottom: 3 plates (G_pB_w/bg_r1, G_pB_w/bg_r2, G_pB_w/bg_r3)	Z-stack bright-field images covering 1000 µm total depth were acquired at 100 µm intervals (11 steps per acquisition) every 4 hours for 5 days.	5-Fluorouacil: 3 6µM Cisplatin: 3 100µM Cisplatin: 3 Docetaxel: 3 DMSO: 3	Plastic-bottom: n=55,581 instances; m=2,402 tracklets<br><br>Glass-bottom: n=53,078 instances; m=2,461 tracklets	n/a
Reviewer Supplementary Figure 3p-s	Pooled SPOT analysis of all plates from Reviewer Supplementary Figure 3b-o						
Supplementary Figure 3a-b	Nunc MicroWell 96-Well Optical-Bottom Plates with Polymer Base, Cell culture, black	Patient B; Patient C	2455: 2 plates (96-well): (G_pB_wo/bg_r1, G_pB_wo/bg_r2)<br><br>4139: 1 plate (96-well): (G_pC_w/bg_r1)	Z-stack bright-field images covering 1000 µm total depth were acquired at 100 µm intervals	5-Fluorouacil: 3 6µM Cisplatin: 3 100µM Cisplatin: 3 Docetaxel: 3 DMSO: 3 for each of caspase and cytotox	Patient B (2 plates): n=70,757 instances; m= 2,574 tracklets<br><br>Patient C (1 plate): n= 40,416 instances; n= 39,057 instances	n/a

				(11 steps per acquisition) every 4 hours for 5 days.	stains, and each patient	(after artefact removal); m=1,592 tracklets	
Supplementary Figure 3c-g	[Corning® Costar® TC-Treated Multiple Well Plates]()	Mouse	1 plate (96-well)	Z-stack images of 1000 µm in depth were acquired at 100 µm intervals (11 steps per acquisition) approximately every 15 minutes for 5 days.	WT: 20<br>WT-VC: 20<br>KO: 20<br>KO-VC: 20	n=68,243 (all timepoints); n=34,121 instances (after halving timepoints); m=567 tracklets	n/a
Supplementary Figure 3h-k (resegment and tracked of Supplementary Figure 3c-g)	[Corning® Costar® TC-Treated Multiple Well Plates]()	Mouse	1 plate (96-well)	Z-stack images of 1000 µm in depth were acquired at 100 µm intervals (11 steps per acquisition) approximately every 15 minutes for 5 days.	WT: 20<br>WT-VC: 20<br>KO: 20<br>KO-VC: 20	n=55,380 (all timepoints); n=27,690 instances (after halving timepoints); m=677 tracklets	n/a
Supplementary Figure 3l-s	Comparative analysis of results in Reviewer Supplementary Figure 3t and Reviewer Supplementary Figure 3u						
Supplementary Figure 4c-f	[Corning® Costar® TC-Treated]()	Mouse	2 plates (384-well)	Z-stack bright-field images	**Plate 1:**<br>WT: 4;<br>$APC^{min/+}$: 9;<br>$KRAS^{G12D/+}$:	n= 626,083 instances; m=6,528 tracklets	n/a

	Multiple Well Plates			covering 1000 µm total depth were acquired at 100 µm intervals (11 steps per acquisition) every 4 hours for 5 days.	0; $KRAS^{G12D/+}$: $p53^{null}$: 10; $p53^{null}$: 9; $p53^{R172H/R172H}$: 9 **Plate 2:** WT: 20; $APC^{min/+}$: 10; $KRAS^{G12D/+}$: 0; $KRAS^{G12D/+}$: $p53^{null}$: 9; $p53^{null}$: 10; $p53^{R172H/R172H}$: 8		
Supplementary Figure 4g-h (same dataset as Fig. 4)	384-well plate, Flat bottom, rounded square wells	Mouse	-	Z-stack bright-field images covering 1000 µm total depth were acquired at 100 µm intervals (11 steps per acquisition) every 4 hours for 5 days.	1 well per condition	-	n/a
Supplementary Figure 5 (same dataset as Fig 5)	Nunc Micro Well 96-Well Optical-Bottom Plates with Polymer Base, Cell culture, black	Patient B, Patient D, Patient E	1 plate	Z-stack images 1000 µm in depth were acquired at 100 µm intervals (11 steps per acquisition) approximately every 60 min for 14 days.	For each patient: HOM: 3 wells ENR+WNT 3: 3 wells ENR+WNT 6: 3 wells ENR+WNT 9: 3 wells ENR only: 3 wells	n=451,216 instances; m=22,856 tracklets	n/a

Supplementary Table 4. PCR primers used to validate the genotype of murine mutant colon organoids.

Genotype	Sequence (5’ to 3’),<br>**F:** *forward*, **R:** *reverse*
APC wild type	F: GCC ATC CCT TCA CGT TAG
	R: TTC CAC TTT GGC ATA AGG C
APCmin	F: TTC TGA GAA AGA CAG AAG TTA
	R: TTC CAC TTT GGC ATA AGG C
KRAS G12D conditional allele	F: CTA GCC ACC ATG GCT TGA GT
	R: TCC GAA TTC AGT GAC TAC AGA TG
KRAS G12D recombined allele	F: ATG TCT TTC CCC AGC ACA GT
	R: TCC GAA TTC AGT GAC TAC AGA TG
p53 R172H conditional allele	F: AGC TAG CCA CCA TGG CTT GAG
	R: CTT GGA GAC ATA GCC ACA CTG A
p53 R172H recombined allele	F: CTG GGT CCA CCA TGG TGA TG
	R: CTT GGA GAC ATA GCC ACA CTG A
