# Supplementary material for: Identifying phenotype-genotype-function coupling in 3D organoid imaging using Shape, Appearance and Motion Phenotype Observation Tool (SPOT)
Source: Nat Commun. 2026 Aug 17;17:8410. doi: 10.1038/s41467-026-75506-7 (PMC13482367; doi:10.1038/s41467-026-75506-7)
Supplement: Supplementary file 2 [file 41467_2026_75506_MOESM2_ESM.pdf]

Inventory of Supplementary Files

SUPPLEMENTARY MOVIES

Supplementary Movie 1. Tracking and segmentation of patient-derived duodenum organoids treated with different chemotherapy drugs. One example video of organoids treated with each chemotherapy drug at the highest concentration and dimethyl sulfoxide (DMSO) control. For each treatment condition we firstly show the input spatiotemporally registered video; secondly, the bounding box detection and tracked individual organoids; and thirdly, the final filtered and segmented individual organoid boundaries. The current centroid of individual organoids is denoted by a white dot. Bounding box or contour color-matched dots denote historical centroid positions of the same organoid. The final boundaries remove all organoid instances near the border of the field of view to exclude partially segmented organoids from downstream phenotype analysis. Videos are organized such that the top and bottom row represent less toxic and more toxic drugs respectively, corresponding to the left and right clade of the hierarchical clustering in Fig. 3. Scalebar: 250 μm.

Supplementary Movie 2. Tracking and segmentation of wild type and $p53^{-/-}$ mouse small intestine organoids with and without V/C (valproic acid/CHIR99021) treatment. One example video of wild type ($p53^{+/+}$) and $p53^{-/-}$ organoid cultures, with and without V/C treatment. For each treatment we firstly show the input spatiotemporally registered video; secondly, the bounding box detection and tracked individual organoids; and thirdly, the final filtered and segmented individual organoid boundaries. The current centroid of individual organoids is denoted by a white dot. Bounding box or contour color-matched dots denote historical centroid positions of the same organoid. The final boundaries remove all organoid instances near the border of the field of view to exclude partially segmented organoids from downstream phenotype analysis. Scalebar: 50 μm.

Supplementary Movie 3. Tracking and segmentation of murine mutant colon organoids. One example video of organoid culture for each mutant genotype; $APC^{min/+\ mCherry}$, $KRAS^{G12D/+\ EYFP}$:$p53^{null}$, $KRAS^{G12D/+}$, $p53^{null\ mBanana}$, $p53^{R172H/R172H\ mBanana}$. For each mutant we firstly show the input spatiotemporally registered video; secondly, the bounding box detection and tracked individual organoids; and thirdly, the final filtered and segmented individual organoid boundaries. The current centroid of individual organoids is denoted by a white dot. Bounding box or contour color-matched dots denote historical centroid positions of the same organoid. The final boundaries remove all organoid instances near the border of the field of view to exclude partially segmented organoids from downstream phenotype analysis. Scalebar: 200 μm.

Supplementary Movie 4. Tracking and segmentation of patient-derived duodenum organoids in human organoid media (HOM) vs ENR media only and with WNT3A restoration, from three patients. One example video of organoid culture in normal human organoid (HOM), ENR only media, and ENR with WNT3A restoration after the specified number of days from the start of the timelapse for each of the three analyzed patients. For each patient, for each condition, we firstly show the input spatiotemporally registered video; secondly, the bounding box detection and tracked individual organoids; and thirdly, the final filtered and segmented individual organoid boundaries. The current centroid of individual organoids is denoted by a white dot. Bounding box or contour color-matched dots denote historical centroid positions of the same organoid. The final boundaries remove all organoid instances near the border of the field of view to exclude partially segmented organoids from downstream phenotype analysis. Scalebar: 200 μm.

SUPPLEMENTARY DATA

Supplementary Data 1. Metadata associated with simulated organoid datasets
