# Supplementary material for: Identifying phenotype-genotype-function coupling in 3D organoid imaging using Shape, Appearance and Motion Phenotype Observation Tool (SPOT)
Source: Nat Commun. 2026 Aug 17;17:8410. doi: 10.1038/s41467-026-75506-7 (PMC13482367; doi:10.1038/s41467-026-75506-7)
Supplement: Supplementary file 3 [file 41467_2026_75506_MOESM3_ESM.pdf]

nature portfolio

Corresponding author(s): Felix Zhou, Xin Lu

Last updated by author(s): 11/06/2026

Reporting Summary

Nature Portfolio wishes to improve the reproducibility of the work that we publish. This form provides structure for consistency and transparency in reporting. For further information on Nature Portfolio policies, see our Editorial Policies and the Editorial Policy Checklist.

Statistics

For all statistical analyses, confirm that the following items are present in the figure legend, table legend, main text, or Methods section.

n/a	Confirmed	
[ ]	[x]	The exact sample size (*n*) for each experimental group/condition, given as a discrete number and unit of measurement
[ ]	[x]	A statement on whether measurements were taken from distinct samples or whether the same sample was measured repeatedly
[x]	[ ]	The statistical test(s) used AND whether they are one- or two-sided<br>*Only common tests should be described solely by name; describe more complex techniques in the Methods section.*
[x]	[ ]	A description of all covariates tested
[x]	[ ]	A description of any assumptions or corrections, such as tests of normality and adjustment for multiple comparisons
[ ]	[x]	A full description of the statistical parameters including central tendency (e.g. means) or other basic estimates (e.g. regression coefficient) AND variation (e.g. standard deviation) or associated estimates of uncertainty (e.g. confidence intervals)
[x]	[ ]	For null hypothesis testing, the test statistic (e.g. *F*, *t*, *r*) with confidence intervals, effect sizes, degrees of freedom and *P* value noted<br>*Give P values as exact values whenever suitable.*
[x]	[ ]	For Bayesian analysis, information on the choice of priors and Markov chain Monte Carlo settings
[x]	[ ]	For hierarchical and complex designs, identification of the appropriate level for tests and full reporting of outcomes
[ ]	[x]	Estimates of effect sizes (e.g. Cohen's *d*, Pearson's *r*), indicating how they were calculated

Our web collection on statistics for biologists contains articles on many of the points above.

Software and code

Policy information about availability of computer code

Data collection	Phase-contrast timelapse microscopy and label-free timelapse microscopy data was collected using Molecular Devices MetaMorph 7.10.1 Timelapse confocal microscopy data was collected using Zeiss Zen Black v2012. Synthetic organoid datasets with defined phenotypes for benchmarking SPOT were simulated with custom Python code and made available in Zenodo (https://zenodo.org/records/14265519). The code is part of a future methods paper on organoid simulation. It can be provided on request to the lead contact.

Data analysis	Image Analysis: All experiments were analyzed using our custom developed SPOT library, which is available free for academic use at https://github.com/fyz11/SPOT and installable from the Python Package Index at https://pypi.org/project/SAM-SPOT. CellProfiler features were computed using CellProfiler v4.2.6 downloaded from https://cellprofiler.org/releases. Some image to video conversion was performed using FIJI, available at https://fiji.sc/. YOLOv3 (from http://github.com/AlexeyAB/darknet) model was trained to detect bounding boxes of individual organoids. To maintain Python compatibility, the SPOT library runs these trained weights using a Python Keras reimplementation of the YOLOv3 architecture.<br><br>Sequencing: Counts per million (cpm) were computed from the raw counts using edgeR (version 3.16.5) and R (version 3.3.3). Cell Ranger (v3.1.0) was used for pre-processing of scRNA-seq data.Initial downstream processing was conducted using Seurat (v3.9.9.9038). As an initial filter for low-quality cells, cellular barcodes with less than 2000 genes detected or more than 10% mitochondrial reads were removed. Normalization, variance-stabilizing transformation (VST), and scaling were conducted on the remaining cells using Seurat's implementation of sctransform (v0.3.2.9002), and the scaled VST counts for the 3000 most variable features were extracted from the Seurat object (scale.data slot). Sequence alignment used STAR and counts generated by Samtools.<br><br>Imaging: ImageJ (v1.5)

For manuscripts utilizing custom algorithms or software that are central to the research but not yet described in published literature, software must be made available to editors and reviewers. We strongly encourage code deposition in a community repository (e.g. GitHub). See the Nature Portfolio guidelines for submitting code & software for further information.

Data

Policy information about availability of data

All manuscripts must include a data availability statement. This statement should provide the following information, where applicable:

- Accession codes, unique identifiers, or web links for publicly available datasets
- A description of any restrictions on data availability
- For clinical datasets or third party data, please ensure that the statement adheres to our policy

Sequencing datasets used in this study are available from GEO: mutant mouse colon organoid scRNA-seq, accession number GSE218339, mouse small intestinal bulk RNA-seq, accession number GSE218337 and human small intestinal scRNA-seq, accession number pending.
Simulated organoid datasets A and B used in this study are available at DOI: 10.5281/zenodo.14265519.
The developed SPOT Python library used to detect, track and segment individual organoids and to compute and analyse the SAM phenome in this paper is available at GitHub, https://github.com/fyz11/SPOT, and from the Python Package Index, https://pypi.org/project/SAM-SPOT. The code is also archived on Zenodo, https://zenodo.org/records/20629367. Example datasets and scripts accompany the code for verification.
The size of raw datasets exceed public repository quotas. The lead contact will make these available upon request.

Research involving human participants, their data, or biological material

Policy information about studies with human participants or human data. See also policy information about sex, gender (identity/presentation), and sexual orientation and race, ethnicity and racism.

Reporting on sex and gender	NA
Reporting on race, ethnicity, or other socially relevant groupings	NA
Population characteristics	Patient A (Figure 3b, 3c, 3d, Reviewer Suppl Fig 3a) - Male, 65<br>Patient B (Figure 5, Suppl figure 5, Suppl Fig 1a-c, Suppl Fig 3a,b, Reviewer Suppl Fig 3b-c, 3l-o) - Male, 65<br>Patient C (Suppl Fig 3a,b, Reviewer Suppl Fig 3f-h) - Male, 64<br>Patient D (Figure 5, 6f-g and suppl figure 5) - Male, 71<br>Patient E (Figure 5, and suppl figure 5) - Male, 46
Recruitment	Patients presenting with Barrett's Esophagus were systematically approached for recruitment to the Oxford Gastrointestinal Illness Biobank at routine endoscopy appointments at the Endoscopy Department, John Radcliffe Hospital, Oxford.
Ethics oversight	Duodenum (D2) biopsies were collected from outpatients during endoscopy at the John Radcliffe Hospital, Oxford, with written informed consent (through the Oxford Gastrointestinal Illness Biobank, authorized by Yorkshire & The Humber - Sheffield Research Ethics Committee: 16/YH/0247; laboratory research using the samples authorized by South Central - Oxford C Research Ethics Committee: 09/H0606/78).

Note that full information on the approval of the study protocol must also be provided in the manuscript.

Field-specific reporting

Please select the one below that is the best fit for your research. If you are not sure, read the appropriate sections before making your selection.

[x] Life sciences [ ] Behavioural & social sciences [ ] Ecological, evolutionary & environmental sciences

For a reference copy of the document with all sections, see nature.com/documents/nr-reporting-summary-flat.pdf

Life sciences study design

All studies must disclose on these points even when the disclosure is negative.

Sample size	Synthetic organoid datasets were generated with similar sample size and number of frames as that of the experimentally collected timelapse confocal microscopy images of mutant colon organoids (Fig. 4). For organoid experiments, given a lack of expectations for effect size before the experiments were performed, no a priori power analyses were conducted to determine adequate n-values – instead, efforts were made to produce the highest n-values that could be reasonably achieved for each experiment.
Data exclusions	No data was excluded. Statistical outliers were detected and filtered out during analysis as part of our SPOT workflow.
Replication	We validated the replicability and general applicability of SPOT on simulated organoid datasets with defined dynamic phenotypes which we have deposited in Zenodo (DOI: 10.5281/zenodo.14265519). We also validated on computer vision and cell imaging datasets detailed in our companion methods-focussed paper, published in parallel with this paper (Zhou et al., "Development of a universal imaging "phenome" using Shape, Appearance and Motion (SAM) features and the SAM Observation Tool (SPOT)"). For organoid experiments we included at least 3 independent technical replicates (videos of a well) per condition.
Randomization	NA
Blinding	NA

Reporting for specific materials, systems and methods

We require information from authors about some types of materials, experimental systems and methods used in many studies. Here, indicate whether each material, system or method listed is relevant to your study. If you are not sure if a list item applies to your research, read the appropriate section before selecting a response.

Materials & experimental systems

n/a	Involved in the study	
x		Antibodies
	x	Eukaryotic cell lines
x		Palaeontology and archaeology
	x	Animals and other organisms
x		Clinical data
x		Dual use research of concern
x		Plants

Methods

n/a	Involved in the study	
x		ChIP-seq
x		Flow cytometry
x		MRI-based neuroimaging

Eukaryotic cell lines

Policy information about cell lines and Sex and Gender in Research

Cell line source(s)	HEK293T Rspo1-Fc cells (provided by the laboratory of Prof. Gijs van den Brink), HEK293T Nog-Fc cells (provided by the laboratory of Prof. Calvin Kuo, Stanford University) and L Wnt3A (CRL-2647™) .
Authentication	None of the cell lines were authenticated.
Mycoplasma contamination	All cell lines tested negative for mycoplasma contamination.
Commonly misidentified lines (See ICLAC register)	N/A

Animals and other research organisms

Policy information about studies involving animals; ARRIVE guidelines recommended for reporting animal research, and Sex and Gender in Research

Laboratory animals

Figure 4, 6a-e and suppl figure 4 Mus musculus C57BL/6J Wild Type - 6weeks
Figure 4, 6a-e and suppl figure 4 Mus musculus C57BL/6J Apcmin/+ - 14weeks and 4 days
Figure 4, 6a-e and suppl figure 4: Mus musculus C57BL/6J KrasLSL-G12D/+ Villin-CreERT2-EYFP - 43 weeks
Figure 4, 6a-e and suppl figure 4: Mus musculus C57BL/6J KrasLSL-G12D/+ Villin-CreERT2-EYFP:p53Null - 6 weeks and 2 days
Figure 4, 6a-e and suppl figure 4: Mus musculus C57BL/6J p53LSL-R172H: Hom - 6 weeks
Suppl figure 3c-s: Mus musculus BALB/cOlaHsd Wild Type 1 - 8 weeks and 3 days
Suppl figure 3c-s: Mus musculus BALB/cOlaHsd Wild Type 2 - 8 weeks and 3 days
Suppl figure 3c-s: Mus musculus BALB/cOlaHsd p53LSL-R172H: Hom 1 - 8 weeks and 3 days
Suppl figure 3c-s: Mus musculus BALB/cOlaHsd p53LSL-R172H: Hom 2 - 8 weeks and 3 days

Wild animals	This study did not involve wild animals.
Reporting on sex	Figure 4, 6a-e and suppl figure 4a: C57BL/6J Wild Type - Female<br>Figure 4, 6a-e and suppl figure 4: C57BL/6J Apcmin/+ - Male<br>Figure 4, 6a-e and suppl figure 4: C57BL/6J KrasLSL-G12D/+ Villin-CreERT2-EYFP - Male<br>Figure 4, 6a-e and suppl figure 4: C57BL/6J KrasLSL-G12D/+ Villin-CreERT2-EYFP:p53Null - Female<br>Figure 4, 6a-e and suppl figure 4: C57BL/6J p53LSL-R172H:Hom - Female<br>Suppl figure 3c-s: Mus musculus BALB/cOlaHsd Wild Type 1 - Male<br>Suppl figure 3c-s: Mus musculus BALB/cOlaHsd Wild Type 2 - Male<br>Suppl figure 3c-s: Mus musculus BALB/cOlaHsd p53LSL-R172H: Hom 1 - Male<br>Suppl figure 3c-s: Mus musculus BALB/cOlaHsd p53LSL-R172H: Hom 2 - Male
Field-collected samples	This study did not involve field-collected samples.
Ethics oversight	Animal work was approved by the Animal Welfare Ethical Review Body at the University of Oxford and licensed by the UK Home Office (PPLs 30/3451 and PP7833295).

Note that full information on the approval of the study protocol must also be provided in the manuscript.

Plants

Seed stocks	*Report on the source of all seed stocks or other plant material used. If applicable, state the seed stock centre and catalogue number. If plant specimens were collected from the field, describe the collection location, date and sampling procedures.*
Novel plant genotypes	*Describe the methods by which all novel plant genotypes were produced. This includes those generated by transgenic approaches, gene editing, chemical/radiation-based mutagenesis and hybridization. For transgenic lines, describe the transformation method, the number of independent lines analyzed and the generation upon which experiments were performed. For gene-edited lines, describe the editor used, the endogenous sequence targeted for editing, the targeting guide RNA sequence (if applicable) and how the editor was applied.*
Authentication	*Describe any authentication procedures for each seed stock used or novel genotype generated. Describe any experiments used to assess the effect of a mutation and, where applicable, how potential secondary effects (e.g. second site T-DNA insertions, mosiacism, off-target gene editing) were examined.*
