# Supplementary material for: Identifying phenotype-genotype-function coupling in 3D organoid imaging using Shape, Appearance and Motion Phenotype Observation Tool (SPOT)
Source: Nat Commun. 2026 Aug 17;17:8410. doi: 10.1038/s41467-026-75506-7 (PMC13482367; doi:10.1038/s41467-026-75506-7)
Supplement: Supplementary file 4 [file 41467_2026_75506_MOESM9_ESM.pdf]

nature portfolio

Peer Review File

Identifying phenotype-genotype-function coupling in 3D organoid imaging using Shape, Appearance and Motion Phenotype Observation Tool (SPOT)

Corresponding Author: Professor Xin Lu

This file contains all reviewer reports in order by version, followed by all author rebuttals in order by version.

Version 0:

Reviewer comments:

Reviewer #1

(Remarks to the Author)
This manuscript introduced a pipeline to track the phenotypic changes of objects in 2D videos, and applied it on living-cell videos. Together with single cell sequencing data, this tool may study the phenotype-genotype association. I have the following comments:

*It is hard to call any chosen summary features as comprehensive and unbiased phenotypic description of the video, as long as you do not use all the pixel information. For example, your feature does not cover the acceleration of segmented objects even if you only work on 2D objects from single color 2D images.

*line 169-170: how did you select the tuning parameters 200 and 3 here? Is it possible to automatically select by certain version of cross-validation?

*The success of the whole pipeline is dependent on accurate object tracking/tracklet. Can you quantify the accuracy of this step of your pipeline and discuss how this accuracy will affect the downstream results numerically?

*The 3D organoid imaging produces z-stack 3D images. Is it too rough to simply project the z-stack to one 2D image as in line 1476? You may want to as least discuss on how to better treatment 3D images.

*Your hidden Markov model description is confusing. According to line 1642-1643, both the observed data and the hidden states are phenotype cluster labels. You should differ them. Also, the entropy of the emission distribution may be related to the differentiability of your phenotype clusters, thus worthy of discussion.

*Is this work related to other shape and motion work/patent of the first author? If yes, you shall explain the connection.

*for the phenotype-genotype coupling, since it is currently impossible to get both the phenotype and genotype of the same cell at the single cell level, what potential problem does the population-level coupling have? How will poor coupling/mis-alignment affect the study?

(Remarks on code availability)
The author provided the code and README, but the code is too complicated for me to run.

Reviewer #2

(Remarks to the Author)
This scientific paper introduces a concept in the field of imaging analysis, termed the "imaging phenome," which is analogous to the transcriptome in single-cell transcriptomics. The authors propose a comprehensive framework to describe phenotypic diversity in dynamic objects through a standardized set of image descriptors. These descriptors are categorized

under three major properties: Shape, Appearance, and Motion (SAM), each of which is considered as an individual imaging "transcript."
To materialize this concept, the paper details the establishment of a standardized SAM "phenome," comprising 2185 distinct SAM features. This extensive collection of features provides a robust and comprehensive method to characterize various dynamic objects in imaging studies. Alongside this, the authors have developed a sophisticated analytical tool called SPOT (SAM Phenotype Observation Tool). SPOT is described as an imaging "sequencer," capable of analyzing both label-free and fluorescent images.
The paper underscores the potential of this approach in enhancing the understanding of phenotypic diversity through detailed and standardized imaging analysis, akin to the insights provided by transcriptomic analysis in genetics.

I've read the paper with great interest and the paper is well written and the strategy is well explained. However, I cannot advice for publication of the manuscript in its current form and the scientific work needs several improvements. Nonetheless, I think it is an interesting strategy, although applying scRNA analysis tools to single organoid image analysis is not novel (https://doi.org/10.1038/s41698-023-00480-y). But I think the approach has a lot of potential, if applied correctly.

• The manuscript is way too long and lacks focus. For me, these are at least two or three independent papers. (i) Development of the SAM tool and algorithms and testing it on the public databases. (ii) applying SAM to 2D cell culture (iii) applying SAM to 3D organoid culture. For readers, it is very difficult to extract the information that is relevant for them, and these three topics mostly have a different target audience.

• I wonder how relevant the use of the public databases from youtube and MPEG-7 is. How relevant are these features in cell biology and using this to compare with CellProfiler seems strange. Would a repository such as LIVECell not be more relevant to determine is SAM can accurately cluster distinct cellular morphologies rather than irrelevant images and videos (https://www.nature.com/articles/s41592-021-01249-6)?

• My main concern is the images that are used for the organoids. SAM quantifies 2185 features, so it is very sensitive to imaging artifacts. Unfortunately, the imaging and culturing strategy that was applied is not compatible with SAM.

o Organoids are grown in ECM domes, therefore they are not in the same plane which is a minimal requirement for this approach. There are various methods and plates available to image organoids in a single plane such as microwell plates (e.g. Gri3D) or flat-bottom 384-well ULA plates (https://www.jove.com/v/64434/multiparametric-tumor-organoid-drug-screening-using-widefield-live).
o From image 3B it is very evident that the clusters are mainly based on imaging artefacts. For example, cluster 3 are out of focus organoids, cluster 2 are overexposed organoids, cluster 7 are underexposed organoid, cluster 4 out of focus and underexposed… Although there might be some phenotypic differences, features extracted from these images are completely unreliable due to the imaging artefacts.
o The label-free organoid segmentation is not accurate enough, and tracking of the organoids is not consistent over time (e.g. video 4) or the density of the organoids is way too high (e.g. video 5). Organoids merge together, overlap, get detected – undetected,… so there is no consistency that allows for kinetic measurements crucial for SAM. Again, this can all be resolved by the use of proper imaging-compatible culturing techniques and plates.

• I'm wondering how much features are actually relevant. For example, for figure 3, studying the drug response in the organoids, the same conclusion can be drawn with just basic imaging features and do not require quantification of 2184 features. So for basic drug screening I think this is overkill. In addition, the authors make statements about that certain clusters correlate with for example high levels of cell death (line 575) but no evidence of cell death is provided (e.g. fluorescent cell death marker). So I understand unbiased clustering of features is interesting, but it would be more interesting if they can be linked to processes such as viability, cell death, apoptosis,… by using corresponding fluorescent markers. This way you can determine SAM features related to specific biological processes and give meaning to a cluster (similar to linking scRNA seq clusters to biological pathways based on genesets).

• The authors use the term 'flatted organoid morgphology' (line 765). It is difficult to see from the image, but are these cells that adhere to the plastic surface and start growing in 2D? This might be evidence of a more invasive, migratory behaviour.

While I find that the current version of the manuscript does not meet the necessary criteria for publication in its present form, I would like to emphasize the valuable insights and potential contributions your work offers. The breadth of the topics covered is commendable, yet a more focused approach could enhance the clarity and impact of your research. I strongly encourage you to consider refining the scope of your paper or split up the paper into two or three papers, sharpening the central arguments, and addressing the highlighted issues. A revised version that addresses these aspects would be a welcome submission to this journal. Your efforts in advancing research in this field are appreciated, and I look forward to potentially seeing a revised manuscript, in particular with a focus to organoids. I would like to express openness to potential collaboration. In my current research, we have developed an extensive library of organoid images treated with a variety of therapies. If you find it relevant, I believe a collaborative effort could enrich both our research endeavors, leveraging our respective resources and expertise.

Yours sincerely,
Prof. dr. Christophe Deben

(Remarks on code availability)

Reviewer #3

(Remarks to the Author)
The paper describes an extension to the toolbox of researchers that appears to outperform the established standard, namely CellProfiler. The authors argue that the introduction of motion in their pipeline, and a determination between global, local-regional and pattern-pixel levels are key differentiators for success. The authors support this statement well in text and figures, namely through the reporting of contribution scores for their clustering interpretation.

The validation work of the developed tool, SPOT, also revealed a potential new insight into the working of the WNT-pathway. The authors demonstrated that for two contradicting configurations, WNT containing mouse organoids and WNT deprived human organoids, the observed morphological behavior to have a similar underlying cause. The authors support this with single-cell-sequencing and investigated phototoxicity as a potential cause of these events. I believe this to be a new finding, with a clear and soundly executed methodology.

The currently proposed tool, SPOT, has been validated on multiple 2-dimensional datasets. The authors do show the potential of spot for organoid segmentation from Matrigel droplets, a 3-dimensional environment where organoids significantly overlap in the image. It is however currently unclear whether SPOT will work well for 3-dimensional time-series data. A possible complication can be that the motion module in SPOT can confuse X/Z movement for a phenotypic response of cells.

Overall, the paper is very well written, providing a new toolbox to researchers that has the potential to become of significant value to the field.

(Remarks on code availability)

Version 3:

Reviewer comments:

Reviewer #1

(Remarks to the Author)
My previous comments are mostly addressed to the extend that the authors could do, except for the HMM one.

Here is my previous comment: Your hidden Markov model description is confusing. According to line 1642-1643, both the observed data and the hidden states are phenotype cluster labels. You should differ them.

Here is authors' reply:
We apologise to the reviewer for the confusion. The Hidden Markov Model is used here to temporally smooth and compute the transition between phenotype cluster transition labels, as in Held et al We have already employed cluster number selection for identifying the phenotype clusters earlier in the workflow. We set the number of emission states to be equal to the number of hidden states.

So, indeed, both the observed data and the hidden states are phenotype cluster labels? What is the scientific point of smoothing the first-stage clustering result? If you think the cluster label shall be spatially clustered, you may want to build this spatial clustering mechanism into the first-stage clustering to increase statistical power.

(Remarks on code availability)
I did not run it successfully.

Reviewer #2

(Remarks to the Author)
I would like to thank the authors for their extensive work and for splitting the manuscript into two focused papers. This separation has certainly improved the clarity and readability of each individual contribution.
However, I must note that several of my core concerns from the initial review have not been fully addressed—particularly regarding the robustness and reproducibility of the SPOT framework, and the added value of this complex phenotypic analysis compared to more conventional, well-established approaches in the field of drug screening.

The authors present three central assumptions to support their handling of imaging artefacts:
1. Each cluster does not necessarily represent a distinct biological phenotype, and multiple clusters may correspond to the same underlying phenotype.
2. Only clusters with true biological dynamics are expected to show differential temporal evolution.
3. Artefacts are assumed to be temporally static and affect all experimental conditions equally.

While I understand the rationale and agree that temporal dynamics can help differentiate artefactual from biological changes, I find the assumptions overly optimistic. In real-world time-lapse imaging—especially with 3D organoids—artefacts can indeed change over time. Organoids may move in and out of focus, shift between lighting gradients, or become partially occluded, leading to non-biological temporal variation in the SAM features.

Although the inclusion of simulated datasets is a useful first step, they do not sufficiently capture the complexity and variability of real experimental data, particularly in organoid imaging where segmentation and intensity artefacts are common and often heterogeneous.

I think the influence of the artefacts depends on the level of information you want to get out the analysis. In this context, it was not clear to me what the author mean with 'SPOT analysis generates the same relative drug toxicity despite differences in measured SAM (Supp.Fig. 3b) in line 405, when comparing plastic and optical-bottom plates. Is this referring specifically to the classification into low/mild/high categories, or something else? Clarifying this explicitly would help avoid confusion.

The comparison between optical-bottom and standard plastic plates demonstrates substantial variability:
• The number of clusters differs (8 vs. 7).
• Cluster distributions and density "hotspots" vary significantly between plate types for the same drug (e.g., 100 µM cisplatin). Supp. Fig. 3b b (ii)
• The final timepoint cluster compositions differ noticeably, despite being the same treatment condition. Supp. Fig. 3b b (iii)

So while their 'relative drug toxicity' might classify the same (low and higer toxicity), and phenotype trajectory similarity is also clustered the same, these are very high level outputs which could also be achieved which much simpler approaches. It would be more usefull to have a direct comparison of the underlying SAM modules that contribute to specific phenotype clusters. Since differences in plates should be static and non-temperal, the SAM modules and features should be consistent between both plates if I'm following the logic of the authors correctly. It would also be interesting to see how the conditions cluster when the data of both plates (and importanly independent biological replicates) is pooled together. If the method is robust and replicatable, I would assume that treatment conditions would cluster together between the two plates (or replicates). So I would like to hear the authours opinion on this matter, and if they share the opininion that similar SAM modules/features should underly the different clusters identified in these two different plates since plate differences should be excluded according to their hypothesis. In addition, I would love to see how robust this approach is for biological replicates, but I understand that this might be difficult to achieve. Maybe the technical replicates could be analysed seperatly to detemine is the same SPOT outcome (clusters and underlying SAM modules and features) are extracted from the replicates or another drug screening dataset could be used.

In summary, I am convinced by the technical sophistication of the SAM and SPOT framework. However, I remain unconvinced about the robustness of the approach for images with a lot of artefacts as used in this manuscript, as the authors do not provide replicate datasets of the same experiment (unless I missed it). It would be critical to demonstrate whether the same number of phenotype clusters—and, importantly, the same underlying SAM modules and features—are consistently identified across independent replicates or between the different plates. This would substantiate the claim that imaging artefacts are effectively accounted for or excluded because they are static, which is not fully supported by the current comparison between plastic and optical-bottom plates. Since the authours make the comparison with scRNAseq, the clustering is a first step. Afterwards we would dig deaper into the features that make up this cluster so it is important that they are consistent.

Moreover, the added value of this complex high-dimensional approach compared to standard kinetic measurements of cytostatic and cytotoxic responses remains unclear. The classification into low, mild, and high toxicity could likely be achieved with simpler and more interpretable metrics. While the clustering of 5-FU and gefitinib presents an intriguing finding, its biological significance is difficult to interpret given their distinct mechanisms of action and lack of biological explanation.

Overall, the approach shows promise, but to justify its broader adoption, it would be important to more clearly demonstrate how it provides novel and reproducible biological insights beyond what can be obtained through established methods, particurly in the field of drug screening to clearly demonstrate its added value in distinguishing treatment specific phenotypic. I leave it up to the editor if more in-depth analysis is required (biological replicates, more therapies,...) to support the application of SAM-SPOT for drug screening or that the example you provided in the manuscript is sufficient.

Small remarks:
1) In Supplementary Figures 3a c(i), organoids treated with docetaxel appear to exhibit strong caspase signal, despite maintaining a morphologically healthy appearance (i.e., not dark, fragmented, or granulated). This seems contradictory, as high caspase activity is generally associated with apoptotic progression and eventual cell death.

Furthermore, according to Figure f, the number of Cytotox Green spots (used to mark cell death) for the docetaxel condition remains constant suggesting that this caspase activity is not translating into cytotoxicity.

Why do healthy-looking organoids exhibit such strong caspase activity and could there be technical artefacts in the caspase reporter signal? It would be helpful if the authors could clarify this observation.

2) Classification of Toxicity Levels in Figure 3c (iv)

The authors classify the tested drugs into “low,” “mild,” and “high” toxicity groups (Fig. 3c iv). However, it is not clearly described how these toxicity classifications were determined. To strengthen the biological relevance and interpretability of the findings, it is essential to validate these classifications against standard drug-response metrics, such as growth rate based drug response metrics (e.g., GR or NDR), which differentiate cytostatic effects (likely corresponding to “mild toxicity”) from cytotoxic effects (presumably “high toxicity”). Without such evidence, the presented classification appears arbitrary or subjective. Incorporating these standard benchmarks would significantly enhance the clarity and robustness of the authors' conclusions.

3) The term ‘flattening’ of the organoids is used. If I'm correct, these are cells that migrate towards the bottom of the Matrigel dome and attach to the bottom, indicating invasive and migratory behaviour? So it might be interesting to look at EMT related gene expression and the role of WNT3A in maintaining epithelial integrity in normal organoids.

(Remarks on code availability)

Reviewer #3

(Remarks to the Author)
Reply to author comments addressing main concerns:
The three primary concerns identified by the authors: presence of artifacts (1), impact of inaccurate organoid segmentation (2), and applicability for 3D time series data (3) are adequately addressed in the reply and thoroughly addressed in the manuscript through additional figures, additional supplementary data, and the establishment of simulated datasets.

(1)
The authors address this point in reply to reviewer 2s comments. They comment: “Each cluster is not necessarily a biological phenotype, and more than one cluster could relate to the same biological process e.g., two clusters relate to cell death. The primary biological analysis is in the phenotype evolution of organoids between conditions. SPOT assumes that over time, only phenotype clusters corresponding to true biological phenotype dynamics will exhibit differential temporal evolution”.

I agree that most artifacts encountered (debris, illumination induced) shouldn't exhibit any temporal dynamics, and that this is a strong safeguard of the method.

The authors further support the robustness of SPOT in Supplementary Fig 2c. I would note that special care should be taken with some of these imaging and segmentation based errors.

The artifact labelled (ii - Overexposure) is also a key biological event that occurs e.g. organoids becoming cystic (darkening), in later timepoints of organoid culture.

And (v – Overlap) shows consistent significant disagreement between the mutual information, and adjusted random index traces (Phenotypic clustering agreement).

(2)
Supplementary movie 2 (Movie S2 mouse intestinal organoids plus VC treatment), Timestamp 0:50-1:04 shows that despite the listed improvements by the author, special care should be taken when applying SPOT to non-simulated organoid data.

Performance varies significantly in the 4 treated conditions, with the WT + V/C condition showing the appearance of a “regular organoid” in the traced outline (teal in bottom right image), but the actual morphology being strikingly different from the other samples.

Supplementary movie 4 (Movie S4 - human organoids wnt3a addback), Timestamp 0:44-049 demonstrate that when organoids become cystic (Dark, clumping/aggregation, and highly irregular in shape) segmentation performance severely degrades.

In the HOM (Control), ENR + WNT3A (day 9) & ENR (no WNT3A) conditions, organoids become cystic, and the object detector fails to capture these for an extended number of frames.

The authors comment on this in their reply: “We also emphasise that the tracking is implemented only to identify genuine organoids (alive or dead) which we define as being tracked for a minimum number of frames (here, 5 frames)” which works to prevent faulty segmentation from being picked up, but does not allow the method to capture the unique morphology captured by these conditions. Improvements to the organoid detection routine would strengthen SPOT to better capture these unique morphologies.

However, this would serve to enrich the analysis – and the current methodology is adequately equipped to cover most tested and demonstrated conditions, erring on the side of caution.

(3)
The creation of an organoid simulator, simulated datasets A & B, and creation of additional supplementary figures are greatly appreciated. 8 scenes are provided across 2 different datasets (with replicates for the XYZ condition), with individually tracked organoids, their masks, and resulting projections.

Specifically on the original remark “It is however currently unclear whether SPOT will work well for 3-dimensional time-series data. A possible complication can be that the motion module in SPOT can confuse X/Z movement for a phenotypic response of cells” the authors demonstrate that XYZ movement shows a distinct SAM phenome for 2 replicates compared to the XY movement.

As the authors point out, the stacked barplots of cluster frequency do not show distinct differences, but that the XYZ movement is captured in the phenomic landscape (annotated with XYZ-only patch).

This supports the claim that SPOT, even when working with projected data, shows promise to be applicable in 3D time series data. As the authors themselves pointed out (Supplementary Figure 1b), “whilst the available space in the ECM dome constrains organoid movement to be largely planar in xy” this reviewer acknowledges the evidence and effort to make the methodology robust for non-ECM-dome configured organoid tracking as well.

I have enjoyed reading through the revised manuscript and applaud the authors for their significant improvements to the manuscript, and the other reviewers for their constructive and extensive feedback. Splitting up the manuscript helped bring focus to the text, allowing more evidence to be provided to support applicability of SPOT to real-world organoid data.

(Remarks on code availability)

Reviewer #4

(Remarks to the Author)
I am confused as the authors seem to have two versions of the same article. One named "Development of a universal imaging “phenome” using Shape, Appearance and Motion (SAM) features and the SAM Observation Tool (SPOT)" and one this one that I am writing review for now. I have already wrote reviews for the "developement ..." version and I will paste it here as well. My only new comment for this current version "Identifying ..." is that this version seems to have more support from biological and simulated data but lacks the comparison to the state-of-the-art. Why did not the authors submit one version with all three in place (biological verification, simulated experiments, literature comparison)? That would have been best in my opinion.

The below is the same comments as I had for the "Development ..." version of the paper which are relavent for the current version, i.e., "Identifying ..." too. I do not have the biological knowledge to verify any biological verification that the "Identifying ..." version claims, as I am a computer scientist.

1. What are the noteworthy results?

The authors claim that a universal set of 2185 image-derived features is developed to quantify an object’s phenotype (“standard” image-based “phenome”); subsequently, they provide a tool for image “sequencing” analysis of phenomes. For temporal analysis, they compute the HMM phenotype cluster transition graph that shows the different state of each detected phenome cluster over time.

2. How does it compare to the established literature?

I. In the introduction, the authors mention two previous feature descriptors, CellProfiler and TISmorph. But they only compare to Cellprofiler. The comparison is not enough. The authors should compare to TISmorph36 too.

II. The comparison is limited to two methods, i.e., merely done for CNN and CNN-LSTM based autoencoder. The authors should compare to more methods e.g. generative models and graph-based models. Two examples of such methods: A) Dynamic graph convolutional foldingnet in "3D single-cell shape analysis of cancer cells using geometric deep learning", B) "DeepSea is an efficient deep-learning model for single-cell segmentation and tracking in time-lapse microscopy". Then given their claim of rotation-invariance, they should compare to different VAE tackling rotation-invariance and not just a naive auto-encoder: C) “ME-VAE: Multi-Encoder Variational AutoEncoder for Controlling Multiple Transformational Features in Single Cell Image Analysis”

3. Does the work support the conclusions and claims, or is additional evidence needed?

I. In suppl. Fig. 8 the number of features differ between SAM and CNN-LSTM. What happens if the number of features set to be the same? That way it may be more fair to compare. The same should be done for the table of results comparing the accuracy of CNN and CNN-LSTM (right now SAM has not the same number of features as the other two).

II. In all illustrations of HMM cluster transition probability, it looks like a state machine rather than a probabilistic state machine (HMM. The author should annotate what each edge coloring represents as a probability.

III. See points 2 and 5 and address them too.

4. Are there any flaws in the data analysis, interpretation and conclusions? Do these prohibit publication or require revision?

I cannot see any.

5. Is the methodology sound? Does the work meet the expected standards in your field?

I. I do not understand their HMM. I thought first Kmeans is used to find 7-8 clusters and then for "each cluster" the temporal analysis is done (this is what the authors claim in line 1319 section). This makes sense to me. However, in their HMM transition (shape of state machine with color-coded arrows), I see that they show transition between those 7-8 clusters. Why this? and why not the state transition for each cluster separately? If the author answer this as "we want to see transition between different clusters" then my judgment is that their modeling is not sensible - they should NOT first do Kmeans to detect states of an HMM (the whole point of HMM is to find those states). Instead, they should have directly applied HMM and then Viterbi to find the most probable sequence, which would unfold the trajectory of states - the states would be detected by fitting the HMM and would not need to have K-means in advance.

II. The authors should provide answer to this: why SAM? I am not sure if motion is sufficiently captured since it is "Motion as combined changes in the object's shape, appearance, and centroid between consecutive timepoints". That is, why not separately consider rotation and other potential spacial variations which can change the perception. See, for example, "ME-VAE: Multi-Encoder Variational AutoEncoder for Controlling Multiple Transformational Features in Single Cell Image Analysis". Finally, a way to convince reviewers is to show an example of "rotation" and an example of "polar orientation" that is correctly detected using their method, and it is not confused and perceived as a change in shape.

III. The authors state that "Motion as combined changes in the object's shape, appearance, and centroid between consecutive timepoints". Mathematically, thinking SAM should capture mutually exclusive features of change, but due to their definition of motion, it implies that motion does not capture a distinct feature. How do the authors support the sufficiency of their definition of the motion?

IV. How is it determined that rotation should always be ignored? Maybe a rotation is part of a meaningful motion? I am not sure about this but I put this as a question to be answered.

V. The preprocessing contains removal of highly variant features "All dataset dependent preprocessing steps such as zero-valued and high variance features". I do not see why this is sensible. Conversely, I can see that high variance features are useful signals.

6. Is there enough detail provided in the methods for the work to be reproduced?

Yes.

(Remarks on code availability)
I am confused as the authors seem to have two versions of the same article. One named "Development of a universal imaging "phenome" using Shape, Appearance and Motion (SAM) features and the SAM Observation Tool (SPOT)" and one this one that I am writing review for now. I have already wrote reviews for the "developement ..." version and I will paste it here as well. My only new comment for this current version "Identifying ..." is that this version seems to have more support from biological and simulated data but lacks the comparison to the state-of-the-art. Why did not the authors submit one version with all three in place (biological verification, simulated experiments, literature comparison)? That would have been best in my opinion.

The below is the same comments as I had for the "Development ..." version of the paper which are relavent for the current version, i.e., "Identifying ..." too. I do not have the biological knowledge to verify any biological verification that the "Identifying ..." version claims, as I am a computer scientist.

1. What are the noteworthy results?

The authors claim that a universal set of 2185 image-derived features is developed to quantify an object's phenotype ("standard" image-based "phenome"); subsequently, they provide a tool for image "sequencing" analysis of phenomes. For temporal analysis, they compute the HMM phenotype cluster transition graph that shows the different state of each detected phenome cluster over time.

2. How does it compare to the established literature?

I. In the introduction, the authors mention two previous feature descriptors, CellProfiler and TISmorph. But they only compare to Cellprofiler. The comparison is not enough. The authors should compare to TISmorph36 too.

II. The comparison is limited to two methods, i.e., merely done for CNN and CNN-LSTM based autoencoder. The authors should compare to more methods e.g. generative models and graph-based models. Two examples of such methods: A) Dynamic graph convolutional foldingnet in "3D single-cell shape analysis of cancer cells using geometric deep learning", B)

"DeepSea is an efficient deep-learning model for single-cell segmentation and tracking in time-lapse microscopy". Then given their claim of rotation-invariance, they should compare to different VAE tackling rotation-invariance and not just a naive auto-encoder: C) "ME-VAE: Multi-Encoder Variational AutoEncoder for Controlling Multiple Transformational Features in Single Cell Image Analysis"

3. Does the work support the conclusions and claims, or is additional evidence needed?

I. In suppl. Fig. 8 the number of features differ between SAM and CNN-LSTM. What happens if the number of features set to be the same? That way it may be more fair to compare. The same should be done for the table of results comparing the accuracy of CNN and CNN-LSTM (right now SAM has not the same number of features as the other two).

II. In all illustrations of HMM cluster transition probability, it looks like a state machine rather than a probabilistic state machine (HMM. The author should annotate what each edge coloring represents as a probability.

III. See points 2 and 5 and address them too.

4. Are there any flaws in the data analysis, interpretation and conclusions? Do these prohibit publication or require revision?

I cannot see any.

5. Is the methodology sound? Does the work meet the expected standards in your field?

I. I do not understand their HMM. I thought first Kmeans is used to find 7-8 clusters and then for "each cluster" the temporal analysis is done (this is what the authors claim in line 1319 section). This makes sense to me. However, in their HMM transition (shape of state machine with color-coded arrows), I see that they show transition between those 7-8 clusters. Why this? and why not the state transition for each cluster separately? If the author answer this as "we want to see transition between different clusters" then my judgment is that their modeling is not sensible - they should NOT first do Kmeans to detect states of an HMM (the whole point of HMM is to find those states). Instead, they should have directly applied HMM and then Viterbi to find the most probable sequence, which would unfold the trajectory of states - the states would be detected by fitting the HMM and would not need to have K-means in advance.

II. The authors should provide answer to this: why SAM? I am not sure if motion is sufficiently captured since it is "Motion as combined changes in the object's shape, appearance, and centroid between consecutive timepoints". That is, why not separately consider rotation and other potential spacial variations which can change the perception. See, for example, "ME-VAE: Multi-Encoder Variational AutoEncoder for Controlling Multiple Transformational Features in Single Cell Image Analysis". Finally, a way to convince reviewers is to show an example of "rotation" and an example of "polar orientation" that is correctly detected using their method, and it is not confused and perceived as a change in shape.

III. The authors state that "Motion as combined changes in the object's shape, appearance, and centroid between consecutive timepoints". Mathematically, thinking SAM should capture mutually exclusive features of change, but due to their definition of motion, it implies that motion does not capture a distinct feature. How do the authors support the sufficiency of their definition of the motion?

IV. How is it determined that rotation should always be ignored? Maybe a rotation is part of a meaningful motion? I am not sure about this but I put this as a question to be answered.

V. The preprocessing contains removal of highly variant features "All dataset dependent preprocessing steps such as zero-valued and high variance features". I do not see why this is sensible. Conversely, I can see that high variance features are useful signals.

6. Is there enough detail provided in the methods for the work to be reproduced?

Yes.

Version 4:

Reviewer comments:

Reviewer #4

(Remarks to the Author)
The authors have addressed all the points, and I thank them for all the effort.

(Remarks on code availability)

Open Access This Peer Review File is licensed under a Creative Commons Attribution 4.0 International License, which permits use, sharing, adaptation, distribution and reproduction in any medium or format, as long as you give appropriate credit to the original author(s) and the source, provide a link to the Creative Commons license, and indicate if changes were made.
In cases where reviewers are anonymous, credit should be given to 'Anonymous Referee' and the source.
The images or other third party material in this Peer Review File are included in the article's Creative Commons license, unless indicated otherwise in a credit line to the material. If material is not included in the article's Creative Commons license and your intended use is not permitted by statutory regulation or exceeds the permitted use, you will need to obtain permission directly from the copyright holder.
To view a copy of this license, visit https://creativecommons.org/licenses/by/4.0/

Reply to Reviewers' main concerns:

The Reviewers raised three main concerns:

1) SPOT's ability to deal with the presence of artefacts in the analysis;

2) the impact of potential inaccuracies in object segmentation and tracking; and

3) SPOT's suitability for 3D time-series imaging data.

To address all three of these concerns systematically and objectively, we built an organoid simulator to synthesize simulated *de novo* video datasets with known ground truth dynamic phenotypes. Each organoid can be prescribed with defined and dynamic organoid phenotypes, to simulate changes in only one or more of shape, appearance, or motion characteristics. Below, we summarise our response to the Reviewers' three primary concerns:

1. ***Potential sensitivity to artefacts:***

Reviewer 2: ***My main concern is the images that are used for the organoids. SAM quantifies 2185 features, so it is very sensitive to imaging artifacts.***
From image 3B it is very evident that the clusters are mainly based on imaging artefacts. For example, cluster 3 are out of focus organoids, cluster 2 are overexposed organoids, cluster 7 are underexposed organoid, cluster 4 out of focus and underexposed… Although there might be some phenotypic differences, features extracted from these images are completely unreliable due to the imaging artefacts.

Reply:
We apologise for not making it clear that including and reconciling artefacts within SPOT's analysis is a unique designed feature. The phenotype clustering is designed to identify different 'modes' or SAM imaging 'states' in the dataset by discretely partitioning the UMAP phenomic landscape. Each cluster is not necessarily a biological phenotype, and more than one cluster could relate to the same biological process e.g., two clusters relate to cell death. The primary biological analysis is in the phenotype evolution of organoids between conditions. SPOT assumes that over time, only phenotype clusters corresponding to true biological phenotype dynamics will exhibit differential temporal evolution, and this gives a signature to cluster conditions. Not all analysis uses this clustering. SAM phenotype trajectories are derived by the majority UMAP density in each temporal bin. In all cases, artefacts, when present, are assumed to not exhibit temporal evolution and to affect all conditions equally. We discuss these considerations explicitly and demonstrate the interpretation of phenotype cluster on the YouTube dataset of everyday objects in the methodological NC-SPOT-MS2. We further demonstrate the interpretation of phenotype cluster in NC-SPOT-MS1 using the simulated organoid dataset and new chemotherapy drug screens with the addition of a fluorescent caspase marker.

Imaging artefacts are almost always present in some manner during timelapse acquisition. Importantly, these artefacts differ between imaging modality and imaged subject. As a tool designed for high-content analysis, we believe that it would be a limitation to have to firstly

carefully remove artefacts, which requires domain expertise and potentially extensive manual proofreading. Naturally, if the user has trained an appropriate artefact detector for their data, this can be applied to further improve analysis and enhance biological interpretation. Nevertheless, we demonstrate in the new Suppl. Fig. 2 (NC-SPOT-MS1), by analysing simulated organoids subject to defined artefact type perturbations, that SPOT is either robust to an artefact type (such as blurring and intensity changes due to photobleaching), or, it makes the artefacts easily identifiable in the 2D UMAP to allow data subsetting. For example, when we perturb the ground truth simulated dataset with 'union' segmentation errors of varying severities (illustrated in Suppl. Fig. 2b a), the UMAP cleanly partitions the ground truth from the perturbed conditions. We can thus subset for and reperform analysis on only the unaffected images (Suppl. Fig. 2b c).

2. *Potential inaccuracies in object segmentation and tracking*

Reviewer 1: The success of the whole pipeline is dependent on accurate object tracking/tracklet. Can you quantify the accuracy of this step of your pipeline and discuss how this accuracy will affect the downstream results numerically?

Reviewer 2: The label-free organoid segmentation is not accurate enough, and tracking of the organoids is not consistent over time (e.g. video 4) or the density of the organoids is way too high (e.g. video 5). Organoids merge together, overlap, get detected – undetected,… so there is no consistency that allows for kinetic measurements crucial for SAM. Again, this can all be resolved by the use of proper imaging-compatible culturing techniques and plates.

Reply:

SPOT inherently includes safeguards that may affect analysis. With organoid detection, SPOT enforces a minimum temporal consistency in segmentation: all instances of a tracked organoid must have a frame-to-frame shape overlap (as measured by intersection-over-union (IoU)) greater than a user specified threshold. If this cut-off is too high, matching may be too stringent to shape changes between frames. We use a cut-off of IoU=0.25. We also emphasise that the tracking is implemented only to identify genuine organoids (alive or dead) which we define as being tracked for a minimum number of frames (here, 5 frames) from potential short-lived transient artefacts e.g., floating debris. In SPOT analysis, the tracked organoids at different timepoints are mapped as independent individual points in the UMAP. Only the motion features require tracking to compute. Even so, it depends only on a consecutive pair of accurate segmentations. To ensure this, SPOT implements postprocessing after tracking as a safeguard, such as temporal smoothing of segmentations (see Methods in our submitted NC-SPOT-MS2). In summary, SPOT is expected to be robust even if not all object instances are perfectly segmented (due to overlap for example), and robust in the case where some object instances are undetected or filtered out (such as by the IOU tracker, causing fragmented tracks). We validate our claims using our simulated 3D organoids in NC-SPOT-MS1 (NCOMMS-23-61673-T), by introducing varying degrees of segmentation errors 'cutout' and 'union'), that could result from overlap. If such instances occur and are highly prevalent, we find they cluster distinctly and can be removed (new Supplementary Figure 2b c). In addition, we simulated the effect of various degrees of track fragmentation (such as that caused by organoid instances remaining undetected or being filtered out), showing that the conclusions drawn from our analysis approach remain unaffected (new Supplementary Figure 2b d). We

have also added additional text in NC-SPOT-MS2 to explain the careful design considerations of the SAM phenome to also be robust to these upstream processing errors.

3. *Potential issues with SPOT's use for 3D time-series data*

Reviewer 1: The 3D organoid imaging produces z-stack 3D images. Is it too rough to simply project the z-stack to one 2D image as in line 1476? You may want to as least discuss on how to better treatment 3D images.

Reviewer 3: Whether SPOT will work well for 3-dimensional time-series data, a possible complication can be that the motion module in SPOT can confuse X/Z movement for a phenotypic response of cells.

Reply:
We agree with the Reviewers that our study presented here is mainly based on projected 2D images from time-series data, and we recognize the limitation of the current version of SPOT to capture the full richness of 3D biology. The organoids we culture in our studies over 2 weeks grow large such that true 3D imaging acquisition of entire organoids at near-isotropic resolution would require sophisticated and customized light-sheet imaging setups, involving multiple objectives to try to overcome depth-based light scattering. These costly setups are not able to capture all organoids in the field-of-view in a high content nor high through-put manner, nor are they widely accessible. Therefore, we feel that only for particular biological hypotheses would an imaging tool that can accurately analyse true 3D time-series data at the cell/organoid scale be needed currently. Moreover, capturing full 3D significantly enhances phototoxicity and limits total acquisition time.

Organoids are grown in 3D and have emerged as a valuable experimental tool for high-throughput genetic and compound screens because they can recapitulate the cell lineages of their origin organ. A major application is identifying earlier markers of disease transformation. However, organoids take time to develop as the cells require time to differentiate. Consequently, it is much more valuable in live-imaging organoid experiments to take steps to minimize phototoxicity and maximize acquisition length. The standard practice in both pharmaceutical industry and research communities is for label-free microscopy such as bright-field and phase contrast microscopy modalities with small z-stacks. We follow this practice in this work, by imaging a few z-slices for up to 2 weeks and maintaining a relatively high acquisition rate of one frame per hour. In the study presented in NC-SPOT-MS1 (NCOMMS-23-61673-T), this imaging acquisition enabled us to reveal developmental differences due to differences in WNT signalling, in addition to the fact that these differences required several days to manifest, beyond the capability of commercial fluorescent lightsheet systems (Fig. 5). Moreover, as we highlight in Suppl. Fig. 1a,b our data whether label-free or confocal have insufficient z-resolution and offers no further insight beyond 2D analysis, even if we were to extend SAM-SPOT to perform 3D analysis.

To further address the Reviewers' concerns on the applicability of analysing 2D projection videos of z-stacks, we generated synthetic organoid datasets (see below). Simulated dataset B was specifically designed to assess the potential added value of capturing full 3D dynamics. Our results shown in the new Figure 2 illustrates that SPOT captures sufficient information in

2D projection videos to objectively discriminate between a condition where full 3D xyz movement was permitted from a second condition where movement was constrained to the 2D xy-plane.

4. *Creation of organoid simulator and synthetic datasets to validate the applicability and potential of SPOT*

We created an organoid simulator to objectively address all Reviewers' concerns on the sensitivity of SPOT to dataset and processing imperfections and on the extent to which 2D projection videos distinguish 3D phenotypes. Specifically, we generated two synthetic organoid video datasets (Simulated Datasets A and B) where we engineered the 3D phenotypes, then constructed and analysed the associated 2D projection videos with SAM-SPOT. These new datasets with known ground truth enabled us to address SPOT's ability to recognise and reconcile:

1) Noise and artefacts in the analysis;
2) Track fragmentation;
3) Inaccuracies in object segmentation caused by spatially overlapping organoids; and
4) SPOT's suitability for 3D time-series imaging data.

Points #1–3 are addressed by 'Simulated Dataset A', while the consequence of movement in the Z-dimension, Point #4, is addressed using 'Simulated Dataset B'. The synthetic organoids are simulated fully in 3D and then projected into 2D using the same extended focus projection principles as that used for real acquisitions. Using simulations enabled us to have ground-truth phenotypes and to apply a wide range of systematic perturbations (including those identified by the Reviewers) to test how effectively SPOT can extract 3D information from 2D projections, to assess how effectively SPOT can distinguish distinct phenotype categories with a known ground-truth, and to determine how robust SPOT analysis remains in the face of experimental, imaging, and segmentation imperfections, thereby enabling tolerance thresholds to be set.

Simulated Dataset A

We generated a synthetic dataset of simulated 3D organoids, that evolve across time from a small, round initial state into a phenotypically distinct final state (7 conditions, 45 organoids per condition, and 30 frames per organoid) (new Fig. 2b). Each of the conditions share a common starting state with identical shape, appearance, motion behaviour parameterisation, before diverging over the duration of the 30-frame time course into specially-parameterised, condition-specific end states. To generate this dataset, we define one condition-dependent end state as the 'base organoid', with 'basal' parameter values. Then, using the base organoid parameterisation as the template, we modified parameters influencing each of shape, appearance, and motion in isolation, to create 6 further 'high' or 'low' conditions for each. The dataset is comprised of 3-dimensional organoid simulations and their 2-dimensional projections along with pixel-perfect segmentation masks. To generate the 2-dimensional projections, we used an equivalent method to the extended depth of focus projection used on our real timelapse z-stacks. We subjected the entirety of Simulated Dataset A to 8 different

perturbation types, each across 9 severity levels, to identify effects on the analysis relative to the known ground truth.

Simulated Dataset B

We generated another synthetic dataset of simulated 3D organoids, with 3 conditions that share the same shape and appearance parameters, with organoids moving with the same speed but differing in the allowed movement directionality. One condition represents unrestricted xyz movement freedom, a second condition represents a replicate of the first condition with different organoids and the third condition represents movement only in the plane parallel to the projection i.e., xy plane (in total, 3 conditions, 6 replicates per condition, 12 organoids per replicate, 30 timepoints per organoid). This dataset was designed to address the specific concerns of Reviewer 3 regarding the effect of movement in the Z-dimension on phenotype readout.

The key findings of applying SAM-SPOT to the synthetic dataset A is included as new Figure 2 in the main text. The results of the synthetic robustness tests using perturbations of dataset A, and analysis of dataset B are included as additional Suppl. Fig. 2a and 2b.

EXTENDED POINT-BY-POINT RESPONSES TO EACH REVIEWER'S COMMENTS:

Reviewer #1 (Remarks to the Author):

This manuscript introduced a pipeline to track the phenotypic changes of objects in 2D videos, and applied it on living-cell videos. Together with single cell sequencing data, this tool may study the phenotype-genotype association. I have the following comments:

1) It is hard to call any chosen summary features as comprehensive and unbiased phenotypic description of the video, as long as you do not use all the pixel information.

We apologise for not defining the terms "comprehensive" and "unbiased" clearly in the text. We use the terms "comprehensive" and "unbiased" to refer to the way in which the image features are acquired and how the acquired data are analysed, respectively. Specifically, SAM features are "comprehensive" because they have been designed to be a generally applicable set of image features covering all spatial scales of global, regional, and local (i.e. distributional) (akin to late and early layers of a neural network) and the different attributes of shape, appearance and motion. We note local distribution features are derived by using all the pixel information. The specific composition of features is outlined in Methods and corresponding illustrations are also presented in Fig. 2 of the new NC-SPOT-MS2. We use the term *unbiased* to emphasise that all features in the SAM phenome can be and are computed for all datasets in the same manner and are not 'handcrafted' to target a particular phenotype *a priori* (e.g. organoid branching-specific) nor require adaptation towards any imaging modality or imaged object (e.g. number of branches is a feature specific only to objects that branch). This is in contrast to data-driven learning such as convolution neural network (CNN) features trained on a particular dataset, which is a "*biased"* approach in that it necessarily requires fine-tuning on the target dataset to guarantee relevance of generated features. In our new MS2 entitled "Shape, Appearance, and Motion (SAM) features enable construction of the "phenome" and development of SAM Observation Tool (SPOT)", we compared side-by-side the ability of SAM phenome against CNN-features or CNN-LSTM-features to analyse cell morphodynamics and found that, unexpectedly, SAM-phenome outperforms both CNN-features and CNN-LSTM-features in classification and interpretation (Figure 6, NC-SPOT-MS2). In both manuscripts, NC-SPOT-MS1 (NCOMMS-23-61673-T) and NC-SPOT-MS2, we have now explicitly clarified our usage of the terms "comprehensive" and "unbiased" when needed.

Finally, we agree with this reviewer that a higher number of features may be more comprehensive, but too many features make the phenome 'too unique' leading to poorer performance without further sophisticated preprocessing. In our new NC-SPOT-MS2 entitled "Development of a universal imaging "phenome" using Shape, Appearance and Motion (SAM) features and the SAM Observation Tool (SPOT)", we have now provided expanded and detailed description and explanation as to why 2185 SAM features is sufficiently comprehensive but minimally complex to constitute a SAM phenome (Figure 1, Supp Figure 1, NC-SPOT-MS2) as we also demonstrate with extensive empirical experiments. We further added specific discussion of this point in the Discussion of NC-SPOT-MS2.

2) For example, your feature does not cover the acceleration of segmented objects even if you only work on 2D objects from single color 2D images

Reply:

As acceleration is the time derivative of velocity, we would expect a change in acceleration to manifest in the next time frame as a change in velocity. Therefore, SPOT does indeed implicitly capture the acceleration of segmented objects. Generally, we expect higher-order motion statistics to manifest as increasing temporal delays, requiring longer tracking to capture. One frame in a video can represent any temporal unit from 1 ms to 1h to 1 day, depending on the video in question. Equally, the total number of frames can vary. Therefore, to ensure maximum applicability, we designed SPOT to only include motion measures that can be computed from 1 successive frame. We clarify this in NC-SPOT-MS2. This is why we consider it the instantaneous 'SAM phenome' and use this phenome to map all temporal object instances as individual points in a 2D landscape using UMAP. We note the long-term dynamics are not lost and are modelled back through the single object phenotype trajectories and phenotype cluster transitions, as we demonstrate and highlight in our analysis of single cell tracking (Fig. 6, Suppl. Fig. 6, NC-SPOT-MS2) and of cell division (Fig. 7, NC-SPOT-MS2).

3) line 169-170: how did you select the tuning parameters 200 and 3 here? Is it possible to automatically select by certain version of cross-validation?

Reply:

We want to store the segmented organoids in a more efficient manner than storing each binary pixel in individual cropped segmentation masks. However, individual boundaries have different numbers of points and would not allow the stacking of multiple boundaries into a regular array, resulting in larger and irregular storage on disk. We therefore chose to sample the contour of each organoid with 200 equidistant points, as comparable numbers have been used in the literature to represent complex cell morphologies e.g. CAJAL (Govek et al., *Nat. Commun.,* 2021) which used 100 points to classify diverse neuron morphologies. To demonstrate that 200 points does still retain good shape approximation empirically, we assessed the symmetric Hausdorff distance of MPEG-7 shapes with increasing number of sampled equidistant contour points. We find rapidly diminishing returns in capturing the shape complexity above 100 equidistance points. This analysis is included as new Suppl. Fig. 1 in our new NC-SPOT-MS2.

We partitioned the object area into 3 regions of equal distance from its boundary, as this has natural interpretation of the border, middle and central regions of an object. Unfortunately, in order for the number of total features to be identical across for all objects, the number of partitions also needs to be applicable and computable for all objects, irrespective of their area. As such, there is little scope for optimization. Whilst further subdivision is possible, for small objects, these regions would result in smaller (and possibly zero) image area, yielding more intrinsically noisy or uninformative features. Thus we decided to maintain three regions. We also did not consider further division by angular rotation which for concave shapes or shapes of high curvature may under- or over-sample the partitioned area in particular angles. Furthermore, we would require accurate rotational alignment to ensure the derived measurements are rotation-invariant, a key design rationale as we explain in NC-SPOT-MS2. Our choice is consistent with other works such as CellProfiler and TISMorph. We have

included clarification of the above design choices in new Figure 1c of NC-SPOT-MS2, which describes the development of our SAM features.

4) The success of the whole pipeline is dependent on accurate object tracking/tracklet. Can you quantify the accuracy of this step of your pipeline and discuss how this accuracy will affect the downstream results numerically?

a) Accurate object tracking and quantify the accuracy of the tracking pipeline:

Reply:

The tracking approach we implement is 'first detect then link'. Organoids are first detected as a bounding box on a per frame basis, then matched by solving the linear assignment problem using intersection-over-union (IoU) as the cost function. Using IoU intrinsically accounts simultaneously for size and closest distance. This linking approach is the most popular, state-of-the-art and optimally solves bipartite matching problems such as the pairing of detected organoids across two frames, e.g. also used by Fiji TrackMate software. We further extended this strategy by computing the rigid transform of the organoid between the current and next frame from the independently computed pixel optical flow. This predictive step enables us to further handle large morphological changes, and can be seen as the image-based extension of a Kalman Filter. We observed the matching between two frames to be 100% accurate. Our tracking approach is primarily used to enable us to determine whether the per frame based organoids detected are 'real' and to filter out transient objects such as floating debris. We assume that real organoids are detectable for at least 5 frames. Therefore our "detect and link" approach provides the required object tracking accuracy and specificity. Given good organoid detection in individual frames, we will have accurate pairwise matching. Then, only good individual frame organoid segmentation is needed for SAM phenome computation. To ensure sufficient detection and segmentation we trained neural networks (Methods, NC-SPOT-MS1 (NCOMMS-23-61673-T)):

We trained a YOLOv3 object detector for bounding box organoid detection with a total of 43,199 organoids extracted from 1534 images comprising label-free and fluorescent confocal microscopy. Images contained separated and cluttered overlapping organoids ranging from a few organoids per image to roughly 100. We measure a mean average precision with an intersection-over-union (IoU) cutoff of 0.25, $mAP_{25} = 0.91$ *on a test dataset of 43,145 organoids extracted from 1437 images comprising label-free and fluorescent confocal microscopy. Images contained separated and cluttered overlapping organoids ranging from a few organoids per image to roughly 100. If IoU cutoff is set to 0.50, we measure* $mAP_{25} = 0.67$.

We trained a simple attention-UNet model of 4 layers to segment individual organoid from image crops containing one organoid in isolation and spatially overlapping neighbors. The model has 8 features in layer 1 with the number of features doubling in depth as the image size is average pooled by a factor of 2. The model is trained on 542 unique cropped organoids from fluorescent confocal and label-free images resized to 64 x 64 pixels and random image augmentation. We obtain a test accuracy measured by IoU of 0.87 for a total 555 test images.

For SPOT analysis, longer-time tracking may benefit HMM model fitting, but is not critical to the Hidden Markov Model (HMM) based cluster transition analysis, as we demonstrate with our simulated organoid dataset (Suppl. Fig. 2c iv, bottom right, NC-SPOT-MS1). The mean

elementwise difference of the HMM transition matrices remain low relative to the ground truth (100% of frames) as the level of track fragmentation increases (coverage fixed at 50% of frames), showing only a slight positive trend. This indicates that whether the tracks are highly fragmented (short) or not (long), disagreement with the ground truth HMM cluster transitions is consistently low. All other analyses are minimally affected (Suppl. Fig. 2c ii,iii, bottom).

b) how this accuracy will affect the downstream results numerically?

Reply:
A unique feature and advantage of SPOT is its ability to analyse downstream data that are not 100% accurately tracked. Firstly, 420 of the motion features are derived statistics from the per pixel velocity vectors computed by two-frame optical flow. Optical flow uses the video pixel intensity values directly and therefore unaffected by organoid tracking. The optical flow technique used, Farneback, has been previously demonstrated in the first author's PhD thesis to work robustly for organoids, even being utilized to segment organoids and track organoid branching (Zhou, F. (2017). Phenotyping cellular motion [PhD thesis]. University of Oxford). Likewise, the Hidden Markov Model (HMM) model depends only on consecutive frames and estimates model parameters from many organoid instances. Secondly, the pairing of organoids between successive frames is one-to-one. Only one organoid can be paired to another. Even if there is an identity switch when tracking an organoid, the switch only affects that one timepoint of tracking. SPOT additionally implements an IoU consistency check between consecutive times of a tracked organoid whereby a track is split, if the bounding box of the organoid in the next frame differs too much in overlap, to prevent obvious switching errors. In particular, SPOT will split the track into two: one before the switch and one after the switch. For example, suppose we have a video of 20 frames where cultures are very dense, and during timepoint 0–10, we track organoid 1. Then, in frame 11, there is overlap with organoid 2 such that the track of organoid 1 switches to tracking 2, and the track of organoid 2 now tracks 1. Considering the whole track, the tracking of organoid 1 and 2 is 50% accurate, however the matching of organoids from frame-to-frame is high - only the preceding frame when identities switch is incorrect. Therefore inaccurate tracking has minimal effect on SPOT. As each instance of an organoid is an independent observation in the SPOT analysis, there is only one erroneously matched timepoint for organoid 1 and 2 at timepoint 10. All other timepoints are correctly matched. Importantly, during the revision, the accuracy, effectiveness and robustness of SPOT was objectively validated using our newly created 3D organoid simulation movies, with systematic perturbations to segmentation and tracking (new Figure 2 and related Supplementary Figures). Details are now included in the methods section of NC-SPOT-MS1 (NCOMMS-23-61673-T).

5) The 3D organoid imaging produces z-stack 3D images. Is it too rough to simply project the z-stack to one 2D image as in line 1476? You may want to as least discuss on how to better treatment 3D images.

Reply:

We thank the reviewer for this comment and acknowledge that our current approach to project z-stack to one 2D image would be suboptimal, had we captured isotropic resolution images of organoids grown in 3D so that images contain unique 3D SAM information. However, as seen in Suppl. Fig. 1a and 1b, both our label-free and confocal microscopy z-

stacks are resolution-limited. The 3D stack offers effectively only 2D information. Obtaining more z-slices with confocal microscopy might slightly improve 3D resolution but would severely affect photobleaching, drastically limiting acquisition frequency and total duration as well as potentially affecting native biological behaviour. Lightsheet microscope setups may achieve near-isotropic 3D imaging, but is currently insufficiently high-throughput and high-content for our organoid experiments. It also suffers the same issues of photobleaching. Moreover, due to scattering, it cannot capture the full organoid depth without the need to design custom, and complex multi-camera and sample holder setups, beyond the capability of our lab and the scope of this manuscript.

Instead, to assess the extent of mutual information between full 3D images and the equivalent 2D projections used by SPOT, we simulated full 3D organoid movies with defined phenotypes and analysed the 2D extended focus projection organoid scenes with SPOT. Our findings in the new Suppl. Figure 2 of NC-SPOT-MS1 (NCOMMS-23-61673-T) showed from 2D projection videos only, SAM-SPOT can still effectively discriminate simulated organoids whose movement are restricted to the 2D xy-plane from those with unrestricted 3D xyz movement, even with a replicate dataset of unrestricted 3D xyz movement as confounder.

6) Your hidden Markov model description is confusing. According to line 1642-1643, both the observed data and the hidden states are phenotype cluster labels. You should differ them.

Reply:
We apologise to the reviewer for the confusion. The Hidden Markov Model is used here to temporally smooth and compute the transition between phenotype cluster transition labels, as in Held et al. We have already employed cluster number selection for identifying the phenotype clusters earlier in the workflow. We set the number of emission states to be equal to the number of hidden states. We have included this consideration in the Methods of the new methods-focussed manuscript NC-SPOT-MS2.

7) Also, the entropy of the emission distribution may be related to the differentiability of your phenotype clusters, thus worthy of discussion.

We thank the reviewer for this suggestion. We have computed the entropy of the transition matrix for each state as suggested for additional experiments: validation experiments with chemotherapy drugs on organoids (Suppl. Fig 3a, NC-SPOT-MS1 (NCOMMS-23-61673-T)) and on single cell division, with well-known biological dynamics (Figure 7, NC-SPOT-MS2). Entropy quantifies the uncertainty of a cluster transitioning. Mathematically, a cluster has maximum entropy when it has equal probability of transitioning to any other cluster. We observe that high entropy clusters provide a fingerprint of the chemotherapy drug. Considering a cutoff of 0.5 nats, cluster 3 was highly entropic in the milder DMSO and Cisplatin drugs, cluster 1 for the more toxic 5FU and clusters 1,2,4, and 5 for the most toxic Docetaxel (Suppl. Fig. 3a, NC-SPOT-MS1). Importantly cluster 4 is the most toxic phenotype cluster with highest Caspase and Cytotox marker expression. Thus it seems high entropy might help identify the most informative clusters, those involved with the greatest number of cluster transitions, to help distinguish biologically relevant clusters versus 'noise' clusters. This observation appears equally to hold for cell division. Clusters 1, 4, 5, and 6 are identified as high entropy for SPOT-SAM features (Suppl. Fig. 7c, NC-SPOT-MS2). Importantly, each clique of the functional

similarity hierarchical clustering (Fig. 7c, NC-SPOT-MS2) is represented. In contrast, the high entropy clusters 1,3, 5, and for CNN-LSTM features (Suppl. Fig. 7d, NC-SPOT-MS2), are all not distinct, belonging to the same functional similarity clique (Fig. 7i, NC-SPOT-MS2).

8) Is this work related to other shape and motion work/patent of the first author? If yes, you shall explain the connection.

The other shape and motion work/patent of the first author is primarily captured in 5 publications:

1) Multiplexed 3D Analysis of Cell Plasticity and Immune Niches in Melanoma (*BioArxiv, 2024*)
    - Performs 3D segmentation of single cells in multiplexed tissue to help experimental researchers to identify immune interactions in patient tissue biopsies.
2) A general algorithm for consensus 3D cell segmentation from 2D segmented stacks *(BioArxiv, 2024)*
    - Describes a framework for shape representation to allow combining 2D segmented stacks from orthogonal views into a single 3D consensus segmentation
3) Surface-guided computing to analyze subcellular morphology and membrane-associated signals in 3D *(BioArxiv, 2023)*
    - Primarily concerns the unwrapping of surfaces of 3D single cells and monitoring of dynamic cellular protrusions over time.
4) Motion sensing superpixels (MOSES) is a systematic computational framework to quantify and discover cellular motion phenotypes (*eLife*, 2018)
    - Concerns the extraction motion signatures from an entire video without explicitly segmenting individual objects
5) Single-cell phenomics reveals behavioural and mechanical heterogeneities underpinning collective migration during mouse anterior patterning (*BioArxiv*, 2023)
    - Application of unwrapping and MOSES to identify distinct phenotypic behaviour of a subset of migrating cells during 3D embryogenesis.
    - This work extracts targeted shape features for analysis and analysis is guided by knowledge of the embryo geometry.

We regard these works as independent and tangential to the current work, which focuses on developing a general feature set and temporal analyses applicable for analysing all individually segmented 2D object dynamics.

9) for the phenotype-genotype coupling, since it is currently impossible to get both the phenotype and genotype of the same cell at the single cell level, what potential problem does the population-level coupling have? How will poor coupling/mis-alignment affect the study?

We completely agree with this reviewer that it is impossible to obtain the genotype-phenotype coupling at a single cell level. Although cells and organoids are highly

heterogenous, similar morpho-dynamic phenotypes can be reflective of cells or organoids who share similar genotypes and with similar biological functions. The premise we take in the current two manuscripts (NC-SPOT-MS1 and NC-SPOT-MS2) is that it is difficult to infer consensus patterns when considering only individual cell or organoid heterogeneity, whilst a simple average may not reveal sufficient differences. SPOT's proposal is to capture a middle ground of monitoring temporal changes in the distribution of heterogeneity relative to the baseline at timepoint 0, over a longer time horizon than the biological process being studied. Using our stacked barplots, we can begin to make functional inferences more reflective of the mechanistic action of a particular stimulus. Of course, any insights would then need to be supplemented with more targeted molecular assays such as that in Fig. 6 (NC-SPOT-MS1). The idea, however, is that SPOT could represent a relatively inexpensive way to identify those functional timepoints and phenotypes that maximally increase the likelihood of identifying mechanistic differences. These points have now been included in the discussions of both NC-SPOT-MS1 (NCOMMS-23-61673-T) (single cell datasets) and NC-SPOT-MS2 (organoids).

Reviewer #1 (Remarks on code availability):

The author provided the code and README, but the code is too complicated for me to run.

We apologize and thank the reviewer for the feedback. We have created step-by-step instructions for running the Python scripts with the provided example datasets. This is included as part of the GitHub repository as a .docx word file. We further make available the new organoid dataset with chemotherapy drugs and cytotox/caspase stains used in the revision, with its corresponding analysis code for further reference. The code will be continuously maintained in GitHub. Through GitHub issues, we welcome community suggestion and reporting of bugs.

REVIEWER #2 (REMARKS TO THE AUTHOR):

This scientific paper introduces a concept in the field of imaging analysis, termed the "imaging phenome," which is analogous to the transcriptome in single-cell transcriptomics. The authors propose a comprehensive framework to describe phenotypic diversity in dynamic objects through a standardized set of image descriptors. These descriptors are categorized under three major properties: Shape, Appearance, and Motion (SAM), each of which is considered as an individual imaging "transcript."
To materialize this concept, the paper details the establishment of a standardized SAM "phenome," comprising 2185 distinct SAM features. This extensive collection of features provides a robust and comprehensive method to characterize various dynamic objects in imaging studies. Alongside this, the authors have developed a sophisticated analytical tool called SPOT (SAM Phenotype Observation Tool). SPOT is described as an imaging "sequencer," capable of analyzing both label-free and fluorescent images.

The paper underscores the potential of this approach in enhancing the understanding of phenotypic diversity through detailed and standardized imaging analysis, akin to the insights provided by transcriptomic analysis in genetics.

I've read the paper with great interest and the paper is well written and the strategy is well explained. However, I cannot advice for publication of the manuscript in its current form and the scientific work needs several improvements. Nonetheless, I think it is an interesting strategy, although applying scRNA analysis tools to single organoid image analysis is not novel (https://doi.org/10.1038/s41698-023-00480-y). But I think the approach has a lot of potential, if applied correctly.

Reply:

We thank the reviewer for recognising the value of our approach and its potential to make an important impact to the field.

1) The manuscript is way too long and lacks focus. For me, these are at least two or three independent papers. (i) Development of the SAM tool and algorithms and testing it on the public databases. (ii) applying SAM to 2D cell culture (iii) applying SAM to 3D organoid culture. For readers, it is very difficult to extract the information that is relevant for them, and these three topics mostly have a different target audience.

2) I wonder how relevant the use of the public databases from youtube and MPEG-7 is. How relevant are these features in cell biology and using this to compare with CellProfiler seems strange.

3) Would a repository such as LIVECell not be more relevant to determine is SAM can accurately cluster distinct cellular morphologies rather than irrelevant images and videos (https://www.nature.com/articles/s41592-021-01249-6)?

Reply:

We thank the Reviewer for these constructive suggestions. Our motivation for choosing computer vision datasets was to use relatable everyday examples to demonstrate the need to design multiscale spatial features that capture shape, appearance, and motion simultaneously. We also wanted to develop a single feature set or 'phenome' that is applicable to videos of any 2D object which would enable like-for-like comparison between any analysed datasets across assays, conditions and labs.

To increase manuscript focus of organoids and to enable us to present key experiments performed on published 2D datasets leading to the development of our SAM phenome and SPOT, we are submitting two independent but complementary manuscripts, NC-SPOT-MS1 focussed on 3D organoid application and NC-SPOT-MS2 focussed on method development. NC-SPOT-MS1 (NCOMMS-23-61673-T) entitled ""**Identifying phenotype-genotype-function coupling in 3D organoid imaging using Shape, Appearance and Motion Phenotype Observation Tool (SPOT)**" retains a title similar to the original title of NCOMMS-23-61673-T. By removing the detailed description of the SAM-phenome construction and SPOT development, and analyses of computer vision and 2D cell imaging datasets, the revised NCOMMS-23-61673-T is now focused on the adaptation and applicability of SAM-SPOT to identify genotype-phenotype-function coupling in high-content 3D organoid cultures after generating 2D projection videos (new Figure 1), as suggested by this Reviewer. We recognise the value this reviewer places on testing whether ground truth morphologies can be accurately distinguished using a relevant reference dataset. To respond to this, we created a novel organoid simulator to generate synthetic 3-dimesional organoid movies with user-prescribed evolution of their shape, appearance, and motion over time. We used the simulator to generate large datasets of 2D projection videos with complex ground truth SAM phenotypes. Our results demonstrate the effectiveness of SPOT analysis in a highly relevant, organoid-specific context (new Fig. 2 and new Suppl. Fig. 2, SPOT-NC-MS1, NCOMMS-23-61673-T).

Our new NC-SPOT-MS2 entitled "Development of a universal imaging "phenome" using Shape, Appearance and Motion (SAM) features and the SAM Observation Tool (SPOT)" contains the non-organoid information from NCOMMS-23-61673-T, with substantial expansion of the details in constructing our SAM phenome and the development of SPOT analysis framework. NC-SPOT-MS2 provides detailed validation of SAM-SPOT on computer vision and YouTube video datasets with ground truth. Additionally, following this Reviewer's valuable suggestion, we assessed the applicability of SAM-phenome and SPOT to large biological public datasets with known phenotype information, LIVECell for static images and cell division for live-cell videos as suggested by reviewers. We further compared results to using convolutional neural network (CNN)-features and CNN-LSTM (long short term memory)-features after training these AI-based models on the datasets, using 1 million fixed single cell images and >5000 single cell video frames for training, respectively. We then tested the features in the respective datasets when used instead of our SAM phenome in training phenotype classifiers and for SPOT analysis using >400,000 fixed single cell images and >7000 single cell video frames, respectively. Our SAM-phenome significantly outperformed both CNN-features or CNN-LSTM-features in both tasks, training superior phenotype classifiers to

predict cell type or cell division stage, and providing a more informative and interpretable SPOT analysis, thanks to its rationally defined features and explicit categorization of those features by shape, appearance and motion. In summary, NC-SPOT-MS1 and NC-SPOT-MS2 comprehensively demonstrate the power, utility and compatibility of SAM-SPOT for genotype-phenotype coupling for high-content imaging-based screening.

4) My main concern is the images that are used for the organoids. SAM quantifies 2185 features, so it is very sensitive to imaging artifacts.

Reply:
Due to the limitation in space in our previous manuscript, we did not demonstrate that SPOT's design is versatile to reconcile artefacts within the analysis. In our new NC-SPOT-MS2, we now clarify our design rationale in the detailed description of the construction of SAM phenome. Specifically, the 2185 SAM features is the entire SAM phenome and is the resultant number of features after quantifying the parameters of shape, appearance and motion, at global, regional and local spatial scales. The high dimensionality includes intended redundancy across features, which builds robustness that guards against experimental and processing 'noise' errors.

We draw analogy in designing the SAM phenome to single cell sequencing and analysis vs traditional microarray/select biomarker assays. A single biomarker is interpretable but often is not always expressed nor necessarily specific to the desired cell type due to biological stochasticity and measurement noise. Using a panel is the gold standard to perform cell deconvolution and cell type calling and is much more robust. Employing the same philosophy, the SPOT workflow builds on the designed redundancy within SAM features to include many processing steps to further guard against artefacts and to maximize biological discovery. Systematic and multi-stage feature selection is employed on a per experimental basis and this is done by filtering to remove potentially unreliable features that exhibit either too high a statistical variation or those that are zero-valued across all segmentations (and thus which contribute no information). Since motion (differential change over time) is one of the key parameters of SAM features, and a key advantage of live-cell imaging, a feature selection based on regression with time enables us to further identify and remove features associated with systematic errors, as imaging artefacts should not drive temporal variation in the dataset. Thus, by selecting and retaining those SAM features that exhibit temporal variation, SPOT maximizes discrimination between conditions when considering the population-averaged temporal changes or stacked barplots whilst minimizing imaging artefacts. Therefore, in reality, whilst SPOT always computes 2185 SAM features initially, automated feature selection means only a fraction is adapted and used to analyse each experiment. If we instead under-compute features, the information is irrecoverably lost in analysis. If we compute many more features, individual phenomes will be 'too unique' and also worsen performance (See Discussion in new NC-SPOT-MS2). SPOT's preprocessing is analogous to the standard way in which only top-expressing transcripts are used to analyse single-cell experiments, where the full transcriptome still needs to be measured in the raw data. The advantage and the power of computing comprehensive and redundant SAM features from multiple spatial scales, the SAM phenome, versus only selected feature subsets (Suppl. Table 2, and Discussion, NC-SPOT-MS2) is the basis of why SAM phenome significantly outperforms

and is more broadly-applicable than purely data-driven AI-based features both in classification and in providing more informative phenotypical measurements (Figure 6, NC-SPOT-MS2).

5) Unfortunately, the imaging and culturing strategy that was applied is not compatible with SAM.
Organoids are grown in ECM domes, therefore they are not in the same plane which is a minimal requirement for this approach. There are various methods and plates available to image organoids in a single plane such as microwell plates (e.g. Gri3D) or flat-bottom 384-well ULA plates (https://www.jove.com/v/64434/multiparametric-tumor-organoid-drug-screening-using-widefield-live).

Reply:
We thank the reviewer for the careful reading and we agree with this reviewer that "*organoids are grown in ECM domes, therefore they are not in the same plane*". We used various culturing methods and plate types in this study according to the live cell imaging acquisition requirements, as presented now in new Suppl. Table 3, NC-SPOT-MS1 (NCOMMS-23-61673-T). Regardless of culturing method, organoids lie in different planes. To reconcile this in our z-stacks (primarily 11 z-slices) which offer minimal 3D information (Suppl. Fig. 1a, 1b, NC-SPOT-MS1 (NCOMMS-23-61673-T)), we maximize the number of in focus organoids within the dome shaped matrigel in one 2D image by performing 2D extended focus projection, also called focus stacking for label-free microscopy and maximum intensity projection for fluorescent microscopy videos. The new Suppl. Fig. 1a illustrates how in label-free microscopy images, extended focus which projects organoids in different planes to 2D based on the strength of their image gradient is better than maximum intensity projection in generating in-focussed organoids in the resulting 2D image so that we can measure biologically relevant SAM features. We have now updated our Methods and Supplementary Materials section to ensure that all image acquisition details are included. Additionally, we also specified the tissue culture plates used in each of the experiments presented. These are summarised in the below table (new Suppl. Table 3, NC-SPOT-MS1):

Figure	Plate	Z-stack
Figure 3	Corning® Costar® TC-Treated Multiple Well Plates	Z-stack images 1000 µm in depth were acquired at 100 µm intervals (11 steps per acquisition) every hour for 5 days.
Figure 4	384-well plate, Flat bottom, rounded square wells	Z-stack images 1225 µm in depth were acquired at 35 µm intervals (35 steps per acquisition) every 2 hours for 5 days at each of the chosen positions.
Figure 5	Nunc MicroWell 96-Well Optical-Bottom Plates with Polymer Base, Cell culture, black	Z-stack images 1000 µm in depth were acquired at 100 µm intervals (11 steps per acquisition) approximately every hour for 14 days.
Supp Fig 1a	Nunc MicroWell 96-Well Optical-Bottom Plates with Polymer Base, Cell culture, black	Z-stack images 1000 µm in depth were acquired at 100 µm intervals (11 steps per acquisition) approximately every 60 min for 14 days.
Supp Fig 1b	384-well plate, Flat bottom, rounded square wells	Z-stack images 1225 µm in depth were acquired at 35 µm intervals (35 steps

		per acquisition) every 2 hours for 5 days at each of the chosen positions.
Supp Fig 3a.c	Nunc MicroWell 96-Well Optical-Bottom Plates with Polymer Base, Cell culture, black	Z-stack bright-field images covering 1000 µm total depth were acquired at 100 µm intervals (11 steps per acquisition) every 4 hours for 5 days.
Supp Fig 3b, Supp Fig 3c	"Plastic transparent" plates: Corning® Costar® TC-Treated Multiple Well Plates<br><br>"Glass-bottom" plates: Nunc MicroWell 96-Well Optical-Bottom Plates with Polymer Base, Cell culture, black	Z-stack bright-field images covering 1000 µm total depth were acquired at 100 µm intervals (11 steps per acquisition) every 4 hours for 5 days.
Supp Fig 3d	Corning® Costar® TC-Treated Multiple Well Plates	Z-stack images of 1000 µm in depth were acquired at 100 µm intervals (11 steps per acquisition) approximately every 15 minutes for 5 days.
Supp Fig 4a	Nunc MicroWell 96-Well Optical-Bottom Plates with Polymer Base, Cell culture, black	Z-stack bright-field images covering 1000 µm total depth were acquired at 100 µm intervals (11 steps per acquisition) every 4 hours for 5 days.
Supp Fig 5	Nunc MicroWell 96-Well Optical-Bottom Plates with Polymer Base, Cell culture, black	Z-stack images 1000 µm in depth were acquired at 100 µm intervals (11 steps per acquisition) approximately every 60 min for 14 days.

6) From image 3B it is very evident that the clusters are mainly based on imaging artefacts. For example, cluster 3 are out of focus organoids, cluster 2 are overexposed organoids, cluster 7 are underexposed organoid, cluster 4 out of focus and underexposed… Although there might be some phenotypic differences, features extracted from these images are completely unreliable due to the imaging artefacts.

We thank the Reviewer for this comment and apologize for not being sufficiently clear in our description and rationale of 'phenotype cluster'. The term 'phenotype clusters' does not strictly refer to biologically meaningful phenotypes. A detailed description of how the analytical workflow of SPOT functions is now included in Figure 2, new NC-SPOT-MS2. Technically, the phenotype clusters coarsely partition the UMAP phenotypic landscape such that single cell (NC-SPOT-MS2) and organoid (NC-SPOT-MS1) instances within each cluster share more similar instantaneous SAM features. This does not necessarily mean each cluster captures a unique biological phenotype, nor does this exclude multiple clusters describing the same biological process e.g. cell death (Fig. 3, NC-SPOT-MS1 (NCOMMS-23-61673-T)), Fig. 4 YouTube video objects, NC-SPOT-MS2). This also means that some clusters may be attributed to a dominant image artefact e.g. blur. The phenomic landscape is a map of SAM heterogeneity across all time and conditions in a single 2D coordinate space. Whilst phenotype clusters serve as the discrete representation of SAM heterogeneity. The primary biological analysis of SPOT is to assess how phenotype heterogeneity changes temporally, as captured by the phenotype trajectory, phenotype transition and stacked barplot analysis, to make comparative inferences about treatment conditions. In sum, SPOT makes the

fundamental hypothesis that imaging artefacts are systematic, and ergodic (i.e. not time-dependent). Therefore, we expect pure imaging-artefact-only 'phenotype clusters' to exhibit minimal population changes over time, to not be the primary driver for the temporal evolution of phenotype, and to affect all conditions equally. Our additional validation experiment with chemotherapy drug, including the use of Caspase marker, show how multiple phenotype clusters (small organoids and large organoids) can correspond to cell death (Suppl. Fig. 3a, NC-SPOT-MS1). Moreover, independent SPOT analysis of organoids cultured in glass or plastic plates show the same broad classification of drug toxicity (less toxic: DMSO and Cisplatin vs more toxic: 5FU and Docetaxel), (Suppl. Fig 3b, NC-SPOT-MS1). Finally, the ability of SPOT to handle artefacts is further demonstrated using our 3D organoids simulator shown in the new Figure 2, NC-SPOT-MS1.

With respect to the specific comments about clusters shown in Fig. 3b, NC-SPOT-MS1 our interpretations of the results are listed below.

- *Cluster 3 and 4 organoids are out-of-focus:*

These organoids correspond to the same organoids that lie outside our imaged depth of 1000 µm. In the respective density plot (Fig. 3c ii), we see the primary density is located outside of the UMAP areas covered by clusters 3 and 4, and in the stacked barplot (Fig. 3c iii), we see that clusters 3 and 4 are relatively temporally constant subpopulations across time, similar across conditions and largely transiting only between themselves (Fig. 3c iv). The direction of the phenotype trajectories over time does not move towards the UMAP region covered by cluster 3 and 4 in any of the treatments, demonstrating that these 'artefact' clusters do not hinder biological interpretation (Fig. 3c v).

- *Cluster 2 organoids are overexposed:*

These organoids are in fact not overexposed: they have a transparent lumen which means that they are measured as being relatively brighter. In addition, e.g., from Fig. 3d, it can be seen that the intensity (SAM module 3) of Cluster 2 is less than Cluster 1. We know that this cluster reflects growing organoids because the stacked barplot (Fig. 3c iii) shows that its relative proportion expands over time in a stable manner, reflecting the fact that smaller organoids that were not previously classified as cluster 2 are now part of cluster 2 following their growth. We also know biologically that healthy, growing gastrointestinal organoids are transparent. They become dark and opaque with excessive cell death, leading to accumulation of cell death in the lumen. Suppl. Fig. 3a with Caspase and Cytotox verify the primary signatures of cell death as being darker and reduced movement.

- *Cluster 7 organoids are underexposed:*

In contrast to what is suggested by the Reviewer, these organoids are true phenotypes due to cell death where material is accumulated in the normally transparent lumen and the organoid shrinks/buckles in on itself (See Suppl. Fig. 3a with Caspase and Cytotox markers). We see this strongly in the phenotype trajectory following treatment with toxic drugs, which moves towards the UMAP regions covered by cluster 7 over time (Fig. 3c v). Correspondingly, the stacked plots show a much higher end proportion of cluster 7 organoids following treatment with highly toxic drugs (Fig. 3c iii). Again, this underscores the importance of analyzing live-cell imaging and considering the temporal changes.

7) The label-free organoid segmentation is not accurate enough, and tracking of the organoids is not consistent over time (e.g. video 4) or the density of the organoids is way too high (e.g. video 5). Organoids merge together, overlap, get detected – undetected,… so there is no consistency that allows for kinetic measurements crucial for SAM. Again, this can all be resolved by the use of proper imaging-compatible culturing techniques and plates.

We thank the Reviewer for this comment and our newly generated synthetic organoid datasets with defined phenotypic behaviour have provided answers to this important criticism. In particular, we simulated the effect of increasing segmentation errors including overlap, cutout and union and tracking errors through fragmenting organoid tracks on SPOT analysis (Suppl. Fig. 2c, NC-SPOT-MS1). Our results show SPOT is robust to increasing amounts of these artefacts.

Additionally, we would like to clarify the approach in SPOT to ensuring temporal consistency and minimizing errors when generating the final organoid instances to compute SAM features: 1) we only need two frames to compute motion features; 2) organoid instances are only retained if the intersection-over-union of the segmentations frame-to-frame in the whole track is at least >0.25, which we find is an effective threshold for matching, taking into account an acceptable margin for shape changes over the elapsed hour between successive timepoints; 3) we temporally smooth segmentations over a time window which enables both temporal consistency and imputation of partial segmentations; 4) except for the centroid displacement features (only two features), the remaining motion features are based on the pixel-dense motion field, where every pixel of an image has an associated motion vector as computed independently with optical flow techniques. Importantly, optical flow does not depend on segmentation and its error can be constrained by incorporating physical constraints such as ensuring smoothness of motion field and constraining the size of the displacements; 5) the principle of SPOT to monitor the relative temporal change i.e., the delta in the measured heterogeneity over time across the whole population between different conditions. This is shown by our choice of clustering the phenotype trajectories using the dynamic time warping metric to compare trajectory shape instead of using absolute positional coordinates in the UMAP phenotypic landscape. Even if individual segmentation is inaccurate and noisy, as long as these errors are systematic over time, do not exhibit temporal variation, and average out over the population, we expect SPOT to detect relevant biological phenotypes. This is shown by our ability to resolve the effect of WNT addition, despite the organoid density, as independently validated by scRNA-seq (Fig. 5 and 6, NC-SPOT-MS1 (NCOMMS-23-61673-T)).

We would like to emphasize that the WNT biology that we are interested in is dependent on local organoid density. Moreover, the organoid flattening phenotype we observe is not local and covers spatial regions much larger than stereotypical spherical organoids. Here, the concept of organoid detection and segmentation does not apply and Particle Image Velocimetry (PIV) type of approaches are more applicable. Yet, SPOT is still able to resolve the phenotypes because we include comprehensive and redundant SAM measurements. If SPOT were only applicable to spatially delineated organoid cultures and relied upon accurate segmentation and tracking then it would have limited application to discovering novel phenotypes. It is important to note that tissues are densely packed with cells with extensive

cell/cell interactions, therefore certain key biological information will be lost in uniformly formed isolated organoids.

8) I'm wondering how much features are actually relevant. For example, for figure 3, studying the drug response in the organoids, the same conclusion can be drawn with just basic imaging features and do not require quantification of 2184 features. So for basic drug screening I think this is overkill.

We thank the Reviewer for this comment. We agree that for a basic toxicity screen, the number of features is overabundant. However, a central premise in our manuscript is whether we can compute a universal set of SAM features in the same universal manner that is equally applicable for analyzing simple (cell death) and complex scenarios (changes in organoid branching patterns). Whilst specifying a few targeted measurements such as biomarkers is simpler and more direct , it is not robust and interpretation may further be confounded by conflicting results. In Suppl. Fig. 3a (NC-SPOT-MS1), both Caspase and Cytotox are biomarkers of cell death yet they do not fully agree with each other. We also found we had to count the number of punctate spots instead of taking the mean intensity to get an interpretable quantification. For high-content and high-throughput applications, specifying more features then performing feature selection has proven to be far more discriminative for discovery. For example, agnostic sequencing of all available transcripts is essential for scRNAseq to discover new cell types and corresponding biomarkers compared to classical microarrays. We have now outlined more clearly the rationale for our 2185-feature approach in new NC-SPOT-MS2 (Figure 1c).

9) In addition, the authors make statements about that certain clusters correlate with for example high levels of cell death (line 575) but no evidence of cell death is provided (e.g. fluorescent cell death marker). So I understand unbiased clustering of features is interesting, but it would be more interesting if they can be linked **to processes such as viability, cell death, apoptosis,… by using corresponding fluorescent markers.** ***This way you can determine SAM features related to specific biological processes and give meaning to a cluster (similar to linking scRNA seq clusters to biological pathways based on genesets).***

To demonstrate that SPOT phenotypic clusters can be associated to a biological process, we have performed the Reviewer's suggested additional staining experiments using caspase and cytotox to check the cell viability, cell death and cell apoptosis in the chemotherapy drug screen (new Supp. Fig 3a, 3b, 3c).

Our initial SPOT analysis suggested 3 toxicity groups (Fig. 3, NC-SPOT-MS1). We therefore selected the control DMSO and Cisplatin (6 µM and 100 µM) to represent the low-toxicity group, 5-Fluorouacil (5FU) (20 µM) for the mild toxicity group, and Docetaxel (12.5 nM) for the high-toxicity group. The concentrations chosen are the maximum level concentration used in Fig. 3, NC-SPOT-MS1. The organoids were grown for 6 days in a 96-well plate. The medium was changed with HOM and a titration of the drugs and the dyes as outlined below. Z-stack bright-field images and confocal images 1000 µm in-depth were acquired at 100 µm intervals (11 steps per acquisition) approximately every 4 hours for 5 days. Previously images

were acquired every 1 hour for 5 days. The slower acquisition was used to reduce phototoxicity and to account for the additional imaging due to increased channels.

1. The cell viability during the drug screening was tested with Hoechst 33,342 (50 nM/well, ThermoFisher) consistent with literature (*OrBITS: label-free and time-lapse monitoring of patient derived organoids for advanced drug screening. Cellular Oncology. 2022*);
2. Real-time quantification of cell death induced by the toxicity of the drugs was assessed with **Cytotox Green** (60 nM/well, Sartorius, #4633), (*Multiparametric Tumor Organoid Drug Screening Using Widefield Live-Cell Imaging for Bulk and Single-Organoid Analysis. J. Vis. Exp. (190), e64434, doi:10.3791/64434 (2022))* and the cell death was calculated based on the number of detected punctate spots within each segmented organoid;
3. The evaluation of cell apoptosis during the drug screening was assessed with **Caspase 3/7 Green** (2.5 µM/well, Sartorius, #4440), (*OrBITS: label-free and time-lapse monitoring of patient derived organoids for advanced drug screening. Cellular Oncology. (2022); Viability Analysis and High-Content Live-Cell Imaging for Drug Testing in Prostate Cancer Xenograft-Derived Organoids. Cell. 2023)* by counting the number of detected punctate spots within each segmented organoid.

We performed SPOT analysis with the acquired datasets using our proposed SAM phenome only applied to the brightfield microscopy channels, then using the fluorescent Cytotox and Caspase biomarkers afterwards for interpretation. Our results, shown in the new Suppl. Figure 3a, NC-SPOT-MS1 show that SPOT identified 7 phenotype clusters of which clusters 2, 4, and 7 correlated with higher toxicity, with elevated number of measured Caspase and cytotox punctate spots. Interpreting with SAM modules, these clusters correspond two different death phenotypes: the normal accumulation of cell death in the lumen as organoids grow too large (clusters 2 and 4) and the drug-induced cell death whereby organoids remain small or potentially already dead (cluster 7). We found the total number of spots within a segmented organoid (see Methods) was a more reliable and informative measurement than green area/brightfield area due to the nonspecific auto fluorescence. Toxicity was measured highest for Docetaxel, which we had predicted by SPOT with SAM signature of low intensity and reduced movement. Moreover, our UMAP organized both Caspase and Cytotox markers similarly and in a gradient manner. Subsequently, hierarchical clustering of phenotype trajectory showed greater similarity of cisplatin to DMSO, then 5-FU, with Docetaxel most different, consistent with our findings of the larger screen with multiple drug concentrations in Fig. 3, NC-SPOT-MS1. Altogether our analysis demonstrates how SPOT uses multidimensional features and temporal change to robustly stratify toxicity phenotypes compared to using select single/few biomarkers which may itself be subject to experimental variation, and how they are used to extract measurements.

10) The authors use the term 'flattened organoid morphology' (line 765). It is difficult to see from the image, but are these cells that adhere to the plastic surface and start growing in 2D? This might be evidence of a more invasive, migratory behaviour.

We performed fixed staining at the endpoint of APC, KRAS, and p53 mutant mouse colon organoid movies with confocal z-stacks to assess and verify the observed organoid

morphology from 2D projection. Using depth coloured maximum intensity projection (DCP), Suppl. Fig. 4b (NC-SPOT-MS1) shows the morphological transformation in the APC mutant localizes preferentially at the interface of matrigel/substrate or boundaries of the matrigel ECM dome (red and blue coloured organoids in DCP). We agree with the Reviewer that this may reflect a more invasive, migratory behaviour. Both p53 and KRAS mutants are more spherical and lie more centrally in the ECM dome (yellow/green coloured in the DCP).

11) While I find that the current version of the manuscript does not meet the necessary criteria for publication in its present form, I would like to emphasize the valuable insights and potential contributions your work offers. The breadth of the topics covered is commendable, yet a more focused approach could enhance the clarity and impact of your research. I strongly encourage you to consider refining the scope of your paper or split up the paper into two or three papers, sharpening the central arguments, and addressing the highlighted issues. A revised version that addresses these aspects would be a welcome submission to this journal. Your efforts in advancing research in this field are appreciated, and I look forward to potentially seeing a revised manuscript, in particular with a focus to organoids. I would like to express openness to potential collaboration. In my current research, we have developed an extensive library of organoid images treated with a variety of therapies. If you find it relevant, I believe a collaborative effort could enrich both our research endeavors, leveraging our respective resources and expertise.

Yours sincerely,
Prof. dr. Christophe Deben

We would like to thank the Reviewer again for recognising the value of our work and its potential to advance the field. We would also like to thank them for their constructive comments, which we have aimed to address here, and which have substantially improved the clarity of our manuscript. Once the manuscript is published, we will be delighted to collaborate with the Reviewer.

Reviewer #3 (Remarks to the Author):

1) The paper describes an extension to the toolbox of researchers that appears to outperform the established standard, namely CellProfiler. The authors argue that the introduction of motion in their pipeline, and a determination between global, local-regional and pattern-pixel levels are key differentiators for success. The authors support this statement well in text and figures, namely through the reporting of contribution scores for their clustering interpretation.

We thank the reviewer for their positive comments.

2) The validation work of the developed tool, SPOT, also revealed a potential new insight into the working of the WNT-pathway. The authors demonstrated that for two contradicting configurations, WNT containing mouse organoids and WNT deprived human organoids, the observed morphological behavior to have a similar underlying cause. The authors support this with single-cell-sequencing and investigated phototoxicity as a potential cause of these events. I believe this to be a new finding, with a clear and soundly executed methodology.

We thank the reviewer for highlighting the robustness of our methodology and the novelty of our approach.

3) The currently proposed tool, SPOT, has been validated on multiple 2-dimensional datasets. The authors do show the potential of spot for organoid segmentation from Matrigel droplets, a 3-dimensional environment where organoids significantly overlap in the image. It is however currently unclear whether SPOT will work well for 3-dimensional time-series data. A possible complication can be that the motion module in SPOT can confuse X/Z movement for a phenotypic response of cells.

To test the applicability of SPOT analysis of 2D projections to characterize 3D organoid dynamics, we generated simulated organoid Dataset B. Organoids with prescribed shape, appearance and motion 3D dynamics were individually simulated and positioned in 3D scenes. The 3D scenes were then projected as 2D videos for SPOT analysis. We simulated three different 3D motion scenarios: the first condition is of full unrestricted 3D XYZ movement; the second condition restricts movement to 2D XY planar movements only but with the same speed as the first condition; the final third condition is a replicate of full unrestricted 3D XYZ movement with different simulated organoids. We find that whilst visually the phenotype trajectory and stacked barplots of phenotype cluster frequency over time appear indistinguishable, SPOT nevertheless detected differences and reliably clusters the XYZ movement conditions distinctly from the XY planar movement condition, (Suppl. Fig. 2d, NC-SPOT-MS1). We thus conclude the ability of SPOT to reliably differentiate the subtle visually undetectable Z movement demonstrates the suitability of SPOT for 3D time series analysis from 2D projection videos.

4) Overall, the paper is very well written, providing a new toolbox to researchers that has the potential to become of significant value to the field.

We thank the reviewer for their comments and for highlighting the value that our technology will bring to the field.

NC-SPOT-MS1		
**Current Figure**	**Previous Figure (NCOMMS-23-61673-T first submission)**	**Description**
Figure 1	New	Comprehensive shape, appearance and motion (SAM) characterization enables analysis of phenotypic heterogeneity in organoid models.
Figure 2	New	SPOT analysis of synthetic organoid video dataset with prescribed shape, appearance and motion dynamics.
Figure 3	Figure 3	SPOT phenotype analysis of human patient-derived duodenum organoids drug response enables clustering of drugs by pharmacological action.
Figure 4	Figure 4	SPOT identifies unique phenotypic differences in genotype defined murine colon organoids.
Figure 5	Figure 5	SPOT captures the timing of WNT signaling-induced phenotypic dynamics in patient-derived duodenal organoids.
Figure 6	Figure 6	SPOT complements molecular sequencing for scientific and biomedical discovery
Suppl Figure 1a	New	Brightfield phase contrast imaging has limited depth resolution and extended focus projection of a z-stack to a 2D image
Suppl Figure 1b	New	Acquired confocal z-stacks have depth-limited resolution.
Suppl Figure 2a	New	SPOT Phenotype cluster transition and density maps of the simulated organoid dynamics dataset A.
Suppl Figure 2b	New	Robustness of SPOT analysis to imaging artefact, segmentation and tracking errors
Suppl Figure 2c	New	Quantitative assessment of SPOT analysis robustness to imaging artefact, segmentation and tracking errors
Suppl Figure 2d	New	SPOT analysis of 2D projections discriminates z-component motion.
Suppl Figure 3a	New	SPOT phenotype clusters capture different SAM characteristics of cell death after drug treatment.
Suppl Figure 3b	New	Independent SPOT analysis of organoid culture in plastic transparent and optical-bottom plates

Suppl Figure 3c	New	Joint UMAP analysis of chemotherapy treated patient-derived organoids cultured in plastic and glass-bottom plates
Suppl Figure 3d	Extended Fig. 3	SPOT characterizes changes in murine small intestine organoid branching dynamics when treated with HDAC inhibitors.
Suppl Figure 4a	Extended Fig. 4	SPOT draws the same conclusions with label-free imaging of genotype defined murine colon organoids.
Suppl Figure 4b	New	Murine colon organoids exhibit flattened morphologies preferentially at boundary of ECM dome
Suppl Figure 5	Extended Fig. 5	SPOT detects changes in WNT signaling-induced phenotypic dynamics in patient-derived duodenum organoids

NC-SPOT-MS2		
**Current Figure**	**Previous Figure (NCOMMS-23-61673-T first submission)**	**Description**
Figure 1	Figure 1a, b	Shape, appearance and motion phenome characterize imaging phenotypes
Figure 2	Figure 1d	Development of SPOT
Figure 3	Extended Figure 1a-g	Testing Shape, appearance features on public datasets
Figure 4	Extended Figure 1h-j; New	Testing combined SAM on YouTube dataset
Figure 5	Figure 2	Single cell tracking challenge migration dataset
Figure 6	New	SAM-SPOT and CNN-SPOT analysis of LIVECell dataset
Figure 7	New	SAM-SPOT and CNN-LSTM-SPOT analysis of single cell division dataset (HeLa histone H2B)
Suppl Figure 1	New	Assessing the number of contour points required for shape representation
Suppl Figure 2a	New	SPOT module analysis compared with scRNA-seq analysis
Suppl Figure 4c	New	Schematic of SAM module analysis and Shape, Appearance, Motion contribution calculation
Suppl Figure 3	Suppl Figure 1	Comparison of SAM and Cellprofiler features to datasets
Suppl Figure 4a	Suppl Figure 2	A2D dataset similarity graph of joint actor-movement
Suppl Figure 4b	Suppl Figure 3	A2D dataset with different dimensionality techniques
Suppl Figure 5	Extended Figure 2	Single cell division dataset, Cell tracking challenge
Suppl Fig 6	New	SAM-SPOT and CNN-SPOT analysis of LIVECell dataset
Suppl Fig 7	New	SAM-SPOT and CNN-LSTM-SPOT analysis of single cell division dataset (HeLa histone H2B)

Reply to Reviewers' main concerns:

The Reviewers raised three main concerns:

1) SPOT's ability to deal with the presence of artefacts in the analysis;

2) the impact of potential inaccuracies in object segmentation and tracking; and

3) SPOT's suitability for 3D time-series imaging data.

To address all three of these concerns systematically and objectively, we built an organoid simulator to synthesize simulated *de novo* video datasets with known ground truth dynamic phenotypes. Each organoid can be prescribed with defined and dynamic organoid phenotypes, to simulate changes in only one or more of shape, appearance, or motion characteristics. Below, we summarise our response to the Reviewers' three primary concerns:

1. ***Potential sensitivity to artefacts:***

Reviewer 2: ***My main concern is the images that are used for the organoids. SAM quantifies 2185 features, so it is very sensitive to imaging artifacts.***
From image 3B it is very evident that the clusters are mainly based on imaging artefacts. For example, cluster 3 are out of focus organoids, cluster 2 are overexposed organoids, cluster 7 are underexposed organoid, cluster 4 out of focus and underexposed… Although there might be some phenotypic differences, features extracted from these images are completely unreliable due to the imaging artefacts.

Reply:
We apologise for not making it clear that including and reconciling artefacts within SPOT's analysis is a unique designed feature. The phenotype clustering is designed to identify different 'modes' or SAM imaging 'states' in the dataset by discretely partitioning the UMAP phenomic landscape. Each cluster is not necessarily a biological phenotype, and more than one cluster could relate to the same biological process e.g., two clusters relate to cell death. The primary biological analysis is in the phenotype evolution of organoids between conditions. SPOT assumes that over time, only phenotype clusters corresponding to true biological phenotype dynamics will exhibit differential temporal evolution, and this gives a signature to cluster conditions. Not all analysis uses this clustering. SAM phenotype trajectories are derived by the majority UMAP density in each temporal bin. In all cases, artefacts, when present, are assumed to not exhibit temporal evolution and to affect all conditions equally. We demonstrate the interpretation of phenotype cluster in using the simulated organoid dataset (new Fig. 2) and new supplementary chemotherapy drug screens analysed with the addition of a fluorescent caspase marker (new Suppl. Fig. 3a). In the latter, we found at least three SAM phenotype clusters associated with elevated caspase expression, indicative of different modes of death.

Imaging artefacts are almost always present in some manner during timelapse acquisition. Importantly, these artefacts differ between imaging modality and imaged subject. As a tool designed for high-content analysis, we believe that it would be a limitation to have to firstly

carefully remove artefacts, which requires domain expertise and potentially extensive manual proofreading. Naturally, if the user has trained an appropriate artefact detector for their data, this can be applied to further improve analysis and enhance biological interpretation. Nevertheless, as we demonstrate by analysing simulated organoids subject to defined artefact type perturbations in the new Suppl. Fig. 2, that SPOT is either robust to an artefact type (such as blurring and intensity changes due to photobleaching), or, it makes the artefacts easily identifiable in the 2D UMAP to allow data subsetting. For example, when we perturbed the ground truth simulated dataset with 'union' segmentation errors of varying severities (illustrated in Suppl. Fig. 2b a), the UMAP cleanly partitions the ground truth from the perturbed conditions. We can thus subset for and reperform analysis on only the unaffected images (Suppl. Fig. 2b c).

2. Potential inaccuracies in object segmentation and tracking

Reviewer 1: The success of the whole pipeline is dependent on accurate object tracking/tracklet. Can you quantify the accuracy of this step of your pipeline and discuss how this accuracy will affect the downstream results numerically?

Reviewer 2: The label-free organoid segmentation is not accurate enough, and tracking of the organoids is not consistent over time (e.g. video 4) or the density of the organoids is way too high (e.g. video 5). Organoids merge together, overlap, get detected – undetected,… so there is no consistency that allows for kinetic measurements crucial for SAM. Again, this can all be resolved by the use of proper imaging-compatible culturing techniques and plates.

Reply:
SPOT inherently includes safeguards that may affect analysis. With organoid detection, SPOT enforces a minimum temporal consistency in segmentation: all instances of a tracked organoid must have a frame-to-frame shape overlap (as measured by intersection-over-union (IoU)) greater than a user specified threshold. If this cut-off is too high, matching may be too stringent to shape changes between frames. We use a cut-off of IoU=0.25. We also emphasise that the tracking is implemented only to identify genuine organoids (alive or dead) which we define as being tracked for a minimum number of frames (here, 5 frames) from potential short-lived transient artefacts e.g., floating debris. In SPOT analysis, the tracked organoids at different timepoints are mapped as independent individual points in the UMAP. Only the motion features require tracking to compute. Even so, it depends only on a consecutive pair of accurate segmentations. In summary, SPOT is expected to be robust even if not all object instances are perfectly segmented (due to overlap for example), and robust in the case where some object instances are undetected or filtered out (such as by the IOU tracker, causing fragmented tracks). We validate our claims using our simulated 3D organoids, by introducing varying degrees of segmentation errors 'cutout' and 'union'), that could result from overlap. If such instances occur and are highly prevalent, we find they cluster distinctly and can be removed (new Suppl. Fig. 2b c). In addition, we simulated the effect of various degrees of track fragmentation (such as that caused by organoid instances remaining undetected or being filtered out), showing that the conclusions drawn from SPOT analysis remain unaffected (new Suppl. Fig. 2b d).
SPOT inherently includes safeguards that may affect analysis. With organoid detection, SPOT enforces a minimum temporal consistency in segmentation: all instances of a tracked

organoid must have a frame-to-frame shape overlap (as measured by intersection-over-union (IoU)) greater than a user specified threshold. If this cut-off is too high, matching may be too stringent to shape changes between frames. We use a cut-off of IoU=0.25. We also emphasise that the tracking is implemented only to identify genuine organoids (alive or dead) which we define as being tracked for a minimum number of frames (here, 5 frames) from potential short-lived transient artefacts e.g., floating debris. In SPOT analysis, the tracked organoids at different timepoints are mapped as independent individual points in the UMAP. Only the motion features require tracking to compute. Even so, it depends only on a consecutive pair of accurate segmentations. In summary, SPOT is expected to be robust even if not all object instances are perfectly segmented (due to overlap for example), and robust in the case where some object instances are undetected or filtered out (such as by the IOU tracker, causing fragmented tracks). We validate our claims using our simulated 3D organoids, by introducing varying degrees of segmentation errors 'cutout' and 'union'), that could result from overlap. If such instances occur and are highly prevalent, we find they cluster distinctly and can be removed (new Suppl. Fig. 2b c). In addition, we simulated the effect of various degrees of track fragmentation (such as that caused by organoid instances remaining undetected or being filtered out), showing that the conclusions drawn from SPOT analysis remain unaffected (new Suppl. Fig. 2b d).

3. *Potential issues with SPOT's use for 3D time-series data*

Reviewer 1: The 3D organoid imaging produces z-stack 3D images. Is it too rough to simply project the z-stack to one 2D image as in line 1476? You may want to as least discuss on how to better treatment 3D images.

Reviewer 3: Whether SPOT will work well for 3-dimensional time-series data, a possible complication can be that the motion module in SPOT can confuse X/Z movement for a phenotypic response of cells.

Reply:

We agree with the Reviewers that our study presented here is mainly based on projected 2D images from time-series data, and we recognize the limitation of the current version of SPOT to capture the full richness of 3D biology. The organoids we culture in our studies over 2 weeks grow large such that true 3D imaging acquisition of entire organoids at near-isotropic resolution would require sophisticated and customized light-sheet imaging setups, involving multiple objectives to try to overcome depth-based light scattering. These costly setups are not able to capture all organoids in the field-of-view in a high content nor high through-put manner, nor are they widely accessible. Therefore, we feel that only for particular biological hypotheses would an imaging tool that can accurately analyse true 3D time-series data at the cell/organoid scale be needed currently. Moreover, capturing full 3D significantly enhances phototoxicity and limits both total acquisition time and acquisition frequency.

Organoids are grown in 3D and have emerged as a valuable experimental tool for high-throughput genetic and compound screens because they can recapitulate the cell lineages of their origin organ. A major application is identifying earlier markers of disease transformation. However, organoids take time to develop as the cells require time to differentiate. Consequently, it is much more valuable in live-imaging organoid experiments to take steps to minimize phototoxicity and maximize acquisition length. The standard practice in both

pharmaceutical industry and research communities is for label-free microscopy such as bright-field and phase contrast microscopy modalities with small z-stacks. We follow this practice in this work, by imaging a few z-slices for up to 2 weeks and maintaining a relatively high acquisition rate of one frame per hour. In this paper, this imaging acquisition enabled us to reveal developmental differences due to differences in WNT signalling, in addition to the fact that these differences required several days to manifest, beyond the capability of commercial fluorescent lightsheet systems (Fig. 5). Moreover, as we highlight in Suppl. Fig. 1a,b our data whether label-free or confocal have insufficient z-resolution and offers no real further insight beyond 2D analysis, even if we were to extend SAM-SPOT to perform a full 3D analysis.

To further address the Reviewers' concerns on the applicability of analysing 2D projection videos of z-stacks, we generated synthetic organoid datasets (see below). Simulated dataset B was specifically designed to assess the potential added value of capturing full 3D dynamics. Our results shown in the new Fig. 2 illustrates that SPOT captures sufficient information in 2D projection videos to objectively discriminate between a condition where full 3D xyz movement was permitted from a second condition where movement was constrained to the 2D xy-plane.

4. *Creation of organoid simulator and synthetic datasets to validate the applicability and potential of SPOT*

We created an organoid simulator to objectively address all Reviewers' concerns on the sensitivity of SPOT to dataset and processing imperfections and on the extent to which 2D projection videos distinguish 3D phenotypes. Specifically, we generated two synthetic organoid video datasets (Simulated Datasets A and B) where we engineered the 3D phenotypes, then constructed and analysed the associated 2D projection videos with SAM-SPOT. These new datasets with known ground truth enabled us to address SPOT's ability to recognise and reconcile:

1) Noise and artefacts in the analysis;
2) Track fragmentation;
3) Inaccuracies in object segmentation caused by spatially overlapping organoids; and
4) SPOT's suitability for 3D time-series imaging data.

Points #1–3 are addressed by 'Simulated Dataset A', while the consequence of movement in the Z-dimension, Point #4, is addressed using 'Simulated Dataset B'. The synthetic organoids are simulated fully in 3D and then projected into 2D using the same extended focus projection principles as that used for real acquisitions. Using simulations enabled us to have ground-truth phenotypes and to apply a wide range of systematic perturbations (including those identified by the Reviewers) to test how effectively SPOT can extract 3D information from 2D projections, to assess how effectively SPOT can distinguish distinct phenotype categories with a known ground-truth, and to determine how robust SPOT analysis remains in the face of experimental, imaging, and segmentation imperfections, thereby enabling tolerance thresholds to be set.

Simulated Dataset A

We generated a synthetic dataset of simulated 3D organoids, that evolve across time from a small, round initial state into a phenotypically distinct final state (7 conditions, 45 organoids per condition, and 30 frames per organoid) (new Fig. 2b). Each of the conditions share a common starting state with identical shape, appearance, motion behaviour parameterisation, before diverging over the duration of the 30-frame time course into specifically-parameterised, condition-specific end states. To generate this dataset, we define one condition-dependent end state as the 'base organoid', with 'basal' parameter values. Then, using the base organoid parameterisation as the template, we modified parameters influencing each of shape, appearance, and motion in isolation, to create 6 further extremal 'high' or 'low' conditions for each concept. The dataset is comprised of 3-dimensional organoid simulations and their 2-dimensional projections along with pixel-perfect segmentation masks. To generate the 2-dimensional projections, we used an equivalent method to the extended depth of focus projection used on our real timelapse z-stacks. We subjected the entirety of Simulated Dataset A to 8 different perturbation types, each across 9 severity levels, to identify effects on the analysis relative to the known ground truth.

Simulated Dataset B

We generated another synthetic dataset of simulated 3D organoids, with 3 conditions that share the same shape and appearance parameters, with organoids moving with the same speed but differing in the allowed movement directionality. One condition represents unrestricted xyz movement freedom, a second condition represents a replicate of the first condition with different organoids and the third condition represents movement only in the plane parallel to the projection i.e., xy plane (in total, 3 conditions, 6 replicates per condition, 12 organoids per replicate, 30 timepoints per organoid). This dataset was designed to address the specific concerns of Reviewer 3 regarding the effect of movement in the Z-dimension on phenotype readout.

The key findings of applying SAM-SPOT to the synthetic dataset A is included as new Figure 2 in the main text. The results of the synthetic robustness tests using perturbations of dataset A, and analysis of dataset B are included as additional Suppl. Fig. 2a and 2b.

EXTENDED POINT-BY-POINT RESPONSES TO EACH REVIEWER'S COMMENTS:

Reviewer #1 (Remarks to the Author):

This manuscript introduced a pipeline to track the phenotypic changes of objects in 2D videos, and applied it on living-cell videos. Together with single cell sequencing data, this tool may study the phenotype-genotype association. I have the following comments:

1) It is hard to call any chosen summary features as comprehensive and unbiased phenotypic description of the video, as long as you do not use all the pixel information.

We apologise for not defining the terms "comprehensive" and "unbiased" clearly in the text. We use the terms "comprehensive" and "unbiased" to refer to the way in which the image features are acquired and how the acquired data are analysed, respectively. Specifically, SAM features are "comprehensive" because they have been designed to be a generally applicable set of image features covering all spatial scales of global, regional, and local (i.e. distributional) (akin to late and early layers of a neural network) and the different attributes of shape, appearance and motion. We note local distribution features are derived by using all the pixel information. The specific composition of features is now illustrated in Fig. 2 of our new companion paper, which we include below for reference.

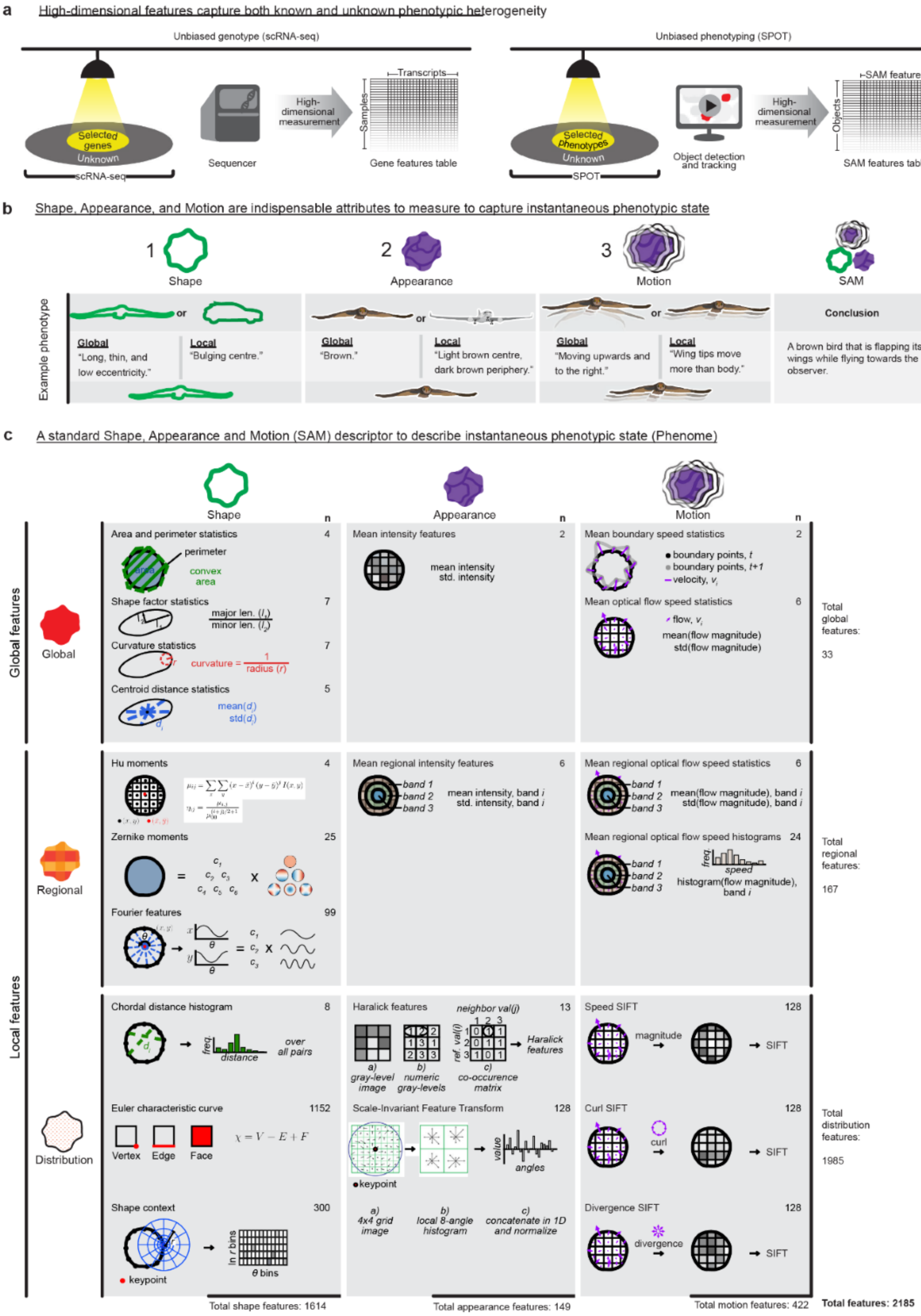
a
High-dimensional features capture both known and unknown phenotypic heterogeneity
Unbiased genotype (scRNA-seq)
Selected genes
Unknown
scRNA-seq
Sequencer
High-dimensional measurement
Transcripts
Samples
Gene features table
Unbiased phenotyping (SPOT)
Selected phenotypes
Unknown
SPOT
Object detection and tracking
High-dimensional measurement
SAM features
Objects
SAM features table
b
Shape, Appearance, and Motion are indispensable attributes to measure to capture instantaneous phenotypic state
1 Shape
2 Appearance
3 Motion
SAM
Example phenotype
or
Global
"Long, thin, and low eccentricity."
Local
"Bulging centre."
Global
"Brown."
Local
"Light brown centre, dark brown periphery."
Global
"Moving upwards and to the right."
Local
"Wing tips move more than body."
Conclusion
A brown bird that is flapping its wings while flying towards the observer.
c
A standard Shape, Appearance and Motion (SAM) descriptor to describe instantaneous phenotypic state (Phenome)
Shape
Appearance
Motion
n
Global features
Global
Area and perimeter statistics 4
perimeter
area
convex area
Shape factor statistics 7
major len. (l_1) / minor len. (l_2)
Curvature statistics 7
curvature = 1 / radius (r)
Centroid distance statistics 5
mean(d_i)
std(d_i)
Mean intensity features 2
mean intensity
std. intensity
Mean boundary speed statistics 2
boundary points, t
boundary points, t+1
velocity, v_i
Mean optical flow speed statistics 6
flow, v_i
mean(flow magnitude)
std(flow magnitude)
Total global features: 33
Local features
Regional
Hu moments 4
$\mu_{ij} = \sum_x \sum_y (x-\bar{x})^i (y-\bar{y})^j I(x,y)$
$\eta_{ij} = \frac{\mu_{ij}}{\mu_{00}^{(i+j)/2+1}}$
Zernike moments 25
c_1 c_2 c_3 c_4 c_5 c_6
Fourier features 99
c_1 c_2 c_3
Mean regional intensity features 6
band 1
band 2
band 3
mean intensity, band i
std. intensity, band i
Mean regional optical flow speed statistics 6
band 1
band 2
band 3
mean(flow magnitude), band i
std(flow magnitude), band i
Mean regional optical flow speed histograms 24
freq
speed
histogram(flow magnitude), band i
Total regional features: 167
Distribution
Chordal distance histogram 8
freq.
distance
over all pairs
Euler characteristic curve 1152
Vertex
Edge
Face
$\chi = V - E + F$
Shape context 300
keypoint
ln r bins
θ bins
Haralick features 13
neighbor val(j)
ref. val(i)
Haralick features
a) gray-level image
b) numeric gray-levels
c) co-occurence matrix
Scale-Invariant Feature Transform 128
keypoint
value
angles
a) 4x4 grid image
b) local 8-angle histogram
c) concatenate in 1D and normalize
Speed SIFT 128
magnitude
SIFT
Curl SIFT 128
curl
SIFT
Divergence SIFT 128
divergence
SIFT
Total distribution features: 1985
Total shape features: 1614
Total appearance features: 149
Total motion features: 422
Total features: 2185

We use the term *unbiased* to emphasise that all features in the SAM phenome can be and are computed for all datasets in the same manner and are not 'handcrafted' to target a particular phenotype *a priori* (e.g. organoid branching-specific) nor require adaptation to be computed for any imaging modality or imaged object (e.g. number of branches is a feature specific only to objects that branch). This is in contrast to data-driven learning such as convolution neural network (CNN) features trained on a particular dataset, which is by definition a "*biased"* approach in that it necessarily requires training on the target dataset to guarantee relevance of generated features.

Our new companion methods-focussed paper entitled *"Shape, Appearance, and Motion (SAM) features enable construction of the phenome and development of SAM Observation Tool (SPOT)"* describes in detail the rationale used for constructing the SAM phenome.

"*Specifically, to ensure a maximally applicable SAM phenome for real data, we established our SAM phenome upon four design principles (Fig. 1c). First, to be comprehensive, SAM properties are measured at multiple spatial scales; global (whole object), local-regional (parts of whole object) and local-(pixel-level) distribution. Second, at each scale, a broad set of SAM features is measured to capture all potential SAM variations, not limited to only identifying object phenotypes within a particular analyzed dataset. This includes measuring redundant features, which characterize the same property but are each computed differently. Redundancy builds robustness to inevitable inaccuracies in segmentation and tracking, whilst still providing additional information. For example, area is significantly more sensitive when an object is partially segmented than the convex area. However, using convex area may underestimate the difference in actual area between two genuinely concave shapes, e.g. a cell with protrusions or that between a convex and concave shape. Third, features are computed to be invariant to object translation or rotation. Evidently, the object is the same under these transformations and therefore should have the same SAM phenome. Reflections of an object are treated as being a different object and therefore have different SAM phenomes. These might reflect differences in the underlying biological process or systematic difference in acquisition (e.g. camera flipped). Last, to enable application to videos of any duration and acquisition frequency, we consider only first order motion features; those computable using a pair of consecutive frames. Higher order motion properties such as acceleration are implicitly captured in tracking over multiple frames.*

Briefly, for Shape, we compiled 1614 features, including 23 global (area and perimeter, shape factor, curvature and centroid distance statistics), 131 regional (Fourier features[62,63], Hu[64] and Zernike[62,65] moments) and 1460 distribution features (chordal distance histogram[36], Euler characteristic curve[66,67], shape context[68]). For Appearance, we compiled 149 features, including 2 global (mean intensity), 6 regional (regional intensity) and 141 distribution features

(Haralick[69], Scale-Invariant Feature Transform (SIFT)[70,71]). For Motion, we generated 442 features, including 8 global (mean boundary speed, mean optical flow speed statistics), 30 regional (mean regional optical flow speed statistics and histograms) and 384 distribution features (Speed, Curl and Div SIFT). The concatenation of all Shape, Appearance and Motion features (1614+149+442=2185) form our final 2185-D SAM phenome (Fig. 1c and Suppl. Table 1)."

Finally, we agree with this reviewer that a higher number of features may be more comprehensive, but too many features make the phenome 'too unique' leading to poorer performance without further sophisticated preprocessing. Our reasoning is detailed in the discussion of our new methods focussed manuscript, supported by empirical benchmarking results on computer vision datasets:

"Rational construction of the SAM phenome was critical to this success (Fig. 1, Methods). A sufficient number of image features is necessary to be 1) comprehensive; characterizing the properties of shape, appearance, and motion at multiple spatial scales (global, regional and pixelwise (i.e. distribution)); and 2) robust to artefacts from image acquisition, segmentation and tracking; achieved by computing redundant features with complementary information e.g., computing both area and convex area. However, excessive features make each SAM phenome too 'unique', and hamper pattern finding. As such, our final full 2185-feature phenome represents a good balance. This principle can be best seen in our benchmarking of different subgroupings of SAM-S features on MPEG-7 shapes (Suppl. Table 2). The full SAM-S (kernel ECC) phenome (ranked 1st, Suppl Table 2) outperformed all subgroupings, but only due to preprocessing of ECC features (n=1152, 11th) to reduce its dimensionality to 100 features before concatenation. The raw SAM-S phenome (n=1614) significantly underperforms, ranking 6th, thus lower than its constituent subgroupings: Shape context (3rd, n=300), Zernike moment (4th, n=25) and geometrical (5th, n=17). It might also be supposed that an imbalance in the number of shape, appearance and motion-associated features in our SAM phenome may bias analysis. However, we do not find evidence of this empirically. The contribution scores computed by SPOT in all studied datasets reflect the expected relative importance of shape, appearance and motion. Notably, SAM-SPOT analysis identified specific shape, appearance and motion-associated modules in a simulated organoid dataset relating to the predefined shape-, appearance- and motion-specific phenotypes (NC-SPOT-MS1)."

The relevant results table assessing the use of subsets of the SAM-S phenome is included below:

	k-means clustering to identify MPEG-7 shape categories (k=70)					
	**Feature Descriptor**	**AMI**	**ARI**	**1/2*(AMI+ARI)**	**Average Ranking**	**Feature Scope**
Subset features of SAM-A	Shape context, n=300	0.652	0.435	0.544	3	Distribution
	Zernike moment, n=25	0.638	0.413	0.526	4	Regional
	Geometrical, n=17	0.587	0.378	0.483	5	Global
	chordal distance histograms, n=8	0.555	0.327	0.441	7	Distribution
	kernel Euler Characteristic Curve (kernel ECC), n=100 **	0.528	0.320	0.424	8	Distribution
	centroid statistics, n=5	0.510	0.296	0.403	9	Global
	Hu moments, n=7	0.469	0.200	0.335	10	Regional
	Euler Characteristic Curve (ECC), n=1152	0.506	0.133	0.320	11	Distribution
	Fourier features, n=99	0.275	0.114	0.195	12	Regional
ll SAM Appearance descriptor (SAM	SAM-S (ECC), n=1614	0.634	0.327	0.481	6	Combination
	**SAM-S (kernel ECC), n=562**	**0.710**	**0.518**	**0.614**	**1**	Combination
Cellprofiler Shape features	Cellprofiler shape features, n=98	0.684	0.466	0.575	2	Combination
Negative control	Random label permutation	0.000	0.000	0.000	13	n/a
	**** : kernel ECC is a dimensionality reduction of ECC features, based on comparing the similarity of ECC over multiple objects and therefore is not a direct measure but only computable over a given video dataset**					
**AMI** - adjusted mutual information score (upper-bounded by 1) the larger the better.	https://scikit-learn.org/stable/modules/generated/sklearn.metrics.adjusted_mutual_info_score.html					
**ARI** - adjusted rand index score (-0.5 to 1.0). -0.5 = especially discordant, 0.0 = random labelings, 1.0 = perfect match	https://scikit-learn.org/stable/modules/generated/sklearn.metrics.adjusted_rand_score.html#sklearn.metrics.adjusted_rand_score					

2) For example, your feature does not cover the acceleration of segmented objects even if you only work on 2D objects from single color 2D images

Reply:

As acceleration is the time derivative of velocity, we would expect a change in acceleration to manifest in the next time frame as a change in velocity. Therefore, SPOT does implicitly capture the acceleration of segmented objects. Generally, we expect higher-order motion statistics to manifest as increasing temporal delays, requiring longer tracking to capture. One frame in a video can represent any temporal unit from 1 ms to 1h to 1 day, depending on the video in question. Equally, the total number of frames can vary. Therefore, to ensure maximum applicability, we designed the SPOT analysis method to only include motion measures that can be computed from 1 successive frame. We have explicitly clarified this in our new companion methods-focussed paper, with the relevant text cited in the above point. Incidentally, this is why we refer to our construction as the instantaneous 'SAM phenome' and can use this phenome to map all temporal object instances as individual points in a 2D landscape using UMAP. We note that long-term dynamics are not lost however and are modelled back through the single object phenotype trajectories and phenotype cluster transitions, best demonstrated by our analysis of cell division in Fig. 7 of our methods-focussed companion paper.

As seen in the selected panels b) and e) below, the SAM phenome derived phenomic landscape maps all seven cell division stages, and the SAM phenotype trajectory constructed using the landscape successfully infers a cyclic path recovering the temporal order of stages.

Notably each turning point in the trajectory denote a significant change in SAM characteristics between stages.

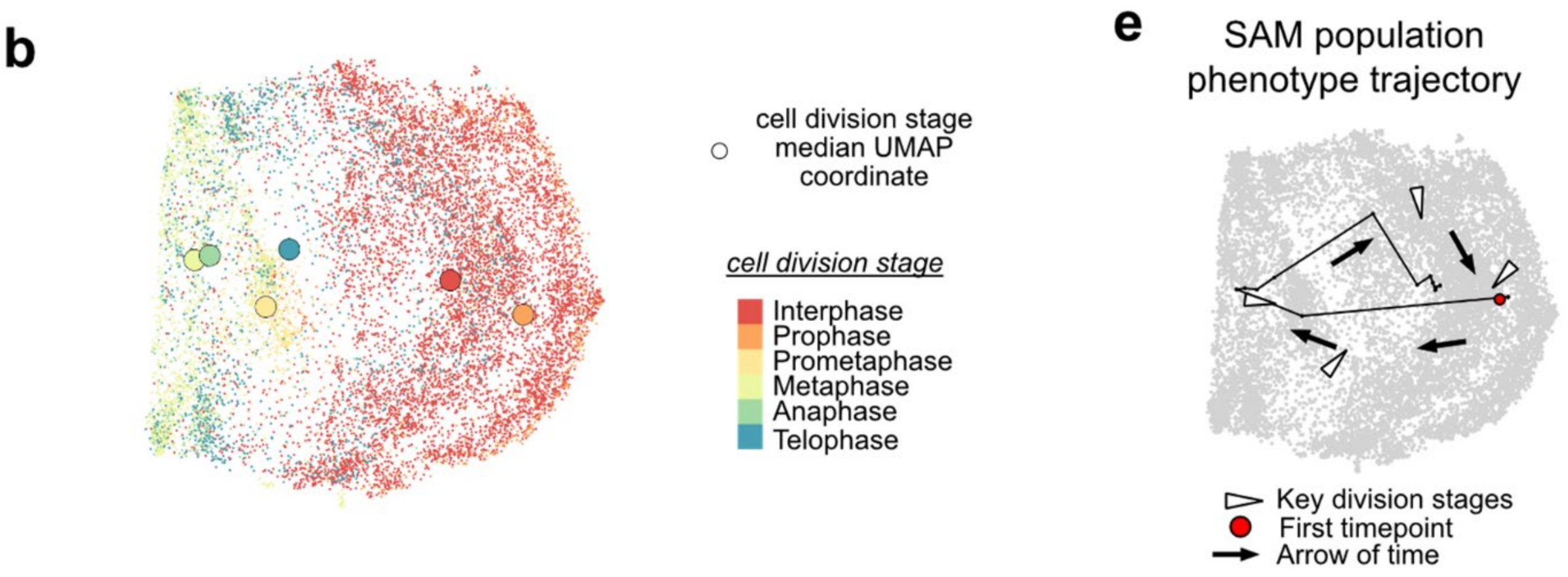

3) line 169-170: how did you select the tuning parameters 200 and 3 here? Is it possible to automatically select by certain version of cross-validation?

Reply:

We want to store the segmented organoids in a more efficient manner than storing each binary pixel in individual cropped segmentation masks. However, individual boundaries have different numbers of points and would not allow the stacking of multiple boundaries into a regular array, resulting in larger and irregular storage on disk. We therefore chose to sample the contour of each organoid with 200 equidistant points, as comparable numbers have been used in the literature to represent complex cell morphologies e.g. CAJAL ([Govek et al., *Nat. Commun.,* 2021]) which used 100 points to classify diverse neuron morphologies. To demonstrate that 200 points does still retain good shape approximation empirically, we assessed the symmetric Hausdorff distance of MPEG-7 shapes with increasing number of sampled equidistant contour points. The MPEG-7 dataset, (https://dabi.temple.edu/external/shape/MPEG7/dataset.html) captures diverse shape variations in 70 different everyday objects. We find rapidly diminishing returns in capturing the shape complexity above 100 equidistance points.

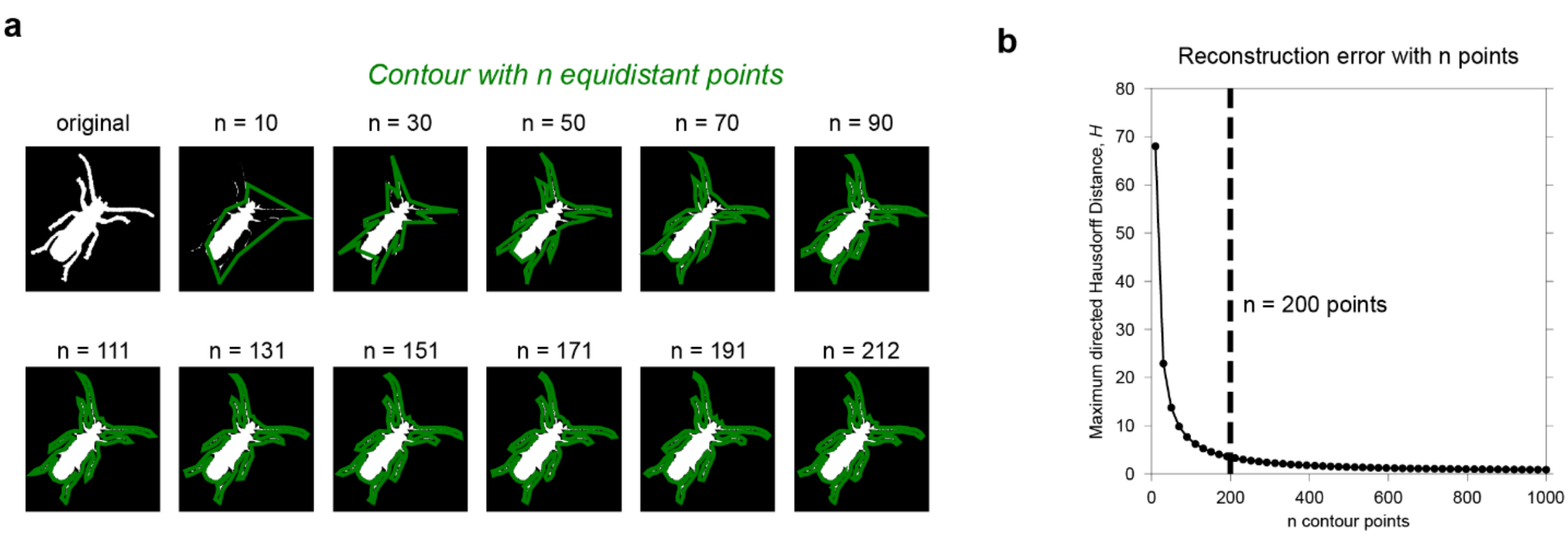

We partitioned the object area into 3 regions of equal distance from its boundary, as this has natural interpretation of the border, middle and central regions of an object. Unfortunately, in order for the number of total features to be identical across for all objects, the number of

partitions also needs to be applicable and computable for all objects, irrespective of their area. As such, there is little scope for optimization. Whilst further subdivision is possible, for small objects, these regions would result in smaller (and possibly zero) image area, yielding more intrinsically noisy or uninformative features. Thus we decided to maintain three regions. We also did not consider further division by angular rotation which for concave shapes or shapes of high curvature may under- or over-sample the partitioned area in particular angles. Furthermore, we would require accurate rotational alignment to ensure the derived measurements are rotation-invariant – the same object but rotated is the same object and should have the same phenome.

4) The success of the whole pipeline is dependent on accurate object tracking/tracklet. Can you quantify the accuracy of this step of your pipeline and discuss how this accuracy will affect the downstream results numerically?

a) Accurate object tracking and quantify the accuracy of the tracking pipeline:

Reply:

The tracking approach we implement is 'first detect then link'. Organoids are first detected as a bounding box on a per frame basis, then matched by solving the linear assignment problem using intersection-over-union (IoU) as the cost function. Using IoU intrinsically accounts simultaneously for size and closest distance. This linking approach is the most popular, state-of-the-art and optimally solves bipartite matching problems such as the pairing of detected organoids across two frames, e.g. also used by Fiji TrackMate software. We further extended this strategy by computing the rigid transform of the organoid between the current and next frame from the independently computed pixel optical flow. This predictive step enables us to further handle large morphological changes, and can be seen as the image-based extension of a Kalman Filter. We observed the matching between two frames to be 100% accurate. Our tracking approach is primarily used to enable us to determine whether the per frame based organoids detected are 'real' and to filter out transient objects such as floating debris. We assume that real organoids are detectable for at least 5 frames. Therefore our "detect and link" approach provides the required object tracking accuracy and specificity. Given good organoid detection in individual frames, we will have accurate pairwise matching. Then, only good individual frame organoid segmentation is needed for SAM phenome computation. To ensure sufficient detection and segmentation we trained neural networks whose performance has been additionally reported in the methods:

"We trained a YOLOv3 object detector for bounding box organoid detection with a total of 43,199 organoids extracted from 1534 images comprising label-free and fluorescent confocal microscopy. Images contained separated and cluttered overlapping organoids ranging from a few organoids per image to roughly 100. We measure a mean average precision with an intersection-over-union (IoU) cutoff of 0.25, $mAP_{25} = 0.91$ *on a test dataset of 43,145 organoids extracted from 1437 images comprising label-free and fluorescent confocal microscopy. Images contained separated and cluttered overlapping organoids ranging from a few organoids per image to roughly 100. If IoU cutoff is set to 0.50, we measure* $mAP_{25} = 0.67$.

We trained a simple attention-UNet model of 4 layers to segment individual organoid from image crops containing one organoid in isolation and spatially overlapping neighbors. The model has 8 features in layer 1 with the number of features doubling in depth as the image size is average pooled by a factor of 2. The model is trained on 542 unique cropped organoids

from fluorescent confocal and label-free images resized to 64 x 64 pixels and random image augmentation. We obtain a test accuracy measured by IoU of 0.87 for a total 555 test images."

For SPOT analysis, longer-time tracking may benefit HMM model fitting, but is not critical to the Hidden Markov Model (HMM) based cluster transition analysis, as we demonstrate with our simulated organoid dataset (Suppl. Fig. 2c iv, bottom right). The mean elementwise difference of the HMM transition matrices remain low relative to the ground truth (100% of frames) as the level of track fragmentation increases (coverage fixed at 50% of frames), showing only a slight positive trend. This indicates that whether the tracks are highly fragmented (short) or not (long), disagreement with the ground truth HMM cluster transitions is consistently low. All other analyses are minimally affected (Suppl. Fig. 2c ii,iii, bottom).

b) how this accuracy will affect the downstream results numerically?

Reply:
A unique feature and advantage of SPOT is its ability to analyse downstream data that are not 100% accurately tracked. Firstly, 420 of the motion features are derived statistics from the per pixel velocity vectors computed by two-frame optical flow. Optical flow uses the video pixel intensity values directly and therefore unaffected by organoid tracking. The optical flow technique used, Farneback, has been previously demonstrated in the first author's PhD thesis to work robustly for organoids, even being utilized to segment organoids and track organoid branching (Zhou, F. (2017). Phenotyping cellular motion [PhD thesis]. University of Oxford). Likewise, the Hidden Markov Model (HMM) model depends only on consecutive frames and estimates model parameters from many organoid instances. Secondly, the pairing of organoids between successive frames is one-to-one. Only one organoid can be paired to another. Even if there is an identity switch when tracking an organoid, the switch only affects that one timepoint of tracking. SPOT additionally implements an IoU consistency check between consecutive times of a tracked organoid whereby a track is split, if the bounding box of the organoid in the next frame differs too much in overlap, to prevent obvious switching errors. In particular, SPOT will split the track into two: one before the switch and one after the switch. For example, suppose we have a video of 20 frames where cultures are very dense, and during timepoint 0–10, we track organoid 1. Then, in frame 11, there is overlap with organoid 2 such that the track of organoid 1 switches to tracking 2, and the track of organoid 2 now tracks 1. Considering the whole track, the tracking of organoid 1 and 2 is 50% accurate, however the matching of organoids from frame-to-frame is high - only the preceding frame when identities switch is incorrect. Therefore inaccurate tracking has minimal effect on SPOT. As each instance of an organoid is an independent observation in the SPOT analysis, there is only one erroneously matched timepoint for organoid 1 and 2 at timepoint 10. All other timepoints are correctly matched. Importantly, during the revision, the accuracy, effectiveness and robustness of SPOT was objectively validated using our newly created 3D organoid simulation movies, with systematic perturbations to segmentation and tracking (new Figure 2 and related Supplementary Figures).

5) The 3D organoid imaging produces z-stack 3D images. Is it too rough to simply project the z-stack to one 2D image as in line 1476? You may want to as least discuss on how to better treatment 3D images.

Reply:

We thank the reviewer for this comment and acknowledge that our current approach to project z-stack to one 2D image would be suboptimal, had we captured isotropic resolution images of organoids grown in 3D so that images contain unique 3D SAM information. However, as seen in Suppl. Fig. 1a and 1b, both our label-free and confocal microscopy z-stacks are resolution-limited. The 3D stack offers effectively only 2D information. Obtaining more z-slices with confocal microscopy might slightly improve 3D resolution but would severely affect photobleaching, drastically limiting acquisition frequency and total duration as well as potentially affecting native biological behaviour. Lightsheet microscope setups may achieve near-isotropic 3D imaging, but is currently insufficiently high-throughput and high-content for our organoid experiments. It also suffers the same issues of photobleaching. Moreover, due to scattering, it cannot capture the full organoid depth without the need to design custom, and complex multi-camera and sample holder setups, beyond the capability of our lab and the scope of this manuscript.

Instead, to assess the extent of mutual information between full 3D images and the equivalent 2D projections used by SPOT, we simulated full 3D organoid movies with defined phenotypes and analysed the 2D extended focus projection organoid scenes with SPOT. Our findings in the new Suppl. Figure 2 showed from 2D projection videos only, SAM-SPOT can still effectively discriminate simulated organoids whose movement are restricted to the 2D xy-plane from those with unrestricted 3D xyz movement, even with a replicate dataset of unrestricted 3D xyz movement as confounder.

6) Your hidden Markov model description is confusing. According to line 1642-1643, both the observed data and the hidden states are phenotype cluster labels. You should differ them.

Reply:
We apologise to the reviewer for the confusion. The Hidden Markov Model is used here to temporally smooth and compute the transition between phenotype cluster transition labels, as in Held et al We have already employed cluster number selection for identifying the phenotype clusters earlier in the workflow. We set the number of emission states to be equal to the number of hidden states. ***7) Also, the entropy of the emission distribution may be related to the differentiability of your phenotype clusters, thus worthy of discussion.***

We thank the reviewer for this suggestion. We have computed the entropy of the transition matrix for each state as suggested for additional experiments: validation experiments with chemotherapy drugs on organoids (Suppl. Fig 3a). Entropy quantifies the uncertainty of a cluster transitioning. Mathematically, a cluster has maximum entropy when it has equal probability of transitioning to any other cluster. We observe that high entropy clusters provide a fingerprint of the chemotherapy drug. Considering a cutoff of 0.5 nats, cluster 3 was highly entropic in the milder DMSO and Cisplatin drugs, cluster 1 for the more toxic 5FU and clusters 1,2,4, and 5 for the most toxic Docetaxel (Suppl. Fig. 3a). Importantly cluster 4 is the most toxic phenotype cluster with highest Caspase and Cytotox marker expression. Thus it seems high entropy might help in combination with mean SAM feature expression within individual phenotype clusters to identify the most informative clusters, namely those involved

with the greatest number of cluster transitions, to help distinguish biologically relevant clusters versus 'noise' clusters.

8) Is this work related to other shape and motion work/patent of the first author? If yes, you shall explain the connection.

The other shape and motion work/patent of the first author is primarily captured in 5 publications:

1) Multiplexed 3D Analysis of Cell Plasticity and Immune Niches in Melanoma (*BioArxiv, 2024*)
   - Performs 3D segmentation of single cells in multiplexed tissue to help experimental researchers to identify immune interactions in patient tissue biopsies.
2) A general algorithm for consensus 3D cell segmentation from 2D segmented stacks *(BioArxiv, 2024)*
   - Describes a framework for shape representation to allow combining 2D segmented stacks from orthogonal views into a single 3D consensus segmentation
3) Surface-guided computing to analyze subcellular morphology and membrane-associated signals in 3D *(BioArxiv, 2023)*
   - Primarily concerns the unwrapping of surfaces of 3D single cells and monitoring of dynamic cellular protrusions over time.
4) Motion sensing superpixels (MOSES) is a systematic computational framework to quantify and discover cellular motion phenotypes (*eLife*, 2018)
   - Concerns the extraction motion signatures from an entire video without explicitly segmenting individual objects
5) Single-cell phenomics reveals behavioural and mechanical heterogeneities underpinning collective migration during mouse anterior patterning (*BioArxiv*, 2023)
   - Application of unwrapping and MOSES to identify distinct phenotypic behaviour of a subset of migrating cells during 3D embryogenesis.
   - This work extracts targeted shape features for analysis and analysis is guided by knowledge of the embryo geometry.

We regard these works as independent and tangential to the current work, which focuses on developing a general feature set and temporal analyses applicable for analysing all individually segmented 2D object dynamics.

9) for the phenotype-genotype coupling, since it is currently impossible to get both the phenotype and genotype of the same cell at the single cell level, what potential problem does the population-level coupling have? How will poor coupling/mis-alignment affect the study?

We completely agree with this reviewer that it is impossible to obtain the genotype-phenotype coupling at a single cell level. Although cells and organoids are highly heterogenous, similar morpho-dynamic phenotypes can be reflective of cells or organoids who share similar genotypes and with similar biological functions. The premise we take in the manuscript is that it is difficult to infer consensus patterns when considering only individual

cell or organoid heterogeneity, whilst a simple average may not reveal sufficient differences. SPOT's proposal is to capture a middle ground of monitoring temporal changes i.e. the *delta* in the distribution of heterogeneity relative to the baseline at timepoint 0, over a longer time horizon than the biological process being studied. Using our stacked barplots, we can then begin to make functional inferences more reflective of the mechanistic action of a particular stimulus. Of course, any insights would then need to be supplemented with more targeted molecular assays such as that in Fig. 6. The primary motivation, however, is that SPOT could represent a relatively inexpensive way to identify those functional timepoints and phenotypes that maximally increase the likelihood of identifying mechanistic differences. These points have now been included in the discussion.

Reviewer #1 (Remarks on code availability):

The author provided the code and README, but the code is too complicated for me to run.

We apologize and thank the reviewer for the feedback. We have created step-by-step instructions for running the Python scripts with the provided example datasets. This is included as part of the GitHub repository as a .docx word file. We further make available the new organoid dataset with chemotherapy drugs and cytotox/caspase stains used in the revision, with its corresponding analysis code for further reference. The code will be continuously maintained in GitHub. Through GitHub issues, we welcome community suggestion and reporting of bugs.

REVIEWER #2 (REMARKS TO THE AUTHOR):

This scientific paper introduces a concept in the field of imaging analysis, termed the "imaging phenome," which is analogous to the transcriptome in single-cell transcriptomics. The authors propose a comprehensive framework to describe phenotypic diversity in dynamic objects through a standardized set of image descriptors. These descriptors are categorized under three major properties: Shape, Appearance, and Motion (SAM), each of which is considered as an individual imaging "transcript." To materialize this concept, the paper details the establishment of a standardized SAM "phenome," comprising 2185 distinct SAM features. This extensive collection of features provides a robust and comprehensive method to characterize various dynamic objects in imaging studies. Alongside this, the authors have developed a sophisticated analytical tool called SPOT (SAM Phenotype Observation Tool). SPOT is described as an imaging "sequencer," capable of analyzing both label-free and fluorescent images.

The paper underscores the potential of this approach in enhancing the understanding of phenotypic diversity through detailed and standardized imaging analysis, akin to the insights provided by transcriptomic analysis in genetics.

I've read the paper with great interest and the paper is well written and the strategy is well explained. However, I cannot advice for publication of the manuscript in its current form and the scientific work needs several improvements. Nonetheless, I think it is an interesting strategy, although applying scRNA analysis tools to single organoid image analysis is not novel (https://doi.org/10.1038/s41698-023-00480-y). But I think the approach has a lot of potential, if applied correctly.

Reply:

We thank the reviewer for recognising the value of our approach and its potential to make an important impact to the field.

1) The manuscript is way too long and lacks focus. For me, these are at least two or three independent papers. (i) Development of the SAM tool and algorithms and testing it on the public databases. (ii) applying SAM to 2D cell culture (iii) applying SAM to 3D organoid culture. For readers, it is very difficult to extract the information that is relevant for them, and these three topics mostly have a different target audience.

2) I wonder how relevant the use of the public databases from youtube and MPEG-7 is. How relevant are these features in cell biology and using this to compare with CellProfiler seems strange.

3) Would a repository such as LIVECell not be more relevant to determine is SAM can accurately cluster distinct cellular morphologies rather than irrelevant images and videos (https://www.nature.com/articles/s41592-021-01249-6)?

Reply:

We thank the Reviewer for these constructive suggestions. Our motivation for choosing computer vision datasets was to use relatable everyday examples to demonstrate the need to design multiscale spatial features that capture shape, appearance, and motion simultaneously. We also wanted to develop a single feature set or 'phenome' that is applicable to videos of any 2D object which would enable like-for-like comparison between any analysed datasets across assays, conditions and labs.

To increase manuscript focus of organoids and to enable us to present key experiments performed on published 2D datasets leading to the development of our SAM phenome and SPOT, we have developed the original submitted manuscripts into two independent but complementary manuscripts: the current revised manuscript, focussing on the application of SAM phenome and SPOT analysis to analyse 3D organoids, and a new parallel manuscript submission entitled *"Development of a universal imaging "phenome" using Shape, Appearance and Motion (SAM) features and the SAM Observation Tool (SPOT)"* focussed on the details of the method development. By moving the detailed description of the SAM-phenome construction and SPOT development into the methods-focussed paper, the revised paper can focus exclusively on establishing the applicability of SAM-SPOT to identify genotype-phenotype-function coupling in high-content 3D organoid cultures using 2D projection videos (new Figure 1), as suggested by this Reviewer. We recognise the value this reviewer places on testing whether ground truth morphologies can be accurately distinguished using a relevant reference dataset. To respond to this, we created a novel organoid simulator to generate synthetic 3-dimesional organoid movies with user-prescribed evolution of their shape, appearance, and motion over time. We used the simulator to generate large datasets of 2D projection videos with complex ground truth SAM phenotypes. Our results demonstrate the effectiveness of SPOT analysis in a highly relevant, organoid-specific context (new Fig. 2 and new Suppl. Fig. 2).

Our new methods manuscript, which we invite the Reviewers to request access via the Editor, contains the non-organoid information with substantial expansion of the details in constructing our SAM phenome and the development of SPOT analysis framework. This paper also provides detailed validation of SAM-SPOT on computer vision and YouTube video datasets with ground truth. Additionally, following this Reviewer's valuable suggestion, we assessed the applicability of SAM-phenome and SPOT to large biological public datasets with known phenotype information, LIVECell for static images and cell division for live-cell videos as suggested by reviewers. We further compared results to using convolutional neural network (CNN)-features and CNN-LSTM (long short term memory)-features after training these AI-based models on the datasets, using 1 million fixed single cell images and >5000 single cell video frames for training, respectively. We then tested the features in the respective datasets when used instead of our SAM phenome in training phenotype classifiers and for SPOT analysis using >400,000 fixed single cell images and >7000 single cell video frames, respectively. Our SAM-phenome significantly outperformed both CNN-features or CNN-LSTM-features in both tasks, training superior phenotype classifiers to predict cell type or cell division stage, and providing a more informative and interpretable SPOT analysis, thanks to its rationally defined features and explicit categorization of those features by shape,

appearance and motion. In summary, both manuscripts comprehensively demonstrate the power, utility and compatibility of SAM-SPOT for genotype-phenotype coupling for high-content imaging-based screening.

4) My main concern is the images that are used for the organoids. SAM quantifies 2185 features, so it is very sensitive to imaging artifacts.

Reply:

Due to the limitation in space in our previous manuscript, we did not demonstrate that SPOT's design is versatile to reconcile artefacts within the analysis. In our new methods-focussed companion paper, we now clarify our design rationale in the detailed description of the construction of SAM phenome. Specifically, the 2185 SAM features is the entire SAM phenome and is the resultant number of features after quantifying the parameters of shape, appearance and motion, at global, regional and local spatial scales. The high dimensionality includes intended redundancy across features, which builds robustness that guards against experimental and processing 'noise' errors.

We draw analogy in designing the SAM phenome to single cell sequencing and analysis vs traditional microarray/select biomarker assays. A single biomarker is interpretable but often is not always expressed nor necessarily specific to the desired cell type due to biological stochasticity and measurement noise. Using a panel is the gold standard to perform cell deconvolution and cell type calling and is much more robust. Employing the same philosophy, the SPOT workflow builds on the designed redundancy within SAM features to include many processing steps to further guard against artefacts and to maximize biological discovery. Systematic and multi-stage feature selection is employed on a per experimental basis and this is done by filtering to remove potentially unreliable features that exhibit either too high a statistical variation or those that are zero-valued across all segmentations (and thus which contribute no information). Since motion (differential change over time) is one of the key parameters of SAM features, and a key advantage of live-cell imaging, a feature selection based on regression with time enables us to further identify and remove features associated with systematic errors, as imaging artefacts should not drive temporal variation in the dataset. Thus, by selecting and retaining those SAM features that exhibit temporal variation, SPOT maximizes discrimination between conditions when considering the population-averaged temporal changes or stacked barplots whilst minimizing imaging artefacts. Therefore, in reality, whilst SPOT always computes 2185 SAM features initially, automated feature selection means only a fraction is adapted and used to analyse each experiment. If we instead under-compute features, the information is irrecoverably lost in analysis. If we compute many more features, individual phenomes will be 'too unique' and also worsen performance (See reply to reviewer 1 above). SPOT's preprocessing is analogous to the standard way in which only top-expressing transcripts are used to analyse single-cell experiments, where the full transcriptome still needs to be measured in the raw data. The advantage and the power of computing comprehensive and redundant SAM features from multiple spatial scales, the SAM phenome, versus only selected feature subsets is the basis of why SAM phenome significantly outperforms and is more broadly-applicable than purely data-driven AI-based features both in classification and in providing more informative

phenotypical measurements. To highlight this, we include below Fig. 6 analysing the LIVECell dataset in our companion methods-focussed paper).

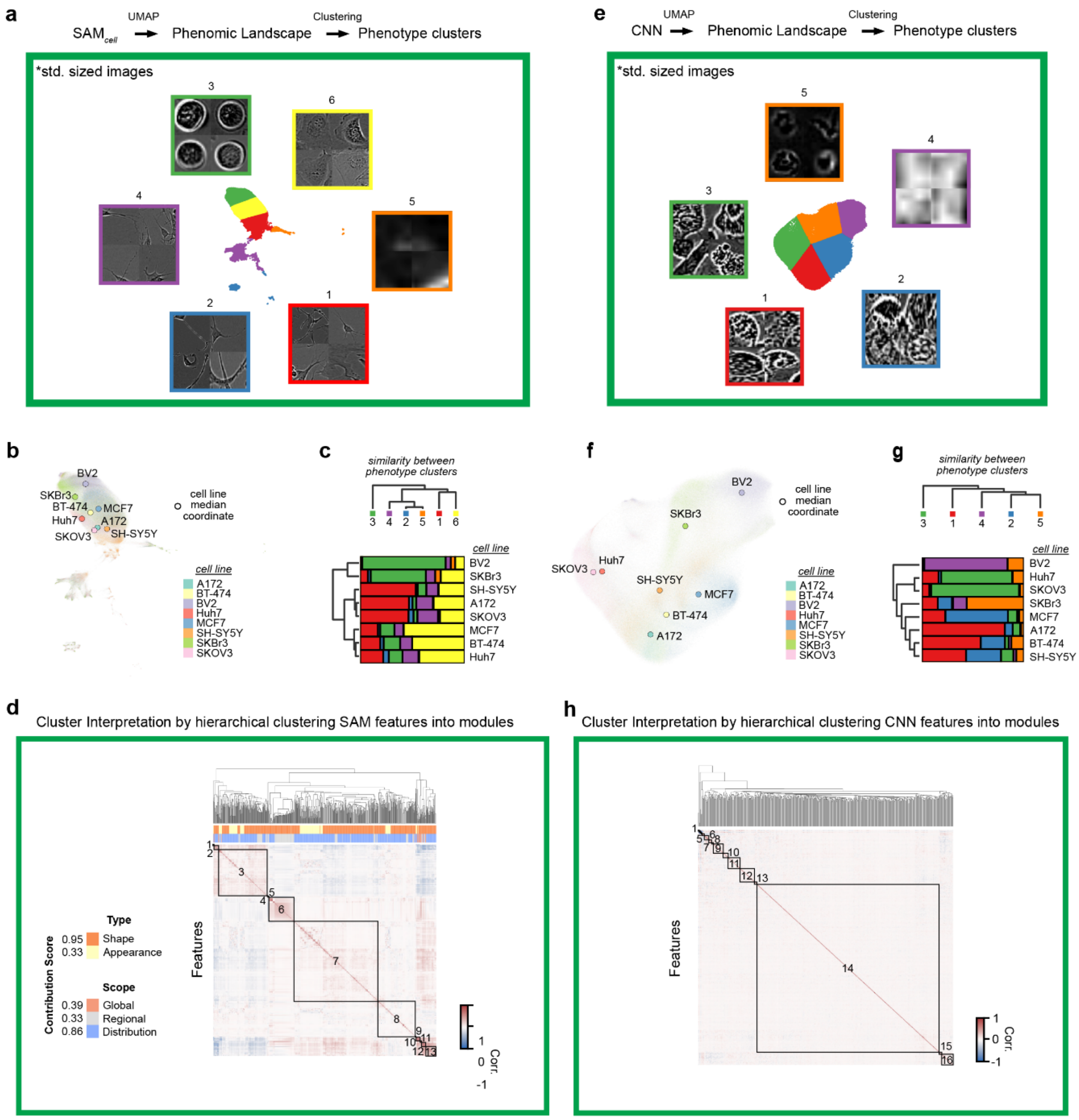

The panels in the left column show that our SAM phenome finds (a) more and unique morphotypes, which allows it to find more similarity between individual cell lines (b,c) and is more interpretable, supporting the computation of shape, appearance and motion contributions to heterogeneity and the finding of distinct feature modules. In contrast, neural network derived features are less informative, notably shown by repetitive exemplar images between phenotype clusters (e), and complete lack of intra-module similarity in individual features (h).

5) Unfortunately, the imaging and culturing strategy that was applied is not compatible with SAM.

Organoids are grown in ECM domes, therefore they are not in the same plane which is a

minimal requirement for this approach. There are various methods and plates available to image organoids in a single plane such as microwell plates (e.g. Gri3D) or flat-bottom 384-well ULA plates (https://www.jove.com/v/64434/multiparametric-tumor-organoid-drug-screening-using-widefield-live).

Reply:
We thank the reviewer for the careful reading and we agree with this reviewer that "*organoids are grown in ECM domes, therefore they are not in the same plane*". We used various culturing methods and plate types in this study according to the live cell imaging acquisition requirements, as presented now in new Suppl. Table 3. Regardless of culturing method, organoids lie in different planes. To reconcile this in our z-stacks (primarily 11 z-slices) which offer minimal 3D information (Suppl. Fig. 1a, 1b), we maximize the number of in focus organoids within the dome shaped matrigel in one 2D image by performing 2D extended focus projection, also called focus stacking for label-free microscopy and maximum intensity projection for fluorescent microscopy videos. The new Suppl. Fig. 1a illustrates how in label-free microscopy images, extended focus which projects organoids in different planes to 2D based on the strength of their image gradient is better than maximum intensity projection in generating in-focussed organoids in the resulting 2D image so that we can measure biologically relevant SAM features. We have now updated our Methods and Supplementary Materials section to ensure that all image acquisition details are included. Additionally, we also specified the tissue culture plates used in each of the experiments presented. These are summarised in the below table (new Suppl. Table 3):

Figure	Plate	Z-stack
Figure 3	Corning® Costar® TC-Treated Multiple Well Plates	Z-stack images 1000 µm in depth were acquired at 100 µm intervals (11 steps per acquisition) every hour for 5 days.
Figure 4	384-well plate, Flat bottom, rounded square wells	Z-stack images 1225 µm in depth were acquired at 35 µm intervals (35 steps per acquisition) every 2 hours for 5 days at each of the chosen positions.
Figure 5	Nunc MicroWell 96-Well Optical-Bottom Plates with Polymer Base, Cell culture, black	Z-stack images 1000 µm in depth were acquired at 100 µm intervals (11 steps per acquisition) approximately every hour for 14 days.
Supp Fig 1a	Nunc MicroWell 96-Well Optical-Bottom Plates with Polymer Base, Cell culture, black	Z-stack images 1000 µm in depth were acquired at 100 µm intervals (11 steps per acquisition) approximately every 60 min for 14 days.
Supp Fig 1b	384-well plate, Flat bottom, rounded square wells	Z-stack images 1225 µm in depth were acquired at 35 µm intervals (35 steps per acquisition) every 2 hours for 5 days at each of the chosen positions.
Supp Fig 3a.c	Nunc MicroWell 96-Well Optical-Bottom Plates with Polymer Base, Cell culture, black	Z-stack bright-field images covering 1000 µm total depth were acquired at 100 µm intervals (11 steps per acquisition) every 4 hours for 5 days.
Supp Fig 3b, Supp Fig 3c	"Plastic transparent" plates: Corning® Costar® TC-Treated Multiple Well Plates	Z-stack bright-field images covering 1000 µm total depth were acquired at 100 µm intervals (11 steps per acquisition) every 4 hours for 5 days.

	"Glass-bottom" plates: Nunc MicroWell 96-Well Optical-Bottom Plates with Polymer Base, Cell culture, black	
Supp Fig 3d	Corning® Costar® TC-Treated Multiple Well Plates	Z-stack images of 1000 µm in depth were acquired at 100 µm intervals (11 steps per acquisition) approximately every 15 minutes for 5 days.
Supp Fig 4a	Nunc MicroWell 96-Well Optical-Bottom Plates with Polymer Base, Cell culture, black	Z-stack bright-field images covering 1000 µm total depth were acquired at 100 µm intervals (11 steps per acquisition) every 4 hours for 5 days.
Supp Fig 5	Nunc MicroWell 96-Well Optical-Bottom Plates with Polymer Base, Cell culture, black	Z-stack images 1000 µm in depth were acquired at 100 µm intervals (11 steps per acquisition) approximately every 60 min for 14 days.

6) From image 3B it is very evident that the clusters are mainly based on imaging artefacts. For example, cluster 3 are out of focus organoids, cluster 2 are overexposed organoids, cluster 7 are underexposed organoid, cluster 4 out of focus and underexposed… Although there might be some phenotypic differences, features extracted from these images are completely unreliable due to the imaging artefacts.

We thank the Reviewer for this comment and apologize for not being sufficiently clear in our description and rationale of 'phenotype cluster'. The term 'phenotype clusters' does not strictly refer to biologically meaningful phenotypes. A detailed description of the rationale for each step of the the analytical workflow of SPOT is now included in Fig. 2 of our methods-focussed paper, included below.

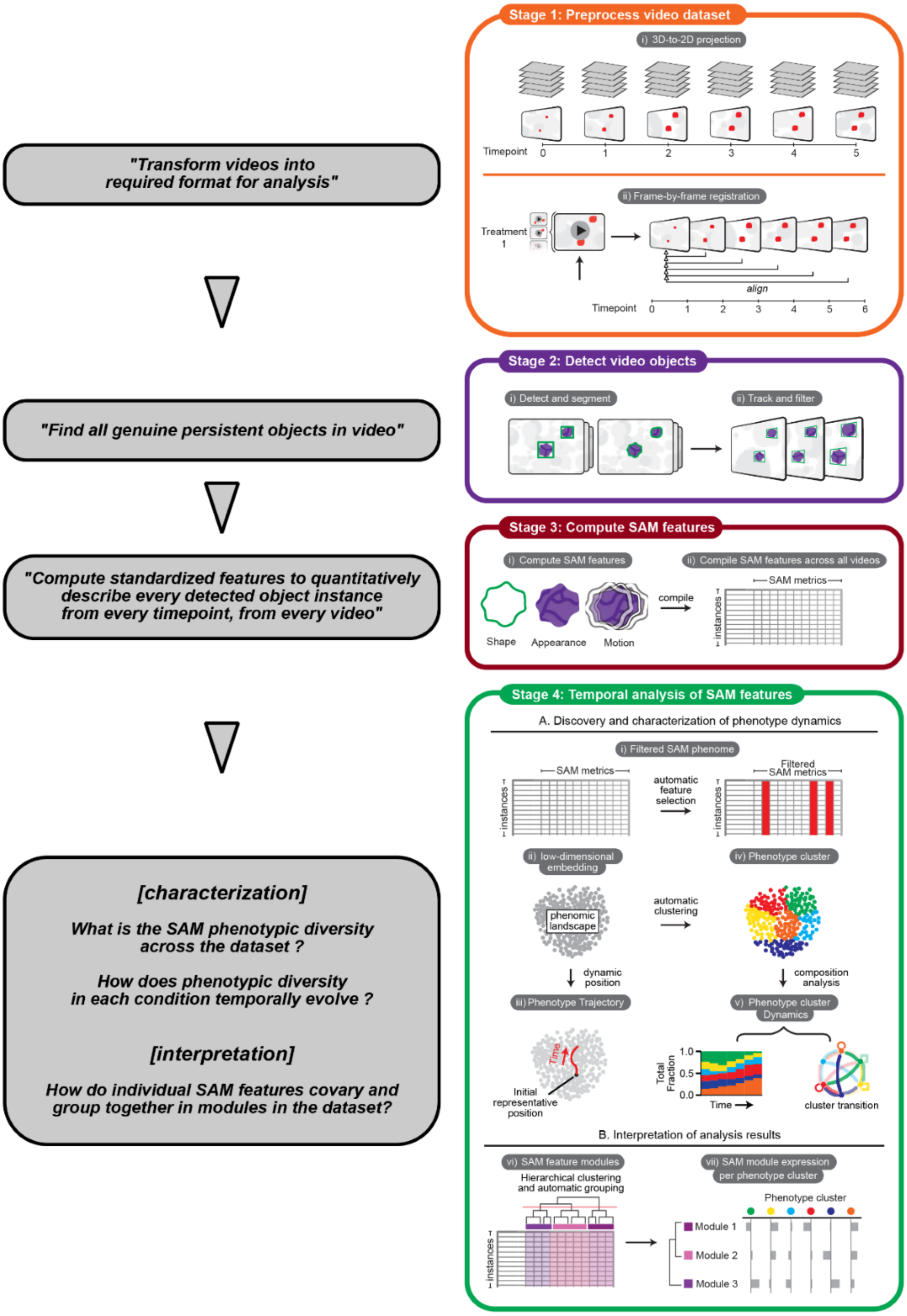

Technically, the phenotype clusters coarsely partition the UMAP phenotypic landscape such that organoid instances within each cluster share more similar instantaneous SAM features. This does not necessarily mean each cluster captures a unique biological phenotype, nor does this exclude multiple clusters describing the same biological process e.g. cell death (Fig. 3). This also means that some clusters may be attributed to a dominant image artefact e.g. blur. The phenomic landscape is a map of SAM heterogeneity across all time and conditions in a single 2D coordinate space. Whilst phenotype clusters serve as the discrete representation of

SAM heterogeneity. The primary biological analysis of SPOT is to assess how phenotype heterogeneity changes temporally, as captured by the phenotype trajectory, phenotype transition and stacked barplot analysis, to make comparative inferences about treatment conditions. In sum, SPOT makes the fundamental hypothesis that imaging artefacts are systematic, and ergodic (i.e. not time-dependent). Therefore, we expect pure imaging-artefact-only 'phenotype clusters' to exhibit minimal population changes over time, to not be the primary driver for the temporal evolution of phenotype, and to affect all conditions equally. Our additional validation experiment with chemotherapy drug, including the use of Caspase marker, show how multiple phenotype clusters (small organoids and large organoids) can correspond to cell death (Suppl. Fig. 3a). Moreover, independent SPOT analysis of organoids cultured in glass or plastic plates show the same broad classification of drug toxicity (less toxic: DMSO and Cisplatin vs more toxic: 5FU and Docetaxel), (Suppl. Fig 3b). Finally, the ability of SPOT to handle artefacts is further demonstrated using our 3D organoids simulator shown in the new Figure 2.

With respect to the specific comments about clusters shown in Fig. 3b, our interpretations of the results are listed below.

- *Cluster 3 and 4 organoids are out-of-focus:*

These organoids correspond to the same organoids that lie outside our imaged depth of 1000 µm. In the respective density plot (Fig. 3c ii), we see the primary density is located outside of the UMAP areas covered by clusters 3 and 4, and in the stacked barplot (Fig. 3c iii), we see that clusters 3 and 4 are relatively temporally constant subpopulations across time, similar across conditions and largely transiting only between themselves (Fig. 3c iv). The direction of the phenotype trajectories over time does not move towards the UMAP region covered by cluster 3 and 4 in any of the treatments, demonstrating that these 'artefact' clusters do not hinder biological interpretation (Fig. 3c v).

- *Cluster 2 organoids are overexposed:*

These organoids are in fact not overexposed: they have a transparent lumen which means that they are measured as being relatively brighter. In addition, e.g., from Fig. 3d, it can be seen that the intensity (SAM module 3) of Cluster 2 is less than Cluster 1. We know that this cluster reflects growing organoids because the stacked barplot (Fig. 3c iii) shows that its relative proportion expands over time in a stable manner, reflecting the fact that smaller organoids that were not previously classified as cluster 2 are now part of cluster 2 following their growth. We also know biologically that healthy, growing gastrointestinal organoids are transparent. They become dark and opaque with excessive cell death, leading to accumulation of cell death in the lumen. Suppl. Fig. 3a with Caspase and Cytotox verify the primary signatures of cell death as being darker and reduced movement.

- *Cluster 7 organoids are underexposed:*

In contrast to what is suggested by the Reviewer, these organoids are true phenotypes due to cell death where material is accumulated in the normally transparent lumen and the organoid shrinks/buckles in on itself (See Suppl. Fig. 3a with Caspase and Cytotox markers). We see this strongly in the phenotype trajectory following treatment with toxic drugs, which moves towards the UMAP regions covered by cluster 7 over time (Fig. 3c v). Correspondingly, the stacked plots show a much higher end proportion of cluster 7 organoids following

treatment with highly toxic drugs (Fig. 3c iii). Again, this underscores the importance of analyzing live-cell imaging and considering the temporal changes.

7) The label-free organoid segmentation is not accurate enough, and tracking of the organoids is not consistent over time (e.g. video 4) or the density of the organoids is way too high (e.g. video 5). Organoids merge together, overlap, get detected – undetected,… so there is no consistency that allows for kinetic measurements crucial for SAM. Again, this can all be resolved by the use of proper imaging-compatible culturing techniques and plates.

We thank the Reviewer for this comment and our newly generated synthetic organoid datasets with defined phenotypic behaviour have provided answers to this important criticism. In particular, we simulated the effect of increasing segmentation errors including overlap, cutout and union and tracking errors through fragmenting organoid tracks on SPOT analysis (Suppl. Fig. 2c). Our results show SPOT is robust to increasing amounts of these artefacts.

Additionally, we would like to clarify the approach in SPOT to ensuring temporal consistency and minimizing errors when generating the final organoid instances to compute SAM features: 1) we only need two frames to compute motion features; 2) organoid instances are only retained if the intersection-over-union of the segmentations frame-to-frame in the whole track is at least >0.25, which we find is an effective threshold for matching, taking into account an acceptable margin for shape changes over the elapsed hour between successive timepoints; 3) we temporally smooth segmentations over a time window which enables both temporal consistency and imputation of partial segmentations; 4) except for the centroid displacement features (only two features), the remaining motion features are based on the pixel-dense motion field, where every pixel of an image has an associated motion vector as computed independently with optical flow techniques. Importantly, optical flow does not depend on segmentation and its error can be constrained by incorporating physical constraints such as ensuring smoothness of motion field and constraining the size of the displacements; 5) the principle of SPOT to monitor the relative temporal change i.e., the delta in the measured heterogeneity over time across the whole population between different conditions. This is shown by our choice of clustering the phenotype trajectories using the dynamic time warping metric to compare trajectory shape instead of using absolute positional coordinates in the UMAP phenotypic landscape. Even if individual segmentation is inaccurate and noisy, as long as these errors are systematic over time, do not exhibit temporal variation, and average out over the population, we expect SPOT to detect relevant biological phenotypes. This is shown by our ability to resolve the effect of WNT addition, despite the organoid density, as independently validated by scRNA-seq (Fig. 5 and 6).

We would like to emphasize that the WNT biology that we are interested in is dependent on local organoid density. Moreover, the organoid flattening phenotype we observe is not local and covers spatial regions much larger than stereotypical spherical organoids. Here, the concept of organoid detection and segmentation does not apply and Particle Image Velocimetry (PIV) type of approaches are more applicable. Yet, SPOT is still able to resolve the phenotypes because we include comprehensive and redundant SAM measurements. If SPOT were only applicable to spatially delineated organoid cultures and relied upon accurate segmentation and tracking then it would have limited application to discovering novel

phenotypes. It is important to note that tissues are densely packed with cells with extensive cell/cell interactions, therefore certain key biological information will be lost in uniformly formed isolated organoids.

8) I'm wondering how much features are actually relevant. For example, for figure 3, studying the drug response in the organoids, the same conclusion can be drawn with just basic imaging features and do not require quantification of 2184 features. So for basic drug screening I think this is overkill.

We thank the Reviewer for this comment. We agree that for a basic toxicity screen, the number of features is overabundant. However, a central premise in our manuscript is whether we can compute a universal set of SAM features in the same universal manner that is equally applicable for analyzing simple (cell death) and complex scenarios (changes in organoid branching patterns). Whilst specifying a few targeted measurements such as biomarkers is simpler and more direct , it is not robust and interpretation may further be confounded by conflicting results. In Suppl. Fig. 3a, both Caspase and Cytotox are biomarkers of cell death yet they do not fully agree with each other. We also found we had to count the number of punctate spots instead of taking the mean intensity to get an interpretable quantification. For high-content and high-throughput applications, specifying more features than performing feature selection has proven to be more discriminative for discovery. For example, agnostic sequencing of all available transcripts is essential for scRNAseq to discover new cell types and corresponding biomarkers compared to classical microarrays.

9) In addition, the authors make statements about that certain clusters correlate with for example high levels of cell death (line 575) but no evidence of cell death is provided (e.g. fluorescent cell death marker). So I understand unbiased clustering of features is interesting, but it would be more interesting if they can be linked **to processes such as viability, cell death, apoptosis,… by using corresponding fluorescent markers.** ***This way you can determine SAM features related to specific biological processes and give meaning to a cluster (similar to linking scRNA seq clusters to biological pathways based on genesets).***

To demonstrate that SPOT phenotypic clusters can be associated to a biological process, we have performed the Reviewer's suggested additional staining experiments using caspase and cytotox to check the cell viability, cell death and cell apoptosis in the chemotherapy drug screen (new Supp. Fig 3a, 3b, 3c).

Our initial SPOT analysis suggested 3 toxicity groups (Fig. 3). We therefore selected the control DMSO and Cisplatin (6 µM and 100 µM) to represent the low-toxicity group, 5-Fluorouacil (5FU) (20 µM) for the mild toxicity group, and Docetaxel (12.5 nM) for the high-toxicity group. The concentrations chosen are the maximum level concentration used in Fig. 3. The organoids were grown for 6 days in a 96-well plate. The medium was changed with HOM and a titration of the drugs and the dyes as outlined below. Z-stack bright-field images and confocal images 1000 µm in-depth were acquired at 100 µm intervals (11 steps per acquisition) approximately every 4 hours for 5 days. Previously images were acquired every 1 hour for 5 days. The slower acquisition was used to reduce phototoxicity and to account for the additional imaging due to increased channels.

1. The cell viability during the drug screening was tested with Hoechst 33,342 (50 nM/well, ThermoFisher) consistent with literature (*OrBITS: label-free and time-lapse monitoring of patient derived organoids for advanced drug screening. Cellular Oncology. 2022*);
2. Real-time quantification of cell death induced by the toxicity of the drugs was assessed with **Cytotox Green** (60 nM/well, Sartorius, #4633), (*Multiparametric Tumor Organoid Drug Screening Using Widefield Live-Cell Imaging for Bulk and Single-Organoid Analysis. J. Vis. Exp. (190), e64434, doi:10.3791/64434 (2022))* and the cell death was calculated based on the number of detected punctate spots within each segmented organoid;
3. The evaluation of cell apoptosis during the drug screening was assessed with **Caspase 3/7 Green** (2.5 µM/well, Sartorius, #4440), (*OrBITS: label-free and time-lapse monitoring of patient derived organoids for advanced drug screening. Cellular Oncology. (2022); Viability Analysis and High-Content Live-Cell Imaging for Drug Testing in Prostate Cancer Xenograft-Derived Organoids. Cell. 2023)* by counting the number of detected punctate spots within each segmented organoid.

We performed SPOT analysis with the acquired datasets using our proposed SAM phenome only applied to the brightfield microscopy channels, then using the fluorescent Cytotox and Caspase biomarkers afterwards for interpretation. Our results, shown in the new Suppl. Figure 3a, show that SPOT identified 7 phenotype clusters of which clusters 2, 4, and 7 correlated with higher toxicity, with elevated number of measured Caspase and cytotox punctate spots. Interpreting with SAM modules, these clusters correspond two different death phenotypes: the normal accumulation of cell death in the lumen as organoids grow too large (clusters 2 and 4) and the drug-induced cell death whereby organoids remain small or potentially already dead (cluster 7). We found the total number of spots within a segmented organoid (see Methods) was a more reliable and informative measurement than green area/brightfield area due to the nonspecific auto fluorescence. Toxicity was measured highest for Docetaxel, which we had predicted by SPOT with SAM signature of low intensity and reduced movement. Moreover, our UMAP organized both Caspase and Cytotox markers similarly and in a gradient manner. Subsequently, hierarchical clustering of phenotype trajectory showed greater similarity of cisplatin to DMSO, then 5-FU, with Docetaxel most different, consistent with our findings of the larger screen with multiple drug concentrations in Fig. 3. Altogether our analysis demonstrates how SPOT uses multidimensional features and temporal change to robustly stratify toxicity phenotypes compared to using select single/few biomarkers which may itself be subject to experimental variation, and how they are used to extract measurements.

10) The authors use the term 'flattened organoid morphology' (line 765). It is difficult to see from the image, but are these cells that adhere to the plastic surface and start growing in 2D? This might be evidence of a more invasive, migratory behaviour.

We performed fixed staining at the endpoint of APC, KRAS, and p53 mutant mouse colon organoid movies with confocal z-stacks to assess and verify the observed organoid morphology from 2D projection. Using depth coloured maximum intensity projection (DCP), Suppl. Fig. 4b shows the morphological transformation in the APC mutant localizes preferentially at the interface of matrigel/substrate or boundaries of the matrigel ECM

dome (red and blue coloured organoids in DCP). We agree with the Reviewer that this may reflect a more invasive, migratory behaviour. Both p53 and KRAS mutants are more spherical and lie more centrally in the ECM dome (yellow/green coloured in the DCP).

11) While I find that the current version of the manuscript does not meet the necessary criteria for publication in its present form, I would like to emphasize the valuable insights and potential contributions your work offers. The breadth of the topics covered is commendable, yet a more focused approach could enhance the clarity and impact of your research. I strongly encourage you to consider refining the scope of your paper <u>or split up the paper into two or three papers, sharpening the central arguments, and addressing the highlighted issues</u>. A revised version that addresses these aspects would be a welcome submission to this journal. Your efforts in advancing research in this field are appreciated, and I look forward to potentially seeing a revised manuscript, in particular with a focus to organoids. I would like to express openness to potential collaboration. In my current research, we have developed an extensive library of organoid images treated with a variety of therapies. If you find it relevant, I believe a collaborative effort could enrich both our research endeavors, leveraging our respective resources and expertise.

Yours sincerely,
Prof. dr. Christophe Deben

We would like to thank the Reviewer again for recognising the value of our work and its potential to advance the field. We would also like to thank them for their constructive comments, which we have aimed to address here, and which have substantially improved the clarity of our manuscript. Once the manuscript is published, we will be delighted to collaborate with the Reviewer.

Reviewer #3 (Remarks to the Author):

1) The paper describes an extension to the toolbox of researchers that appears to outperform the established standard, namely CellProfiler. The authors argue that the introduction of motion in their pipeline, and a determination between global, local-regional and pattern-pixel levels are key differentiators for success. The authors support this statement well in text and figures, namely through the reporting of contribution scores for their clustering interpretation.

We thank the reviewer for their positive comments.

2) The validation work of the developed tool, SPOT, also revealed a potential new insight into the working of the WNT-pathway. The authors demonstrated that for two contradicting configurations, WNT containing mouse organoids and WNT deprived human organoids, the observed morphological behavior to have a similar underlying cause. The authors support this with single-cell-sequencing and investigated phototoxicity as a potential cause of these events. I believe this to be a new finding, with a clear and soundly executed methodology.

We thank the reviewer for highlighting the robustness of our methodology and the novelty of our approach.

3) The currently proposed tool, SPOT, has been validated on multiple 2-dimensional datasets. The authors do show the potential of spot for organoid segmentation from Matrigel droplets, a 3-dimensional environment where organoids significantly overlap in the image. It is however currently unclear whether SPOT will work well for 3-dimensional time-series data. A possible complication can be that the motion module in SPOT can confuse X/Z movement for a phenotypic response of cells.

To test the applicability of SPOT analysis of 2D projections to characterize 3D organoid dynamics, we generated simulated organoid Dataset B. Organoids with prescribed shape, appearance and motion 3D dynamics were individually simulated and positioned in 3D scenes. The 3D scenes were then projected as 2D videos for SPOT analysis. We simulated three different 3D motion scenarios: the first condition is of full unrestricted 3D XYZ movement; the second condition restricts movement to 2D XY planar movements only but with the same speed as the first condition; the final third condition is a replicate of full unrestricted 3D XYZ movement with different simulated organoids. We find that whilst visually the phenotype trajectory and stacked barplots of phenotype cluster frequency over time appear indistinguishable, SPOT nevertheless detected differences and reliably clusters the XYZ movement conditions distinctly from the XY planar movement condition, (Suppl. Fig. 2d). We thus conclude the ability of SPOT to reliably differentiate the subtle visually undetectable Z movement demonstrates the suitability of SPOT for 3D time series analysis from 2D projection videos.

4) Overall, the paper is very well written, providing a new toolbox to researchers that has the potential to become of significant value to the field.

We thank the reviewer for their comments and for highlighting the value that our technology will bring to the field.

NCOMMS-23-61673-B (Revised manuscript, organoid-focussed application of SAM-SPOT)		
**Current Figure**	**Previous Figure (NCOMMS-23-61673-T first submission)**	**Description**
Figure 1	New	Comprehensive shape, appearance and motion (SAM) characterization enables analysis of phenotypic heterogeneity in organoid models.
Figure 2	New	SPOT analysis of synthetic organoid video dataset with prescribed shape, appearance and motion dynamics.
Figure 3	Figure 3	SPOT phenotype analysis of human patient-derived duodenum organoids drug response enables clustering of drugs by pharmacological action.
Figure 4	Figure 4	SPOT identifies unique phenotypic differences in genotype defined murine colon organoids.
Figure 5	Figure 5	SPOT captures the timing of WNT signaling-induced phenotypic dynamics in patient-derived duodenal organoids.
Figure 6	Figure 6	SPOT complements molecular sequencing for scientific and biomedical discovery
Suppl Figure 1a	New	Brightfield phase contrast imaging has limited depth resolution and extended focus projection of a z-stack to a 2D image
Suppl Figure 1b	New	Acquired confocal z-stacks have depth-limited resolution.
Suppl Figure 2a	New	SPOT Phenotype cluster transition and density maps of the simulated organoid dynamics dataset A.
Suppl Figure 2b	New	Robustness of SPOT analysis to imaging artefact, segmentation and tracking errors
Suppl Figure 2c	New	Quantitative assessment of SPOT analysis robustness to imaging artefact, segmentation and tracking errors
Suppl Figure 2d	New	SPOT analysis of 2D projections discriminates z-component motion.
Suppl Figure 3a	New	SPOT phenotype clusters capture different SAM characteristics of cell death after drug treatment.
Suppl Figure 3b	New	Independent SPOT analysis of organoid culture in plastic transparent and optical-bottom plates

Suppl Figure 3c	New	Joint UMAP analysis of chemotherapy treated patient-derived organoids cultured in plastic and glass-bottom plates
Suppl Figure 3d	Extended Fig. 3	SPOT characterizes changes in murine small intestine organoid branching dynamics when treated with HDAC inhibitors.
Suppl Figure 4a	Extended Fig. 4	SPOT draws the same conclusions with label-free imaging of genotype defined murine colon organoids.
Suppl Figure 4b	New	Murine colon organoids exhibit flattened morphologies preferentially at boundary of ECM dome
Suppl Figure 5	Extended Fig. 5	SPOT detects changes in WNT signaling-induced phenotypic dynamics in patient-derived duodenum organoids

Companion Methods Development Focussed Manuscript		
**Current Figure**	**Previous Figure (NCOMMS-23-61673-T first submission)**	**Description**
Figure 1	Figure 1a, b	Shape, appearance and motion phenome characterize imaging phenotypes
Figure 2	Figure 1d	Development of SPOT
Figure 3	Extended Figure 1a-g	Testing Shape, appearance features on public computer vision datasets
Figure 4	Extended Figure 1h-j; New	Testing combined SAM on A2D YouTube dataset
Figure 5	Figure 2	Single cell tracking challenge migration dataset
Figure 6	New	SAM-SPOT and CNN-SPOT analysis of LIVECell dataset
Figure 7	New	SAM-SPOT and CNN-LSTM-SPOT analysis of single cell division dataset (HeLa histone H2B)
Suppl Figure 1	New	Assessing the number of contour points required for shape representation
Suppl Figure 2a	New	Schematic illustrating SPOT module analysis in analogy with scRNA-seq analysis
Suppl Figure 2b	New	Schematic of SPOT module analysis and computation of SAM/scope contribution scores
Suppl Figure 3	Suppl Figure 1	Comparison of SAM and Cellprofiler features to computer vision datasets
Suppl Figure 4a	Suppl Figure 2	A2D dataset similarity graph of joint object-movement classes
Suppl Figure 4b	Suppl Figure 3	A2D dataset transformed into 2D, applying different dimensionality techniques to SAM phenome
Suppl Figure 5	Extended Figure 2	Single cell division dataset, Cell tracking challenge
Suppl Fig 6	New	SAM-SPOT and CNN-SPOT analysis of LIVECell dataset
Suppl Fig 7	New	UMAP density heatmaps of SAM-SPOT and CNN-SPOT analysis of LIVECell dataset
Suppl Fig 8	New	SAM-SPOT and CNN-LSTM-SPOT analysis of single cell division dataset (HeLa histone H2B)
Suppl Fig 9	New	UMAP density heatmaps of SAM-SPOT and CNN-LSTM-SPOT analysis of single cell division dataset (HeLa histone H2B)

Reply to Reviewers' main concerns:

The Reviewers raised three main concerns:

1) SPOT's ability to deal with the presence of artefacts in the analysis;

2) the impact of potential inaccuracies in object segmentation and tracking; and

3) SPOT's suitability for 3D time-series imaging data.

To address all three of these concerns systematically and objectively, we built an organoid simulator to synthesize simulated *de novo* video datasets with known ground truth dynamic phenotypes. Each organoid can be prescribed with defined and dynamic organoid phenotypes, to simulate changes in only one or more of shape, appearance, or motion characteristics. Below, we summarise our response to the Reviewers' three primary concerns:

1. ***Potential sensitivity to artefacts:***

Reviewer 2: ***My main concern is the images that are used for the organoids. SAM quantifies 2185 features, so it is very sensitive to imaging artifacts.***
From image 3B it is very evident that the clusters are mainly based on imaging artefacts. For example, cluster 3 are out of focus organoids, cluster 2 are overexposed organoids, cluster 7 are underexposed organoid, cluster 4 out of focus and underexposed… Although there might be some phenotypic differences, features extracted from these images are completely unreliable due to the imaging artefacts.

Reply:
We apologise for not making it clear that including and reconciling artefacts within SPOT's analysis is a unique designed feature. The phenotype clustering is designed to identify different 'modes' or SAM imaging 'states' in the dataset by discretely partitioning the UMAP phenomic landscape. Each cluster is not necessarily a biological phenotype, and more than one cluster could relate to the same biological process e.g., two clusters relate to cell death. The primary biological analysis is in the phenotype evolution of organoids between conditions. SPOT assumes that over time, only phenotype clusters corresponding to true biological phenotype dynamics will exhibit differential temporal evolution, and this gives a signature to cluster conditions. Not all analysis uses this clustering. SAM phenotype trajectories are derived by the majority UMAP density in each temporal bin. In all cases, artefacts, when present, are assumed to not exhibit temporal evolution and to affect all conditions equally. We demonstrate the interpretation of phenotype cluster in using the simulated organoid dataset (**new Fig. 2**, NCOMMS-23-61673-C) and new supplementary chemotherapy drug screens analysed with the addition of a fluorescent caspase marker (**new Suppl. Fig. 3a,** NCOMMS-23-61673-C). In the latter, we found at least three SAM phenotype clusters associated with elevated caspase expression, indicative of different modes of death.

Imaging artefacts are almost always present in some manner during timelapse acquisition. Importantly, these artefacts differ between imaging modality and imaged subject. As a tool designed for high-content analysis, we believe that it would be a limitation to have to firstly

carefully remove artefacts, which requires domain expertise and potentially extensive manual proofreading. Naturally, if the user has trained an appropriate artefact detector for their data, this can be applied to further improve analysis and enhance biological interpretation. Nevertheless, as we demonstrate by analysing simulated organoids subject to defined artefact type perturbations in the **new Suppl. Fig. 2**, NCOMMS-23-61673-C , that SPOT is either robust to an artefact type (such as blurring and intensity changes due to photobleaching), or, it makes the artefacts easily identifiable in the 2D UMAP to allow data subsetting. For example, when we perturbed the ground truth simulated dataset with 'union' segmentation errors of varying severities (illustrated in **Suppl. Fig. 2b a,** NCOMMS-23-61673-C), the UMAP cleanly partitions the ground truth from the perturbed conditions. We can thus subset for and reperform analysis on only the unaffected images (**Suppl. Fig. 2b c,** NCOMMS-23-61673-C).

2. Potential inaccuracies in object segmentation and tracking

Reviewer 1: The success of the whole pipeline is dependent on accurate object tracking/tracklet. Can you quantify the accuracy of this step of your pipeline and discuss how this accuracy will affect the downstream results numerically?

Reviewer 2: The label-free organoid segmentation is not accurate enough, and tracking of the organoids is not consistent over time (e.g. video 4) or the density of the organoids is way too high (e.g. video 5). Organoids merge together, overlap, get detected – undetected,… so there is no consistency that allows for kinetic measurements crucial for SAM. Again, this can all be resolved by the use of proper imaging-compatible culturing techniques and plates.

Reply:
SPOT inherently includes safeguards that may affect analysis. With organoid detection, SPOT enforces a minimum temporal consistency in segmentation: all instances of a tracked organoid must have a frame-to-frame shape overlap (as measured by intersection-over-union (IoU)) greater than a user specified threshold. If this cut-off is too high, matching may be too stringent to shape changes between frames. We use a cut-off of IoU=0.25. We also emphasise that the tracking is implemented only to identify genuine organoids (alive or dead) which we define as being tracked for a minimum number of frames (here, 5 frames) from potential short-lived transient artefacts e.g., floating debris. In SPOT analysis, the tracked organoids at different timepoints are mapped as independent individual points in the UMAP. Only the motion features require tracking to compute. Even so, it depends only on a consecutive pair of accurate segmentations. In summary, SPOT is expected to be robust even if not all object instances are perfectly segmented (due to overlap for example), and robust in the case where some object instances are undetected or filtered out (such as by the IOU tracker, causing fragmented tracks). We validate our claims using our simulated 3D organoids, by introducing varying degrees of segmentation errors 'cutout' and 'union'), that could result from overlap. If such instances occur and are highly prevalent, we find they cluster distinctly and can be removed (**new Suppl. Fig. 2b c,** NCOMMS-23-61673-C). In addition, we simulated the effect of various degrees of track fragmentation (such as that caused by organoid instances remaining undetected or being filtered out), showing that the conclusions drawn from SPOT analysis remain unaffected (**new Suppl. Fig. 2b d,** NCOMMS-23-61673-C).

3. Potential issues with SPOT's use for 3D time-series data

Reviewer 1: The 3D organoid imaging produces z-stack 3D images. Is it too rough to simply project the z-stack to one 2D image as in line 1476? You may want to as least discuss on how to better treatment 3D images.

Reviewer 3: Whether SPOT will work well for 3-dimensional time-series data, a possible complication can be that the motion module in SPOT can confuse X/Z movement for a phenotypic response of cells.

Reply:

We agree with the Reviewers that our study presented here is mainly based on projected 2D images from time-series data, and we recognize the limitation of the current version of SPOT to capture the full richness of 3D biology. The organoids we culture in our studies over 2 weeks grow large such that true 3D imaging acquisition of entire organoids at near-isotropic resolution would require sophisticated and customized light-sheet imaging setups, involving multiple objectives to try to overcome depth-based light scattering. These costly setups are not able to capture all organoids in the field-of-view in a high content nor high through-put manner, nor are they widely accessible. Therefore, we feel that only for particular biological hypotheses would an imaging tool that can accurately analyse true 3D time-series data at the cell/organoid scale be needed currently. Moreover, capturing full 3D significantly enhances phototoxicity and limits both total acquisition time and acquisition frequency.

Organoids are grown in 3D and have emerged as a valuable experimental tool for high-throughput genetic and compound screens because they can recapitulate the cell lineages of their origin organ. A major application is identifying earlier markers of disease transformation. However, organoids take time to develop as the cells require time to differentiate. Consequently, it is much more valuable in live-imaging organoid experiments to take steps to minimize phototoxicity and maximize acquisition length. The standard practice in both pharmaceutical industry and research communities is for label-free microscopy such as bright-field and phase contrast microscopy modalities with small z-stacks. We follow this practice in this work, by imaging a few z-slices for up to 2 weeks and maintaining a relatively high acquisition rate of one frame per hour. In this paper, this imaging acquisition enabled us to reveal developmental differences due to differences in WNT signalling, in addition to the fact that these differences required several days to manifest, beyond the capability of commercial fluorescent lightsheet systems (**Fig. 5**, NCOMMS-23-61673-C). Moreover, as we highlight in **Suppl. Fig. 1a,b of** NCOMMS-23-61673-C, our data whether label-free or confocal have insufficient z-resolution and offers no real further insight beyond 2D analysis, even if we were to extend SAM-SPOT to perform a full 3D analysis.

To further address the Reviewers' concerns on the applicability of analysing 2D projection videos of z-stacks, we generated synthetic organoid datasets (see below). Simulated dataset B was specifically designed to assess the potential added value of capturing full 3D dynamics. Our results shown in the **new Fig. 2**, NCOMMS-23-61673-C, illustrates that SPOT captures sufficient information in 2D projection videos to objectively discriminate between a condition where full 3D xyz movement was permitted from a second condition where movement was constrained to the 2D xy-plane.

4. *Creation of organoid simulator and synthetic datasets to validate the applicability and potential of SPOT*

We created an organoid simulator to objectively address all Reviewers' concerns on the sensitivity of SPOT to dataset and processing imperfections and on the extent to which 2D projection videos distinguish 3D phenotypes. Specifically, we generated two synthetic organoid video datasets (Simulated Datasets A and B) where we engineered the 3D phenotypes, then constructed and analysed the associated 2D projection videos with SAM-SPOT. These new datasets with known ground truth enabled us to address SPOT's ability to recognise and reconcile:

1) Noise and artefacts in the analysis;
2) Track fragmentation;
3) Inaccuracies in object segmentation caused by spatially overlapping organoids; and
4) SPOT's suitability for 3D time-series imaging data.

Points #1–3 are addressed by 'Simulated Dataset A', while the consequence of movement in the Z-dimension, Point #4, is addressed using 'Simulated Dataset B'. The synthetic organoids are simulated fully in 3D and then projected into 2D using the same extended focus projection principles as that used for real acquisitions. Using simulations enabled us to have ground-truth phenotypes and to apply a wide range of systematic perturbations (including those identified by the Reviewers) to test how effectively SPOT can extract 3D information from 2D projections, to assess how effectively SPOT can distinguish distinct phenotype categories with a known ground-truth, and to determine how robust SPOT analysis remains in the face of experimental, imaging, and segmentation imperfections, thereby enabling tolerance thresholds to be set.

Simulated Dataset A

We generated a synthetic dataset of simulated 3D organoids, that evolve across time from a small, round initial state into a phenotypically distinct final state (7 conditions, 45 organoids per condition, and 30 frames per organoid) (**new Fig. 2b,** NCOMMS-23-61673-C). Each of the conditions share a common starting state with identical shape, appearance, motion behaviour parameterisation, before diverging over the duration of the 30-frame time course into specifically-parameterised, condition-specific end states. To generate this dataset, we define one condition-dependent end state as the 'base organoid', with 'basal' parameter values. Then, using the base organoid parameterisation as the template, we modified parameters influencing each of shape, appearance, and motion in isolation, to create 6 further extremal 'high' or 'low' conditions for each concept. The dataset is comprised of 3-dimensional organoid simulations and their 2-dimensional projections along with pixel-perfect segmentation masks. To generate the 2-dimensional projections, we used an equivalent method to the extended depth of focus projection used on our real timelapse z-stacks. We subjected the entirety of Simulated Dataset A to 8 different perturbation types, each across 9 severity levels, to identify effects on the analysis relative to the known ground truth.

Simulated Dataset B

We generated another synthetic dataset of simulated 3D organoids, with 3 conditions that share the same shape and appearance parameters, with organoids moving with the same speed but differing in the allowed movement directionality. One condition represents unrestricted xyz movement freedom, a second condition represents a replicate of the first condition with different organoids and the third condition represents movement only in the plane parallel to the projection i.e., xy plane (in total, 3 conditions, 6 replicates per condition, 12 organoids per replicate, 30 timepoints per organoid). This dataset was designed to address the specific concerns of Reviewer 3 regarding the effect of movement in the Z-dimension on phenotype readout.

The key findings of applying SAM-SPOT to the synthetic dataset A is included as **new Figure 2 of** NCOMMS-23-61673-C in the main text. The results of the synthetic robustness tests using perturbations of dataset A, and analysis of dataset B are included as additional **Suppl. Fig. 2a and 2b,** NCOMMS-23-61673-C.

EXTENDED POINT-BY-POINT RESPONSES TO EACH REVIEWER'S COMMENTS:

Reviewer #1 (Remarks to the Author):

This manuscript introduced a pipeline to track the phenotypic changes of objects in 2D videos, and applied it on living-cell videos. Together with single cell sequencing data, this tool may study the phenotype-genotype association. I have the following comments:

1) It is hard to call any chosen summary features as comprehensive and unbiased phenotypic description of the video, as long as you do not use all the pixel information.

We apologise for not defining the terms "comprehensive" and "unbiased" clearly in the text. We use the terms "comprehensive" and "unbiased" to refer to the way in which the image features are acquired and how the acquired data are analysed, respectively. Specifically, SAM features are "comprehensive" because they have been designed to be a generally applicable set of image features covering all spatial scales of global, regional, and local (i.e. distributional) (akin to late and early layers of a neural network) and the different attributes of shape, appearance and motion. We note local distribution features are derived by using all the pixel information. The specific composition of features is now illustrated in **Fig. 1** of our new companion paper, NCOMMS-25-25126, which we include below for reference.

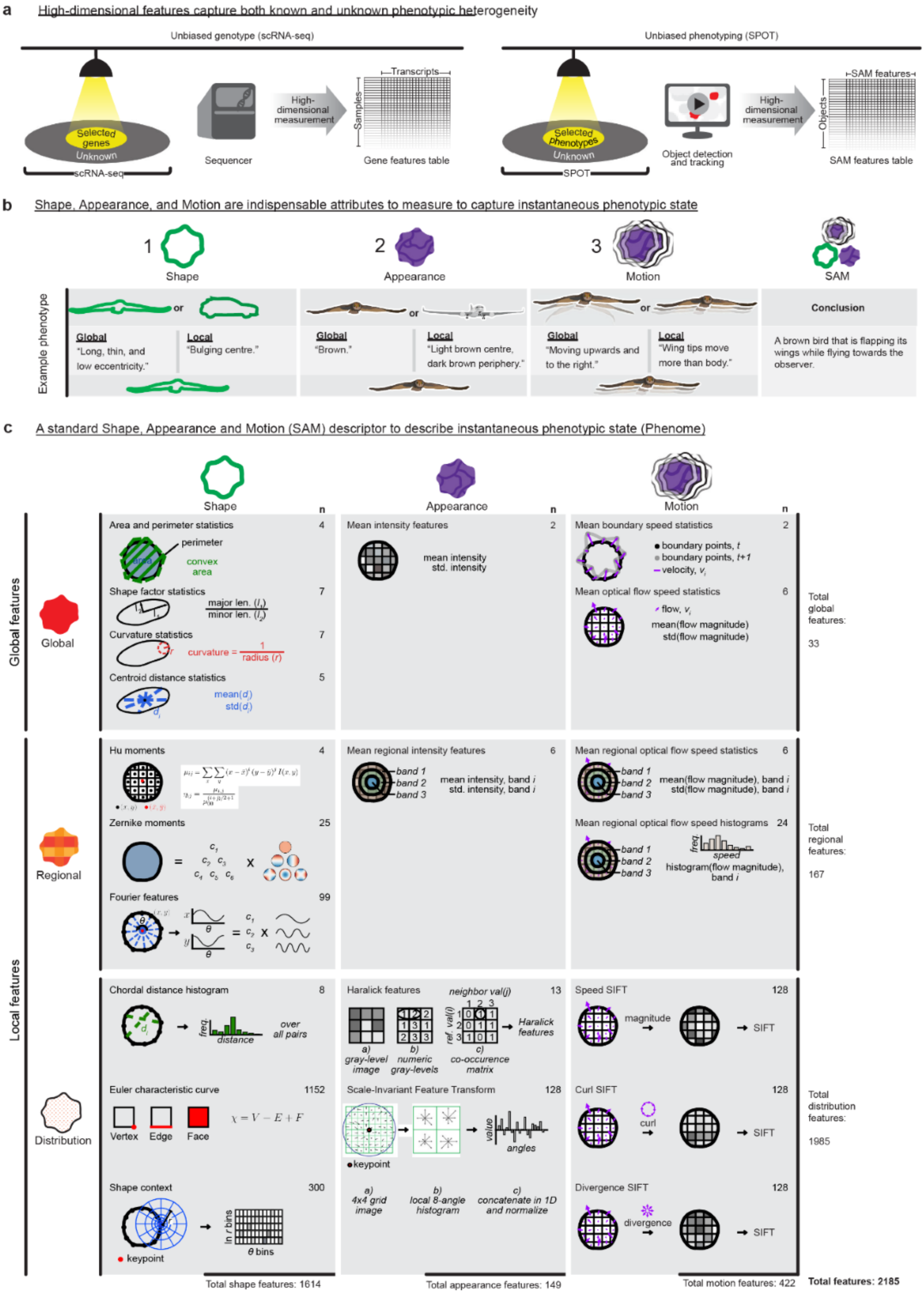

a
High-dimensional features capture both known and unknown phenotypic heterogeneity
Unbiased genotype (scRNA-seq)
Selected genes
Unknown
scRNA-seq
Sequencer
High-dimensional measurement
Transcripts
Samples
Gene features table
Unbiased phenotyping (SPOT)
Selected phenotypes
Unknown
SPOT
Object detection and tracking
High-dimensional measurement
SAM features
Objects
SAM features table
b
Shape, Appearance, and Motion are indispensable attributes to measure to capture instantaneous phenotypic state
1 Shape
2 Appearance
3 Motion
SAM
Example phenotype
or
Global
"Long, thin, and low eccentricity."
Local
"Bulging centre."
Global
"Brown."
Local
"Light brown centre, dark brown periphery."
Global
"Moving upwards and to the right."
Local
"Wing tips move more than body."
Conclusion
A brown bird that is flapping its wings while flying towards the observer.
c
A standard Shape, Appearance and Motion (SAM) descriptor to describe instantaneous phenotypic state (Phenome)
Shape
n
Appearance
n
Motion
n
Global features
Global
Area and perimeter statistics 4
perimeter
convex area
area
Shape factor statistics 7
major len. (l_1) / minor len. (l_2)
Curvature statistics 7
curvature = 1 / radius (r)
Centroid distance statistics 5
mean(d_i)
std(d_i)
Mean intensity features 2
mean intensity
std. intensity
Mean boundary speed statistics 2
boundary points, t
boundary points, t+1
velocity, v_i
Mean optical flow speed statistics 6
flow, v_i
mean(flow magnitude)
std(flow magnitude)
Total global features: 33
Local features
Regional
Hu moments 4
Zernike moments 25
Fourier features 99
Mean regional intensity features 6
band 1
band 2
band 3
mean intensity, band i
std. intensity, band i
Mean regional optical flow speed statistics 6
mean(flow magnitude), band i
std(flow magnitude), band i
Mean regional optical flow speed histograms 24
freq
speed
histogram(flow magnitude), band i
Total regional features: 167
Distribution
Chordal distance histogram 8
freq.
distance
over all pairs
Euler characteristic curve 1152
Vertex
Edge
Face
$\chi = V - E + F$
Shape context 300
ln r bins
θ bins
keypoint
Haralick features 13
neighbor val(j)
ref. val(i)
Haralick features
a) gray-level image
b) numeric gray-levels
c) co-occurence matrix
Scale-Invariant Feature Transform 128
keypoint
value
angles
a) 4x4 grid image
b) local 8-angle histogram
c) concatenate in 1D and normalize
Speed SIFT 128
magnitude
SIFT
Curl SIFT 128
curl
SIFT
Divergence SIFT 128
divergence
SIFT
Total distribution features: 1985
Total shape features: 1614
Total appearance features: 149
Total motion features: 422
Total features: 2185

Figure 1 of NCOMMS-25-25126. Comprehensive shape, appearance and motion (SAM) characterization enables analysis of phenotypic heterogeneity in timelapse imaging. a) *Measurement of a standardized high-dimensional library of gene transcripts in single-cell RNA sequencing (scRNA-seq) enables unbiased characterization of the full transcriptome: both that which is known or selected a priori (yellow circle) and unknown (grey ring). Similarly measurement of a standardized high-dimensional set of SAM features could characterize imaging phenotypes that are known or selected a priori (yellow circle) and unknown (grey ring).* ***b)*** *Example illustrating how shape, appearance and motion cues provide complementary information and are the minimum three essential concepts necessary to describe the instantaneous state of a dynamic object fully.* ***c)*** *Illustration and categorization of the standardized SAM feature set (phenome) used in this study with respect to shape, appearance, or motion (columns) and the spatial scale of object detail they measure (rows). Global refers to the whole object, regional refers to a part of the object, and distribution measures the spatial scale of the object at the level of individual constituent pixels. See Suppl. Table 1 for the definition of individual features. The number of feature dimensions is given assuming an object contour of 200 equidistantly spaced points.*

We use the term *unbiased* to emphasise that all features in the SAM phenome can be and are computed for all datasets in the same manner and are not 'handcrafted' to target a particular phenotype *a priori* (e.g. organoid branching-specific) nor require adaptation to be computed for any imaging modality or imaged object (e.g. number of branches is a feature specific only to objects that branch). This is in contrast to data-driven learning such as convolution neural network (CNN) features trained on a particular dataset, which is by definition a "*biased"* approach in that it necessarily requires training on the target dataset to guarantee relevance of generated features.

Our new companion methods-focussed paper entitled *"Shape, Appearance, and Motion (SAM) features enable construction of the phenome and development of SAM Observation Tool (SPOT)"* (submitted under the identifier NCOMMS-25-25126) describes in detail the rationale used for constructing the SAM phenome.

"*Specifically, to ensure a maximally applicable SAM phenome for real data, we established our SAM phenome upon four design principles (Fig. 1c). First, to be comprehensive, SAM properties are measured at multiple spatial scales; global (whole object), local-regional (parts of whole object) and local-(pixel-level) distribution. Second, at each scale, a broad set of SAM features is measured to capture all potential SAM variations, not limited to only identifying object phenotypes within a particular analyzed dataset. This includes measuring redundant features, which characterize the same property but are each computed differently. Redundancy builds robustness to inevitable inaccuracies in segmentation and tracking, whilst*

still providing additional information. For example, area is significantly more sensitive when an object is partially segmented than the convex area. However, using convex area may underestimate the difference in actual area between two genuinely concave shapes, e.g. a cell with protrusions or that between a convex and concave shape. Third, features are computed to be invariant to object translation or rotation. Evidently, the object is the same under these transformations and therefore should have the same SAM phenome. Reflections of an object are treated as being a different object and therefore have different SAM phenomes. These might reflect differences in the underlying biological process or systematic difference in acquisition (e.g. camera flipped). Last, to enable application to videos of any duration and acquisition frequency, we consider only first order motion features; those computable using a pair of consecutive frames. Higher order motion properties such as acceleration are implicitly captured in tracking over multiple frames.

Briefly, for Shape, we compiled 1614 features, including 23 global (area and perimeter, shape factor, curvature and centroid distance statistics), 131 regional (Fourier features[62,63], Hu[64] and Zernike[62,65] moments) and 1460 distribution features (chordal distance histogram[36], Euler characteristic curve[66,67], shape context[68]). For Appearance, we compiled 149 features, including 2 global (mean intensity), 6 regional (regional intensity) and 141 distribution features (Haralick[69], Scale-Invariant Feature Transform (SIFT)[70,71]). For Motion, we generated 442 features, including 8 global (mean boundary speed, mean optical flow speed statistics), 30 regional (mean regional optical flow speed statistics and histograms) and 384 distribution features (Speed, Curl and Div SIFT). The concatenation of all Shape, Appearance and Motion features (1614+149+442=2185) form our final 2185-D SAM phenome (Fig. 1c and Suppl. Table 1).”

Finally, we agree with this reviewer that a higher number of features may be more comprehensive, but too many features make the phenome ‘too unique’ leading to poorer performance without further sophisticated preprocessing. Our reasoning is detailed in the discussion of our new methods focussed manuscript NCOMMS-25-25126, supported by empirical benchmarking results on computer vision datasets:

“Rational construction of the SAM phenome was critical to this success (Fig. 1, Methods). A sufficient number of image features is necessary to be 1) comprehensive; characterizing the properties of shape, appearance, and motion at multiple spatial scales (global, regional and pixelwise (i.e. distribution)); and 2) robust to artefacts from image acquisition, segmentation and tracking; achieved by computing redundant features with complementary information e.g., computing both area and convex area. However, excessive features make each SAM

phenome too 'unique', and hamper pattern finding. As such, our final full 2185-feature phenome represents a good balance. This principle can be best seen in our benchmarking of different subgroupings of SAM-S features on MPEG-7 shapes (Suppl. Table 2). The full SAM-S (kernel ECC) phenome (ranked 1st, Suppl Table 2) outperformed all subgroupings, but only due to preprocessing of ECC features (n=1152, 11th) to reduce its dimensionality to 100 features before concatenation. The raw SAM-S phenome (n=1614) significantly underperforms, ranking 6th, thus lower than its constituent subgroupings: Shape context (3rd, n=300), Zernike moment (4th, n=25) and geometrical (5th, n=17). It might also be supposed that an imbalance in the number of shape, appearance and motion-associated features in our SAM phenome may bias analysis. However, we do not find evidence of this empirically. The contribution scores computed by SPOT in all studied datasets reflect the expected relative importance of shape, appearance and motion. Notably, SAM-SPOT analysis identified specific shape, appearance and motion-associated modules in a simulated organoid dataset relating to the predefined shape-, appearance- and motion-specific phenotypes (NC-SPOT-MS1)."

The relevant results table assessing the use of subsets of the SAM-S phenome is included below (**Suppl. Table 2** of NCOMMS-25-25126):

	k-means clustering to identify MPEG-7 shape categories (k=70)					
	**Feature Descriptor**	**AMI**	**ARI**	**1/2*(AMI+ARI)**	**Average Ranking**	**Feature Scope**
Subset features of SAM-A	Shape context, n=300	0.652	0.435	0.544	3	Distribution
	Zernike moment, n=25	0.638	0.413	0.526	4	Regional
	Geometrical, n=17	0.587	0.378	0.483	5	Global
	chordal distance histograms, n=8	0.555	0.327	0.441	7	Distribution
	kernel Euler Characteristic Curve (kernel ECC), n=100 **	0.528	0.320	0.424	8	Distribution
	centroid statistics, n=5	0.510	0.296	0.403	9	Global
	Hu moments, n=7	0.469	0.200	0.335	10	Regional
	Euler Characteristic Curve (ECC), n=1152	0.506	0.133	0.320	11	Distribution
	Fourier features, n=99	0.275	0.114	0.195	12	Regional
l SAM Appearance descriptor (SAM-	SAM-S (ECC), n=1614	0.634	0.327	0.481	6	Combination
	SAM-S (kernel ECC), n=562	0.710	0.518	0.614	1	Combination
Cellprofiler Shape features	Cellprofiler shape features, n=98	0.684	0.466	0.575	2	Combination
Negative control	Random label permutation	0.000	0.000	0.000	13	n/a

**** : kernel ECC is a dimensionality reduction of ECC features, based on comparing the similarity of ECC over multiple objects and therefore is not a direct measure but only computable over a given video dataset**

AMI - adjusted mutual information score (upper-bounded by 1) the larger the better. https://scikit-learn.org/stable/modules/generated/sklearn.metrics.adjusted_mutual_info_score.html

ARI - adjusted rand index score (-0.5 to 1.0). -0.5 = especially discordant, 0.0 = random labelings, 1.0 = perfect match https://scikit-learn.org/stable/modules/generated/sklearn.metrics.adjusted_rand_score.html#sklearn.metrics.adjusted_rand_score

2) For example, your feature does not cover the acceleration of segmented objects even if you only work on 2D objects from single color 2D images

Reply:

As acceleration is the time derivative of velocity, we would expect a change in acceleration to manifest in the next time frame as a change in velocity. Therefore, SPOT does implicitly capture the acceleration of segmented objects. Generally, we expect higher-order motion statistics to manifest as increasing temporal delays, requiring longer tracking to capture. One frame in a video can represent any temporal unit from 1 ms to 1h to 1 day, depending on the video in question. Equally, the total number of frames can vary. Therefore, to ensure maximum applicability, we designed the SPOT analysis method to only include motion measures that can be computed from 1 successive frame. We have explicitly clarified this in our new companion methods-focussed paper, with the relevant text cited in the above point. Incidentally, this is why we refer to our construction as the instantaneous 'SAM phenome' and can use this phenome to map all temporal object instances as individual points in a 2D landscape using UMAP. We note that long-term dynamics are not lost however and are modelled back through the single object phenotype trajectories and phenotype cluster transitions, best demonstrated by our analysis of cell division in **Fig. 7** of our methods-focussed companion paper NCOMMS-25-25126.

As seen in the selected panels b) and e) below, the SAM phenome derived phenomic landscape maps all seven cell division stages, and the SAM phenotype trajectory constructed using the landscape successfully infers a cyclic path recovering the temporal order of stages. Notably each turning point in the trajectory denote a significant change in SAM characteristics between stages.

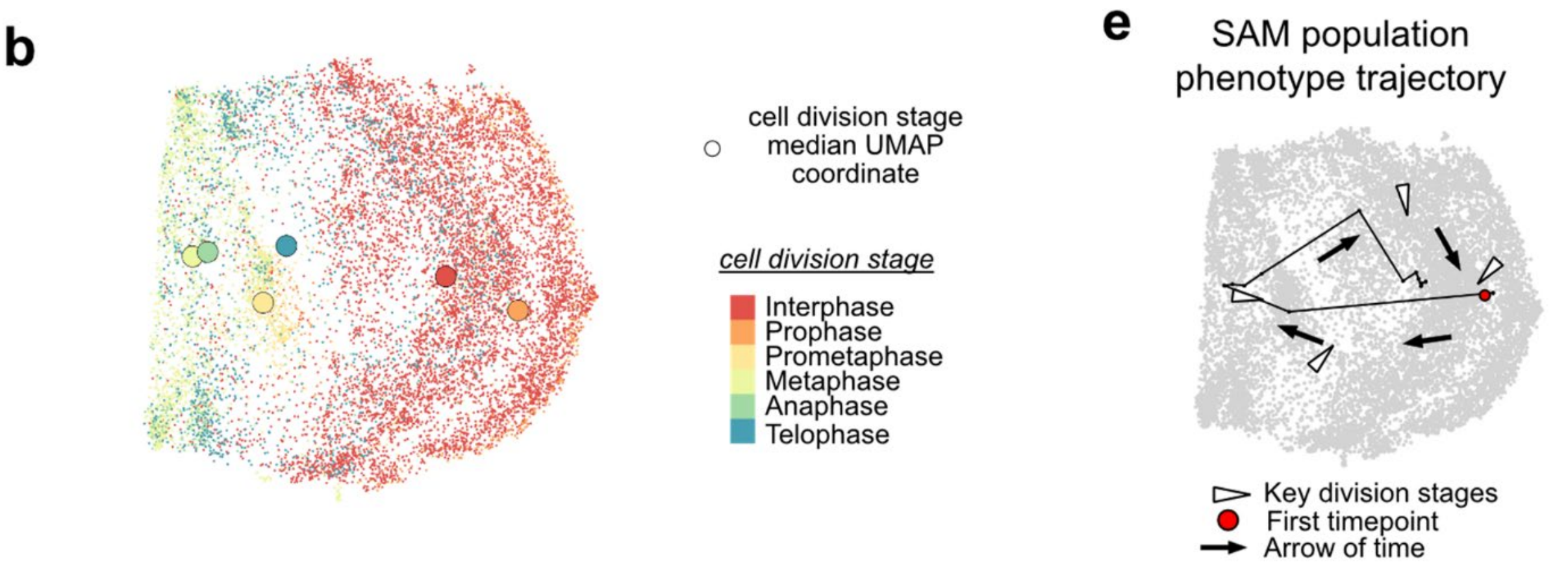

3) line 169-170: how did you select the tuning parameters 200 and 3 here? Is it possible to automatically select by certain version of cross-validation?

Reply:
We want to store the segmented organoids in a more efficient manner than storing each binary pixel in individual cropped segmentation masks. However, individual boundaries have different numbers of points and would not allow the stacking of multiple boundaries into a regular array, resulting in larger and irregular storage on disk. We therefore chose to sample the contour of each organoid with 200 equidistant points (see **Suppl. Fig. 1** of our methods-focussed companion manuscript NCOMMS-25-25126, included below), as comparable numbers have been used in the literature to represent complex cell morphologies e.g. CAJAL (Govek et al., *Nat. Commun.,* 2021) which used 100 points to classify diverse neuron morphologies. To demonstrate that 200 points does still retain good shape approximation

empirically, we assessed the symmetric Hausdorff distance of MPEG-7 shapes with increasing number of sampled equidistant contour points. The MPEG-7 dataset, (https://dabi.temple.edu/external/shape/MPEG7/dataset.html) captures diverse shape variations in 70 different everyday objects. We find rapidly diminishing returns in capturing the shape complexity above 100 equidistance points.

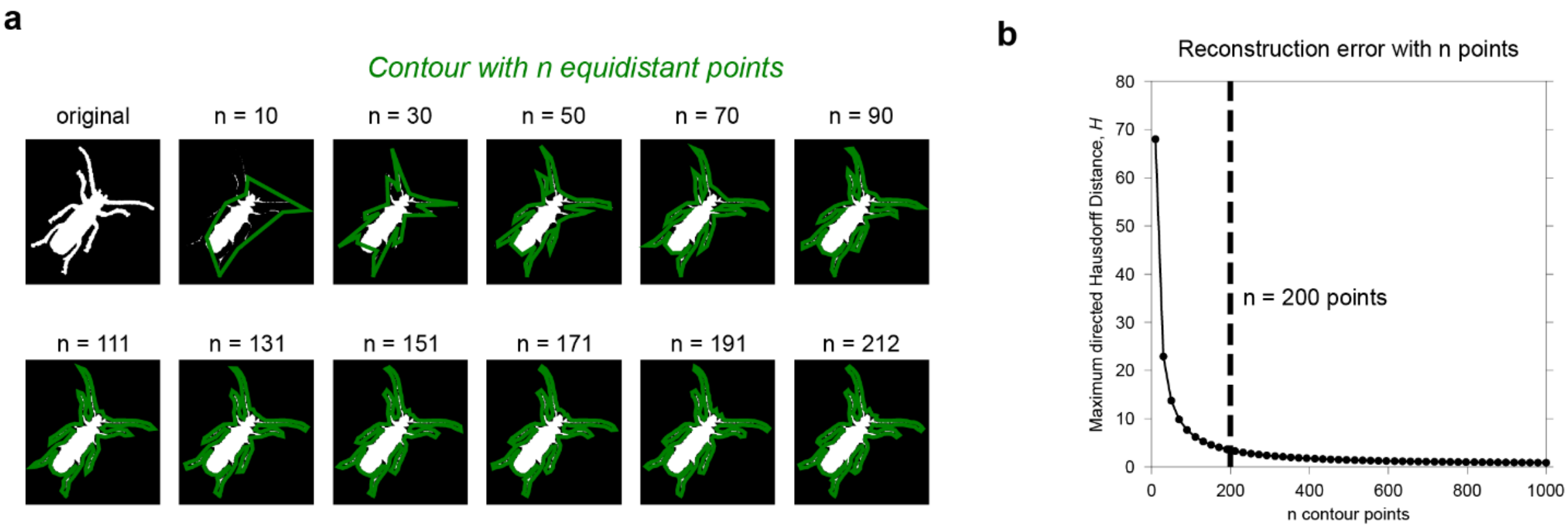

Supplementary Figure 1 of NCOMMS-25-25126. Assessment of the number of equidistantly sampled contour points to represent a shape contour. a) *Resulting contour (green) of a beetle image in the MPEG-7 dataset when we use n points equidistantly sampled from the true contour. As n increases, the more accurate the approximation.* ***b).*** *Mean of the maximum directed Hausdorff distance, H between the n-point contour approximation and the true contour over all images in the MPEG-7 dataset, as n is increased. The dashed line shows the $n = 200$ used as default in SPOT which achieves a balance between minimizing the number of points and the reconstruction error.*

We partitioned the object area into 3 regions of equal distance from its boundary, as this has natural interpretation of the border, middle and central regions of an object. Unfortunately, in order for the number of total features to be identical across for all objects, the number of partitions also needs to be applicable and computable for all objects, irrespective of their area. As such, there is little scope for optimization. Whilst further subdivision is possible, for small objects, these regions would result in smaller (and possibly zero) image area, yielding more intrinsically noisy or uninformative features. Thus we decided to maintain three regions. We also did not consider further division by angular rotation which for concave shapes or shapes of high curvature may under- or over-sample the partitioned area in particular angles. Furthermore, we would require accurate rotational alignment to ensure the derived measurements are rotation-invariant – the same object but rotated is the same object and should have the same phenome.

4) The success of the whole pipeline is dependent on accurate object tracking/tracklet. Can you quantify the accuracy of this step of your pipeline and discuss how this accuracy will affect the downstream results numerically?

a) Accurate object tracking and quantify the accuracy of the tracking pipeline:

Reply:

The tracking approach we implement is 'first detect then link'. Organoids are first detected as a bounding box on a per frame basis, then matched by solving the linear assignment problem using intersection-over-union (IoU) as the cost function. Using IoU intrinsically accounts simultaneously for size and closest distance. This linking approach is the most popular, state-of-the-art and optimally solves bipartite matching problems such as the pairing of detected organoids across two frames, e.g. also used by Fiji TrackMate software. We further extended this strategy by computing the rigid transform of the organoid between the current and next frame from the independently computed pixel optical flow. This predictive step enables us to further handle large morphological changes, and can be seen as the image-based extension of a Kalman Filter. We observed the matching between two frames to be 100% accurate. Our tracking approach is primarily used to enable us to determine whether the per frame based organoids detected are 'real' and to filter out transient objects such as floating debris. We assume that real organoids are detectable for at least 5 frames. Therefore our "detect and link" approach provides the required object tracking accuracy and specificity. Given good organoid detection in individual frames, we will have accurate pairwise matching. Then, only good individual frame organoid segmentation is needed for SAM phenome computation. To ensure sufficient detection and segmentation we trained neural networks whose performance has been additionally reported in the methods:

"We trained a YOLOv3 object detector for bounding box organoid detection with a total of 43,199 organoids extracted from 1534 images comprising label-free and fluorescent confocal microscopy. Images contained separated and cluttered overlapping organoids ranging from a few organoids per image to roughly 100. We measure a mean average precision with an intersection-over-union (IoU) cutoff of 0.25, $mAP_{25} = 0.91$ *on a test dataset of 43,145 organoids extracted from 1437 images comprising label-free and fluorescent confocal microscopy. Images contained separated and cluttered overlapping organoids ranging from a few organoids per image to roughly 100. If IoU cutoff is set to 0.50, we measure* $mAP_{25} = 0.67$.

We trained a simple attention-UNet model of 4 layers to segment individual organoid from image crops containing one organoid in isolation and spatially overlapping neighbors. The model has 8 features in layer 1 with the number of features doubling in depth as the image size is average pooled by a factor of 2. The model is trained on 542 unique cropped organoids from fluorescent confocal and label-free images resized to 64 x 64 pixels and random image augmentation. We obtain a test accuracy measured by IoU of 0.87 for a total 555 test images."

For SPOT analysis, longer-time tracking may benefit HMM model fitting, but is not critical to the Hidden Markov Model (HMM) based cluster transition analysis, as we demonstrate with our simulated organoid dataset (**Suppl. Fig. 2c iv**, NCOMMS-23-61673-C, bottom right). The mean elementwise difference of the HMM transition matrices remain low relative to the ground truth (100% of frames) as the level of track fragmentation increases (coverage fixed at 50% of frames), showing only a slight positive trend. This indicates that whether the tracks are highly fragmented (short) or not (long), disagreement with the ground truth HMM cluster transitions is consistently low. All other analyses are minimally affected (**Suppl. Fig. 2c ii,iii**, NCOMMS-23-61673-C, bottom).

b) how this accuracy will affect the downstream results numerically?

Reply:

A unique feature and advantage of SPOT is its ability to analyse downstream data that are not 100% accurately tracked. Firstly, 420 of the motion features are derived statistics from the per pixel velocity vectors computed by two-frame optical flow. Optical flow uses the video pixel intensity values directly and therefore unaffected by organoid tracking. The optical flow technique used, Farneback, has been previously demonstrated in the first author's PhD thesis to work robustly for organoids, even being utilized to segment organoids and track organoid branching (Zhou, F. (2017). Phenotyping cellular motion [PhD thesis]. University of Oxford). Likewise, the Hidden Markov Model (HMM) model depends only on consecutive frames and estimates model parameters from many organoid instances. Secondly, the pairing of organoids between successive frames is one-to-one. Only one organoid can be paired to another. Even if there is an identity switch when tracking an organoid, the switch only affects that one timepoint of tracking. SPOT additionally implements an IoU consistency check between consecutive times of a tracked organoid whereby a track is split, if the bounding box of the organoid in the next frame differs too much in overlap, to prevent obvious switching errors. In particular, SPOT will split the track into two: one before the switch and one after the switch. For example, suppose we have a video of 20 frames where cultures are very dense, and during timepoint 0–10, we track organoid 1. Then, in frame 11, there is overlap with organoid 2 such that the track of organoid 1 switches to tracking 2, and the track of organoid 2 now tracks 1. Considering the whole track, the tracking of organoid 1 and 2 is 50% accurate, however the matching of organoids from frame-to-frame is high - only the preceding frame when identities switch is incorrect. Therefore inaccurate tracking has minimal effect on SPOT. As each instance of an organoid is an independent observation in the SPOT analysis, there is only one erroneously matched timepoint for organoid 1 and 2 at timepoint 10. All other timepoints are correctly matched. Importantly, during the revision, the accuracy, effectiveness and robustness of SPOT was objectively validated using our newly created 3D organoid simulation movies, with systematic perturbations to segmentation and tracking (**new Figure 2 and related Supplementary Figures**, NCOMMS-23-61673-C).

5) The 3D organoid imaging produces z-stack 3D images. Is it too rough to simply project the z-stack to one 2D image as in line 1476? You may want to as least discuss on how to better treatment 3D images.

Reply:

We thank the reviewer for this comment and acknowledge that our current approach to project z-stack to one 2D image would be suboptimal, had we captured isotropic resolution images of organoids grown in 3D so that images contain unique 3D SAM information. However, as seen in **Suppl. Fig. 1a and 1b**, NCOMMS-23-61673-C, both our label-free and confocal microscopy z-stacks are resolution-limited. The 3D stack offers effectively only 2D information. Obtaining more z-slices with confocal microscopy might slightly improve 3D resolution but would severely affect photobleaching, drastically limiting acquisition frequency and total duration as well as potentially affecting native biological behaviour. Lightsheet microscope setups may achieve near-isotropic 3D imaging, but is currently insufficiently high-throughput and high-content for our organoid experiments. It also suffers the same issues of photobleaching. Moreover, due to scattering, it cannot capture the full organoid depth without the need to design custom, and complex multi-camera and sample holder setups, beyond the capability of our lab and the scope of this manuscript.

Instead, to assess the extent of mutual information between full 3D images and the equivalent 2D projections used by SPOT, we simulated full 3D organoid movies with defined phenotypes and analysed the 2D extended focus projection organoid scenes with SPOT. Our findings in the new Suppl. Figure 2 showed from 2D projection videos only, SAM-SPOT can still effectively discriminate simulated organoids whose movement are restricted to the 2D xy-plane from those with unrestricted 3D xyz movement, even with a replicate dataset of unrestricted 3D xyz movement as confounder.

6) Your hidden Markov model description is confusing. According to line 1642-1643, both the observed data and the hidden states are phenotype cluster labels. You should differ them.

Reply:
We apologise to the reviewer for the confusion. The Hidden Markov Model is used here to temporally smooth and compute the transition between phenotype cluster transition labels, as in Held et al We have already employed cluster number selection for identifying the phenotype clusters earlier in the workflow. We set the number of emission states to be equal to the number of hidden states.

7) Also, the entropy of the emission distribution may be related to the differentiability of your phenotype clusters, thus worthy of discussion.

We thank the reviewer for this suggestion. We have computed the entropy of the transition matrix for each state as suggested for additional experiments: validation experiments with chemotherapy drugs on organoids (**Suppl. Fig 3a,** NCOMMS-23-61673-C). Entropy quantifies the uncertainty of a cluster transitioning. Mathematically, a cluster has maximum entropy when it has equal probability of transitioning to any other cluster. We observe that high entropy clusters provide a fingerprint of the chemotherapy drug. Considering a cutoff of 0.5 nats, cluster 3 was highly entropic in the milder DMSO and Cisplatin drugs, cluster 1 for the more toxic 5FU and clusters 1,2,4, and 5 for the most toxic Docetaxel (**Suppl. Fig. 3a,** NCOMMS-23-61673-C). Importantly cluster 4 is the most toxic phenotype cluster with highest Caspase and Cytotox marker expression. Thus it seems high entropy might help in combination with mean SAM feature expression within individual phenotype clusters to identify the most informative clusters, namely those involved with the greatest number of cluster transitions, to help distinguish biologically relevant clusters versus 'noise' clusters.

8) Is this work related to other shape and motion work/patent of the first author? If yes, you shall explain the connection.

The other shape and motion work/patent of the first author is primarily captured in 5 publications:

1) Multiplexed 3D Analysis of Cell Plasticity and Immune Niches in Melanoma (*BioArxiv, 2024*)

    - Performs 3D segmentation of single cells in multiplexed tissue to help experimental researchers to identify immune interactions in patient tissue biopsies.
2) A general algorithm for consensus 3D cell segmentation from 2D segmented stacks *(BioArxiv, 2024)*
    - Describes a framework for shape representation to allow combining 2D segmented stacks from orthogonal views into a single 3D consensus segmentation
3) Surface-guided computing to analyze subcellular morphology and membrane-associated signals in 3D *(BioArxiv, 2023)*
    - Primarily concerns the unwrapping of surfaces of 3D single cells and monitoring of dynamic cellular protrusions over time.
4) Motion sensing superpixels (MOSES) is a systematic computational framework to quantify and discover cellular motion phenotypes (*eLife*, 2018)
    - Concerns the extraction motion signatures from an entire video without explicitly segmenting individual objects
5) Single-cell phenomics reveals behavioural and mechanical heterogeneities underpinning collective migration during mouse anterior patterning (*BioArxiv*, 2023)
    - Application of unwrapping and MOSES to identify distinct phenotypic behaviour of a subset of migrating cells during 3D embryogenesis.
    - This work extracts targeted shape features for analysis and analysis is guided by knowledge of the embryo geometry.

We regard these works as independent and tangential to the current work, which focuses on developing a general feature set and temporal analyses applicable for analysing all individually segmented 2D object dynamics.

9) for the phenotype-genotype coupling, since it is currently impossible to get both the phenotype and genotype of the same cell at the single cell level, what potential problem does the population-level coupling have? How will poor coupling/mis-alignment affect the study?

We completely agree with this reviewer that it is impossible to obtain the genotype-phenotype coupling at a single cell level. Although cells and organoids are highly heterogenous, similar morpho-dynamic phenotypes can be reflective of cells or organoids who share similar genotypes and with similar biological functions. The premise we take in the manuscript is that it is difficult to infer consensus patterns when considering only individual cell or organoid heterogeneity, whilst a simple average may not reveal sufficient differences. SPOT's proposal is to capture a middle ground of monitoring temporal changes i.e. the *delta* in the distribution of heterogeneity relative to the baseline at timepoint 0, over a longer time horizon than the biological process being studied. Using our stacked barplots, we can then begin to make functional inferences more reflective of the mechanistic action of a particular stimulus. Of course, any insights would then need to be supplemented with more targeted molecular assays such as that in Fig. 6. The primary motivation, however, is that SPOT could represent a relatively inexpensive way to identify those functional timepoints and phenotypes

that maximally increase the likelihood of identifying mechanistic differences. These points have now been included in the discussion.

Reviewer #1 (Remarks on code availability):

The author provided the code and README, but the code is too complicated for me to run.

We apologize and thank the reviewer for the feedback. We have created step-by-step instructions for running the Python scripts with the provided example datasets. This is included as part of the GitHub repository as a .docx word file. We further make available the new organoid dataset with chemotherapy drugs and cytotox/caspase stains used in the revision, with its corresponding analysis code for further reference. The code will be continuously maintained in GitHub. Through GitHub issues, we welcome community suggestion and reporting of bugs.

REVIEWER #2 (REMARKS TO THE AUTHOR):

This scientific paper introduces a concept in the field of imaging analysis, termed the "imaging phenome," which is analogous to the transcriptome in single-cell transcriptomics. The authors propose a comprehensive framework to describe phenotypic diversity in dynamic objects through a standardized set of image descriptors. These descriptors are categorized under three major properties: Shape, Appearance, and Motion (SAM), each of which is considered as an individual imaging "transcript."
To materialize this concept, the paper details the establishment of a standardized SAM "phenome," comprising 2185 distinct SAM features. This extensive collection of features provides a robust and comprehensive method to characterize various dynamic objects in imaging studies. Alongside this, the authors have developed a sophisticated analytical tool called SPOT (SAM Phenotype Observation Tool). SPOT is described as an imaging "sequencer," capable of analyzing both label-free and fluorescent images.

The paper underscores the potential of this approach in enhancing the understanding of phenotypic diversity through detailed and standardized imaging analysis, akin to the insights provided by transcriptomic analysis in genetics.

I've read the paper with great interest and the paper is well written and the strategy is well explained. However, I cannot advice for publication of the manuscript in its current form and the scientific work needs several improvements. Nonetheless, I think it is an interesting strategy, although applying scRNA analysis tools to single organoid image analysis is not novel (https://doi.org/10.1038/s41698-023-00480-y). But I think the approach has a lot of potential, if applied correctly.

Reply:

We thank the reviewer for recognising the value of our approach and its potential to make an important impact to the field.

1) The manuscript is way too long and lacks focus. For me, these are at least two or three independent papers. (i) Development of the SAM tool and algorithms and testing it on the public databases. (ii) applying SAM to 2D cell culture (iii) applying SAM to 3D organoid culture. For readers, it is very difficult to extract the information that is relevant for them, and these three topics mostly have a different target audience.

2) I wonder how relevant the use of the public databases from youtube and MPEG-7 is. How relevant are these features in cell biology and using this to compare with CellProfiler seems strange.

3) Would a repository such as LIVECell not be more relevant to determine is SAM can accurately cluster distinct cellular morphologies rather than irrelevant images and videos (https://www.nature.com/articles/s41592-021-01249-6)?

Reply:

We thank the Reviewer for these constructive suggestions. Our motivation for choosing computer vision datasets was to use relatable everyday examples to demonstrate the need to design multiscale spatial features that capture shape, appearance, and motion simultaneously. We also wanted to develop a single feature set or 'phenome' that is applicable to videos of any 2D object which would enable like-for-like comparison between any analysed datasets across assays, conditions and labs.

To increase manuscript focus of organoids and to enable us to present key experiments performed on published 2D datasets leading to the development of our SAM phenome and SPOT, we have developed the original submitted manuscript into two independent but complementary manuscripts: the current revised manuscript, focussing on the application of SAM phenome and SPOT analysis to analyse 3D organoids, and a new parallel manuscript submission entitled *"Development of a universal imaging "phenome" using Shape, Appearance and Motion (SAM) features and the SAM Observation Tool (SPOT)"* focussed on the details of the method development. By moving the detailed description of the SAM-phenome construction and SPOT development into the methods-focussed paper, the revised paper can focus exclusively on establishing the applicability of SAM-SPOT to identify genotype-phenotype-function coupling in high-content 3D organoid cultures using 2D projection videos (**new Figure 1,** NCOMMS-23-61673-C), as suggested by this Reviewer. We recognise the value this reviewer places on testing whether ground truth morphologies can be accurately distinguished using a relevant reference dataset. To respond to this, we created a novel organoid simulator to generate synthetic 3-dimesional organoid movies with user-prescribed evolution of their shape, appearance, and motion over time. We used the simulator to generate large datasets of 2D projection videos with complex ground truth SAM phenotypes. Our results demonstrate the effectiveness of SPOT analysis in a highly relevant, organoid-specific context (**new Fig. 2** and **new Suppl. Fig. 2,** NCOMMS-23-61673-C).

Our new methods manuscript, which we invite the Reviewers to request access to via the Editor, contains the non-organoid information with substantial expansion of the details in constructing our SAM phenome and the development of SPOT analysis framework. This paper also provides detailed validation of SAM-SPOT on computer vision and YouTube video datasets with ground truth. Additionally, following this Reviewer's valuable suggestion, we assessed the applicability of SAM-phenome and SPOT to large biological public datasets with known phenotype information, LIVECell for static images and cell division for live-cell videos as suggested by reviewers. We further compared results to using convolutional neural network (CNN)-features and CNN-LSTM (long short term memory)-features after training these AI-based models on the datasets, using 1 million fixed single cell images and >5000 single cell video frames for training, respectively. We then tested the features in the respective datasets when used instead of our SAM phenome in training phenotype classifiers and for SPOT analysis using >400,000 fixed single cell images and >7000 single cell video frames, respectively. Our SAM-phenome significantly outperformed both CNN-features or CNN-LSTM-features in both tasks, training superior phenotype classifiers to predict cell type or cell division stage, and providing a more informative and interpretable SPOT analysis, thanks to its rationally defined features and explicit categorization of those features by shape,

appearance and motion. In summary, both manuscripts comprehensively demonstrate the power, utility and compatibility of SAM-SPOT for genotype-phenotype coupling for high-content imaging-based screening.

4) My main concern is the images that are used for the organoids. SAM quantifies 2185 features, so it is very sensitive to imaging artifacts.

Reply:

Due to the limitation in space in our previous manuscript, we did not demonstrate that SPOT's design is versatile to reconcile artefacts within the analysis. In our new methods-focussed companion paper, we now clarify our design rationale in the detailed description of the construction of SAM phenome. Specifically, the 2185 SAM features is the entire SAM phenome and is the resultant number of features after quantifying the parameters of shape, appearance and motion, at global, regional and local spatial scales. The high dimensionality includes intended redundancy across features, which builds robustness that guards against experimental and processing 'noise' errors.

We draw analogy in designing the SAM phenome to single cell sequencing and analysis vs traditional microarray/select biomarker assays. A single biomarker is interpretable but often is not always expressed nor necessarily specific to the desired cell type due to biological stochasticity and measurement noise. Using a panel is the gold standard to perform cell deconvolution and cell type calling and is much more robust. Employing the same philosophy, the SPOT workflow builds on the designed redundancy within SAM features to include many processing steps to further guard against artefacts and to maximize biological discovery. Systematic and multi-stage feature selection is employed on a per experimental basis and this is done by filtering to remove potentially unreliable features that exhibit either too high a statistical variation or those that are zero-valued across all segmentations (and thus which contribute no information). Since motion (differential change over time) is one of the key parameters of SAM features, and a key advantage of live-cell imaging, a feature selection based on regression with time enables us to further identify and remove features associated with systematic errors, as imaging artefacts should not drive temporal variation in the dataset. Thus, by selecting and retaining those SAM features that exhibit temporal variation, SPOT maximizes discrimination between conditions when considering the population-averaged temporal changes or stacked barplots whilst minimizing imaging artefacts. Therefore, in reality, whilst SPOT always computes 2185 SAM features initially, automated feature selection means only a fraction is adapted and used to analyse each experiment. If we instead under-compute features, the information is irrecoverably lost in analysis. If we compute many more features, individual phenomes will be 'too unique' and also worsen performance (See reply to Reviewer 1 above). SPOT's preprocessing is analogous to the standard way in which only top-expressing transcripts are used to analyse single-cell experiments, where the full transcriptome still needs to be measured in the raw data. The advantage and the power of computing comprehensive and redundant SAM features from multiple spatial scales, the SAM phenome, versus only selected feature subsets is the basis of why SAM phenome significantly outperforms and is more broadly-applicable than purely data-driven AI-based features both in classification and in providing more informative

phenotypical measurements. To highlight this, we include below **Fig. 6** analysing the LIVECell dataset in our companion methods-focussed paper NCOMMS-25-25126).

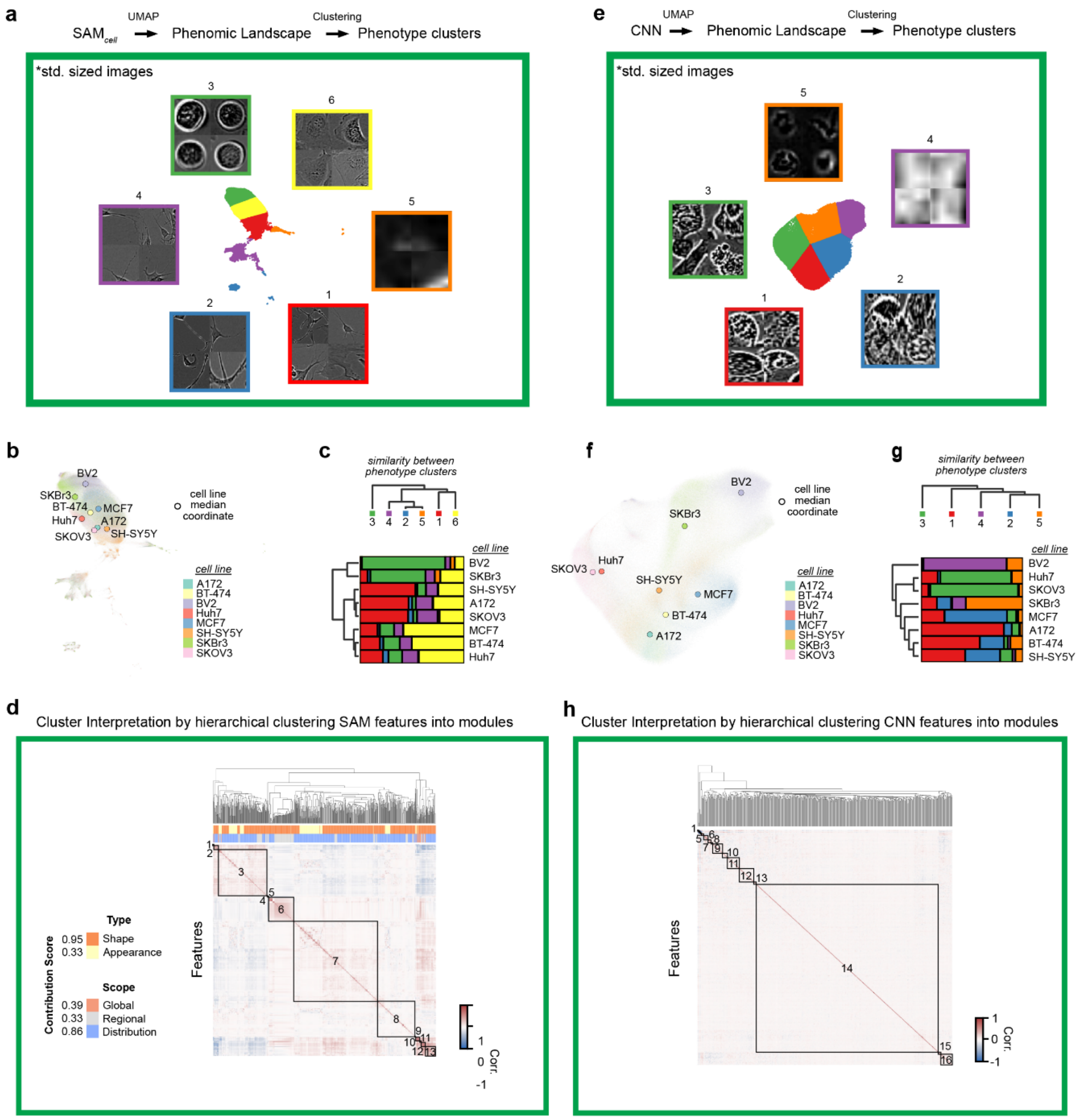

Figure 6 of NCOMMS-25-25126. SPOT characterizes phenotypic heterogeneity of different cell types in the LIVECell dataset. a) *SAM phenomic landscape constructed by applying UMAP to the SAM phenome, followed by identification of phenotype clusters by k-means clustering of 2D-UMAP coordinates using the elbow method. 2x2 image panels show exemplars of the principal phenotypes in each color-coded cluster (Methods).* ***b)*** *SAM phenomic landscape with each point (a cell), colored by its cell line. Colored circles show the median coordinate of each cell line.* ***c)*** *Hierarchical clustering using average linkage of phenotype cluster based on occurrence across the 8 cell lines (top) and of the 8 cell lines based on their phenotype cluster composition (bottom).* ***d)*** *Contribution score of shape, appearance and motion, and global, regional and distributional features in explaining the*

dataset variance defined as the absolute value of the first principal component. Automated hierarchical clustering of individual SAM features based on pairwise correlation between features to identify SAM modules (middle, modules outlined and numbered along the diagonal). ***e)*** *Phenomic landscape constructed by applying UMAP to CNN features. Cluster coloring and associated exemplar 2x2 image panels as in Fig. 5b.* ***c)*** *UMAP with each point (a cell), colored by its cell line. Colored circles show the median coordinate of each cell line.* ***g)*** *Hierarchical clustering of phenotype cluster based on occurrence across the 8 cell lines (top) and of cell line using based on their phenotype cluster composition (bottom).* ***h)*** *Clustering of individual CNN features into modules based on pairwise correlation between features using hierarchical clustering, as in d).*

The panels in the left column show that our SAM phenome finds (a) more and unique morphotypes, which allows it to find more similarity between individual cell lines (b,c) and is more interpretable, supporting the computation of shape, appearance and motion contributions to heterogeneity and the finding of distinct feature modules. In contrast, neural network derived features are less informative, notably shown by repetitive exemplar images between phenotype clusters (e), and complete lack of intra-module similarity in individual features (h).

5) Unfortunately, the imaging and culturing strategy that was applied is not compatible with SAM.
Organoids are grown in ECM domes, therefore they are not in the same plane which is a minimal requirement for this approach. There are various methods and plates available to image organoids in a single plane such as microwell plates (e.g. Gri3D) or flat-bottom 384-well ULA plates (https://www.jove.com/v/64434/multiparametric-tumor-organoid-drug-screening-using-widefield-live).

Reply:
We thank the reviewer for the careful reading and we agree with this reviewer that "*organoids are grown in ECM domes, therefore they are not in the same plane*". We used various culturing methods and plate types in this study according to the live cell imaging acquisition requirements, as presented now in new Suppl. Table 3. Regardless of culturing method, organoids lie in different planes. To reconcile this in our z-stacks (primarily 11 z-slices) which offer minimal 3D information (**Suppl. Fig. 1a, 1b,** NCOMMS-23-61673-C), we maximize the number of in focus organoids within the dome shaped matrigel in one 2D image by performing 2D extended focus projection, also called focus stacking for label-free microscopy and maximum intensity projection for fluorescent microscopy videos. The **new Suppl. Fig. 1a**, NCOMMS-23-61673-C, illustrates how in label-free microscopy images, extended focus which projects organoids in different planes to 2D based on the strength of their image gradient is better than maximum intensity projection in generating in-focussed organoids in the resulting 2D image so that we can measure biologically relevant SAM features. We have now updated our Methods and Supplementary Materials section to ensure

that all image acquisition details are included. Additionally, we also specified the tissue culture plates used in each of the experiments presented. These are summarised in the below table (new **Suppl. Table 3**, NCOMMS-23-61673-C):

Figure	Plate	Z-stack
Figure 3	Corning® Costar® TC-Treated Multiple Well Plates	Z-stack images 1000 μm in depth were acquired at 100 μm intervals (11 steps per acquisition) every hour for 5 days.
Figure 4	384-well plate, Flat bottom, rounded square wells	Z-stack images 1225 μm in depth were acquired at 35 μm intervals (35 steps per acquisition) every 2 hours for 5 days at each of the chosen positions.
Figure 5	Nunc MicroWell 96-Well Optical-Bottom Plates with Polymer Base, Cell culture, black	Z-stack images 1000 μm in depth were acquired at 100 μm intervals (11 steps per acquisition) approximately every hour for 14 days.
Supp Fig 1a	Nunc MicroWell 96-Well Optical-Bottom Plates with Polymer Base, Cell culture, black	Z-stack images 1000 μm in depth were acquired at 100 μm intervals (11 steps per acquisition) approximately every 60 min for 14 days.
Supp Fig 1b	384-well plate, Flat bottom, rounded square wells	Z-stack images 1225 μm in depth were acquired at 35 μm intervals (35 steps per acquisition) every 2 hours for 5 days at each of the chosen positions.
Supp Fig 3a.c	Nunc MicroWell 96-Well Optical-Bottom Plates with Polymer Base, Cell culture, black	Z-stack bright-field images covering 1000 μm total depth were acquired at 100 μm intervals (11 steps per acquisition) every 4 hours for 5 days.
Supp Fig 3b, Supp Fig 3c	"Plastic transparent" plates: Corning® Costar® TC-Treated Multiple Well Plates<br><br>"Glass-bottom" plates: Nunc MicroWell 96-Well Optical-Bottom Plates with Polymer Base, Cell culture, black	Z-stack bright-field images covering 1000 μm total depth were acquired at 100 μm intervals (11 steps per acquisition) every 4 hours for 5 days.
Supp Fig 3d	Corning® Costar® TC-Treated Multiple Well Plates	Z-stack images of 1000 μm in depth were acquired at 100 μm intervals (11 steps per acquisition) approximately every 15 minutes for 5 days.
Supp Fig 4a	Nunc MicroWell 96-Well Optical-Bottom Plates with Polymer Base, Cell culture, black	Z-stack bright-field images covering 1000 μm total depth were acquired at 100 μm intervals (11 steps per acquisition) every 4 hours for 5 days.
Supp Fig 5	Nunc MicroWell 96-Well Optical-Bottom Plates with Polymer Base, Cell culture, black	Z-stack images 1000 μm in depth were acquired at 100 μm intervals (11 steps per acquisition) approximately every 60 min for 14 days.

6) From image 3B it is very evident that the clusters are mainly based on imaging artefacts. For example, cluster 3 are out of focus organoids, cluster 2 are overexposed

organoids, cluster 7 are underexposed organoid, cluster 4 out of focus and underexposed… Although there might be some phenotypic differences, features extracted from these images are completely unreliable due to the imaging artefacts.

We thank the Reviewer for this comment and apologize for not being sufficiently clear in our description and rationale of 'phenotype cluster'. The term 'phenotype clusters' does not strictly refer to biologically meaningful phenotypes. A detailed description of the rationale for each step of the the analytical workflow of SPOT is now included in **Fig. 2** of our methods-focussed paper NCOMMS-25-25126, included below.

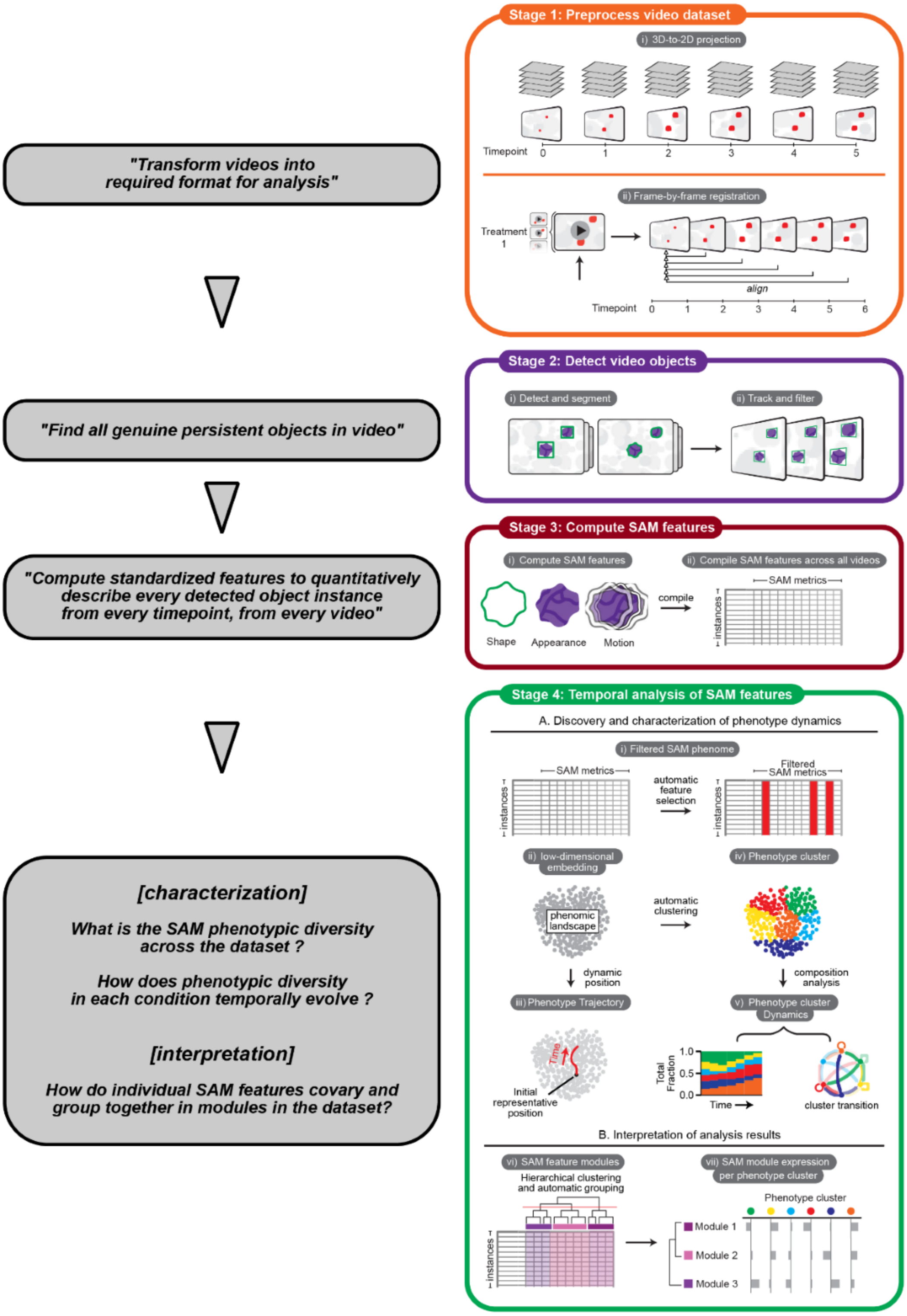

Technically, the phenotype clusters coarsely partition the UMAP phenotypic landscape such that organoid instances within each cluster share more similar instantaneous SAM features. This does not necessarily mean each cluster captures a unique biological phenotype, nor does this exclude multiple clusters describing the same biological process e.g. cell death (**Fig. 3**, NCOMMS-23-61673-C). This also means that some clusters may be attributed to a dominant image artefact e.g. blur. The phenomic landscape is a map of SAM heterogeneity across all time and conditions in a single 2D coordinate space. Whilst phenotype clusters serve as the

discrete representation of SAM heterogeneity. The primary biological analysis of SPOT is to assess how phenotype heterogeneity changes temporally, as captured by the phenotype trajectory, phenotype transition and stacked barplot analysis, to make comparative inferences about treatment conditions. In sum, SPOT makes the fundamental hypothesis that imaging artefacts are systematic, and ergodic (i.e. not time-dependent). Therefore, we expect pure imaging-artefact-only 'phenotype clusters' to exhibit minimal population changes over time, to not be the primary driver for the temporal evolution of phenotype, and to affect all conditions equally. Our additional validation experiment with chemotherapy drug, including the use of Caspase marker, show how multiple phenotype clusters (small organoids and large organoids) can correspond to cell death (**Suppl. Fig. 3a,** NCOMMS-23-61673-C). Moreover, independent SPOT analysis of organoids cultured in glass or plastic plates show the same broad classification of drug toxicity (less toxic: DMSO and Cisplatin vs more toxic: 5FU and Docetaxel), (**Suppl. Fig 3b,** NCOMMS-23-61673-C). Finally, the ability of SPOT to handle artefacts is further demonstrated using our 3D organoids simulator shown in the **new Figure 2,** NCOMMS-23-61673-C.

With respect to the specific comments about clusters shown in **Fig. 3b**, NCOMMS-23-61673-C, our interpretations of the results are listed below.

- *Cluster 3 and 4 organoids are out-of-focus:*

These organoids correspond to the same organoids that lie outside our imaged depth of 1000 µm. In the respective density plot (Fig. 3c ii), we see the primary density is located outside of the UMAP areas covered by clusters 3 and 4, and in the stacked barplot (Fig. 3c iii), we see that clusters 3 and 4 are relatively temporally constant subpopulations across time, similar across conditions and largely transiting only between themselves (Fig. 3c iv). The direction of the phenotype trajectories over time does not move towards the UMAP region covered by cluster 3 and 4 in any of the treatments, demonstrating that these 'artefact' clusters do not hinder biological interpretation (Fig. 3c v).

- *Cluster 2 organoids are overexposed:*

These organoids are in fact not overexposed: they have a transparent lumen which means that they are measured as being relatively brighter. In addition, e.g., from Fig. 3d, it can be seen that the intensity (SAM module 3) of Cluster 2 is less than Cluster 1. We know that this cluster reflects growing organoids because the stacked barplot (Fig. 3c iii) shows that its relative proportion expands over time in a stable manner, reflecting the fact that smaller organoids that were not previously classified as cluster 2 are now part of cluster 2 following their growth. We also know biologically that healthy, growing gastrointestinal organoids are transparent. They become dark and opaque with excessive cell death, leading to accumulation of cell death in the lumen. Suppl. Fig. 3a with Caspase and Cytotox verify the primary signatures of cell death as being darker and reduced movement.

- *Cluster 7 organoids are underexposed:*

In contrast to what is suggested by the Reviewer, these organoids are true phenotypes due to cell death where material is accumulated in the normally transparent lumen and the organoid shrinks/buckles in on itself (See Suppl. Fig. 3a with Caspase and Cytotox markers). We see this strongly in the phenotype trajectory following treatment with toxic drugs, which moves towards the UMAP regions covered by cluster 7 over time (Fig. 3c v). Correspondingly,

the stacked plots show a much higher end proportion of cluster 7 organoids following treatment with highly toxic drugs (Fig. 3c iii). Again, this underscores the importance of analyzing live-cell imaging and considering the temporal changes.

7) The label-free organoid segmentation is not accurate enough, and tracking of the organoids is not consistent over time (e.g. video 4) or the density of the organoids is way too high (e.g. video 5). Organoids merge together, overlap, get detected – undetected,… so there is no consistency that allows for kinetic measurements crucial for SAM. Again, this can all be resolved by the use of proper imaging-compatible culturing techniques and plates.

We thank the Reviewer for this comment and our newly generated synthetic organoid datasets with defined phenotypic behaviour have provided answers to this important criticism. In particular, we simulated the effect of increasing segmentation errors including overlap, cutout and union and tracking errors through fragmenting organoid tracks on SPOT analysis (**Suppl. Fig. 2c**, NCOMMS-23-61673-C). Our results show SPOT is robust to increasing amounts of these artefacts.

Additionally, we would like to clarify the approach in SPOT to ensuring temporal consistency and minimizing errors when generating the final organoid instances to compute SAM features: 1) we only need two frames to compute motion features; 2) organoid instances are only retained if the intersection-over-union of the segmentations frame-to-frame in the whole track is at least >0.25, which we find is an effective threshold for matching, taking into account an acceptable margin for shape changes over the elapsed hour between successive timepoints; 3) we temporally smooth segmentations over a time window which enables both temporal consistency and imputation of partial segmentations; 4) except for the centroid displacement features (only two features), the remaining motion features are based on the pixel-dense motion field, where every pixel of an image has an associated motion vector as computed independently with optical flow techniques. Importantly, optical flow does not depend on segmentation and its error can be constrained by incorporating physical constraints such as ensuring smoothness of motion field and constraining the size of the displacements; 5) the principle of SPOT to monitor the relative temporal change i.e., the delta in the measured heterogeneity over time across the whole population between different conditions. This is shown by our choice of clustering the phenotype trajectories using the dynamic time warping metric to compare trajectory shape instead of using absolute positional coordinates in the UMAP phenotypic landscape. Even if individual segmentation is inaccurate and noisy, as long as these errors are systematic over time, do not exhibit temporal variation, and average out over the population, we expect SPOT to detect relevant biological phenotypes. This is shown by our ability to resolve the effect of WNT addition, despite the organoid density, as independently validated by scRNA-seq (**Fig. 5 and 6**, NCOMMS-23-61673-C).

We would like to emphasize that the WNT biology that we are interested in is dependent on local organoid density. Moreover, the organoid flattening phenotype we observe is not local and covers spatial regions much larger than stereotypical spherical organoids. Here, the concept of organoid detection and segmentation does not apply and Particle Image Velocimetry (PIV) type of approaches are more applicable. Yet, SPOT is still able to resolve the phenotypes because we include comprehensive and redundant SAM measurements. If SPOT

were only applicable to spatially delineated organoid cultures and relied upon accurate segmentation and tracking then it would have limited application to discovering novel phenotypes. It is important to note that tissues are densely packed with cells with extensive cell/cell interactions, therefore certain key biological information will be lost in uniformly formed isolated organoids.

8) I'm wondering how much features are actually relevant. For example, for figure 3, studying the drug response in the organoids, the same conclusion can be drawn with just basic imaging features and do not require quantification of 2184 features. So for basic drug screening I think this is overkill.

We thank the Reviewer for this comment. We agree that for a basic toxicity screen, the number of features is overabundant. However, a central premise in our manuscript is whether we can compute a universal set of SAM features in the same universal manner that is equally applicable for analyzing simple (cell death) and complex scenarios (changes in organoid branching patterns). Whilst specifying a few targeted measurements such as biomarkers is simpler and more direct , it is not robust and interpretation may further be confounded by conflicting results. In **Suppl. Fig. 3a**, NCOMMS-23-61673-C, both Caspase and Cytotox are biomarkers of cell death yet they do not fully agree with each other. We also found we had to count the number of punctate spots instead of taking the mean intensity to get an interpretable quantification. For high-content and high-throughput applications, specifying more features than performing feature selection has proven to be more discriminative for discovery. For example, agnostic sequencing of all available transcripts is essential for scRNAseq to discover new cell types and corresponding biomarkers compared to classical microarrays.

9) In addition, the authors make statements about that certain clusters correlate with for example high levels of cell death (line 575) but no evidence of cell death is provided (e.g. fluorescent cell death marker). So I understand unbiased clustering of features is interesting, but it would be more interesting if they can be linked **to processes such as viability, cell death, apoptosis,… by using corresponding fluorescent markers.** ***This way you can determine SAM features related to specific biological processes and give meaning to a cluster (similar to linking scRNA seq clusters to biological pathways based on genesets).***

To demonstrate that SPOT phenotypic clusters can be associated to a biological process, we have performed the Reviewer's suggested additional staining experiments using caspase and cytotox to check the cell viability, cell death and cell apoptosis in the chemotherapy drug screen (**new Supp. Fig 3a, 3b, 3c,** NCOMMS-23-61673-C).

Our initial SPOT analysis suggested 3 toxicity groups (Fig. 3). We therefore selected the control DMSO and Cisplatin (6 µM and 100 µM) to represent the low-toxicity group, 5-Fluorouacil (5FU) (20 µM) for the mild toxicity group, and Docetaxel (12.5 nM) for the high-toxicity group. The concentrations chosen are the maximum level concentration used in Fig. 3. The organoids were grown for 6 days in a 96-well plate. The medium was changed with HOM and a titration

of the drugs and the dyes as outlined below. Z-stack bright-field images and confocal images 1000 µm in-depth were acquired at 100 µm intervals (11 steps per acquisition) approximately every 4 hours for 5 days. Previously images were acquired every 1 hour for 5 days. The slower acquisition was used to reduce phototoxicity and to account for the additional imaging due to increased channels.

1. The cell viability during the drug screening was tested with Hoechst 33,342 (50 nM/well, ThermoFisher) consistent with literature (*OrBITS: label-free and time-lapse monitoring of patient derived organoids for advanced drug screening. Cellular Oncology. 2022*);
2. Real-time quantification of cell death induced by the toxicity of the drugs was assessed with **Cytotox Green** (60 nM/well, Sartorius, #4633), (*Multiparametric Tumor Organoid Drug Screening Using Widefield Live-Cell Imaging for Bulk and Single-Organoid Analysis. J. Vis. Exp. (190), e64434, doi:10.3791/64434 (2022))* and the cell death was calculated based on the number of detected punctate spots within each segmented organoid;
3. The evaluation of cell apoptosis during the drug screening was assessed with **Caspase 3/7 Green** (2.5 µM/well, Sartorius, #4440), (*OrBITS: label-free and time-lapse monitoring of patient derived organoids for advanced drug screening. Cellular Oncology. (2022); Viability Analysis and High-Content Live-Cell Imaging for Drug Testing in Prostate Cancer Xenograft-Derived Organoids. Cell. 2023)* by counting the number of detected punctate spots within each segmented organoid.

We performed SPOT analysis with the acquired datasets using our proposed SAM phenome only applied to the brightfield microscopy channels, then using the fluorescent Cytotox and Caspase biomarkers afterwards for interpretation. Our results, shown in the **new Suppl. Figure 3a**, NCOMMS-23-61673-C, show that SPOT identified 7 phenotype clusters of which clusters 2, 4, and 7 correlated with higher toxicity, with elevated number of measured Caspase and cytotox punctate spots. Interpreting with SAM modules, these clusters correspond two different death phenotypes: the normal accumulation of cell death in the lumen as organoids grow too large (clusters 2 and 4) and the drug-induced cell death whereby organoids remain small or potentially already dead (cluster 7). We found the total number of spots within a segmented organoid (see Methods) was a more reliable and informative measurement than green area/brightfield area due to the nonspecific auto fluorescence. Toxicity was measured highest for Docetaxel, which we had predicted by SPOT with SAM signature of low intensity and reduced movement. Moreover, our UMAP organized both Caspase and Cytotox markers similarly and in a gradient manner. Subsequently, hierarchical clustering of phenotype trajectory showed greater similarity of cisplatin to DMSO, then 5-FU, with Docetaxel most different, consistent with our findings of the larger screen with multiple drug concentrations in Fig. 3. Altogether our analysis demonstrates how SPOT uses multidimensional features and temporal change to robustly stratify toxicity phenotypes compared to using select single/few biomarkers which may itself be subject to experimental variation, and how they are used to extract measurements.

10) The authors use the term 'flattened organoid morphology' (line 765). It is difficult to see from the image, but are these cells that adhere to the plastic surface and start growing in 2D? This might be evidence of a more invasive, migratory behaviour.

We performed fixed staining at the endpoint of APC, KRAS, and p53 mutant mouse colon organoid movies with confocal z-stacks to assess and verify the observed organoid morphology from 2D projection. Using depth coloured maximum intensity projection (DCP), **Suppl. Fig. 4b,** NCOMMS-23-61673-C, shows the morphological transformation in the APC mutant localizes preferentially at the interface of matrigel/substrate or boundaries of the matrigel ECM dome (red and blue coloured organoids in DCP). We agree with the Reviewer that this may reflect a more invasive, migratory behaviour. Both p53 and KRAS mutants are more spherical and lie more centrally in the ECM dome (yellow/green coloured in the DCP).

11) While I find that the current version of the manuscript does not meet the necessary criteria for publication in its present form, I would like to emphasize the valuable insights and potential contributions your work offers. The breadth of the topics covered is commendable, yet a more focused approach could enhance the clarity and impact of your research. I strongly encourage you to consider refining the scope of your paper <u>or split up the paper into two or three papers, sharpening the central arguments, and addressing the highlighted issues</u>. A revised version that addresses these aspects would be a welcome submission to this journal. Your efforts in advancing research in this field are appreciated, and I look forward to potentially seeing a revised manuscript, in particular with a focus to organoids. I would like to express openness to potential collaboration. In my current research, we have developed an extensive library of organoid images treated with a variety of therapies. If you find it relevant, I believe a collaborative effort could enrich both our research endeavors, leveraging our respective resources and expertise.

Yours sincerely,
Prof. dr. Christophe Deben

We would like to thank the Reviewer again for recognising the value of our work and its potential to advance the field. We would also like to thank them for their constructive comments, which we have aimed to address here, and which have substantially improved the clarity of our manuscript. Once the manuscript is published, we will be delighted to collaborate with the Reviewer.

Reviewer #3 (Remarks to the Author):

1) The paper describes an extension to the toolbox of researchers that appears to outperform the established standard, namely CellProfiler. The authors argue that the introduction of motion in their pipeline, and a determination between global, local-regional and pattern-pixel levels are key differentiators for success. The authors support this statement well in text and figures, namely through the reporting of contribution scores for their clustering interpretation.

We thank the reviewer for their positive comments.

2) The validation work of the developed tool, SPOT, also revealed a potential new insight into the working of the WNT-pathway. The authors demonstrated that for two contradicting configurations, WNT containing mouse organoids and WNT deprived human organoids, the observed morphological behavior to have a similar underlying cause. The authors support this with single-cell-sequencing and investigated phototoxicity as a potential cause of these events. I believe this to be a new finding, with a clear and soundly executed methodology.

We thank the reviewer for highlighting the robustness of our methodology and the novelty of our approach.

3) The currently proposed tool, SPOT, has been validated on multiple 2-dimensional datasets. The authors do show the potential of spot for organoid segmentation from Matrigel droplets, a 3-dimensional environment where organoids significantly overlap in the image. It is however currently unclear whether SPOT will work well for 3-dimensional time-series data. A possible complication can be that the motion module in SPOT can confuse X/Z movement for a phenotypic response of cells.

To test the applicability of SPOT analysis of 2D projections to characterize 3D organoid dynamics, we generated simulated organoid Dataset B. Organoids with prescribed shape, appearance and motion 3D dynamics were individually simulated and positioned in 3D scenes. The 3D scenes were then projected as 2D videos for SPOT analysis. We simulated three different 3D motion scenarios: the first condition is of full unrestricted 3D XYZ movement; the second condition restricts movement to 2D XY planar movements only but with the same speed as the first condition; the final third condition is a replicate of full unrestricted 3D XYZ movement with different simulated organoids. We find that whilst visually the phenotype trajectory and stacked barplots of phenotype cluster frequency over time appear indistinguishable, SPOT nevertheless detected differences and reliably clusters the XYZ movement conditions distinctly from the XY planar movement condition, (**Suppl. Fig. 2d,** NCOMMS-23-61673-C). We thus conclude the ability of SPOT to reliably differentiate the subtle visually undetectable Z movement demonstrates the suitability of SPOT for 3D time series analysis from 2D projection videos.

4) Overall, the paper is very well written, providing a new toolbox to researchers that has the potential to become of significant value to the field.

We thank the reviewer for their comments and for highlighting the value that our technology will bring to the field.

NCOMMS-23-61673-C (Revised manuscript, organoid-focussed application of SAM-SPOT)		
**Current Figure**	**Previous Figure (NCOMMS-23-61673-T first submission)**	**Description**
Figure 1	New	Comprehensive shape, appearance and motion (SAM) characterization enables analysis of phenotypic heterogeneity in organoid models.
Figure 2	New	SPOT analysis of synthetic organoid video dataset with prescribed shape, appearance and motion dynamics.
Figure 3	Figure 3	SPOT phenotype analysis of human patient-derived duodenum organoids drug response enables clustering of drugs by pharmacological action.
Figure 4	Figure 4	SPOT identifies unique phenotypic differences in genotype defined murine colon organoids.
Figure 5	Figure 5	SPOT captures the timing of WNT signaling-induced phenotypic dynamics in patient-derived duodenal organoids.
Figure 6	Figure 6	SPOT complements molecular sequencing for scientific and biomedical discovery
Suppl Figure 1a	New	Brightfield phase contrast imaging has limited depth resolution and extended focus projection of a z-stack to a 2D image
Suppl Figure 1b	New	Acquired confocal z-stacks have depth-limited resolution.
Suppl Figure 2a	New	SPOT Phenotype cluster transition and density maps of the simulated organoid dynamics dataset A.
Suppl Figure 2b	New	Robustness of SPOT analysis to imaging artefact, segmentation and tracking errors
Suppl Figure 2c	New	Quantitative assessment of SPOT analysis robustness to imaging artefact, segmentation and tracking errors
Suppl Figure 2d	New	SPOT analysis of 2D projections discriminates z-component motion.
Suppl Figure 3a	New	SPOT phenotype clusters capture different SAM characteristics of cell death after drug treatment.
Suppl Figure 3b	New	Independent SPOT analysis of organoid culture in plastic transparent and optical-bottom plates

Suppl Figure 3c	New	Joint UMAP analysis of chemotherapy treated patient-derived organoids cultured in plastic and glass-bottom plates
Suppl Figure 3d	Extended Fig. 3	SPOT characterizes changes in murine small intestine organoid branching dynamics when treated with HDAC inhibitors.
Suppl Figure 4a	Extended Fig. 4	SPOT draws the same conclusions with label-free imaging of genotype defined murine colon organoids.
Suppl Figure 4b	New	Murine colon organoids exhibit flattened morphologies preferentially at boundary of ECM dome
Suppl Figure 5	Extended Fig. 5	SPOT detects changes in WNT signaling-induced phenotypic dynamics in patient-derived duodenum organoids

NCOMMS-25-25126 (Companion Methods Development Focussed Manuscript)		
**Current Figure**	**Previous Figure (NCOMMS-23-61673-T first submission)**	**Description**
Figure 1	Figure 1a, b	Shape, appearance and motion phenome characterize imaging phenotypes
Figure 2	Figure 1d	Development of SPOT
Figure 3	Extended Figure 1a-g	Testing Shape, appearance features on public computer vision datasets
Figure 4	Extended Figure 1h-j; New	Testing combined SAM on A2D YouTube dataset
Figure 5	Figure 2	Single cell tracking challenge migration dataset
Figure 6	New	SAM-SPOT and CNN-SPOT analysis of LIVECell dataset
Figure 7	New	SAM-SPOT and CNN-LSTM-SPOT analysis of single cell division dataset (HeLa histone H2B)
Suppl Figure 1	New	Assessing the number of contour points required for shape representation
Suppl Figure 2a	New	Schematic illustrating SPOT module analysis in analogy with scRNA-seq analysis
Suppl Figure 2b	New	Schematic of SPOT module analysis and computation of SAM/scope contribution scores
Suppl Figure 3	Suppl Figure 1	Comparison of SAM and Cellprofiler features to computer vision datasets
Suppl Figure 4a	Suppl Figure 2	A2D dataset similarity graph of joint object-movement classes
Suppl Figure 4b	Suppl Figure 3	A2D dataset transformed into 2D, applying different dimensionality techniques to SAM phenome
Suppl Figure 5	Extended Figure 2	Single cell division dataset, Cell tracking challenge
Suppl Fig 6	New	SAM-SPOT and CNN-SPOT analysis of LIVECell dataset
Suppl Fig 7	New	UMAP density heatmaps of SAM-SPOT and CNN-SPOT analysis of LIVECell dataset
Suppl Fig 8	New	SAM-SPOT and CNN-LSTM-SPOT analysis of single cell division dataset (HeLa histone H2B)
Suppl Fig 9	New	UMAP density heatmaps of SAM-SPOT and CNN-LSTM-SPOT analysis of single cell division dataset (HeLa histone H2B)

POINT-BY-POINT REPLY TO REVIEWERS:

Note to reviewers: as our revisions regarding the robustness and reproducibility of SPOT applied to chemotherapy screens generated a substantial number of additional figures, which may obscure clarity and distract from the main narrative, we have taken the decision to aggregate these into a standalone "Reviewer Supplementary Note item (containing Suppl. Fig. 3a-s)" for this rebuttal. Henceforth, we refer to the figures in this document prefixed with "Review Suppl. Fig.". However, we believe the figures contain many useful insights and validation to the interested reader using our software and hope the document can be officially published as a Supplementary Note item after acceptance. Throughout these documents, newly added text is shown in yellow highlight.

Reviewer #1 (Remarks to the Author):

*My previous comments are mostly addressed to the extend that the authors could do, except for the **HMM** one.*

Here is my previous comment: Your hidden Markov model description is confusing. According to line 1642-1643, both the observed data and the hidden states are phenotype cluster labels. You should differ them.

Here is authors' reply:
We apologise to the reviewer for the confusion. The Hidden Markov Model is used here to temporally smooth and compute the transition between phenotype cluster transition labels, as in Held et al We have already employed cluster number selection for identifying the phenotype clusters earlier in the workflow. We set the number of emission states to be equal to the number of hidden states.

So, indeed, both the observed data and the hidden states are phenotype cluster labels?

Author's reply:

Yes, both observed data and the hidden states are phenotype cluster labels.

What is the scientific point of smoothing the first-stage clustering result?

Author's reply:

We expect cell and organoid state transitions to be smooth. We had originally planned to smooth any phenotype cluster transitions that were short-lived e.g. 1,1,1,1,2,1,2,1,1,1,1, which might be attributed to errors in segmentation, and/or the UMAP not enforcing temporally continuous embedding. However, we recognise that a HMM might not be the best method for this, and simpler methods might be more suitable e.g. a sliding window majority vote filter. We have implemented the sliding window majority vote filter in our code library for user convenience, but did not use it in our analyses in the two papers.

If you think the cluster label shall be spatially clustered, you may want to build this spatial clustering mechanism into the first-stage clustering to increase statistical power.

Author's reply:

In our design of SPOT, to be applicable to variable length datasets we did not want to depend on temporal information when computing the phenotypic landscape and did not want to enforce spatial clustering in the UMAP because of temporal closeness. However, this is an aspect that can be investigated further in future work to improve the accuracy of the next timepoint transition analysis. We now directly estimate the next

timepoint phenotype cluster transition rates, $\Pr(\text{cluster}(t+1) = j \mid \text{cluster}(t) = i)$ by tabulating the observed frequency of cluster *j* succeeding cluster *i* over all the tracked single object trajectory phenotype cluster labels in a condition (See revised Methods in our accompanying methods paper **NCOMMS-25-25126A**).

$$\Pr(\text{cluster}(t+1) = j \mid \text{cluster}(t) = i) = \frac{(\#\text{cluster}(t+1) = j | \text{cluster}(t) = i)}{\Sigma_j (\#\text{cluster}(t+1) = j | \text{cluster}(t) = i)}$$

Reviewer #1 (Remarks on code availability):

I did not run it successfully.

Author's reply:

We retested the code and found:

1) The code dependencies were not compatible with Python >= 3.11. We have updated the readme, and also encoded the constraints in the pyproject.toml setup file
2) We have created a step-by-step install video with annotations to help users get setup and running the example scripts. The video is available at this link, and we have highlighted this in the code repository at https://github.com/fyz11/SPOT.

Reviewer #2 (Remarks to the Author):

I would like to thank the authors for their extensive work and for splitting the manuscript into two focused papers. This separation has certainly improved the clarity and readability of each individual contribution. However, I must note that several of my core concerns from the initial review have not been fully addressed—particularly regarding the robustness and reproducibility of the SPOT framework, and the added value of this complex phenotypic analysis compared to more conventional, well-established approaches in the field of drug screening.

The authors present three central assumptions to support their handling of imaging artefacts:
1. Each cluster does not necessarily represent a distinct biological phenotype, and multiple clusters may correspond to the same underlying phenotype.
2. Only clusters with true biological dynamics are expected to show differential temporal evolution.
3. Artefacts are assumed to be temporally static and affect all experimental conditions equally.

While I understand the rationale and agree that temporal dynamics can help differentiate artefactual from biological changes, I find the assumptions overly optimistic. In real-world time-lapse imaging—especially with 3D organoids—artefacts can indeed change over time. Organoids may move in and out of focus, shift between lighting gradients, or become partially occluded, leading to non-biological temporal variation in the SAM features.

Although the inclusion of simulated datasets is a useful first step, they do not sufficiently capture the complexity and variability of real experimental data, particularly in organoid imaging where segmentation and intensity artefacts are common and often heterogeneous.

I think the influence of the artefacts depends on the level of information you want to get out the analysis. In

this context, it was not clear to me what the author mean with 'SPOT analysis generates the same relative drug toxicity despite differences in measured SAM (Supp.Fig. 3b) in line 405, when comparing plastic and optical-bottom plates. Is this referring specifically to the classification into low/mild/high categories, or something else? Clarifying this explicitly would help avoid confusion.

Author's reply:

Firstly, we thank the reviewer for recognising the improved clarity of our divided manuscripts. We apologise for the confusion with regard to the toxicity classification: yes, we do indeed mean that the analysis finds the same relative toxicity classification of drugs i.e., low/mid/high. Of course, the hierarchical clustering on the subset of drugs: DMSO, cisplatin, 5FU and docetaxel which we subjected to caspase and cytotox dye will not be the same as their clustering in the full drug set in Fig. 3. To illustrate this, we sub-grouped (subsetted) DMSO, Cisplatin, 5FU and Docetaxel and reclustered according to their phenotype trajectories. The results show that DMSO and 6 µM Cisplatin form a low-toxicity cluster, and 5FU and Docetaxel a high-toxicity cluster (Reviewer Suppl. Fig. 3a). In both the caspase/cytotox-dye and label-free experiments, we should expect to find this same general clustering with 100 µM Cisplatin being an unknown condition to be classified. We have amended the text to clarify the statement that SPOT analysis finds the same relative toxicity classification of drugs.

The comparison between optical-bottom and standard plastic plates demonstrates substantial variability:
• The number of clusters differs (8 vs. 7).
• Cluster distributions and density "hotspots" vary significantly between plate types for the same drug (e.g., 100 µM cisplatin). Supp. Fig. 3b b (ii)
• The final timepoint cluster compositions differ noticeably, despite being the same treatment condition. Supp. Fig. 3b b (iii)

So while their 'relative drug toxicity' might classify the same (low and higher toxicity), and phenotype trajectory similarity is also clustered the same, these are very high level outputs which could also be achieved which much simpler approaches. It would be more useful to have a direct comparison of the underlying SAM modules that contribute to specific phenotype clusters. Since differences in plates should be static and non-temporal, the SAM modules and features should be consistent between both plates if I'm following the logic of the authors correctly.

Author's reply:

We thank the reviewer for the comment.

The static and non-temporal refer to the criteria applied to organoids within a well to exclude debris or artefact. We still expect plate-to-plate variation or 'batch' differences. The SAM modules and features will only be identical between two plates or two experiments, if the organoids from both have the same statistical distribution in every SAM feature of the phenome.

To illustrate how individual SPOT outputs are affected by "batch" differences including different culturing conditions, we have analysed technical and biological replicates of the empirical data with caspase/cytotox staining. Specifically, we compared the different independent SPOT analyses between separate 'batch' experiments, using also methods to align the different UMAP phenomics landscapes to match clusters and SAM modules (phenotype clusters, SAM expression, transition matrices, phenotype trajectories, SAM modules):

a) Patient B with and without background subtracted videos;
b) Patient B vs Patient C (which was unfortunately also more unfocused);
c) Plastic vs glass plated organoids from Patient B

Our results showed that:

a) **Patient B with and without background subtracted videos**: the background had minimal impact on the analysis, (Reviewer Suppl. Fig. 3b-e). We find an identical number of phenotype clusters whose SAM characteristics are very similar. Almost the same number of SAM modules were also detected and they all had similar expression in each phenotype cluster. The background does however result in a rotated version of the UMAP (Reviewer Suppl. Fig. 3d b).

b) **Patient B vs Patient C, which was unfortunately also more defocussed**: this comparison also showed high similarity but less than in a), (Reviewer Suppl. Fig. 3b,h-j). The UMAP required rotation and non-linear warping to align. We find the same number of phenotype clusters, but these show slightly less similarity in SAM expression and cluster transitions. The SAM module expression in each phenotype cluster were also similar.

c) **Plastic vs glass plated organoids from Patient B**: this comparison showed the strongest difference (Reviewer Suppl. Fig. 3l-n). The number of phenotype clusters differed by one. Yet, the percentage of shared features were comparable to b) and for the matched 6 out of 7 phenotype clusters, we find similar mean SAM expression, and conserved SAM modules with similar expression patterns across clusters. However, there were more differences between the cluster transitions (Reviewer Suppl. Fig. 3m). This appears to be due to differences in the local arrangement of SAM characteristics in the glass-bottom vs plastic-bottom generated UMAPs (Reviewer Suppl. Fig. 3o).

As further evidence that the discrepancy in the UMAP reflects the extent of batch difference, we showed that independent SPOT analysis of the same murine branching intestinal organoids dataset with the default segmentation (new Suppl. Fig. 3b) and with improved individual organoid segmentation (new Suppl. Fig. 3c) corroborate. Consequently, we recover the same number of phenotype clusters, which have similar SAM feature and SAM module expression, and inter-cluster transitions after UMAP realignment (new Suppl. Fig. 3d).

It would also be interesting to see how the conditions cluster when the data of both plates (and importantly independent biological replicates) is pooled together. If the method is robust and replicatable, I would assume that treatment conditions would cluster together between the two plates (or replicates).

So I would like to hear the authors opinion on this matter, and if they share the opinion that similar SAM modules/features should underly the different clusters identified in these two different plates since plate differences should be excluded according to their hypothesis.

In addition, I would love to see how robust this approach is for biological replicates, but I understand that this might be difficult to achieve. Maybe the technical replicates could be analysed seperately to detemine is the same SPOT outcome (clusters and underlying SAM modules and features) are extracted from the replicates or another drug screening dataset could be used.

So I would like to hear the authors opinion on this matter, and if they share the opinion that similar SAM modules/features should underly the different clusters identified in these two different plates since plate differences should be excluded according to their hypothesis.

Author's reply:

We agree with the reviewer that if the SPOT method is robust, reproducible and able to extract representative features, then the phenotypical clusters generated from joint SPOT analysis should be similar to those generated from independent technical and/or biological replicates. However, since shape, appearance and motion are characteristics affected by 'batch effects' e.g. if organoids don't grow as well in the replicate, or if the acquisition is more un-focussed, we expect to find less similar phenotypical clusters and/or SAM modules, when batch effects are greater. As with scRNAseq, batch effects still need to accounted for externally in analysis or using more controlled image acquisition assays.

To show batch effects, in this revision, we performed independent SPOT analysis of the same experiment and its replicate, then compared the SPOT analysis side-by-side after aligning the features/UMAP landscape/phenotype clusters/SAM modules:

1. Reviewer Suppl. Fig 3d: same chemotherapy screen with same patient (Patient B) in glass plates, with and without video background subtraction. This is the minimal perturbation we performed. We detected almost the same number of organoid instances and recover almost all the same phenotype clusters and SAM modules. Importantly, the phenotype clusters and SAM modules exhibit similar SAM characteristics.

2. New Suppl. Fig. 3d: same murine intestinal branching organoid but re-segmented with an improved algorithm. Now, the segmented shapes are not identical, and consequently the UMAP could not be aligned with a simple rotation operation. Nevertheless, even under these conditions, we recover the same number of phenotype clusters and a majority of SAM modules with similar expression patterns across phenotype clusters.

3. Reviewer Suppl. Fig. 3i: same chemotherapy screen with different patients in glass plates. Unfortunately, the videos of the second patient, Patient C, were more defocussed. Therefore, the SAM difference is likely larger than anticipated. Despite all these unexpected variations, we recover a majority of the same phenotype clusters and SAM modules.

4. Reviewer Suppl. Fig. 3m: same chemotherapy screen with same patient but using glass and plastic bottomed plates. As shown previously and noted by this reviewer, we observed the largest systematic differences between these conditions. Even though the total number of organoid instances detected were similar between the experiments, we detect one less cluster in glass-bottomed plates. Within the matched phenotype clusters, the SAM characteristics were similar, but had different inter-cluster transitions. Despite detecting an overall low number of SAM modules in plastic-bottom plates, we do identify conserved SAM modules which show similar expression pattern across the matched phenotype clusters.

The analysis of our experiments such as glass-bottom vs plastic-bottom generally pools several plates together to increase the organoid numbers (see new Suppl. Table 3 breaking down the plates comprising each chemotherapy SPOT analysis). To investigate the extent of plate-to-plate differences comparably between individual plates and individual conditions across all chemotherapy experiments, we also performed a joint UMAP analysis, pooling all the data from biological and technical replicates of the chemotherapy screen from Patient B and C (i.e. (a)–(c) in the points outlined in the prior response) (Reviewer Suppl. Fig. 3p). We find plate-to-plate variation to be larger than systematic differences like glass vs plastic, and Patient B vs Patient C. Nevertheless, hierarchical clustering of the pooled phenotypic trajectories across plates clustered drug conditions in the same relational manner (i.e. DMSO, 6uM Cisplatin, 100uM Cisplatin: less toxic, and 5FU and Docetaxel: more toxic) as those found in the SPOT analysis of individual experiments (subset grouping of plates). However, the batch effects mean that individual plates are intercorrelated, and do not represent

independent, and identically distributed (i.i.d) statistical samples. This would affect recovery of the same SAM clusters and modules, as can be seen by the misalignment in hotspot distribution in the UMAP density heatmaps of each drug between plates. We tested whether we could remove a significant proportion of the batch effects across plates by performing per-plate SAM feature normalization. We find significantly increased concordance between the UMAP density heatmaps of each drug between plates (Reviewer Suppl. Fig. 3r), and significantly reduced inter-correlation among plates (Reviewer Suppl. Fig. 3s b). However, it did not alter the relative drug toxicity (Reviewer Suppl. Fig. 3s a). This demonstrates that our SAM phenome supports the use of batch-correction techniques in a similar manner to scRNAseq, and that users have the potential to use more sophisticated tools developed in scRNAseq like Harmony to correct for batch effect prior to SPOT analysis. In our two papers, we currently do not perform batch effect correction as default for computational speed, and due to the fact that it does not substantially alter the clustering of treatment conditions. Another consideration is that per plate, the number of organoid instances is relatively small, which means that strong batch effect correction may introduce more artefacts in addition to slowing down computation.

In summary, I am convinced by the technical sophistication of the SAM and SPOT framework. However, I remain unconvinced about the robustness of the approach for images with a lot of artefacts as used in this manuscript, as ***the authors do not provide replicate datasets*** ***of the same experiment*** *(unless I missed it).*

Author's reply:

We apologize for not explicitly clarifying the plate replication. We have now tabulated a new detailed Suppl. Table 3 to outline clearly the technical and biological replicate plates and how they compose the different experiment datasets, and respective figures. This is particularly relevant for the chemotherapy screen analyses where we have further performed a new comprehensive global UMAP analysis integrating all plates subject to the same drug conditions, and dissect the SAM behaviour per plate.

It would be critical to demonstrate whether the same number of phenotype clusters—and, importantly, the same underlying SAM modules and features—are consistently identified across independent replicates or between the different plates. This would substantiate the claim that imaging artefacts are effectively accounted for or excluded because they are static, which is not fully supported by the current comparison between plastic and optical-bottom plates. Since the authours make the comparison with scRNAseq, the clustering is a first step. Afterwards we would dig deaper into the features that make up this cluster so it is important that they are consistent.

Author's reply:

We have performed these more detailed comparisons of SPOT analysis outputs in paired replicate experiments as suggested by the reviewer (Reviewer Suppl. Fig. 3d: same patient, with/without background subtraction; Reviewer Suppl. Fig. 3i: different patients; Reviewer Suppl. Fig. 3m: same patient, glass vs plastic bottom plates). We successfully found similar intermediate outputs: SAM features, phenotype clusters, cluster transitions, SAM module expression patterns etc. in the repeat experiments. The extent of similarity found correlates with the 'batch effect difference'. In the case of glass vs plastic bottomed plates, we observed the largest differences and found a different number of phenotype clusters, and the smallest number of similar SAM modules. We also performed pooled SPOT analysis across all plates and showed that the extent of similarity can be significantly enhanced, by applying per plate batch effect correction, in this case, by per plate feature normalization (see also replies to previous comments).

Moreover, the added value of this complex high-dimensional approach compared to standard kinetic measurements of cytostatic and cytotoxic responses remains unclear. The classification into low, mild, and

high toxicity could likely be achieved with simpler and more interpretable metrics. While the clustering of 5-FU and gefitinib presents an intriguing finding, its biological significance is difficult to interpret given their distinct mechanisms of action and lack of biological explanation.

*Overall, the approach shows promise, but to justify its broader adoption, it would be important to more clearly **demonstrate how it provides novel and reproducible biological insights beyond** what can be obtained **through established methods**, particurly in the field of drug screening to clearly demonstrate its added value in distinguishing treatment specific phenotypic. I leave it up to the editor if more in-depth analysis is required (biological replicates, more therapies,...) to support the application of SAM-SPOT for drug screening or that the example you provided in the manuscript is sufficient.*

Author's reply:

We agree with this reviewer that if we were simply to classify the toxicity of known chemotherapeutic agents, there would be no need to use such a complex high-dimensional approach as SAM/SPOT. Indeed, SAM/SPOT was not developed to only be used in the screening of known cytotoxic chemotherapeutic agents. It was developed as a tool to enable the identification of modulators that can cause subtle photodynamic changes without prior knowledge, i.e. changes that are "imageable". The use of known chemotherapeutic agents here is to provide a proof of principle that SAM/SPOT can classify drug toxicity based on phenotypical changes.

Our objective in developing SAM/SPOT is to generate an analytical workflow that simplifies temporal analysis of biological datasets for non-experts with application to drug screening but also general live-cell imaging assay analyses. In these two manuscripts, we sought to demonstrate the versatility and 'turn-key' nature of the simplified SAM-SPOT workflow and SPOT's versatility across diverse scenarios, with drug screening as one example. A major strength of SAM-SPOT is its ability to generate meaningful insights from small datasets without need for a priori specification of the analysis metrics or reference datasets. Thus, we believe SAM-SPOT will prove useful to a diverse biological imaging community. As demonstrated by the SAM contribution and module analyses SAM-SPOT can auto-tune to the relevant features, and reproducible clusters and modules are discovered across biological and technical replicates. Lastly, with SAM-SPOT it is easy to perform pooled analyses across experiments, and to identify and perform batch correction if needed.

Similar to SAM/SPOT, other state of the art method has been developed to specifically perform organoid toxicity assay and one such method is Normalized Organoid Growth Rate metric (NOGR) (PMID: 39627437). We bench marked SAM/SPOT against NOGR (Reviewer Suppl. Fig. 3k). DMSO and docetaxel treated organoids were used as the negative and positive drug controls. We analyzed the chemotherapy drug screens of the two different Patients B and C with NOGR (Reviewer Suppl. Fig. 3b and 3h, respectively). To minimise batch effect differences between individual plates, we computed NOGR for individual organoids per plate, per condition. NOGR classification of the drug toxicities corroborates with SPOT analysis (Reviewer Suppl. Fig. 3k). However, SPOT does not require specifying reference controls to compute, and does not require a priori detection and removal of dead organoids or accounting for partially tracked organoids which may contribute to the high variance in the NOGR measurement across plates. SPOT provides additional outputs: (enabled by the multidimensional phenome) phenotype cluster stacked bar-plots and transition graphs, and phenotype trajectories that provide additional temporal insight and capture of organoid heterogeneity. We have now included additional discussion on the pros and cons of using multidimensional features vs targeted metrics.

Small remarks:
1) In Supplementary Figures 3a c(i), organoids treated with docetaxel appear to exhibit strong caspase signal, despite maintaining a morphologically healthy appearance (i.e., not dark, fragmented, or granulated). This

seems contradictory, as high caspase activity is generally associated with apoptotic progression and eventual cell death.

Author's reply:

We thank the reviewer for this thoughtful observation. We have re-examined the data and determined that elevated background fluorescence in the green caspase channel may have contributed to an exaggerated appearance of caspase activity.

To address this, we re-processed the images using background subtraction and applied a mild contrast enhancement consistently to all image channels to more accurately distinguish true caspase signal from background noise. This improved image processing revealed that the caspase signal originates from apoptotic activity within:

- granulated, dying organoids,
- dead cells embedded within morphologically healthy organoids, and
- surrounding cellular debris.

To clarify this interpretation, we now include reviewer-only snapshots from the time-lapse videos at 0, 2.5, and 5 days. These include both the caspase channel alone and merged images with the brightfield channel, allowing for clearer visualisation of the dynamic changes in caspase activity and organoid morphology over time.

In each image, we have annotated caspase activity arising from the following sources:

- **(A)** Healthy organoids — clearly visible at day 0 in the merged panel showing one representative example which remains healthy through the time-lapse).
- **(B)** Dead cells embedded within otherwise healthy-appearing organoids.
- **(C)** Dead cells within the surrounding Matrigel.
- **(D)** Granulated, dying organoids — clearly visible by day 5, as originally presented in Suppl. Figures 3b c(i) for docetaxel.

Zoomed-in images of each labelled organoid are included below the original image.

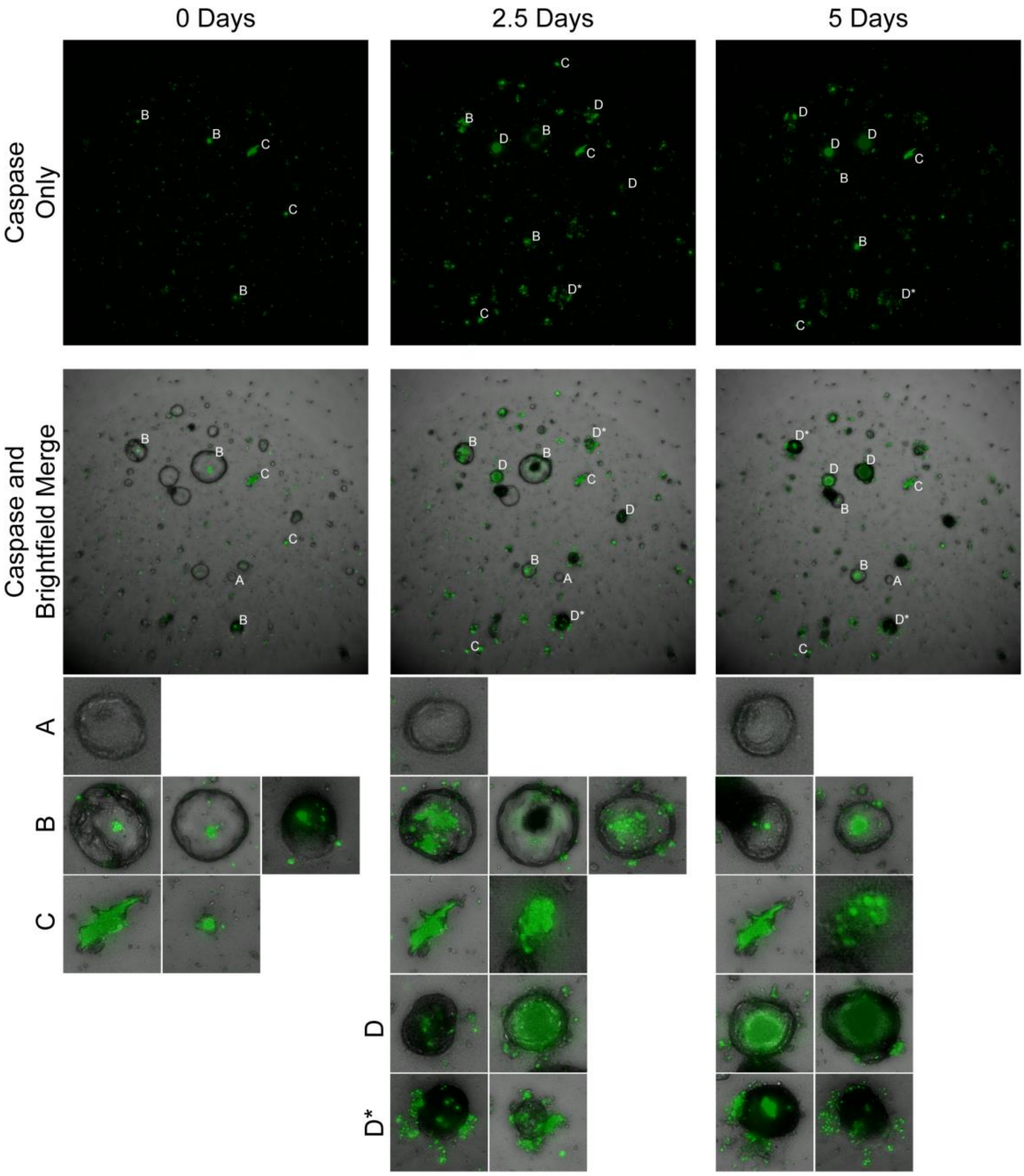

We hope this improved visual representation resolves the apparent discrepancy and provides clarity regarding the origin of the caspase signal in our system.

Furthermore, according to Figure f, the number of Cytotox Green spots (used to mark cell death) for the docetaxel condition remains constant suggesting that this caspase activity is not translating into cytotoxicity.

Why do healthy-looking organoids exhibit such strong caspase activity and could there be technical artefacts in the caspase reporter signal? It would be helpful if the authors could clarify this observation.

Author's reply:

We thank the reviewer again for this valuable observation. As noted, Cytotox Green selectively stains cells that have lost membrane integrity, indicating late-stage apoptosis or necrosis. In contrast, Caspase 3/7 Green detects activated caspases during the early to mid-stages of apoptosis, when the cell membrane remains intact. The compounds used in this screen (Cisplatin, 5-Fluorouracil, and Docetaxel) primarily induce apoptosis.

It is therefore expected that organoids undergoing treatment would display elevated Caspase 3/7 Green signal compared to Cytotox Green, particularly at intermediate timepoints. This reflects the temporal sequence of apoptotic events: caspase activation precedes membrane permeabilization. Hence, caspase activity may not directly translate into detectable cytotoxicity at the time of measurement. While a subset of organoids (e.g. D*) begin to exhibit loss of membrane integrity by the end of the time-lapse experiment, the majority of docetaxel-treated organoids maintain a morphologically intact, spherical structure. This suggests that although apoptotic signalling has been activated, many cells have not yet progressed to late-stage apoptosis where Cytotox Green uptake would occur. The relatively constant level of Cytotox Green staining across treatment conditions likely reflects baseline non-apoptotic cell death or background signal from cellular debris, where membrane integrity has already been compromised.

2) Classification of Toxicity Levels in Figure 3c (iv)
The authors classify the tested drugs into "low," "mild," and "high" toxicity groups (Fig. 3c iv). However, it is not clearly described how these toxicity classifications were determined. To strengthen the biological relevance and interpretability of the findings, it is essential to validate these classifications against standard drug-response metrics, such as growth rate based drug response metrics (e.g., GR or NDR), which differentiate cytostatic effects (likely corresponding to "mild toxicity") from cytotoxic effects (presumably "high toxicity"). Without such evidence, the presented classification appears arbitrary or subjective. Incorporating these standard benchmarks would significantly enhance the clarity and robustness of the authors' conclusions.

Author's reply:

As suggested by the reviewer, we have performed additional analysis on our data with the Normalized Organoid Growth Rate (NOGR) [PMID: 39627437] metric. We used DMSO and Docetaxel as the reference negative and positive drug controls, and analyzed plates from two different patients (Reviewer Suppl. Fig. 3k). The results are consistent with SAM-SPOT classification based on multiple replicate analyses: DMSO, 6µM Cisplatin, 100µM Cisplatin as "low" toxic and 5FU and Docetaxel as "high" toxic. This has now been included in the revised manuscript.

3) The term 'flattening' of the organoids is used. If I'm correct, these are cells that migrate towards the bottom of the Matrigel dome and attach to the bottom, indicating invasive and migratory behaviour? So it might be interesting to look at EMT related gene expression and the role of WNT3A in maintaining epithelial integrity in normal organoids.

Author's reply:

Thank you for the suggestion. To determine whether the "flattening" phenotype is indicative of invasive and migratory behaviour, we analysed the expression of EMT-related genes present in our dataset. Specifically, we looked at the mean normalised expression of EMT master regulator genes (SNAI1, SNAI2, ZEB1), epithelial genes (CDH1, CLDN1, CLDN3, CLDN4, CLDN7), and mesenchymal genes (VIM, AXL). This analysis (top left panel of below reviewer-only figure) revealed that the expression of EMT master regulator genes is negligible across conditions. While VIM shows a higher mean normalised expression in ENR Day 9 (the condition with the most prominent "flattening" phenotype), the expression of epithelial genes (CDH1, CLDN1, CLDN4) remains comparatively high. Moreover, the fraction of cells expressing VIM (top right panel of below reviewer-only figure) is lower than the fraction of cells expressing epithelial genes (CDH1, CLDN1, CLDN4). Critically, the ratio of VIM to CDH1 remains constant across conditions, factoring the error bars (standard error of the mean) per condition. Together, these results suggests that ENR Day 9

organoids are not shifting toward a mesenchymal state, and that the observed “flattening" phenotype is unlikely to be associated with EMT-driven invasive and migratory behaviour. Most of the other EMT-related genes have very low expression across all other conditions.

Mean normalized expression of EMT genes per condition

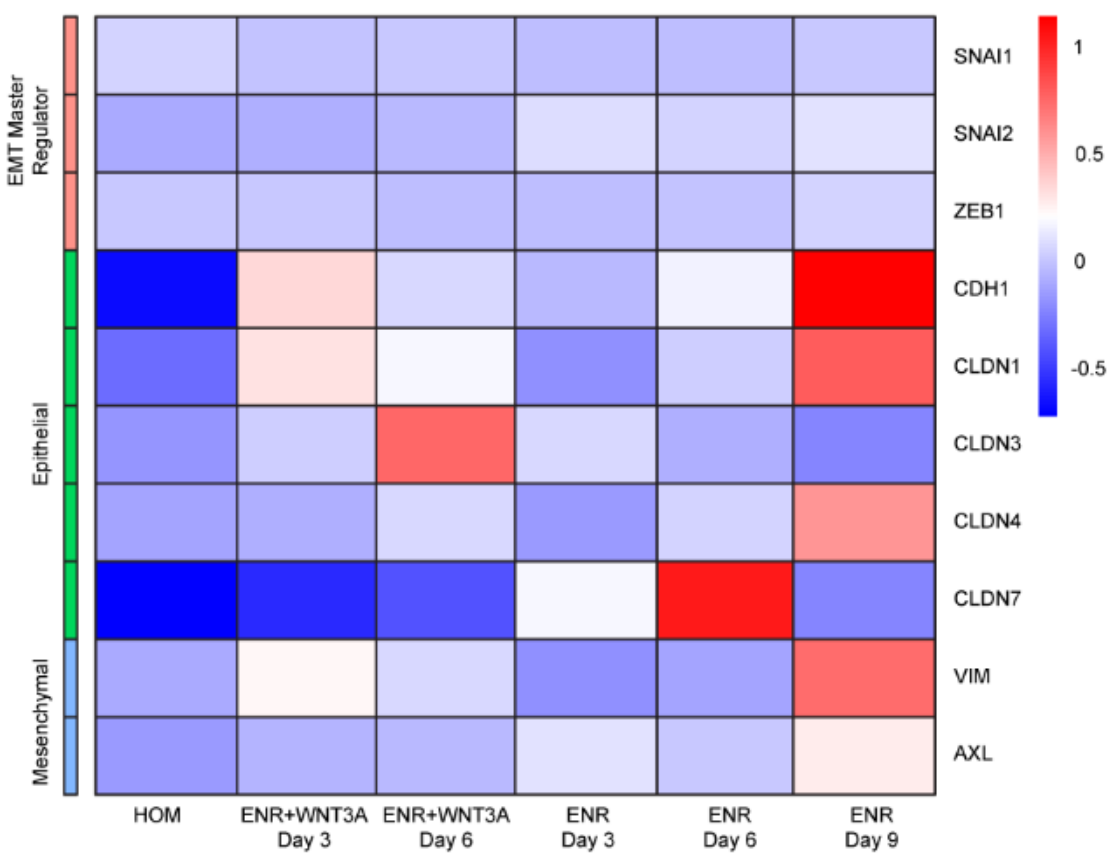

Fraction of cells expressing EMT gene per condition

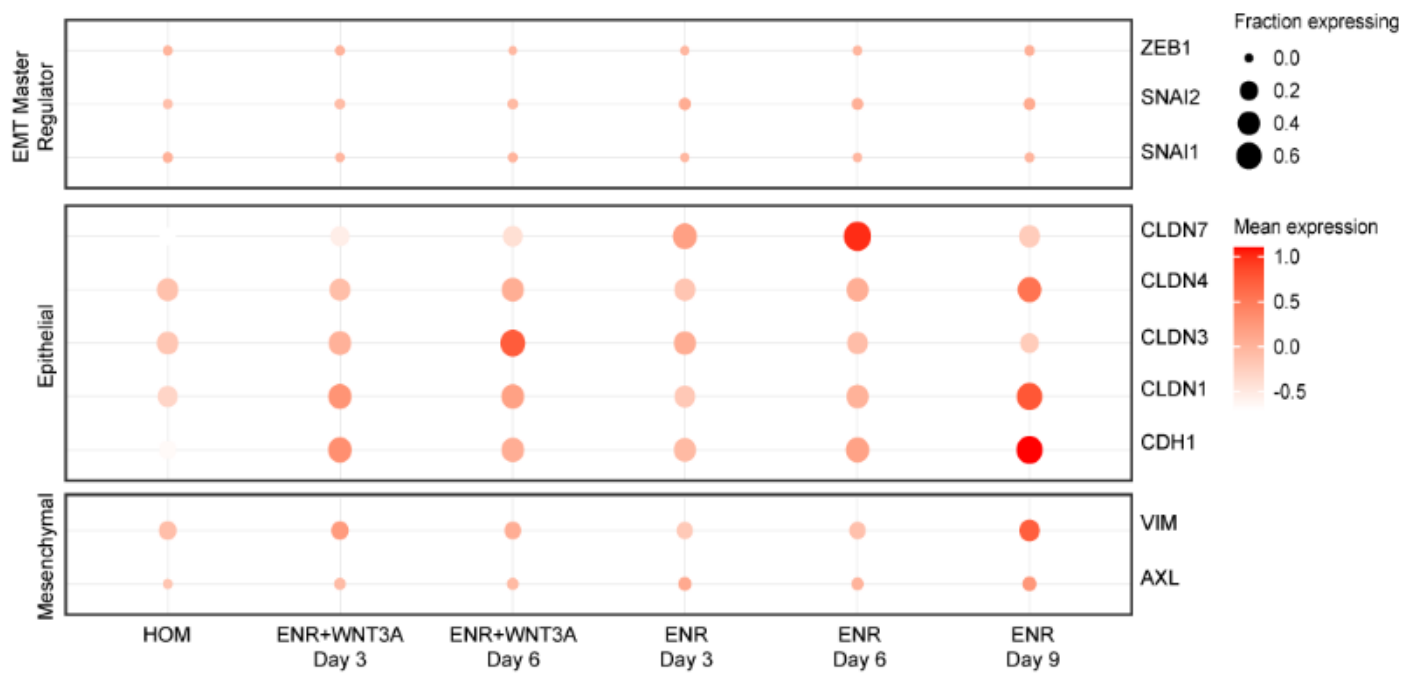

VIM/CDH1 ratio per condition (SEM)

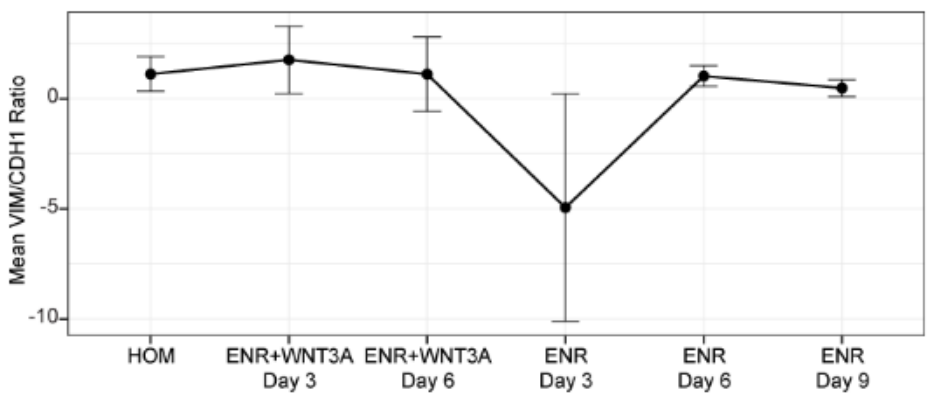

Reviewer #3 (Remarks to the Author):

Reply to author comments addressing main concerns:
The three primary concerns identified by the authors: presence of artifacts (1), impact of inaccurate organoid segmentation (2), and applicability for 3D time series data (3) are adequately addressed in the reply and thoroughly addressed in the manuscript through additional figures, additional supplementary data, and the establishment of simulated datasets.

(1)
The authors address this point in reply to reviewer 2s comments. They comment: “Each cluster is not necessarily a biological phenotype, and more than one cluster could relate to the same biological process e.g., two clusters relate to cell death. The primary biological analysis is in the phenotype evolution of organoids between conditions. SPOT assumes that over time, only phenotype clusters corresponding to true biological phenotype dynamics will exhibit differential temporal evolution”.

I agree that most artifacts encountered (debris, illumination induced) shouldn’t exhibit any temporal dynamics, and that this is a strong safeguard of the method.

Author’s reply:

Thank you for the supportive comment.

The authors further support the robustness of SPOT in Supplementary Fig 2c. I would note that special care should be taken with some of these imaging and segmentation based errors.

The artifact labelled (ii - Overexposure) is also a key biological event that occurs e.g. organoids becoming cystic (darkening), in later timepoints of organoid culture.

Author's reply:

Our simulations show tolerance to slight darkening i.e. at levels 1-4. Beyond this, the darkening of the organoids shifts the position of points in the UMAP space. This is seen in Suppl. Fig. 2c by the discrepancy in stacked barplot disagreement and phenotypic clustering. This higher level of darkening (levels 5-9) is likely to be a result of actual biological processes such as cytotoxicity rather than an acquisition artefact. As shown in these simulation experiments, and particularly in the chemotherapy screen analyses, SAM-SPOT picks up genuine 'darkening' signatures which will also generate a temporal evolution from more transparent organoids to darker organoids.

And (v – Overlap) shows consistent significant disagreement between the mutual information, and adjusted random index traces (Phenotypic clustering agreement).

Author's reply:

Both measures reflect the observation that the UMAP splits into two branches, with each branch corresponding to whether the left or right organoid is overlapped (see below a reviewer-only figure with individual image patches overlaid on the UMAP). The clustering agreement should converge to approximately 50% for a symmetric split as perturbation level increases to level 9. The resulting joint UMAP is however not fully symmetrical and this leads to the discrepancy due to differences in the calculation of the adjusted random index (ARI) and adjusted mutual information scores (AMI). The ARI should be used when the ground truth is expected to have large equal-sized clusters, whilst AMI should be used when the ground truth clustering is unbalanced and there exist small clusters (Romano, Simone, et al., Adjusting for chance clustering comparison measures. *Journal of Machine Learning Research* 17.134 (2016): 1-32) https://jmlr.csail.mit.edu/papers/volume17/15-627/15-627). In this case, ARI is preferred, but we present both for completeness. The trend across levels 1-9 is the same for both metrics.

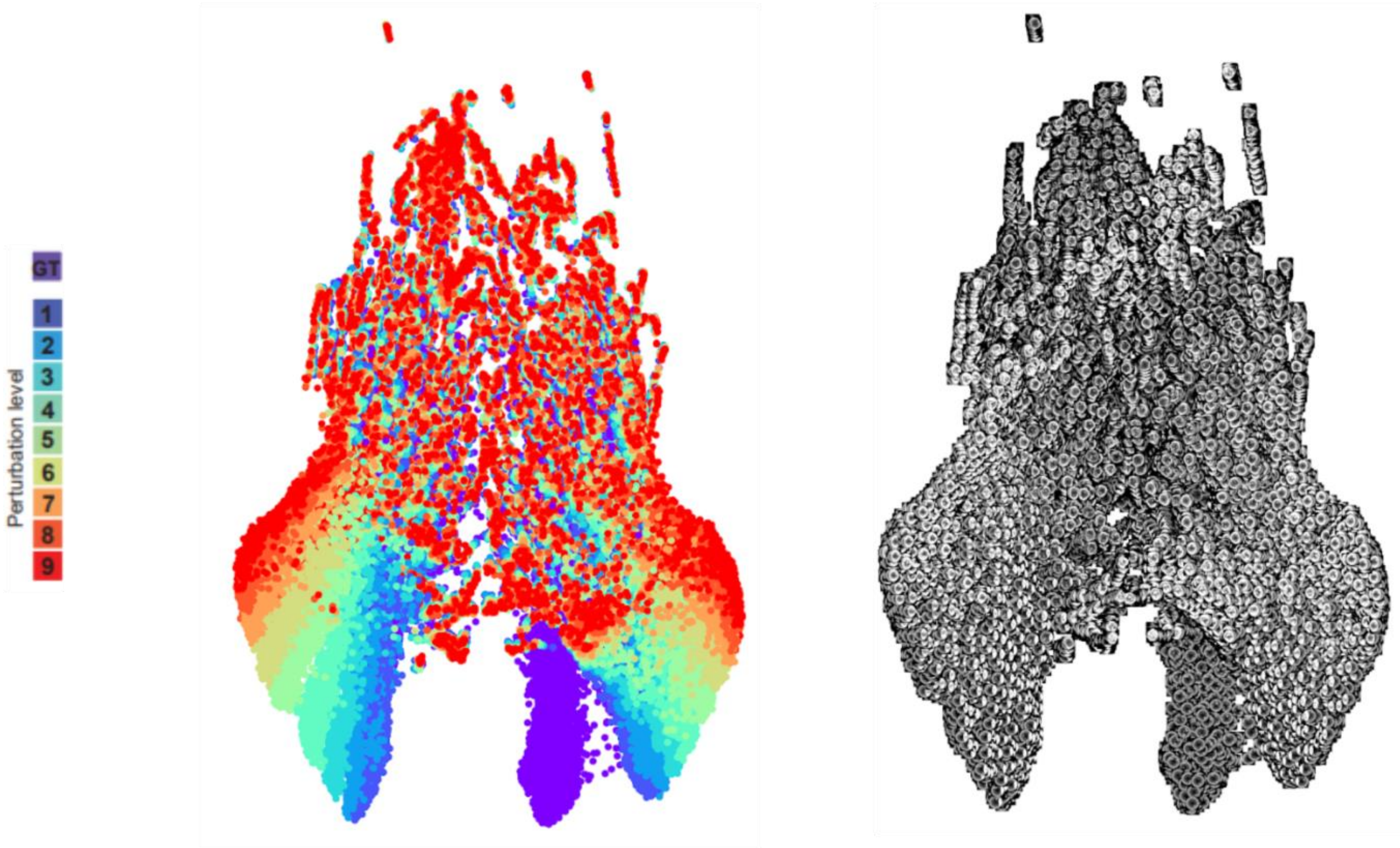

(2) Supplementary movie 2 (Movie S2 mouse intestinal organoids plus VC treatment), Timestamp 0:50-1:04 shows that despite the listed improvements by the author, special care should be taken when applying SPOT to non-simulated organoid data.

Performance varies significantly in the 4 treated conditions, with the WT + V/C condition showing the appearance of a "regular organoid" in the traced outline (teal in bottom right image), but the actual morphology being strikingly different from the other samples.

Author's reply:

We thank the reviewer for this observation. We re-segmented the organoids with the latest state-of-the-art segmentation methods to obtain more accurate segmentation masks. We trained a custom Cellpose3 model (PMID: 39939718) to first detect and segment all individual organoid instances. For comparison, the trained Cellpose3 model has an average precision at IoU cutoff of 0.5, $\mathrm{AP}_{0.5}$ = 0.64 whereas SPOT's provided detect and segment approach has $\mathrm{AP}_{0.5}$=0.45. We then re-tracked the bounding boxes of these improved masks, and fed the bounding boxes as prompts to µSAM (PMID: 40490528) to obtain fully temporally-consistent tracked organoid segmentation masks. Reanalyzing the dataset with the improved segmentation masks, we found the same clustering of the 4 treatments (new Supp. Fig. 3b-d). We further show that the majority of individual outputs: phenotype clusters, transition matrices, and SAM modules are preserved. The additional validation experiments show that suboptimal segmentation does not significantly affect the conclusions of SAM-SPOT analysis.

Supplementary movie 4 (Movie S4 - human organoids wnt3a addback), Timestamp 0:44-049 demonstrate that when organoids become cystic (Dark, clumping/aggregation, and highly irregular in shape) segmentation performance severely degrades.

In the HOM (Control), ENR + WNT3A (day 9) & ENR (no WNT3A) conditions, organoids become cystic, and the object detector fails to capture these for an extended number of frames.

The authors comment on this in their reply: "We also emphasise that the tracking is implemented only to identify genuine organoids (alive or dead) which we define as being tracked for a minimum number of frames (here, 5 frames)" which works to prevent faulty segmentation from being picked up, but does not allow the method to capture the unique morphology captured by these conditions. Improvements to the organoid detection routine would strengthen SPOT to better capture these unique morphologies.

However, this would serve to enrich the analysis – and the current methodology is adequately equipped to cover most tested and demonstrated conditions, erring on the side of caution.

Author's reply:

We thank the reviewer for the insightful comment. SPOT is designed to err on the side of caution and to work without requiring perfect detection, segmentation and tracking. The provided detector and segmenter obtain adequate performance over diverse imaging conditions, as demonstrated. The reanalysis results of the mouse intestinal organoids with improved segmentation masks suggests that the general conclusions are preserved. Nevertheless, prospective users may wish to use more optimal detectors, trackers or segmenters for additional analyses. The flexibility to customize inputs was a major consideration to release our SAM-SPOT framework as a Python Library instead of a fixed step-by-step pipeline. We have added additional discussion to highlight potential adaptations in performing a SPOT analysis.

(3)
The creation of an organoid simulator, simulated datasets A & B, and creation of additional supplementary figures are greatly appreciated. 8 scenes are provided across 2 different datasets (with replicates for the XYZ condition), with individually tracked organoids, their masks, and resulting projections.

Specifically on the original remark "It is however currently unclear whether SPOT will work well for 3-dimensional time-series data. A possible complication can be that the motion module in SPOT can confuse X/Z movement for a phenotypic response of cells" The authors demonstrate that XYZ movement shows a distinct SAM phenome for 2 replicates compared to the XY movement.

As the authors point out, the stacked barplots of cluster frequency do not show distinct differences, but that the XYZ movement is captured in the phenomic landscape (annotated with XYZ-only patch).

This supports the claim that SPOT, even when working with projected data, shows promise to be applicable in 3D time series data. As the authors themselves pointed out (Supplementary Figure 1b), "whilst the available space in the ECM dome constrains organoid movement to be largely planar in xy" this reviewer acknowledges the evidence and effort to make the methodology robust for non-ECM-dome configured organoid tracking as well.

Author's reply:

We thank the reviewer for appreciating our benchmarking efforts. On closer inspection, this time we noticed a slight increase in cluster 2 population for the XYZ-movement replicates in the stacked barplots, and cluster 2 encompasses the UMAP region which only XYZ patches mapped to. The revised cluster transition graphs

show less distinction between XY and XYZ movements, but clustering is still able to distinguish between XY and XYZ movements.

I have enjoyed reading through the revised manuscript and applaud the authors for their significant improvements to the manuscript, and the other reviewers for their constructive and extensive feedback. Splitting up the manuscript helped bring focus to the text, allowing more evidence to be provided to support applicability of SPOT to real-world organoid data.

Author's reply:

We thank the reviewer for taking the time to read and offer feedback on our two manuscripts.

Reviewer #4 (Remarks to the Author):

I am confused as the authors seem to have two versions of the same article. One named "Development of a universal imaging "phenome" using Shape, Appearance and Motion (SAM) features and the SAM Observation Tool (SPOT)" and one this one that I am writing review for now. I have already wrote reviews for the "developement ..." version and I will paste it here as well. My only new comment for this current version "Identifying ..." is that this version seems to have more support from biological and simulated data but lacks the comparison to the state-of-the-art. Why did not the authors submit one version with all three in place (biological verification, simulated experiments, literature comparison)? That would have been best in my opinion.

Author's reply:

Thank you for the observation.

Our initial submission was a combined single manuscript. However, following reviewer 2's comments and during the review process, it was felt that the joint paper lacked focus and was too long for a general readership. Consequently, we split the manuscript into the current two manuscripts: one focussed on introducing the analytical framework, and benchmarking its performance on public datasets with emphasis on single cell imaging; and the second demonstrating the ability to adapt the framework to analyze 3D organoid dynamics through 2D projection videos, introducing a new organoid simulation dataset in the process.

We feel that the separation of these manuscripts adds greater clarity, and caters towards the two different general readerships that would be interested in our work: the tool developers and the organoid community.

The below is the same comments as I had for the "Development ..." version of the paper which are relavent for the current version, i.e., "Identifying ..." too. I do not have the biological knowledge to verify any biological verification that the "Identifying ..." version claims, as I am a computer scientist.

1. What are the noteworthy results?

[Answer from the reviewer to the Editor] *The authors claim that a universal set of 2185 image-derived features is developed to quantify an object's phenotype ("standard" image-based "phenome"); subsequently,*

they provide a tool for image "sequencing" analysis of phenomes. For temporal analysis, they compute the HMM phenotype cluster transition graph that shows the different state of each detected phenome cluster over time.

2. How does it compare to the established literature?

[Answer from the reviewer to the Editor] *I. In the introduction, the authors mention two previous feature descriptors, CellProfiler and TISmorph. But they only compare to Cellprofiler. The comparison is not enough. The authors should compare to TISmorph36 too.*

Author's reply:

We have added additional comparison to TISmorph using the official code (https://github.com/Elaheh-Alizadeh/Quantifiction-of-shape-and-structure) in the methods paper (NCOMMS-25-25126A) for the analysis of MPEG-7 shapes (new Suppl. Fig. 3b a of NCOMMS-25-25126A), Normalized Brodatz textures (new Suppl. Fig. 3b b of NCOMMS-25-25126A) and Brodatz textures MPEG-7 shapes (new Suppl. Fig. 3b c of NCOMMS-25-25126A). As can be seen, TISmorph is the worst performing. For Brodatz textures MPEG-7 shapes, we further assessed the supervised classification performance with respect to the size of the training split (Suppl. Table 3). We see SAM-SPOT is the best overall. However, if there is insufficient training data i.e. <5%, the feature set with lower dimensionality is more superior. Since generally, we use >50% training data, SAM-SPOT is the superior feature set.

II. The comparison is limited to two methods, i.e., merely done for CNN and CNN-LSTM based autoencoder. The authors should compare to more methods e.g. generative models and graph-based models. Two examples of such methods: A) Dynamic graph convolutional foldingnet in "3D single-cell shape analysis of cancer cells using geometric deep learning", B) "DeepSea is an efficient deep-learning model for single-cell segmentation and tracking in time-lapse microscopy". Then given their claim of rotation-invariance, they should compare to different VAE tackling rotation-invariance and not just a naive auto-encoder: C) "ME-VAE: Multi-Encoder Variational AutoEncoder for Controlling Multiple Transformational Features in Single Cell Image Analysis"

Author's reply:

We thank the reviewer for the suggestions of possible additional benchmarks. After further research, we decided to benchmark SAM/SPOT against two state of the art methods; DINOv3 and O2-VAE rather than the methods suggested by this reviewer for the following reasons:

a) Dynamic graph convolutional folding net (PMID: 40112779) was developed for i) shape-only features and ii) 3D point-cloud only inputs. Our work, SAM-SPOT is applicable currently only to 2D videos. In our organoid paper, we project 3D z-stacks to 2D videos for analysis.
b) DeepSea (PMID: 37426758) is only developed for segmenting and tracking organoids. SAM-SPOT doesn't claim novelty in the segmentation and tracking, which are based on training already published neural network architectures. These tasks are not the goal of SAM-SPOT which aims to be a pipeline for analysis after you have segmented and tracked organoids/cells. The segmenter and tracker we provide work well for the diverse datasets we have tested and is provided for user convenience to accomplish these two prerequisite tasks. Indeed, we have designed SAM-SPOT to be modular, and encourage users to utilise their own segmentations and tracking if they are better suited to their particular use case.

c) ME-VAE: Multi-Encoder Variational AutoEncoder (PMID: 35322205) – We agree that this framework would be an interesting benchmark. However, we found that the official code repository, https://github.com/GelatinFrogs/ME-VAE_Architecture does not provide code to create the data transformations for custom training and it would be unfair to benchmark ME-VAE with any custom data transformer we would have to write as replacement. Instead, we found an alternative, O2-VAE (PMID: 38310122) that in the same spirit, aims to learn equivariant features. Different to ME-VAE, O2-VAE uses an orientation-invariant convolution encoder, instead of relying on explicit data transformations (https://github.com/jmhb0/o2vae), allowing us to retrain the network.

The motivation of using CNN and CNN-LSTM was to create baseline methods where the same encoder architecture could be used for both fixed and temporal imaging.

We have now added additional classification benchmarking of LIVECell and HeLa cell division datasets to the following two state-of-art architectures, chosen to represent the two current major lines of research in feature engineering: i) self-supervision, ii) equivariant learning:

1) DINOv3 (https://github.com/facebookresearch/dinov3) – This family is state-of-the-art in self-supervised deep learning for general computer vision and one of the most popular benchmarked also for cell imaging (e.g. PMID: 39929956).
2) O2-VAE (https://github.com/jmhb0/o2vae) – This recent state-of-art cell feature learning VAE network uses prealignment and O(2)-equivariant steerable CNN layers to ensure equivariant feature learning.

We trained the DINO networks using as close to the same number of features as SAM-SPOT as we could not vary this, and trained O2-VAE with the same number of features as SAM-SPOT (in alignment with this reviewer's later comment on maintaining the same number of features). With DINOv3 we could not directly specify the number of features, therefore we used the version with 768 features and then performed L2 penalized linear support vector machine to select a number of top-performing features to match SAM-SPOT. Both networks are designed only for fixed imaging. Therefore, similar to CNN-LSTM, to adapt to the temporal dataset, we first computed the DINOv3 or O2-VAE features for individual video frames then trained a multi-layer perceptron (MLP) encoder with bidirectional LSTM incorporating the same 5-frame time window information as the CNN-LSTM. The temporal features were then the encoded features of the MLP encoder whose number was matched to the number of SAM features.

Our benchmarking results on supervised classification find SAM-SPOT features as the best performing (Suppl. Table 3,4). Moreover, we found the more state-of-the-art networks did not perform much better than CNN and CNN-LSTM features.

3. Does the work support the conclusions and claims, or is additional evidence needed?

[Answer from the reviewer to the Editor] *I. In suppl. Fig. 8 the number of features differ between SAM and CNN-LSTM. What happens if the number of features set to be the same? That way it may be more fair to compare. The same should be done for the table of results comparing the accuracy of CNN and CNN-LSTM (right now SAM has not the same number of features as the other two).*

Author's reply:

We reperformed the comparison, setting CNN and CNN-LSTM feature to match the number of SAM features in the LIVECell and HeLa cell division datasets. We find little difference in classification performance. We find

the same behavior in SPOT analysis with the neural network features not fully capturing the phenotypic diversity. In sum, SAM still greatly outperformed the CNN and CNN-LSTM features.

II. In all illustrations of HMM cluster transition probability, it looks like a state machine rather than a probabilistic state machine (HMM. The author should annotate what each edge coloring represents as a probability.

Author's reply:

Yes, the cluster transition probability is a state machine. To avoid confusion, we have now removed the HMM analysis. The cluster transitions are estimated as a "state machine", based on the observed occurrences of the phenotype cluster in the next timepoint, given the phenotype cluster in the current timepoint:

$$\Pr(\text{cluster}(t+1) = j \mid \text{cluster}(t) = i) = \frac{(\#\text{cluster}(t+1) = j|\text{cluster}(t) = i)}{\Sigma_j(\#\text{cluster}(t+1) = j|\text{cluster}(t) = i)}$$

With this model, however, we found self-cluster transitions i.e. cluster *i* to cluster *i* to dominate the transition probability. Therefore, we now plot the transition graph for the relative inter-cluster transition probability (row-normalized transition matrix after removing the diagonal self-transition entries):

$$\Pr(\text{cluster}(t+1) = j \mid \text{cluster}(t) = i, j \neq i) \in [0,1] = \frac{(\#\text{cluster}(t+1) = j|\text{cluster}(t) = i, j \neq i)}{\Sigma_{j\neq i}(\#\text{cluster}(t+1) = j|\text{cluster}(t) = i, j \neq i)}$$

The arrow is drawn from the source cluster in the current timepoint to the target cluster in the next timepoint. To make this clear, the arrow is colored by the source cluster. The solidity (i.e. transparency) of the arrows' color reflects the magnitude of this probability (see figure below). This information is included in the figure legends. Specifically, to enhance contrast with the revised Markov model, we graph the probability raised to an exponent, γ, $(\Pr(\text{cluster}(t+1) = j \mid \text{cluster}(t) = i, j \neq i))^\gamma$ with a minimum value cutoff and a maximum value cutoff. If not stated in-text, $\gamma = 3$, min. cutoff=0, and max. cutoff=0.6. The arrow widths are all the same.

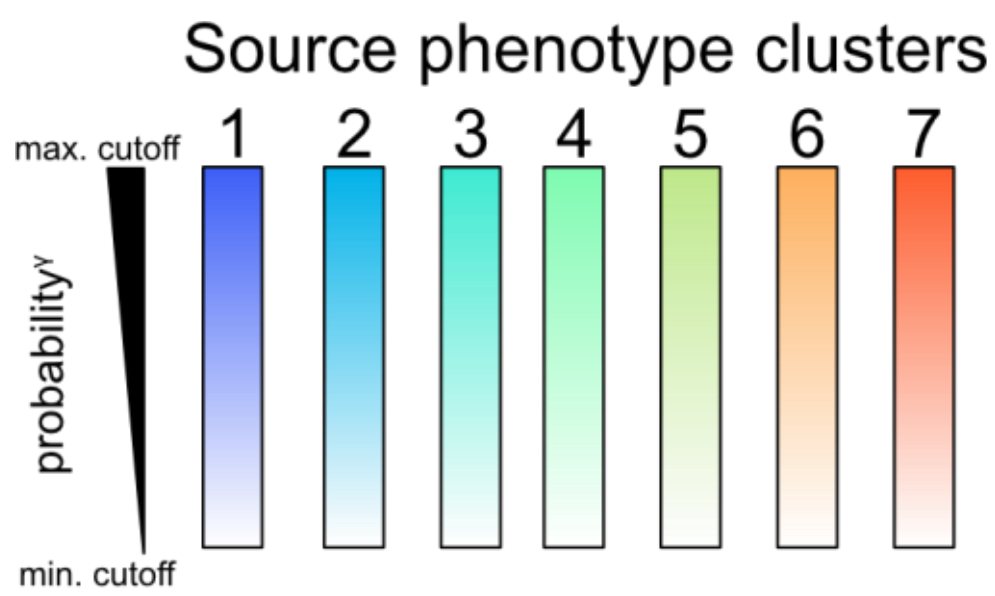

III. See points 2 and 5 and address them too.

Author's reply:

It seems to the authors that these two comments (2. how does it compare to established literature and 5. Is the methodology sound? Does the work meet the expected standards in your field?) were the questions from the editor to reviewer 4. Therefore, we felt it best to leave the reviewer 4 to address these comments. However, as reviewer 4 has made specific comments in relation to point 5, we have provided some specific replies to the comments.

From our point of view, we of course feel strongly that SAM/SPOT represents a sound, state of the art technology in the field and will transform our ability to quantitatively capture valuable information from live imaging.

4. Are there any flaws in the data analysis, interpretation and conclusions? Do these prohibit publication or require revision?

[Answer from the reviewer to the Editor] *I cannot see any.*

Author's reply:

We thank the reviewer for their consideration.

5. Is the methodology sound? Does the work meet the expected standards in your field?

[Answer from the reviewer to the Editor] *I. I do not understand their HMM. I thought first Kmeans is used to find 7-8 clusters and then for "each cluster" the temporal analysis is done (this is what the authors claim in line 1319 section). This makes sense to me. However, in their HMM transition (shape of state machine with color-coded arrows), I see that they show transition between those 7-8 clusters. Why this? and why not the state transition for each cluster separately? If the author answer this as "we want to see transition between different clusters" then my judgment is that their modeling is not sensible - they should NOT first do Kmeans to detect states of an HMM (the whole point of HMM is to find those states). Instead, they should have directly applied HMM and then Viterbi to find the most probable sequence, which would unfold the trajectory of states - the states would be detected by fitting the HMM and would not need to have K-means in advance.*

Author's reply:

We agree with the reviewer that having found our phenotype clusters our aim was to "see transition between different clusters". Specifically, we wanted to estimate the probability of an organoid being of phenotype cluster *i* transitioning to phenotype cluster *j* in the next timepoint. We did not want to explicitly link the hidden states of a HMM with the definition of phenotype clusters as this would restrict the application our pipeline to strictly longitudinal data. In our current design, phenotype clusters can be found for both fixed and live-cell imaging. To avoid confusion of the use of HMM in our data analysis, we have now replaced HMM with the explicit and simplified approach of direct computing the desired cluster transition probabilities directly as a "state machine".

Specifically, we use Kmeans to find automatically a discrete number of clusters, typically 7-8 clusters which partition the UMAP phenomic landscape. Each cluster then represents a prototypical set of SAM characteristics.

We then use the clusters to go back to the single-object tracking information to assign to each object instance in the track its phenotype cluster label. This gives a cluster label trajectory e.g. 1,1,1,1,1,2,2,2,2,2 where the object was tracked for 10 timepoints, initially being cluster 1 and then transitioning to cluster 2. Within our cluster transition analysis, we aim to estimate the probability that an object instance is phenotype cluster *j* at time *t+1,* given that it was phenotype cluster *i* at time *t.*

We want to highlight that we regard i) finding the phenotype clusters, and ii) finding the cluster transition probability as two independent and separated tasks. The advantage of this is that task i) is still applicable

when we have fixed imaging (non-temporal) data. We do not want i) to be linked to the hidden states of a HMM.

We apologise that the HMM has caused confusion in the interpretation of our methodology. As mentioned above, in our revision, we now compute the desired cluster transitions directly as a "state machine", from the observed occurrences of the phenotype cluster in the next timepoint, given the phenotype cluster in the current timepoint, pooling across all tracked objects in the analyzed dataset:

$$\Pr(\text{cluster}(t+1) = j \mid \text{cluster}(t) = i) = \frac{(\#\text{cluster}(t+1) = j|\text{cluster}(t) = i)}{\Sigma_j(\#\text{cluster}(t+1) = j|\text{cluster}(t) = i)}$$

For label smoothing, we provide the users the option of performing a sliding window majority filter. Each label trajectory will be scanned with a sliding window of size *w* and the output is the majority label in the window. Activating this option smooths sporadic, short-lived transitions such as the 1,2,1 transition in the sequence 1,1,1,1,1,2,1,2,2,2,2.

II. The authors should provide answer to this: why SAM? I am not sure if motion is sufficiently captured since it is "Motion as combined changes in the object's shape, appearance, and centroid between consecutive timepoints". That is, why not separately consider rotation and other potential spacial variations which can change the perception. See, for example, "ME-VAE: Multi-Encoder Variational AutoEncoder for Controlling Multiple Transformational Features in Single Cell Image Analysis".

Author's reply:

Computer vision refers to shape as boundary, edge, or contour features, appearance as texture features and motion as optical flow features. The purpose of SAM was to capture instantaneous phenotype and we consider SAM as three key conceptual components to an image. Each provides unique and complementary information as illustrated with the cartoon example of a bird in Fig. 1b of the methods paper (NCOMMS-25-25126A). This is not a new concept, and forms the basis of computer vision prior to deep learning. Computer vision refers to shape as boundary, edge, or contour features, appearance as texture features and motion as optical flow features.

The need for all three sets of features is evidenced by i) classification performance in YouTube Dataset; and ii) the variation contribution of shape, appearance and motion features, and the categorization of top features in each SAM module which changes for each analyzed dataset. If consecutive time motion is uninformative i.e. its inclusion doesn't contribution any information to the analysis, then using motion features should not contribute any improvement in classification performance, or dataset variance.

This is not however what we observe:

i) YouTube classification performance:
Suppl. Table 2 Methods paper highlights how motion features is the best performing single set of features, whilst shape features are the worst. Moreover, the joint SAM outperforms all single feature sets.

ii) Variation contribution and top performing features in SAM module analysis:
- Fig. 5 in our Methods paper NCOMMS-25-25126A shows U373 cells which predominantly migrate and change shape. Correspondingly, we measure contribution scores of 0.60 Shape, 0.20 Appearance and 0.78 Motion. Moreover, most of the SAM modules (8 of 9) are dominated by motion-based features, those prefixed with 'div_sift_', 'curl_sift' and 'speed_reg'.

- Suppl. Fig. 5 in our Methods paper NCOMMS-25-25126A depicts the opposite extrema of HL60 cells dividing in-place with little to no migration. Consequently, we measure contribution scores of 0.59 Shape, 0.78 Appearance and 0.22 Motion. Likewise, few modules (3 of 16) are driven by motion-based features.

We would like to reiterate that the purpose of SAM was to capture the instantaneous phenotype. We agree that to "fully capture motion behavior" e.g. the full migratory sequence of a cell, we should compute features over a longer time interval. However, it is unclear how long this time interval should be. We would then be reverting back to the standard trajectory analysis framework. Conceptually, this would no longer be instantaneous information. For maximum generality, we therefore consider only time, *t* to time *t+1* drawing parallels to RNA velocity (PMID: 30089906). We note however this formulation does not specify the length of actual time elapsed between two timepoints. Therefore, to increase temporal coverage, one can perform autocorrelation analysis to set a minimal duration between timepoints, to have maximum temporal information (i.e. two near-identical video frames offer minimal additional information). In the analysis of mouse organoid branching in new Suppl. Fig. 3 b-d (Organoids paper, NCOMMS-23-61673C), we used every other frame, increasing the time duration between timepoints from 15 mins to 30 mins.

Our motion features is depicted in Fig 1c, 3rd column, Methods paper. We refer to "Motion as combined changes in the object's shape, appearance, and centroid between consecutive timepoints" because our motion features are derived from the computed optical flow ("the pattern of apparent motion of objects") between successive timepoints, and this calculation involves the image intensities (c.f. Farneback dense optical flow), producing a vector per pixel. Our motion features inherently consider rotation and other potential spatial variations of this vector field. Specifically, the same pattern rotated by X degrees, should produce similar features. Whereas ME-VAE uses averaging over explicit data transformation groups to obtain invariance, here in SAM-SPOT, as with O2-VAE, and SIFT, the invariance is built into the computation directly which uses i) magnitude of the vector fields, and ii) permutation-invariant operations like mean and histogram or iii) invariant features like SIFT (scale-invariant feature transform, *D.G.Lowe, "Object Recognition from Local Scale-Invariant Features" (1999)*).

Specifically, breaking it down:

- <u>Mean boundary and optical flow speed statistics</u> are invariant: using the magnitude and the mean which are permutation invariant.
- <u>Mean regional optical flow statistics and histograms</u> are invariant: bands are radial and therefore rotational-invariant, mean and histogram over speed are invariant operators (no orientational ambiguity).
- <u>Speed, Curl and Divergence SIFT features</u> are invariant: we use the scalar magnitude of these quantities as 'intensity images' and compute their SIFT features. SIFT features are well-known to be scale and rotation-invariant.

We note in revision, Fig. 1c (Methods paper, NCOMMS-25-25126A) did not explicitly specify 'curl magnitude' and 'divergence magnitude'. We have added additional clarifying texts to avoid any ambiguity.

Finally, a way to convince reviewers is to show an example of "rotation" and an example of "polar orientation" that is correctly detected using their method, and it is not confused and perceived as a change in shape.

Author's reply:

We thank the reviewer for suggesting the additional benchmarking, and the new results have resulted in improved ways of computing SAM features. All these are now included in our revised methods and discussion sections.

a) SAM/SPOT features are rotation and polar invariant:

As explained in our companion Methods paper NCOMMS-25-25126A, we construct SAM-SPOT features so that they are theoretically rotationally-invariant. In practice, there will be numerical deviations for example due to discretization, so that invariance is not exact. We expect SAM-SPOT features to be polar orientation invariant because inputs are centered before feature extraction. We don't expect mirror-invariance, and so if 180 degree rotations of an object is close to a mirrored version, this can break invariance numerically. Overall, we expect to be able to cluster all rotated instances of a unique object separate from rotations of a second unique object.

b) Benchmarking with MNIST handwriting dataset:

To test our claims empirically, we have performed additional benchmarks in the methods paper using the handwriting MNIST dataset, taking one image each for each of the 10 digits and generating rotationally perturbed, polar orientation perturbed (i.e. translating the digit) and left-right mirrored versions (Suppl. Fig. 1b, Methods paper NCOMMS-25-25126A). For these static datasets, we compute SA (shape-appearance) features and performed principal components analysis to visualize the clustering in 2D space. As can be seen, we correctly cluster all variations of the same digit of 0,1,2 as expected, all distinctly shaped, irrespective of perturbation. For the digit 2, we also see more variation and splitting of images rotated by +/- 90 degrees vs +/-270 degrees, as expected. Surprisingly, we found polar displaced images to exhibit greater feature variation than rotated images. We are not currently sure what is driving this. Across all 10 digits, PCA analysis retains grouping of digits where +/- 90 degree vs +/-270 degree rotations maintain uniqueness, but splitting of point-clouds for those that are more ambiguous like 2 and 5, 6 and 9, 3 and 7. If exact numerical rotational invariances are required, or invariance to other transformations are required, at the expense of cost users can perform geometrical transformations of each input, and average their individual SAM features to obtain a mean SAM feature, similar to the thinking of ME-VAE. Doing this for each digit, with 90 degree rotations (i.e. 4 augmentations per image input), we almost completely abolish the numerical inaccuracies. The rotated, flipped and polar translated points of each digit now all cluster tightly together, and the PCA clearly identifies one point-cloud per digit. Using 45 degree in addition to 90 degree rotations (i.e. 8 augmentations per image input) further increases the invariance, and tightness of clustering. Verifying this is an effective strategy to improve the effectiveness of our SAM features.

III. The authors state that "Motion as combined changes in the object's shape, appearance, and centroid between consecutive timepoints". Mathematically, thinking SAM should capture mutually exclusive features of change, but due to their definition of motion, it implies that motion does not capture a distinct feature. How do the authors support the sufficiency of their definition of the motion?

Author's reply:

We apologize for the confusion in the general language used. Mathematically, we regard motion as the deformation field relating the image intensity at time t to time t+1, as computed by optical flow and the translation displacement in centroids at time t to time t+1. Our motion features measure properties of the deformation field, namely its magnitude (speed) and to first-order, its decomposition into divergence and curl components (Helmholtz decomposition).

We don't enforce mutually exclusive features with shape and appearance, as the optical flow computation inevitably couples it to appearance and shape boundary. Meanwhile appearance should technically be measured independent of the shape, but this is non-trivial. Ideally, we could remap the texture into a fixed geometry like a circle, however any remapping of shape inevitably introduces distortion artefacts (also present in ME-VAE), and therefore distorted appearance. Moreover, these distortion artefacts are reflective of the original geometry. We are not currently aware of a technique which allow us to infinitely tile the texture without distortion. Finally, we could enforce mutual exclusivity of features after compiling of all phenomes e.g. through an orthogonal decomposition, and indeed our SAM module analysis which effectively groups mutually correlated features. However, we would then need some way to reassign modules as shape, appearance and motion which no longer may be 'pure' definitions. Currently, we should thus interpret the features as being derived from shape, appearance and motion considerations. Moving forwards, a next layer of analysis with the phenomes could be to derive SAM modes akin to orthogonal mode decomposition in shape analysis i.e. mutually exclusive joint shape-appearance-motion templates. We have added this to the discussion.

IV. How is it determined that rotation should always be ignored? Maybe a rotation is part of a meaningful motion? I am not sure about this but I put this as a question to be answered.

Author's reply:

We agree that rotation is a meaningful motion. Currently, our motion features do not explicitly compute the pure rotational motion as a feature. This decision was in part due to it not being clear how to include a rotation angle which is a 'directional' feature as a scalar feature to work with the rest of the SAM features. However, as described previously (see reply to 5.II), our motion features implicitly capture rotation and other potential spatial variations of the optical flow computed vector field. To demonstrate this we applied clockwise and anticlockwise 15 and 45 degree rotations to rotations of the same MNIST digits to simulate the next frame of each image. Suppl. Fig. 1c of our Methods paper NCOMMS-25-25126A shows that the motion field inferred by optical flow is dependent on the particular digit. We do not reconstruct a pure rotation, as optical flow is a non-rigid registration technique. Consequently, the PCA of the motion features primarily separates the digits, 0, 1, 2 and the magnitude of rotational motion is not well-differentiated. However, when we reperform the PCA with the motion features together with the shape and appearance features, we find separation of the digits, 0, 1, 2 and the emergence of a more consistent structure within each digit, where we find global directional alignment in PCA with increasing rotation magnitude i.e. a line joining the same image rotated +15 and +45 degree rotation, show the same direction as a line joining an image rotated -15 -to- 45 degree rotation. Thus, SPOT can distinguish the magnitude of rotational motion but not rotation orientation (clockwise (-) vs anticlockwise (+)). The substructure alignment is stronger when we further applied temporal order filtering in preprocessing the SAM features, as expected. To perform this filtering here, we regarded the rotated digits as part of a video with increasing time as rotation increased from 0-360 degrees. The substructure alignment was also stronger when we increase the invariance of shape-appearance features using the augmented SA features (4 rotations per image).

Future work will be needed to investigate how to reinstate the sign of the rotation and account for pure rotational and scaling motions i.e. we intend to introduce a more formal model decomposing motion into affine (translation, rotation, scale, shear) and non-rigid (optical flow after rigid-body registration) components.

V. The preprocessing contains removal of highly variant features "All dataset dependent preprocessing steps such as zero-valued and high variance features". I do not see why this is sensible. Conversely, I can see that high variance features are useful signals.

Author's reply:

We apologise for not making the point clear about zero-valued and high variance features. We have added the mathematical definition of a high variance feature and clarified the zero-valued features to Methods of the methods paper (NCOMMS-25-25126A).

We refer to a zero-valued feature as being zero across all object instances (i.e. all *N*). It therefore has zero variance, and zero information. We also test for potentially non-zero-valued features with zero variance across all object instance and therefore would also contribute zero information.

We agree with the reviewer that high-variance is indicative of signal. However, segmentation and tracking errors also contribute high variance e.g. an undersegmented cell will measure improbably low cell area, likewise a segmentation of two or more cells will measure significantly higher cell area. This will make this feature highly fluctuating such that we regard it as 'unreliable', and dominate the multidimensional analysis. To clarify, the variance criteria is applied to the standard deviation of the feature measured across all object instances i.e. a 'high variance' feature i is one where std(feat_i) >= mean(std(feat_i))_j + threshold_multiplier * std(std(feat_i))_j. This threshold measures variance across all features, and the threshold_multiplier is used to adjust the stringency of the cutoff as a multiplier of std. Typically, we consider this a normal variable and set threshold_multiplier to 2 or 3.

6. Is there enough detail provided in the methods for the work to be reproduced?

[Answer from the reviewer to the Editor] *Yes.*

Author's reply:

Thank you.

NCOMMS-23-61673C: Reviewer Supplementary Note

We validated the consistency of SPOT clustering and our interpretation of relative drug toxicity by reanalyzing the dataset with only DMSO (control), cisplatin, 5-fluorouracil and docetaxel representing low, mild and high toxicity groupings respectively. With reduced conditions, the reanalysis now finds two groups, grouping DMSO and Cisplatin as lower toxicity, than higher toxicity 5-fluorouracil and docetaxel (Reviewer Suppl. Fig. 3a). We then repeated the screen with these drugs and organoids derived from a second patient with multi-channel imaging of markers of cell death (Caspase 3/7 Green) and cell apoptosis (Cytotox Green) to verify phenotype clusters associate with biological processes (Reviewer Suppl. Fig. 3b). Using the SAM phenomes computed on brightfield images only, SPOT generated a phenomic landscape colocalizing organoids with high Caspase and Cytotox expression in the same region (Reviewer Suppl. Fig. 3b b,e). SPOT identified phenotype clusters with the highest Caspase and Cytotox expression (Reviewer Suppl. Fig. 3b g) and these also had the predicted SAM properties (darker lumen, reduced movement) (Reviewer Suppl. Fig. 3b h). Moreover, unlike biomarkers, the clusters identified heterogeneity associated with two potential sources of cell death: i) “normal" death when organoids grow too big (cluster 5), which we labelled as growth phenotype as it reflects lumen darkening due to organoid growth, or ii) premature drug-induced death (toxic phenotype) whereby organoids were already dead or died at early timepoints without undergoing significant growth (clusters 7) (Reviewer Suppl. Fig. 3b h). Lastly, clustering of phenotype trajectories showed the same grouping of relative toxicity: DMSO and cisplatin, 5-fluorouracil and docetaxel. In contrast, mean cytotox expression ranks 5-fluorouracil and docetaxel lowest in toxicity, and caspase expression only identified docetaxel being of higher toxicity. The same conclusions were found analyzing flat-field corrected video frames with reduced background fluorescence intensity in caspase and cytotox (Reviewer Suppl. Fig. 3c). Comparing and aligning the individual outputs of both analyses (Methods) highlights the consistency of SPOT analysis (Reviewer Suppl. Fig. 3d,e). We detect almost identical numbers of organoid instances; UMAP landscape shape and organization of image patches; the same number of phenotype clusters with similar SAM characteristics; caspase, cytotox expression; cluster stacked barplots; cluster transitions; phenotype trajectory directions; and a similar number of SAM modules with

the same expression pattern across phenotype clusters. We further reperformed the screen with organoids derived from a third patient. The comprehensiveness of our full SAM phenome allowed automated detection and removal of bubble and blank segmented imaging artefacts before SPOT analysis (Reviewer Suppl. Fig. 3f), whereas a select set of four SAM features: area, eccentricity, intensity and speed could not (Reviewer Suppl. Fig. 3g). Despite the imaging being less in-focus and organoids less dynamic, SPOT recovered the same relative drug toxicity and similar individual analysis outputs, whilst caspase and cytotox expression analyses were similarly inconclusive (Reviewer Suppl. Fig. 3h,i). Naturally, the degree of similarity was less than flat-field correction of the same patient (Reviewer Suppl. Fig. 3d), and the UMAP embedded images were dissimilar (Reviewer Suppl. Fig. 3j). To independently verify drug toxicity, we computed the mean normalized organoid growth rate metric[32] (NOGR) of each drug across plates of both patients, using DMSO and docetaxel as the negative and positive cytotoxic effect references (Reviewer Suppl. Fig. 3k). NOGR ranks the drugs with same relative toxicity as SPOT and as predicted by SPOT, identified 5-fluorouracil and docetaxel as cytotoxic and growth-arresting (NOGR<0, Methods). NOGR scores also highlighted per plate ‘batch-effect’ differences. In one plate, 5-fluorouracil and docetaxel were not cytotoxic (NOGR>0).

We performed these screens with organoids cultured in optically clear glass-bottom plates (Suppl. Table 3). To highlight the effect of batch differences, and strategies to compensate for it in SPOT analysis, we also performed a like-for-like comparison with organoids cultured in standard transparent, plastic plates. Independent SPOT analyses find the same relative drug toxicity (Reviewer Suppl. Fig. 3l) but differed by one in the number of identified phenotype clusters (Reviewer Suppl. Fig. 3m c). The six matching clusters exhibit similar mean SAM expression patterns (Reviewer Suppl. Fig. 3m d), but showed a greater degree of similarity stacked barplots (Reviewer Suppl. Fig. 3m e) and cluster transitions (Reviewer Suppl. Fig. 3m f). Additionally, we detected the fewest SAM modules with glass plates and consequently recovered the fewest number of shared modules (Reviewer Suppl. Fig. 3m h) compared with background subtraction (Reviewer Suppl. Fig. 3d) or biological replication (Reviewer Suppl. Fig. 3i). Image embeddings highlight a different local spatial arrangement of SAM features in the aligned UMAP (Reviewer Suppl.

Fig. 3n), further confirmed by a pooled UMAP analysis of optical-bottom and plastic plates (Reviewer Suppl. Fig. 3o). To investigate whether the batch effects were plate-based or specific to the plastic-bottom plates we further performed a pooled UMAP analysis across all plates with the four drugs (Reviewer Suppl. Fig. 3p, see Suppl. Table 3 for metadata). The results show that batch effects are primarily plate-to-plate differences in the statistical distribution of SAM characteristics. These variations do not affect drug clustering using the concatenation of individual plate trajectories (Reviewer Suppl. Fig. 3q a), but induce correlation between plates as evidenced by the pairwise affinity matrix (Reviewer Suppl. Fig. 3q b). Ideally, organoids from each plate are independent and identically distributed (i.i.d). We found normalizing SAM phenomes within each plate, instead of globally after pooling, to be a simple strategy to restore i.i.d. Reperforming the pooled UMAP analysis with per-plate normalized SAM phenomes improved the colocalization of UMAP densities of organoids across individual plates treated with the same drug, and reduced the correlation between different plates (Reviewer Suppl. Fig. 3r,s).

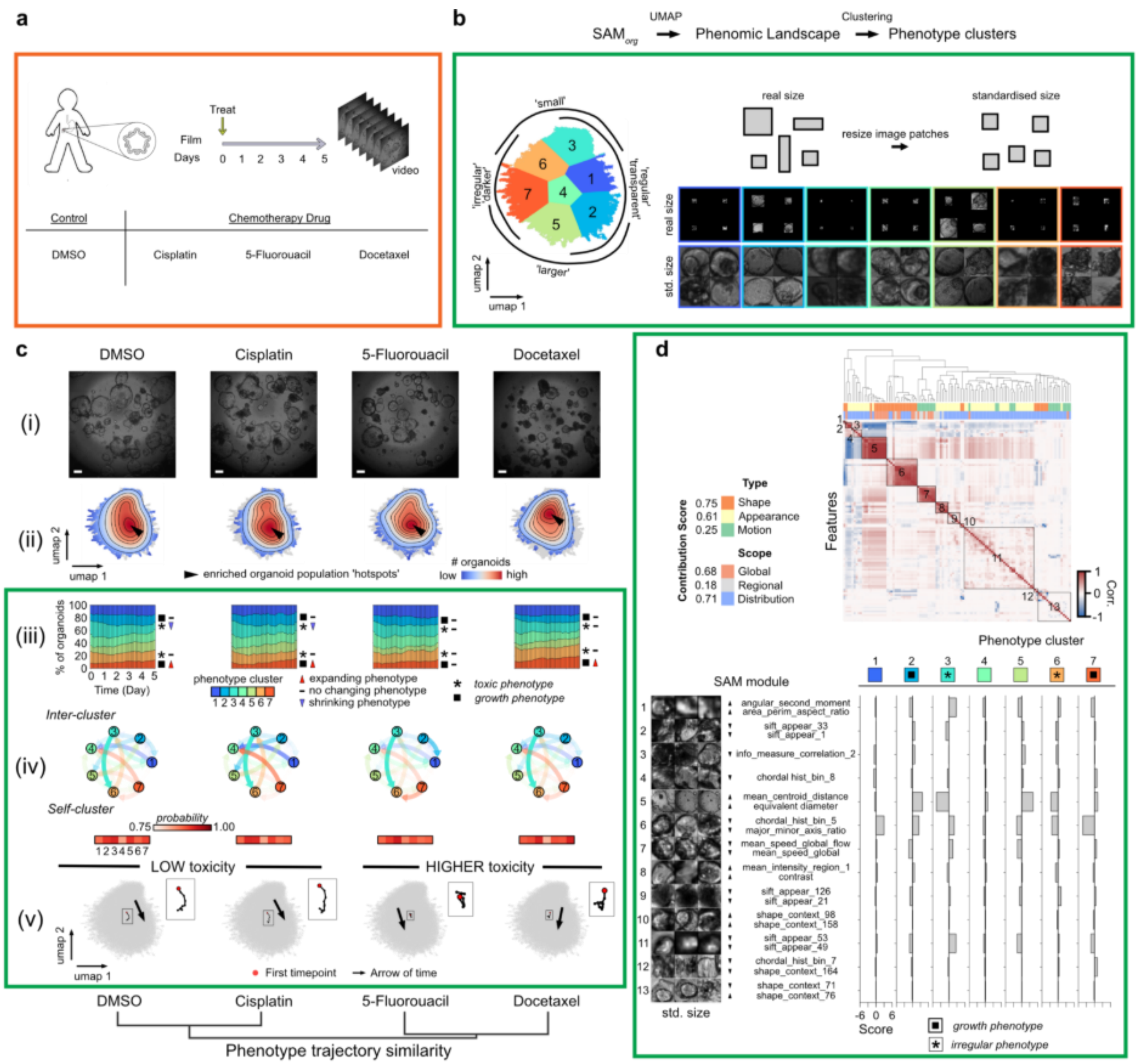

Reviewer Supplementary Figure 3a. SPOT analysis clustering of drug response of human patient-derived duodenum organoids is consistent when analyzed with reduced number of drugs. a) Same patient-derived D2 duodenum organoids grown from endoscopy biopsies, in a 96-well plate with the specified drug treatment and concentration, and filmed every hour for 5 days without labelling as in Fig. 3 for four of the drug conditions: DMSO control, and cisplatin, 5-fluorouacil and docetaxel, each representing low, mild and high toxicity as determined in Fig. 3c. **b)** UMAP phenomic landscape, phenotype clusters and representative cluster images as in Fig. 3 using the filtered SAM phenomes computed with this subset of the full dataset, n=281,600 points. **c)** Hierarchically clustered chemotherapy drugs according to SAM temporal phenotype trajectories using average linkage (bottom). (i) final frame of exemplar videos for each drug treatment, scalebar: 250 µm; (ii) UMAP point density heatmaps; (iii) stacked barplots; (iv) inter-cluster relative probability transition graphs (top), and self-cluster transition probability heatmaps (bottom); and (v) UMAP SAM phenotype trajectories for each drug, as in Fig. 3c. **d)** Contribution scores of shape, appearance and motion, and global, regional and distributional features, and

automated hierarchical clustering to identify SAM feature modules (top row) and SAM module expression in each phenotype cluster (bottom) with representative image exemplars and top driving features for each SAM module, constructed as in Fig. 3d.

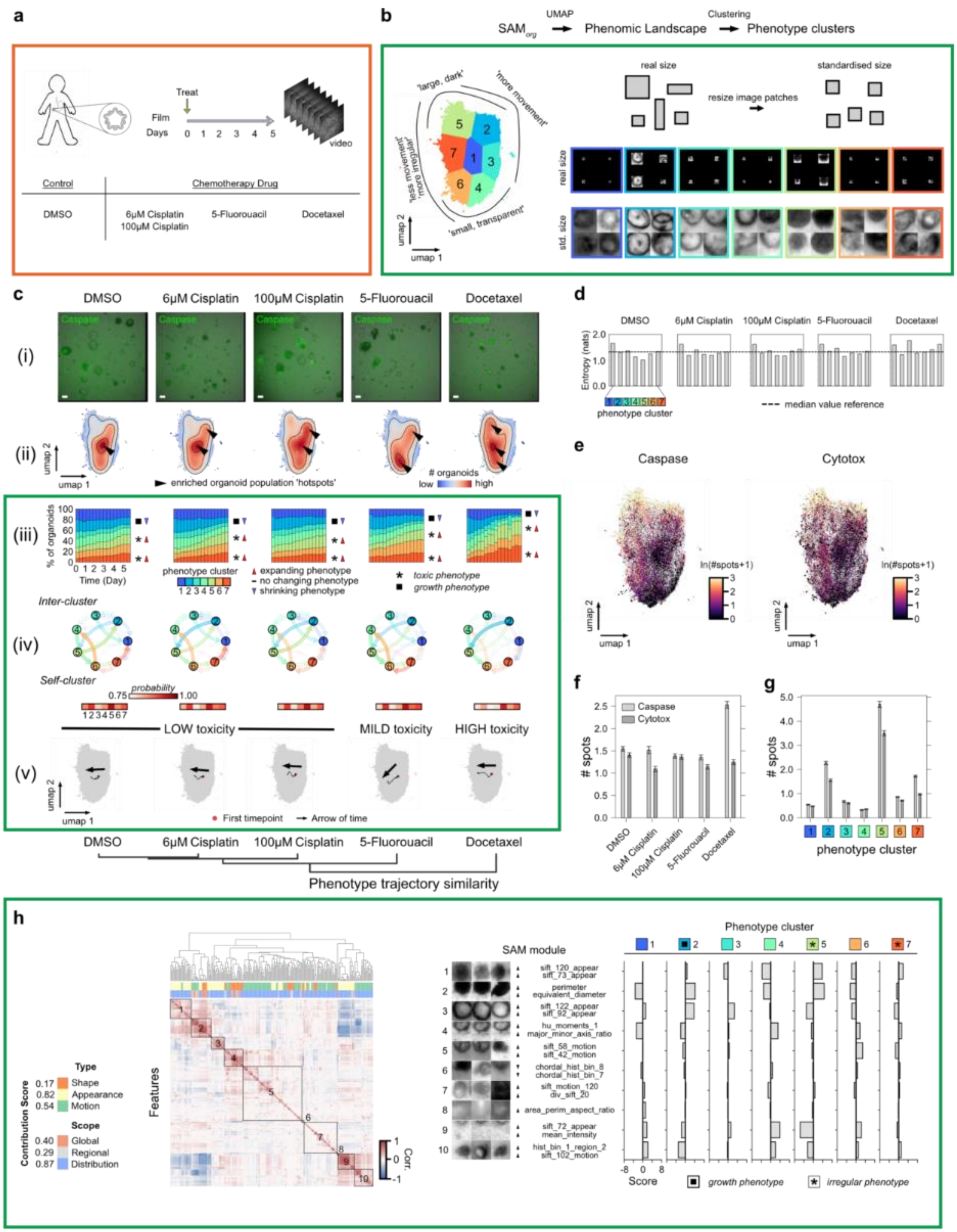

Reviewer Supplementary Figure 3b. SPOT phenotype clusters capture different SAM characteristics of cell death after drug treatment. a) Patient-derived D2 duodenum organoids labeled with Hoescht and additional Caspase 3/7 Green or Cytotox Green were grown from

endoscopy biopsies, in a 96-well plate with the specified drug treatment and concentration, then filmed every 4 hours for 5 days. Drugs without concentration specified used the corresponding maximum concentration in Fig. 3. (Methods). **b)** UMAP phenomic landscape, phenotype clusters and representative cluster images as in Fig. 3 using the filtered SAM phenomes computed from only the brightfield channel from all videos across all drugs and concentrations, n=70,757 points. **c)** Hierarchically clustered chemotherapy drugs according to SAM temporal phenotype trajectories using average linkage (bottom). (i) final frame combined with Caspase 3/7 Green channel of exemplar videos for each indicated drug treatment and drug concentration, scalebar: 250 μm; (ii) UMAP point density heatmaps, same UMAP coordinates as in (b); (iii) stacked barplots showing the temporal changes in phenotype cluster (as characterized in (c)) distribution for each drug, as computed from organoids at 8-hour time intervals; (iv) graphs showing the probabilities of an organoid transitioning to another phenotype cluster (inter-cluster, top) or maintain the same phenotype cluster (self-cluster, bottom) label in the next timepoint (Methods). Arrows are colored by the source cluster. The more transparent the arrow, the smaller the probability of the transition. (v) UMAP SAM phenotype trajectories for a density threshold of mean+3 standard deviations (left) and zoom of the trajectory (right) for each drug. On each trajectory, each black point denotes 8.5-hour increments from the origin red point. A black arrow indicates the directionality of time. **d)** Shannon entropy of the inter-cluster next timepoint transition probability for each phenotype cluster, in each drug condition. **e)** UMAP with points coloured by its number of counted punctate Caspase 3/7 (left) and Cytotox (right) spots. **f)** Mean$\pm$s.e.m number of punctate Caspase 3/7 and Cytotox spots of organoids in each drug condition. **g)** Mean$\pm$s.e.m number of punctate Caspase 3/7 and Cytotox spots of organoids in each phenotype cluster. **h)** Contribution score of shape, appearance and motion, and global, regional and distributional features (left), automated hierarchical clustering to identify SAM feature modules (middle) and SAM module expression in each phenotype cluster (right) constructed as in Fig. 2f.

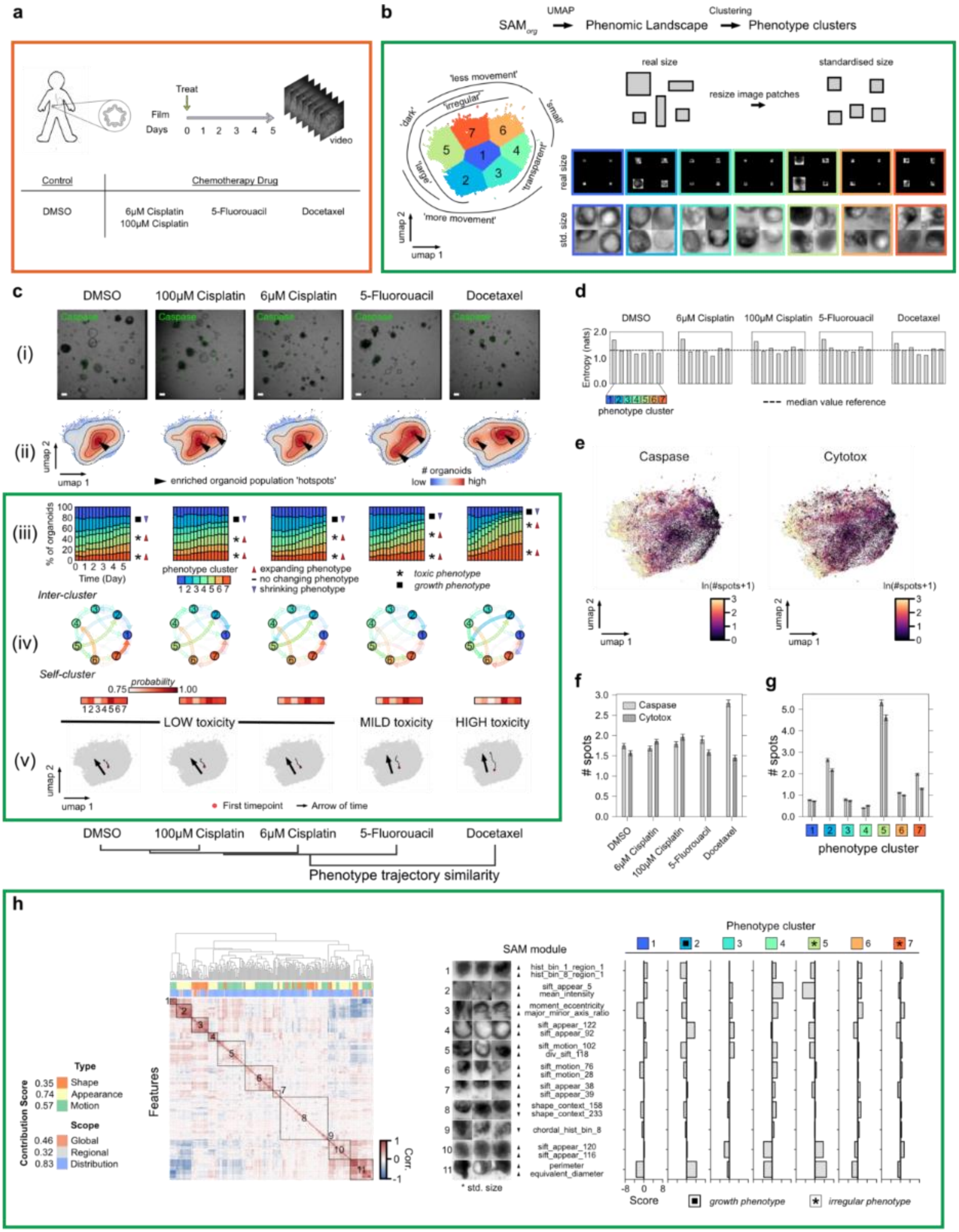

Reviewer Supplementary Figure 3c. SPOT reanalysis of Reviewer Suppl. Fig. 3b of the same patient-derived organoids with background subtracted videos. a) Same patient-

derived D2 duodenum organoids labeled with Hoescht and additional Caspase 3/7 Green or Cytotox Green as in Reviewer Suppl. Fig. 3b, but with images of each channel preprocessed by flatfield background subtraction. **b)** UMAP phenomic landscape, phenotype clusters and representative cluster images as in Reviewer Suppl. Fig. 3a using the filtered SAM phenomes computed from only the background corrected brightfield channel from all videos across all drugs and concentrations, n=70,757 points. Resulting **c)** hierarchically clustered chemotherapy drugs of the SAM temporal phenotype trajectories using average linkage (bottom). (i) background corrected final frame; (ii) UMAP point density heatmaps; (iii) stacked barplots; (iv) next timepoint inter-cluster and self-cluster transition probability graphs; (v) UMAP SAM phenotype trajectories. **d)** Shannon entropy of the inter-cluster next timepoint transition probability for each phenotype cluster, in each drug condition. **e)** UMAP with points coloured by its number of counted punctate Caspase 3/7 (left) and Cytotox (right) spots. **f)** Mean$\pm$s.e.m number of punctate Caspase 3/7 and Cytotox spots of organoids in each drug condition. **g)** Mean$\pm$s.e.m number of punctate Caspase 3/7 and Cytotox spots of organoids in each phenotype cluster. **h)** Contribution score of shape, appearance and motion, and global, regional and distributional features (left), automated hierarchical clustering to identify SAM feature modules (middle) and SAM module expression in each phenotype cluster (right) constructed as in Fig. 2f.

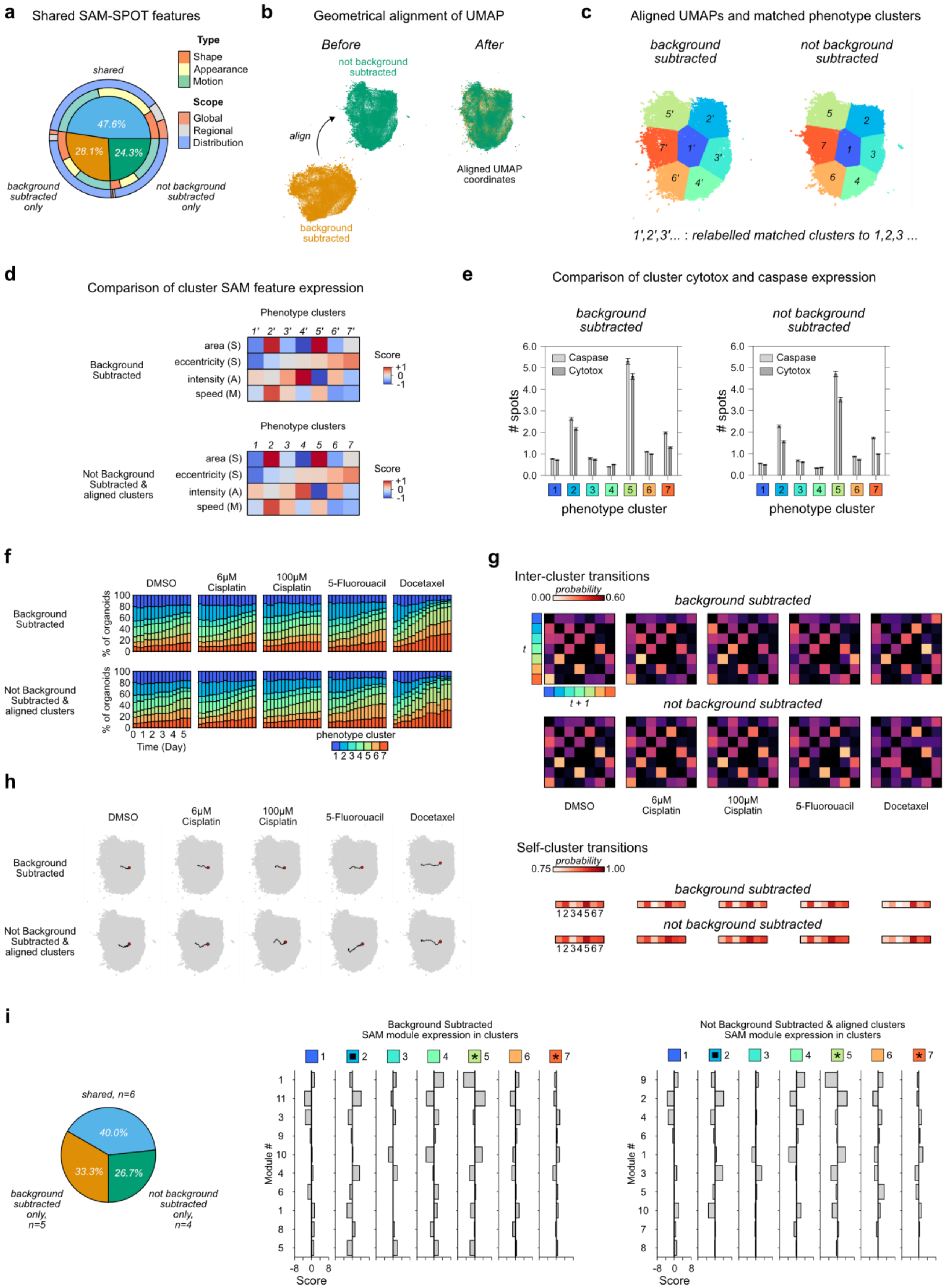

a Shared SAM-SPOT features
b Geometrical alignment of UMAP
c Aligned UMAPs and matched phenotype clusters
d Comparison of cluster SAM feature expression
e Comparison of cluster cytotox and caspase expression
f
g Inter-cluster transitions
Self-cluster transitions
h
i

Reviewer Supplementary Figure 3d. Reproducibility analysis of SPOT analyzed Reviewer Suppl. Fig 3b, 3c of the same patient organoids without and with video background subtraction. a) Piechart of the proportion of features that are unique or shared in the two sets of filtered SPOT-SAM phenomes. Outer two rings illustrate the composition of shape, appearance and motion features, and global, regional and distribution features in each of the unique and shared fractions. **b)** Geometrical alignment by rigid body rotation of the two independent UMAP phenomic landscapes. **c)** Relabeling of the phenotype clusters of the SPOT analyzed Reviewer Suppl. Fig 3b after matching the clusters to those of Reviewer Suppl. Fig 3c by their median UMAP coordinates. The relabeled integer cluster ids are denoted by an additional dash ('). **d)** Heatmap of the normalized mean expression score of selected SAM characteristics in each matched phenotype cluster for the not background subtracted (top) and background subtracted (bottom) analysis. **e)** Mean $\pm$ s.e.m number of punctate Caspase 3/7 and Cytotox spots of organoids in each matched phenotype cluster. **f)** Stacked barplots of matched phenotype clusters. **g)** Inter-cluster (top) and self-cluster (bottom) transition probabilities depicted as a heatmap with matched phenotype clusters for each drug condition. **h)** Phenotypic trajectory for each drug condition after UMAP alignment. **i)** SAM module analysis with phenotype cluster alignment. Piechart of the proportion of SAM modules found that are unique or shared (left). Shared is defined as the matched modules sharing more than a quarter of the same features. Mean expression of matched SAM modules in each of the matched phenotype clusters between the two analyses. SAM modules are ordered top-to-bottom by the extent of shared features in the not background subtracted (middle), and background subtracted (right) SPOT analyses. SAM module numbers correspond to those in the respective original SPOT analyses.

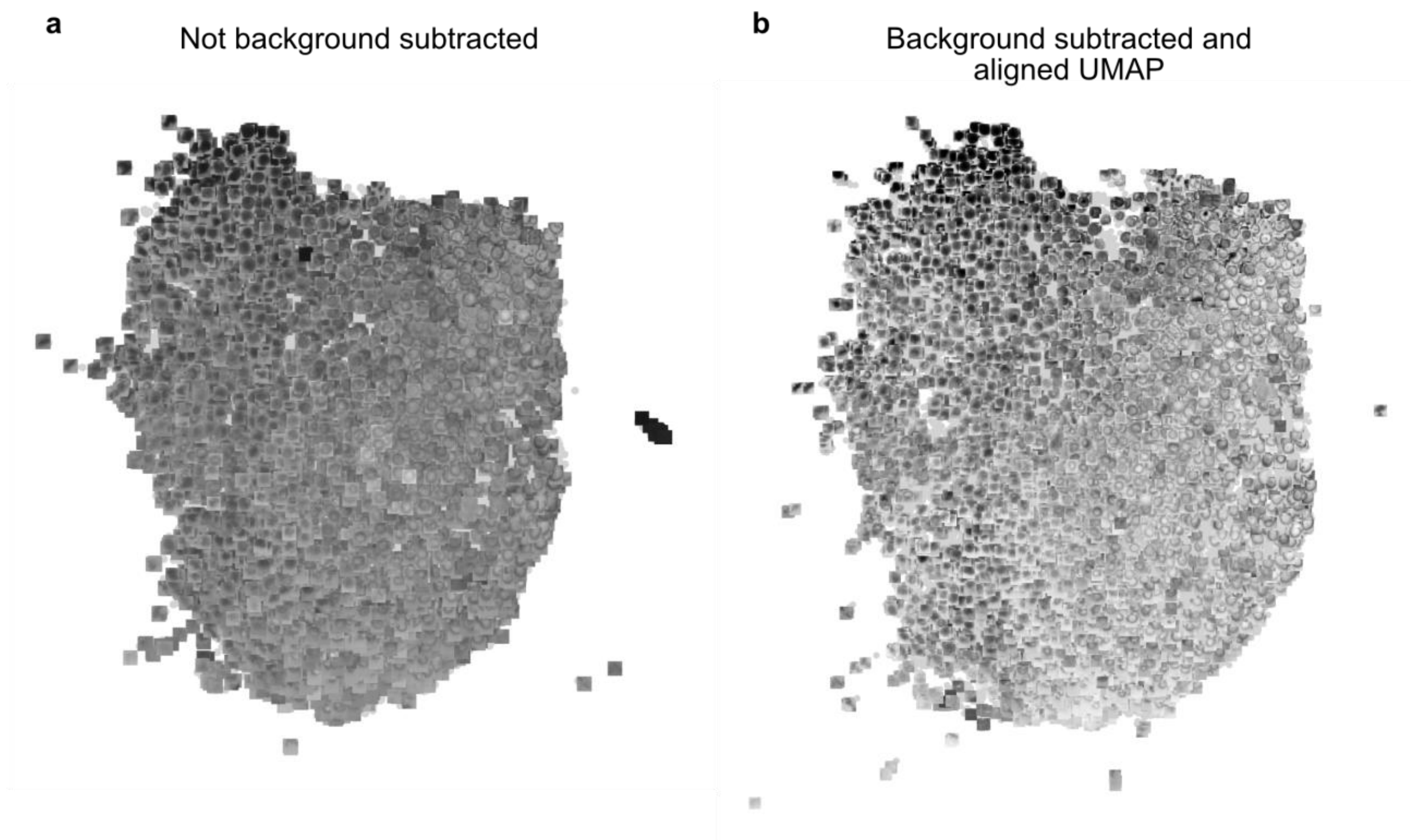

sReviewer Supplementary Figure 3e. Comparison of image patches embedded at the SPOT UMAP coordinates of Reviewer Suppl. Fig. 3b, 3c after UMAP alignment. a) Organoid image patches of Reviewer Suppl. Fig. 3b plotted at SPOT analyzed UMAP coordinates with not background subtracted video frames. **b)** Organoid image patches of Reviewer Suppl. Fig. 3c plotted at the aligned SPOT analyzed UMAP coordinates with background subtracted video frames.

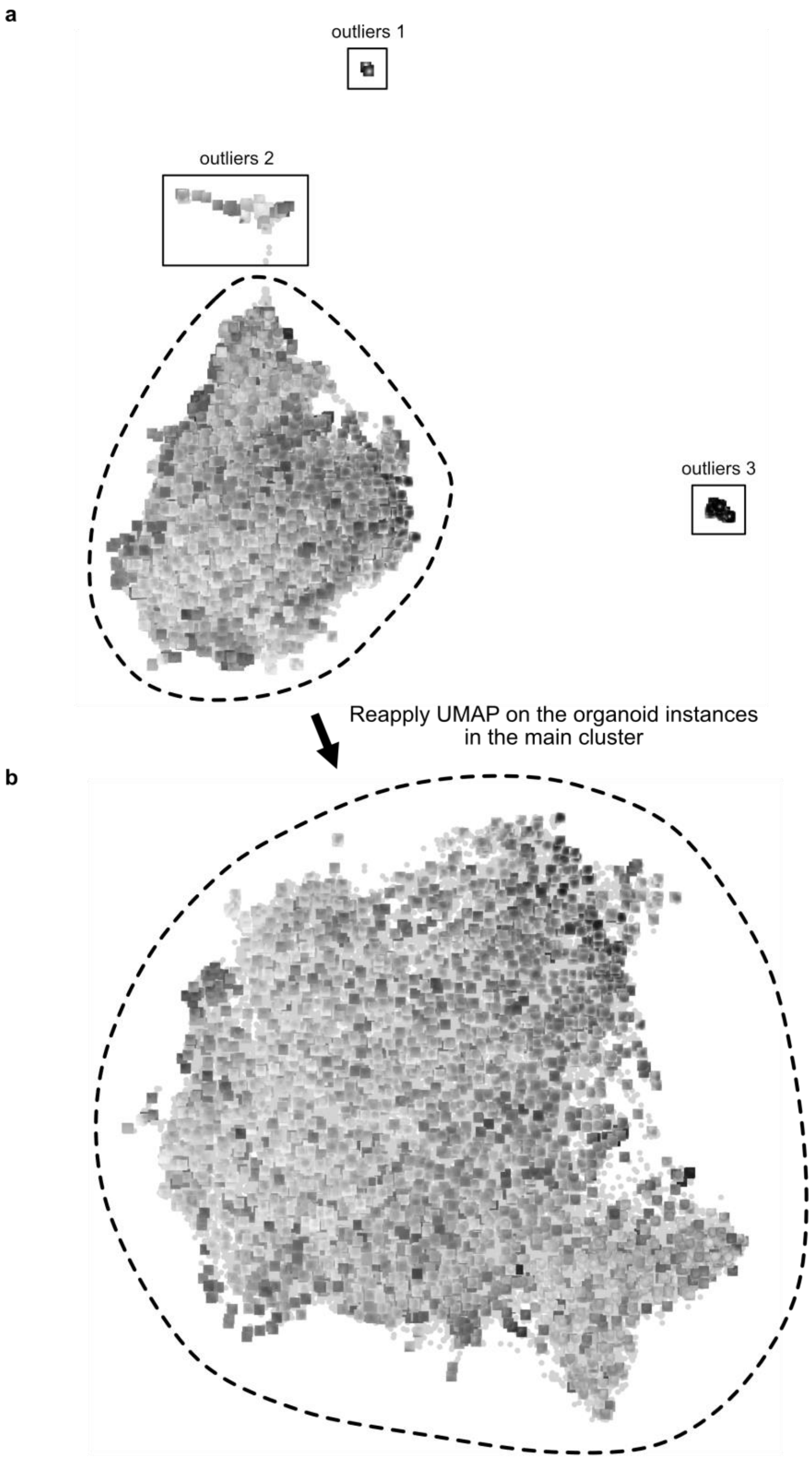

Reviewer Supplementary Figure 3f. SAM phenome allows UMAP detection of outlier artefact phenotypes for removal before analysis. a) UMAP phenomic landscape on all detected organoid instances highlights three isolated outlier groups. Outliers 1 and 3 are air bubbles. Outliers 2 are blank patches of nonuniform illumination. **b)** UMAP phenomic landscape on all retained organoid instances after removing the three outlier groups.

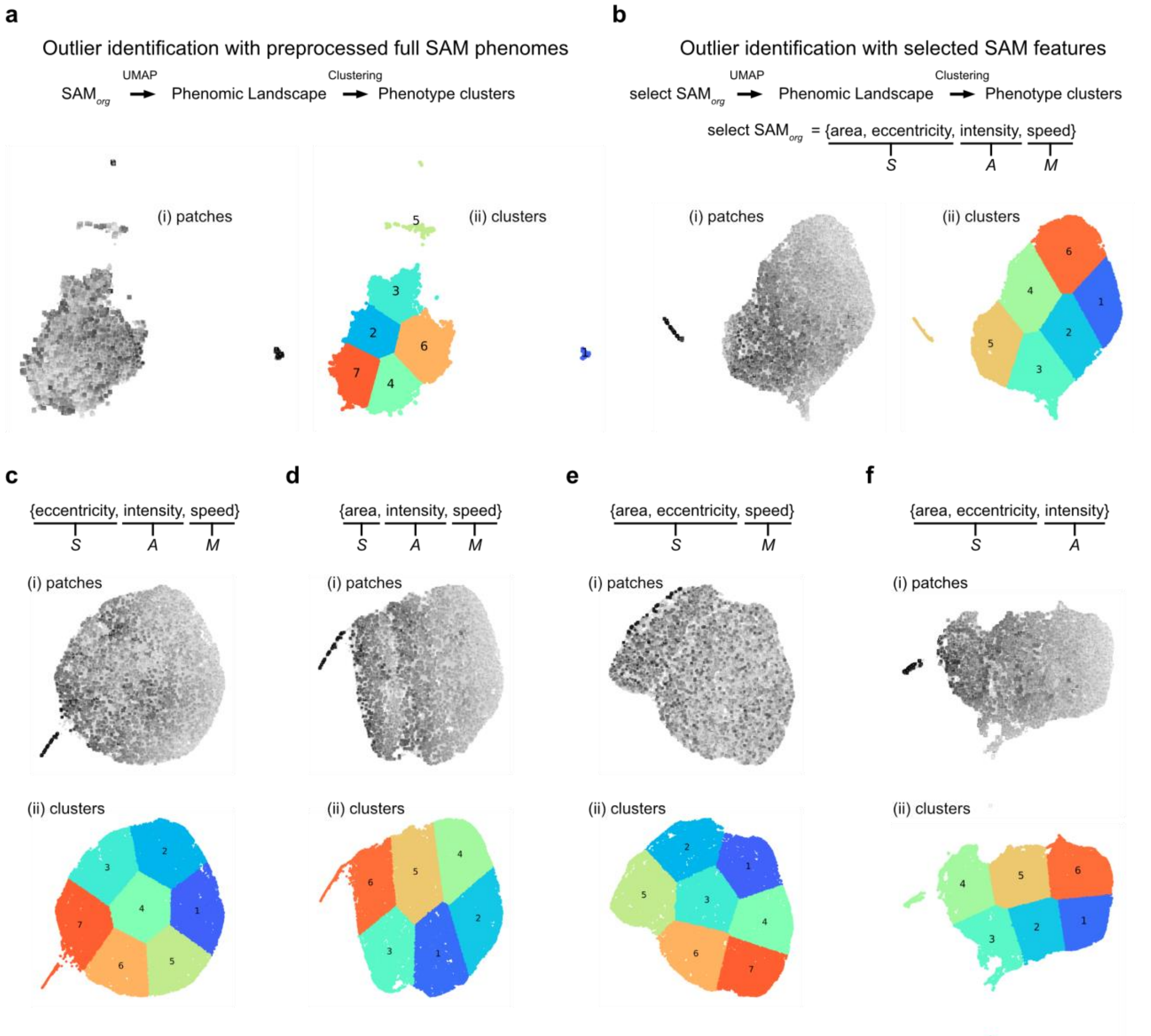

Reviewer Supplementary Figure 3g. Selected global SAM features do not enable automatic UMAP detection of outlier artefact phenotypes for removal before analysis. a) UMAP phenomic landscape with SAM phenome (i) plotting image patches and (ii) coloring points by phenotype cluster. Outliers are detected and isolated as clusters 1,5. **b)** UMAP phenomic landscape with a selected SAM phenome concatenating global shape (S) features: area, eccentricity; appearance (A) features: mean intensity; and motion (M) features: mean speed, (i) plotting image patches and (ii) coloring points by the resultant phenotype clusters, (ii). Phenomic landscape using the same restricted SAM phenome of four features in b), without area, **c)**; without eccentricity, **d);** without mean intensity, **e);** and without speed, **f)**, (i) plotting image patches, and (ii) coloring points by phenotype cluster. In none of b)-f) did kmeans clustering isolate outliers in one or more exclusive clusters.

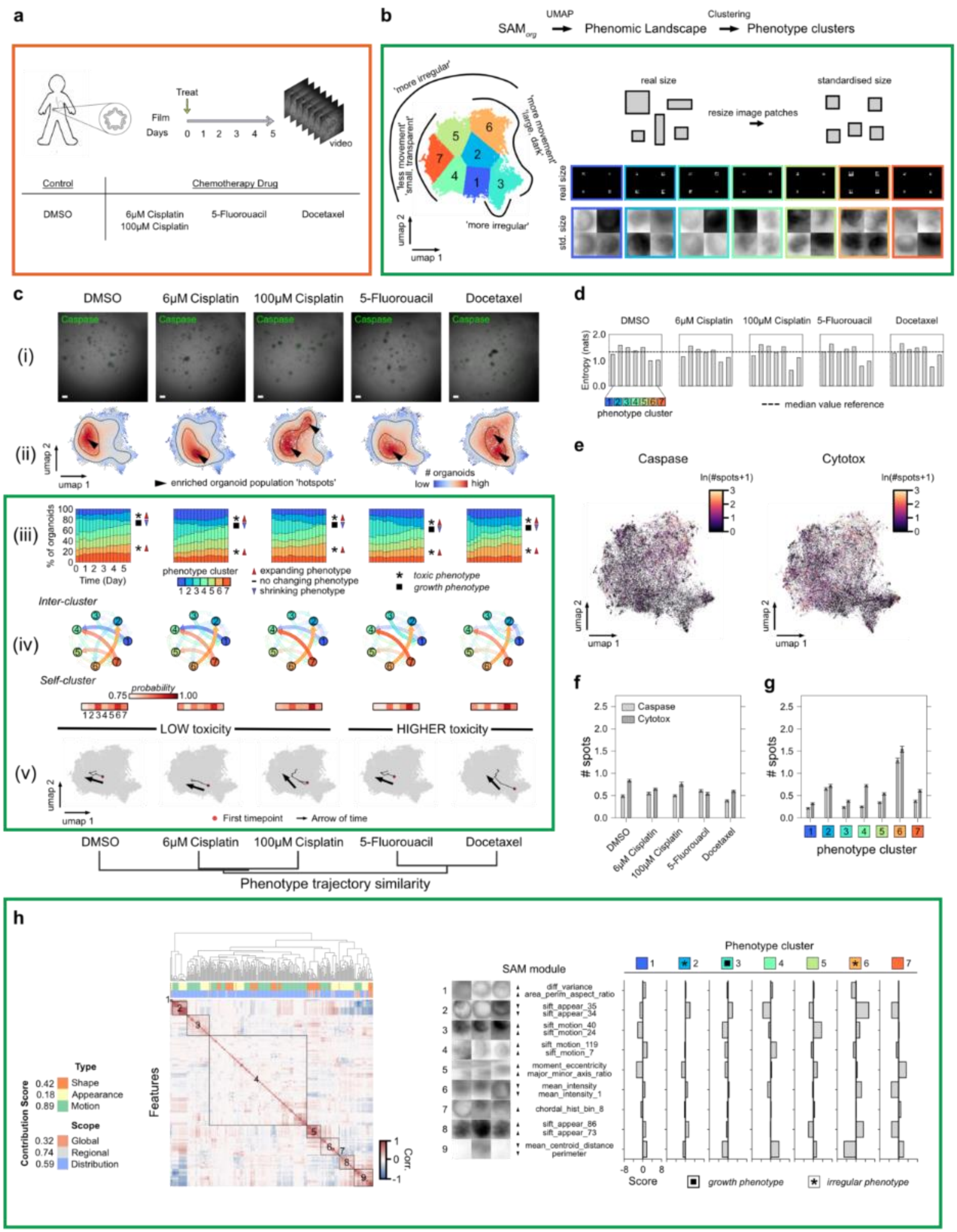

Reviewer Supplementary Figure 3h. Same SPOT analysis as in Reviewer Suppl. Fig. 3b on organoids derived from a second patient without video background subtraction. a) Patient-derived D2 duodenum organoids as in Reviewer Suppl. Fig. 3b from a second patient, labeled

with Hoescht and Caspase 3/7 Green or Cytotox Green. **b)** UMAP phenomic landscape, phenotype clusters and representative cluster images as in Reviewer Suppl. Fig. 3b using the filtered SAM phenomes computed from only the background corrected brightfield channel from all videos across all drugs and concentrations, n=39,057 points. Resulting **c)** hierarchically clustered chemotherapy drugs of the SAM temporal phenotype trajectories using average linkage (bottom). (i) Final frame; (ii) UMAP point density heatmaps; (iii) stacked barplots; (iv) next timepoint inter-cluster and self-cluster transition probability graphs; (v) UMAP SAM phenotype trajectories. **d)** Shannon entropy of the inter-cluster next timepoint transition probability for each phenotype cluster, in each drug condition. **e)** UMAP with points colored by its number of counted punctate Caspase 3/7 (left) and Cytotox (right) spots. **f)** Mean$\pm$s.e.m number of punctate Caspase 3/7 and Cytotox spots of organoids in each drug condition. **g)** Mean$\pm$s.e.m number of punctate Caspase 3/7 and Cytotox spots of organoids in each phenotype cluster. **h)** Contribution score of shape, appearance and motion, and global, regional and distributional features (left), automated hierarchical clustering to identify SAM feature modules (middle) and SAM module expression in each phenotype cluster (right) constructed as in Fig. 2f.

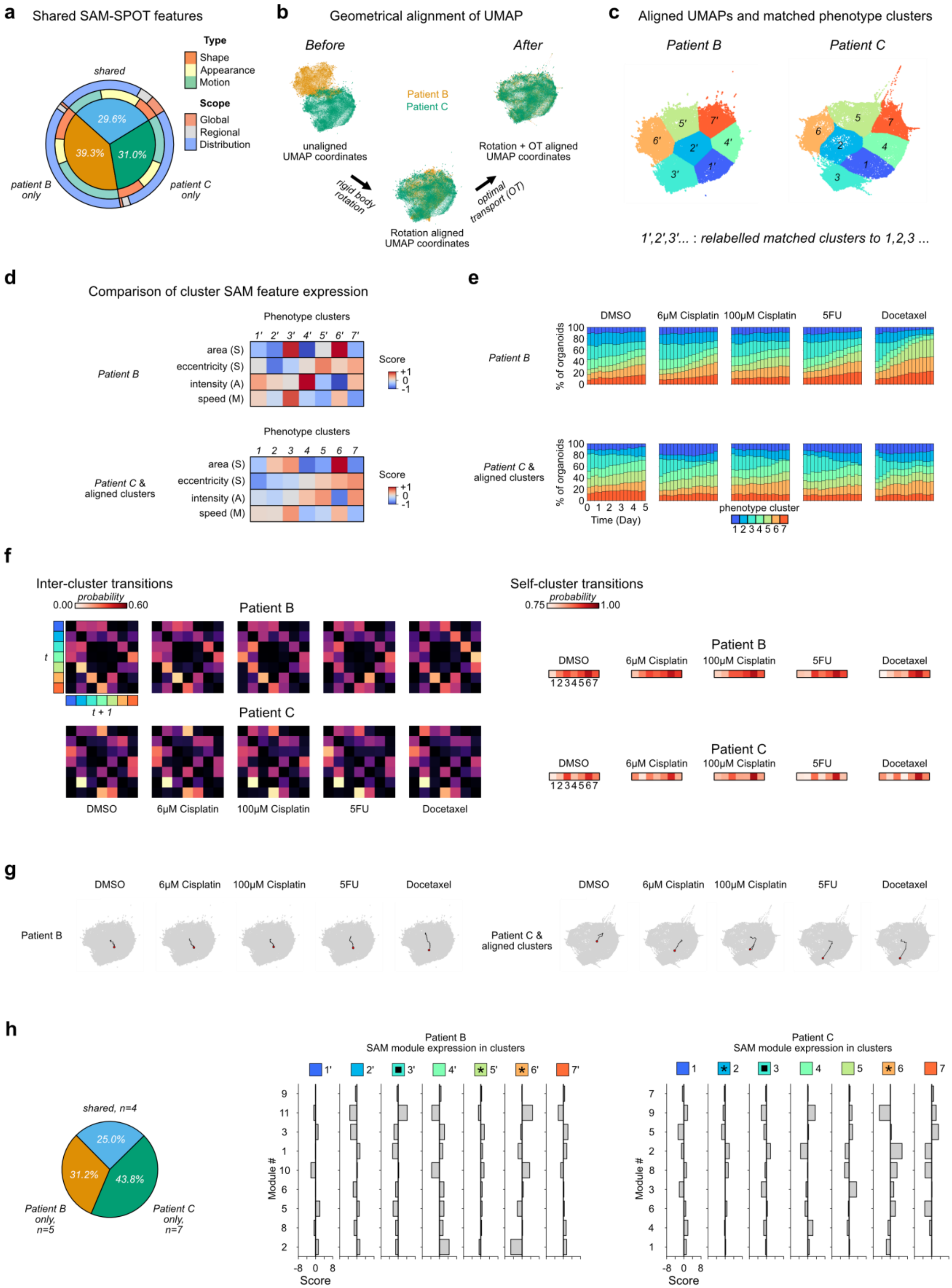
a
Shared SAM-SPOT features
Type
Shape
Appearance
Motion
Scope
Global
Regional
Distribution
shared
29.6%
39.3%
31.0%
patient B only
patient C only
b
Geometrical alignment of UMAP
Before
After
Patient B
Patient C
unaligned UMAP coordinates
Rotation + OT aligned UMAP coordinates
rigid body rotation
optimal transport (OT)
Rotation aligned UMAP coordinates
c
Aligned UMAPs and matched phenotype clusters
Patient B
Patient C
1′,2′,3′... : relabelled matched clusters to 1,2,3 ...
d
Comparison of cluster SAM feature expression
Phenotype clusters
Patient B
area (S)
eccentricity (S)
intensity (A)
speed (M)
Score
Patient C & aligned clusters
e
DMSO
6μM Cisplatin
100μM Cisplatin
5FU
Docetaxel
% of organoids
Time (Day)
phenotype cluster
f
Inter-cluster transitions
probability
0.00
0.60
Patient B
Patient C
t
t + 1
Self-cluster transitions
0.75
1.00
g
Patient B
Patient C & aligned clusters
h
shared, n=4
25.0%
31.2%
43.8%
Patient B only, n=5
Patient C only, n=7
Patient B SAM module expression in clusters
Patient C SAM module expression in clusters
Module #
Score

Reviewer Supplementary Figure 3i. Reproducibility analysis of SPOT analyzed Reviewer Suppl. Fig 3b, and 3h with organoids derived from two independent patients. a) Pie chart of the proportion of features that are unique or shared in the two sets of filtered SPOT-SAM phenomes. Outer two rings illustrate the composition of shape, appearance and motion features, and global, regional and distribution features in each of the unique and shared fractions. **b)** Geometrical alignment by rigid body rotation and optimal transport of the two independent UMAP phenomic landscapes. **c)** Relabeling of the phenotype clusters of the SPOT analyzed Reviewer Suppl. Fig 3b (Patient B) after matching the clusters to those of Reviewer Suppl. Fig 3h (Patient C) by their median UMAP coordinates. The relabeled integer cluster IDs are denoted by an additional dash (’). **d)** Heatmap of the normalized mean expression score of selected SAM characteristics in each matched phenotype cluster for Patient B (top) and Patient C (bottom) analysis. **e)** Stacked barplots of matched phenotype clusters. **f)** Inter-cluster (left) and self-cluster (right) transition probabilities depicted as a heatmap with matched phenotype clusters for each drug condition. **g)** Phenotypic trajectory for each drug condition after UMAP alignment for Patient B (left) and Patient C (right). **h)** SAM module analysis with phenotype cluster alignment. Pie chart of the proportion of SAM modules found that are unique or shared (left). Shared is defined as the matched modules sharing more than a quarter of the same features. Mean expression of matched SAM modules in each of the matched phenotype clusters between the two analyses. SAM modules are ordered top-to-bottom by the extent of shared features in the Patient B (middle), and Patient C (right) SPOT analyses. SAM module numbers correspond to those in the respective original SPOT analyses.

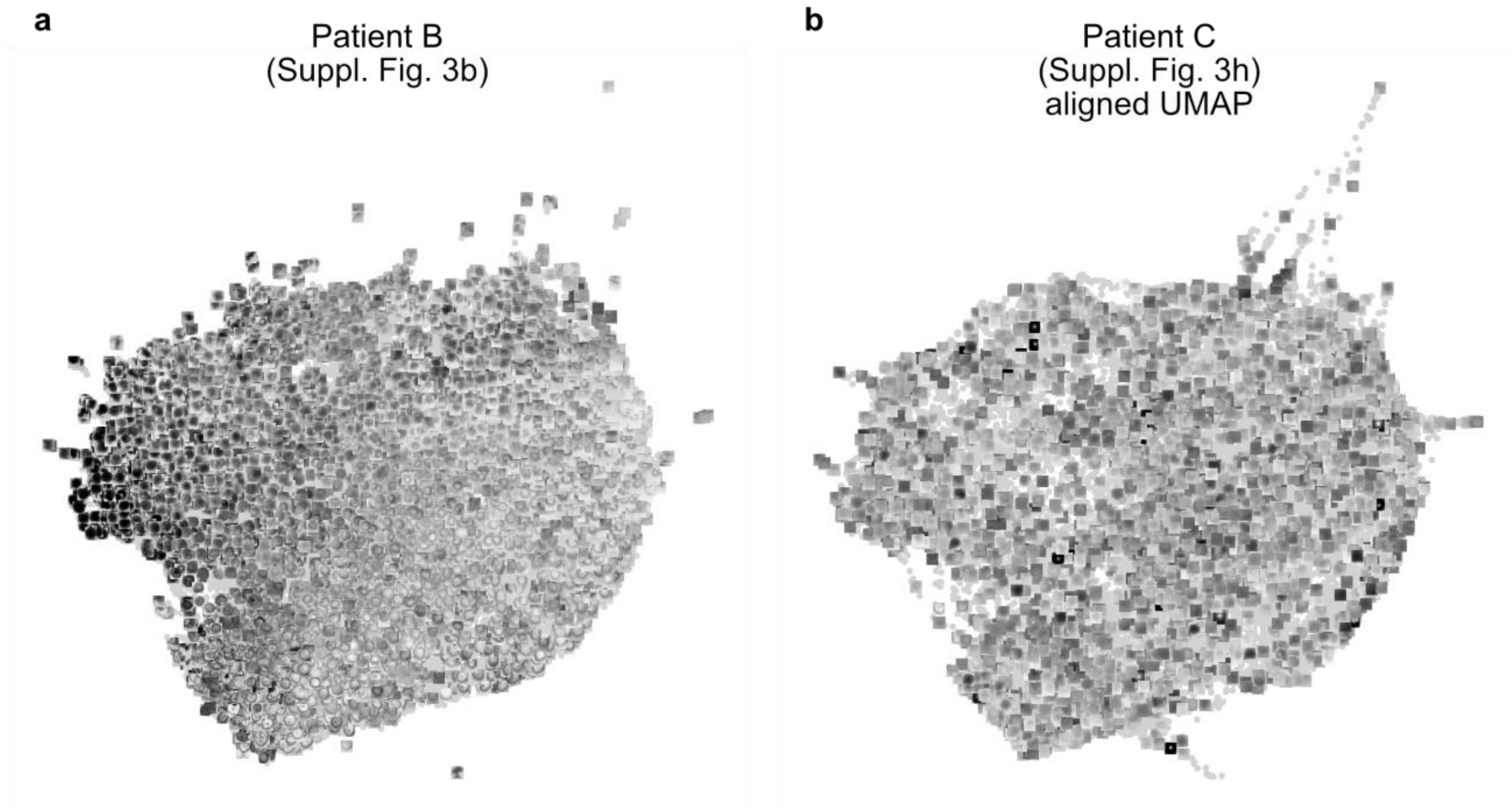

Reviewer Supplementary Figure 3j. Comparison of image patches embedded at the SPOT UMAP coordinates of Reviewer Suppl. Fig. 3b,f between two different patients after alignment. a) Organoid image patches plotted at SPOT analyzed UMAP coordinates with background subtracted video frames of Patient B (Reviewer Suppl. Fig. 3b). **b)** Organoid image patches plotted at the aligned SPOT analyzed UMAP coordinates and not background subtracted video frames of Patient C (Reviewer Suppl. Fig. 3h).

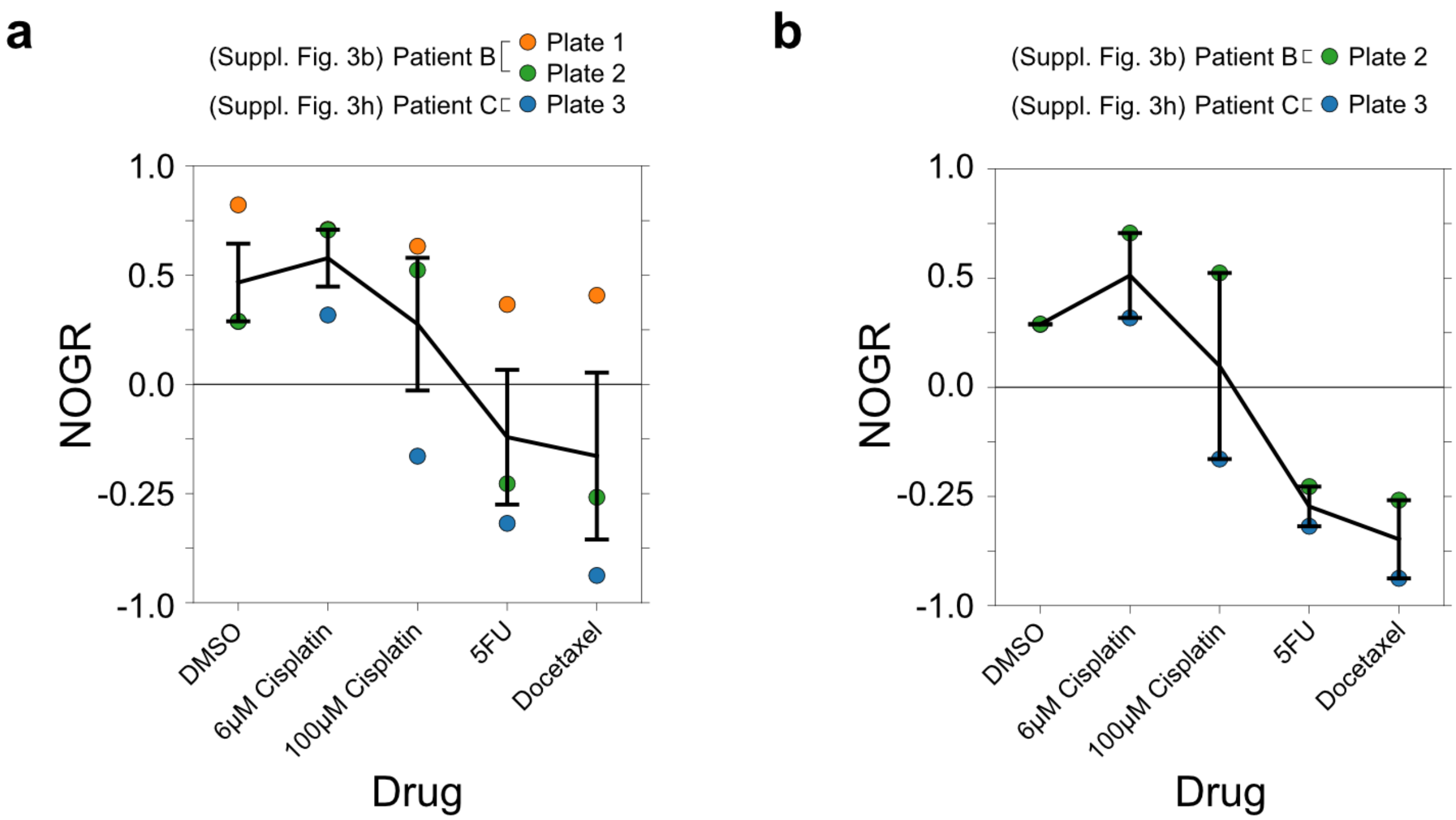

Reviewer Supplementary Figure 3k. Normalized Organoid Growth Rate (NOGR) validation of SPOT analysis. a) Median NOGR of all individual organoids per condition in each plate. **b)** Same as in a) but removing the outlier plate 2 organoids. Patient B and C were analyzed in Reviewer Suppl. Fig. 3b, 3h respectively.

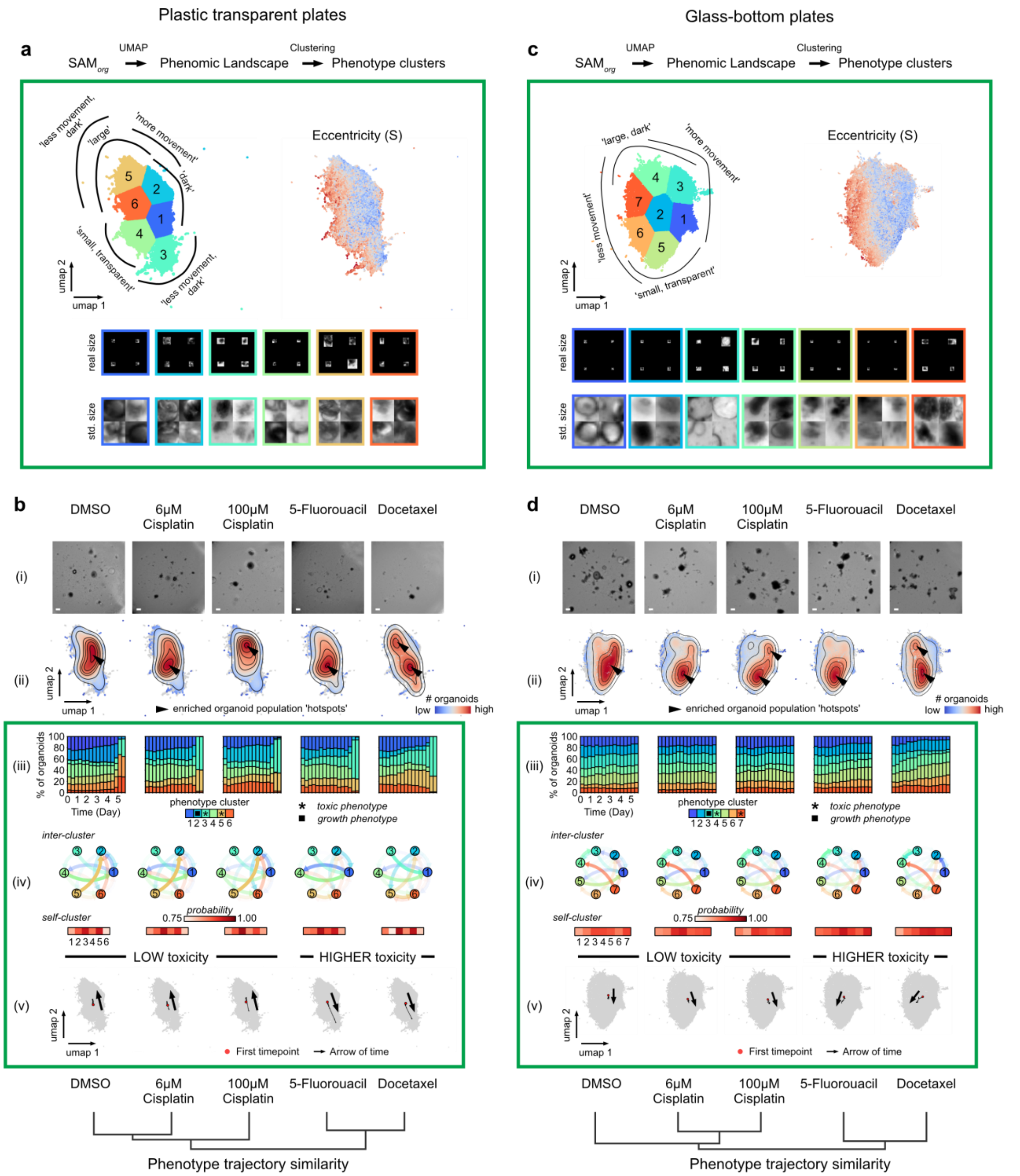

Reviewer Supplementary Figure 3I. Independent SPOT analysis of organoid culture in plastic transparent and optical-bottom plates. a) UMAP phenomic landscape of organoids cultured in plastic transparent plates. Top row: landscape colored by phenotypic clusters, dark blue to orange and labeled by increasing mean shape eccentricity of each cluster (left). Same landscape colored by shape eccentricity (right). Bottom row: four representative images of organoids in each cluster arranged as a 2x2 grid, in real and standardized (std.) size. n=55,581 points. **b)** Hierarchical clustering of drug conditions by phenotype trajectory similarity for plastic

transparent plates. For each drug condition, (i) example final video frame, scalebar: 250μm; (ii) point density heatmap, (iii) stacked barplot of temporal phenotype cluster composition, (iv) next timepoint inter-cluster and self-cluster transition probabilities, and (v) temporal phenotype trajectories. **c)** Same as a) for optical-bottom plates. n=53,078 points. **d)** Same as b) for optical-bottom plates.

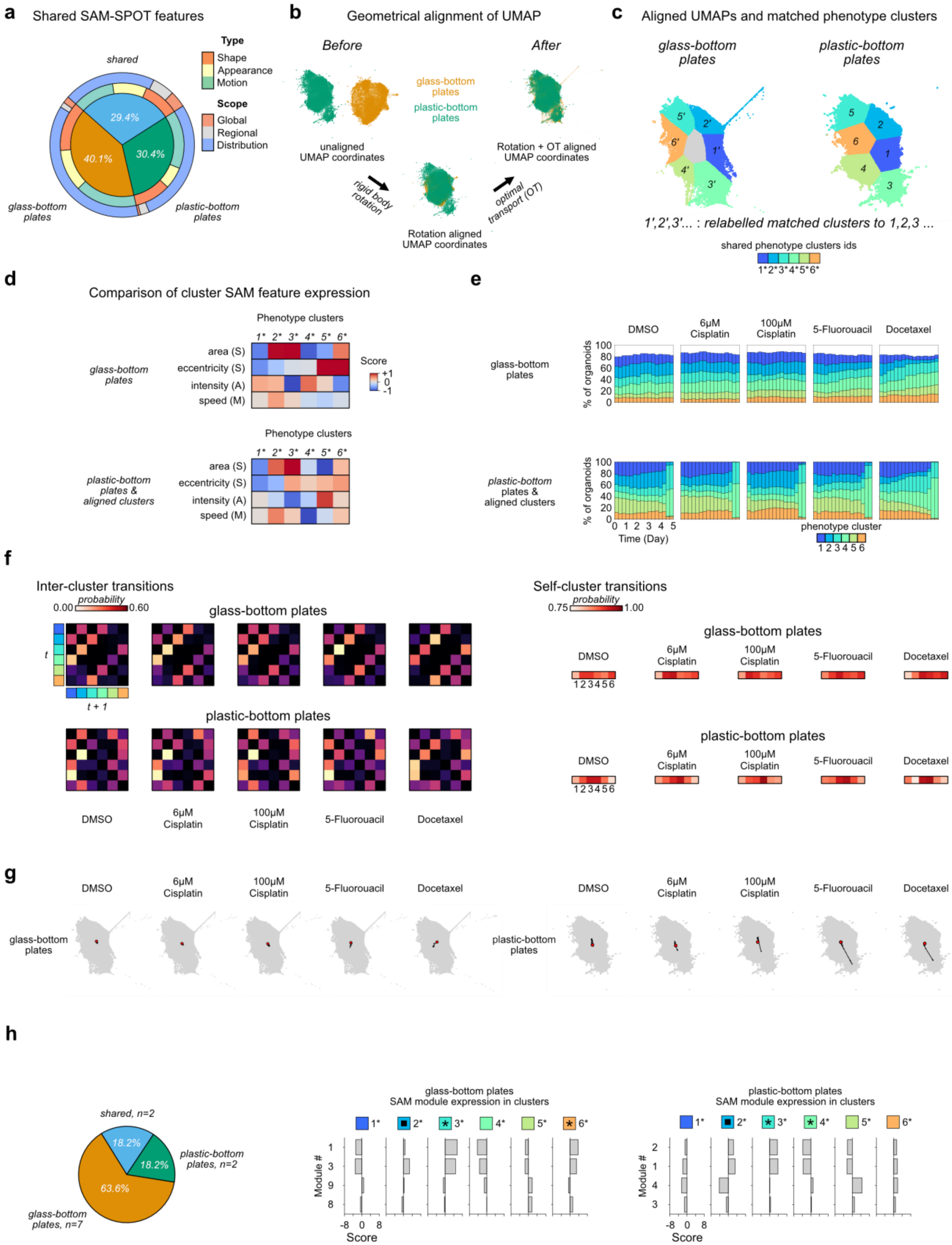

Reviewer Supplementary Figure 3m. Reproducibility analysis of SPOT analyzed organoids derived from the same patient cultured in plastic- vs glass- bottom plates. a) Pie chart of

the proportion of features that are unique or shared in the two sets of filtered SPOT-SAM phenomes. Outer two rings illustrate the composition of shape, appearance and motion features, and global, regional and distribution features in each of the unique and shared fractions. **b)** Geometrical alignment by rigid body rotation and optimal transport of the two independent UMAP phenomic landscapes. **c)** Relabeling of the phenotype clusters of the SPOT analyzed glass-bottom plate cultured organoids (Supp. Fig. 3I c) after matching the clusters to the organoids cultured in plastic-bottom plates (Reviewer Suppl. Fig 3I a) by their median UMAP coordinates. The relabeled integer cluster IDs are denoted by an additional dash ('). As the number of clusters do not match, the shared clusters are relabelled (*). **d)** Heatmap of the normalized mean expression score of selected SAM characteristics in each matched phenotype cluster for the glass-bottom (top) and plastic-bottom (bottom) plates. **e)** Stacked barplots of matched phenotype clusters. **f)** Inter-cluster (left) and self-cluster (right) transition probabilities depicted as a heatmap with matched phenotype clusters for each drug condition. **g)** Phenotypic trajectory for each drug condition after UMAP alignment for glass-bottom (left) and plastic-bottom (right) plates. **h)** SAM module analysis with phenotype cluster alignment. Pie chart of the proportion of SAM modules found that are unique or shared (left). Shared is defined as the matched modules sharing more than a quarter of the same features. Mean expression of matched SAM modules in each of the matched phenotype clusters between the two analyses. SAM modules are ordered top-to-bottom by the extent of shared features in the glass-bottom (middle), and plastic-bottom (right) plate SPOT analyses. SAM module numbers correspond to those in the respective original SPOT analyses in Reviewer Suppl. Fig. 3I.

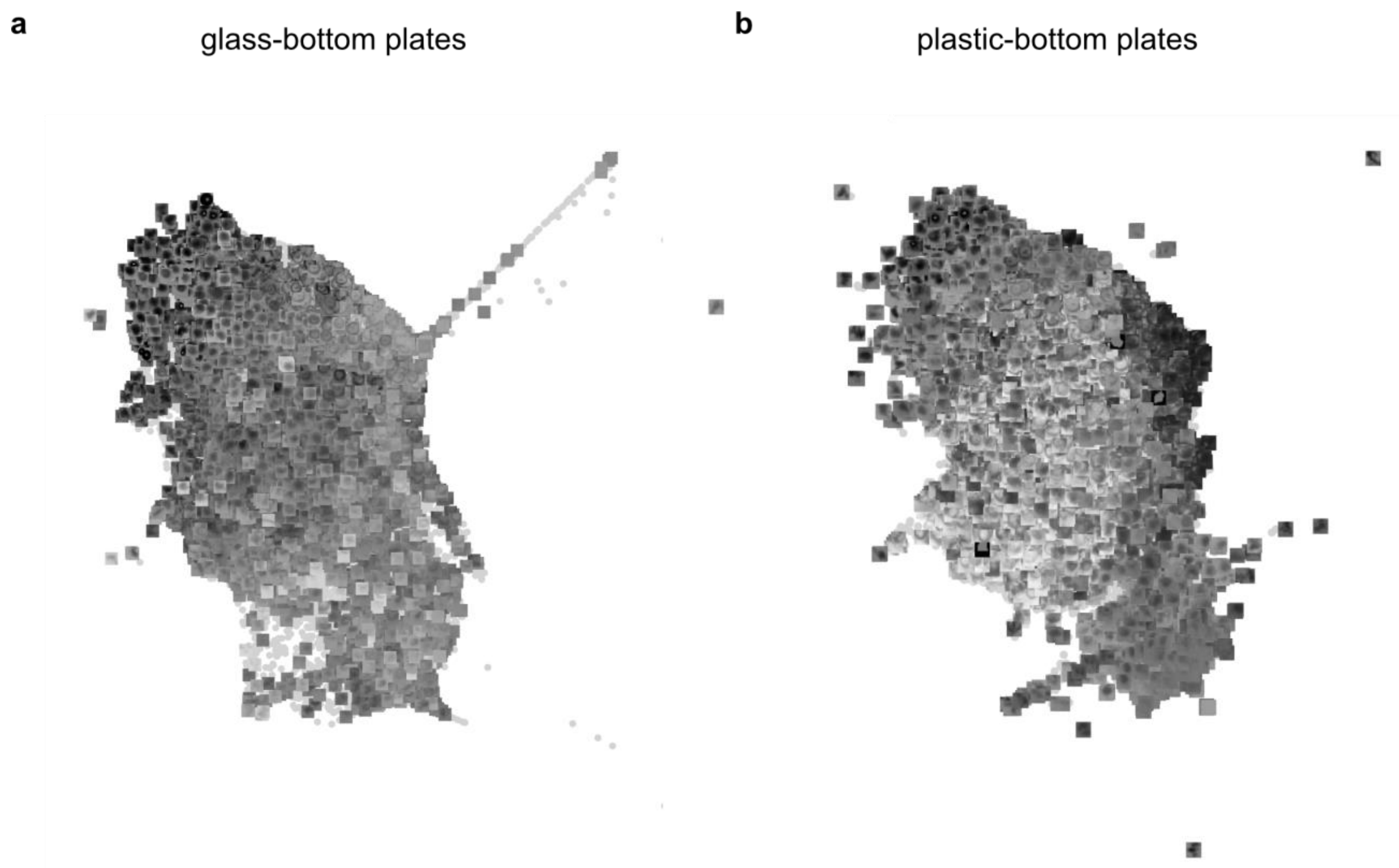

Reviewer Supplementary Figure 3n. Comparison of image patches embedded at the SPOT UMAP coordinates of Reviewer Suppl. Fig. 3l between organoids from the same patient cultured in glass- vs plastic- bottom plates after alignment. a) Organoid image patches plotted at SPOT analyzed UMAP coordinates cultured in glass-bottom plates (Reviewer Suppl. Fig. 3l c). **b)** Organoid image patches plotted at the aligned SPOT analyzed UMAP coordinates cultured in plastic-bottom plates (Reviewer Suppl. Fig. 3l a).

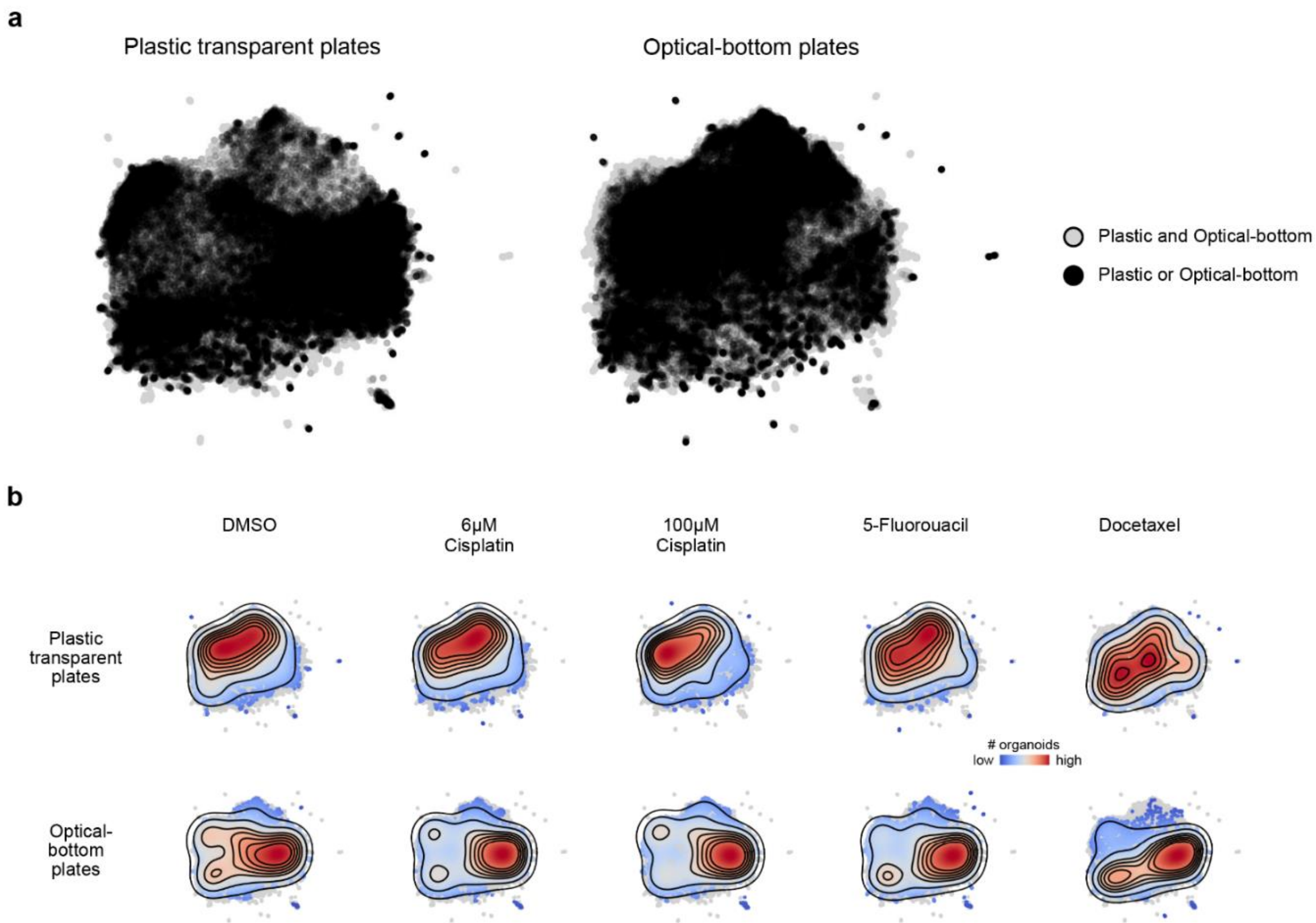

Reviewer Supplementary Figure 3o. Joint UMAP analysis of chemotherapy treated patient-derived organoids cultured in plastic and glass-bottom plates. a) Joint UMAP phenomic landscape (light grey points), using global feature normalization, with organoids cultured in plastic transparent (left) and optical-bottom plates (right) highlighted with black points. **b)** Point density heatmaps showing how organoids cultured in the different plates and drug condition distribute in the UMAP phenomic landscape.

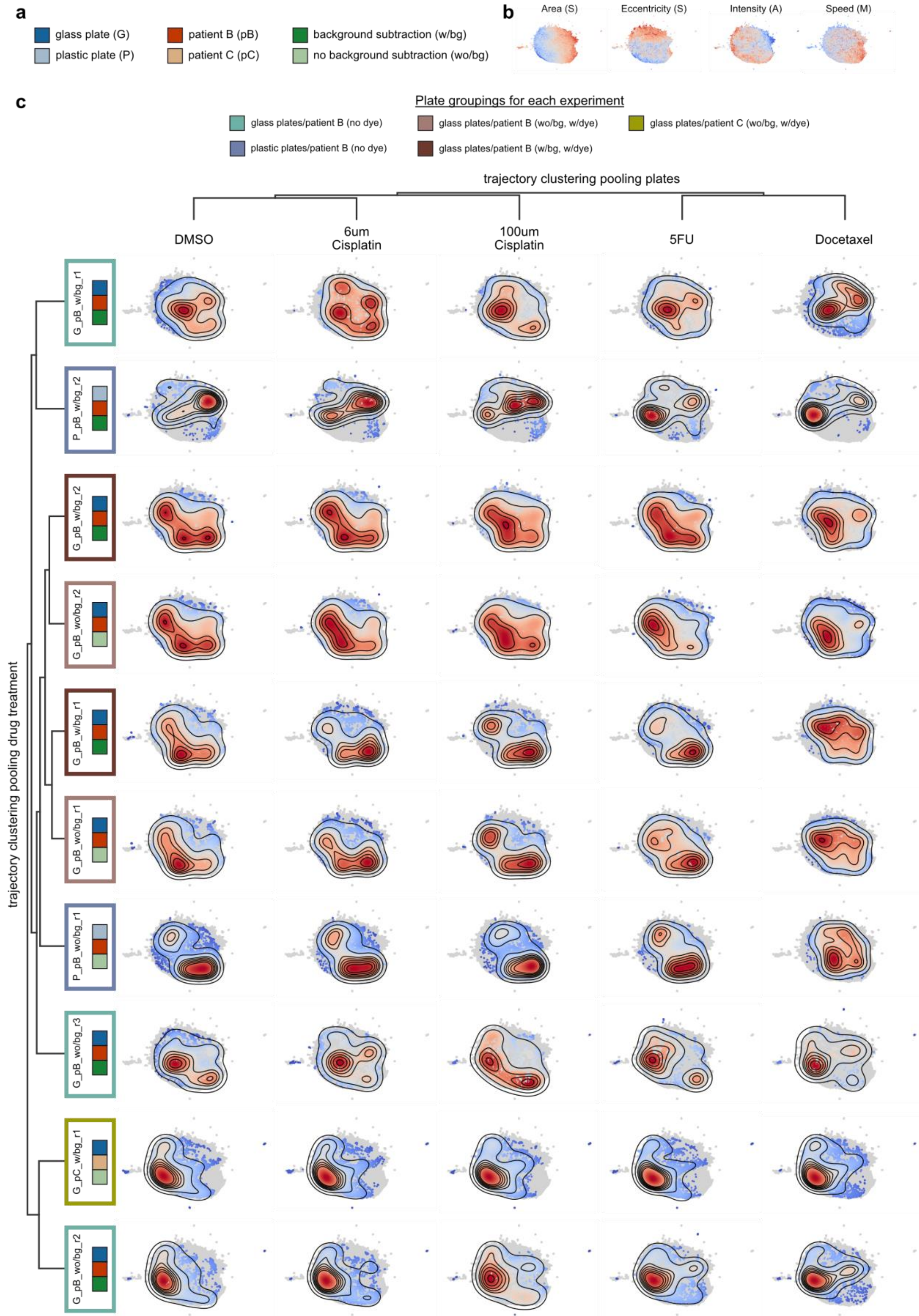

a
glass plate (G)
plastic plate (P)
patient B (pB)
patient C (pC)
background subtraction (w/bg)
no background subtraction (wo/bg)
b
Area (S)
Eccentricity (S)
Intensity (A)
Speed (M)
c
Plate groupings for each experiment
glass plates/patient B (no dye)
plastic plates/patient B (no dye)
glass plates/patient B (wo/bg, w/dye)
glass plates/patient B (w/bg, w/dye)
glass plates/patient C (wo/bg, w/dye)
trajectory clustering pooling plates
DMSO
6um Cisplatin
100um Cisplatin
5FU
Docetaxel
trajectory clustering pooling drug treatment
G_pB_w/bg_r1
P_pB_w/bg_r2
G_pB_w/bg_r2
G_pB_wo/bg_r2
G_pB_w/bg_r1
G_pB_wo/bg_r1
P_pB_wo/bg_r1
G_pB_wo/bg_r3
G_pC_w/bg_r1
G_pB_wo/bg_r2

Reviewer Supplementary Figure 3p. Joint UMAP analysis of chemotherapy treated patient-derived organoids using global feature normalization of SAM phenomes without batch effect correction of individual plates. a) Color coding of plate type, patient, and whether video frames were subject to flatfield background correction. **b)** Joint UMAP colored by selected shape, appearance and motion features. **c)** Point density heatmaps showing how organoids subject to different drug conditions (columns) distribute in the UMAP phenomic landscape in each individual plate (rows).

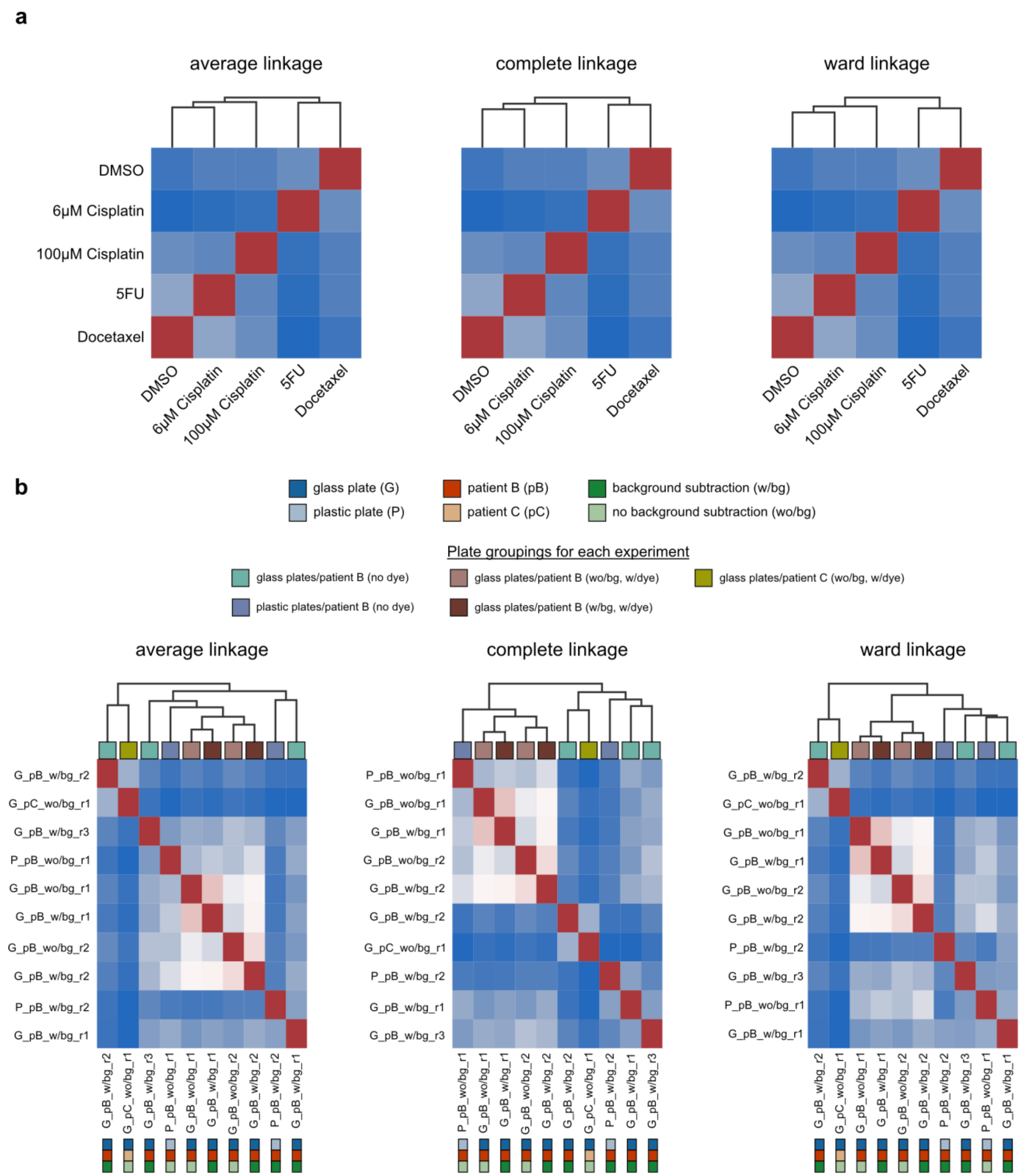

Reviewer Supplementary Figure 3q. Hierarchical clustering of chemotherapy drug treated patient-derived organoids with global feature normalization of SAM phenomes. a) Hierarchical clustering of drug conditions of the affinity matrix constructed from concatenating the trajectories across individual plates and using the specified linkages and Euclidean metric. **b)** Hierarchical clustering of individual plates of the affinity matrix constructed from concatenating the trajectories across drug conditions and using the specified linkages and Euclidean metric.

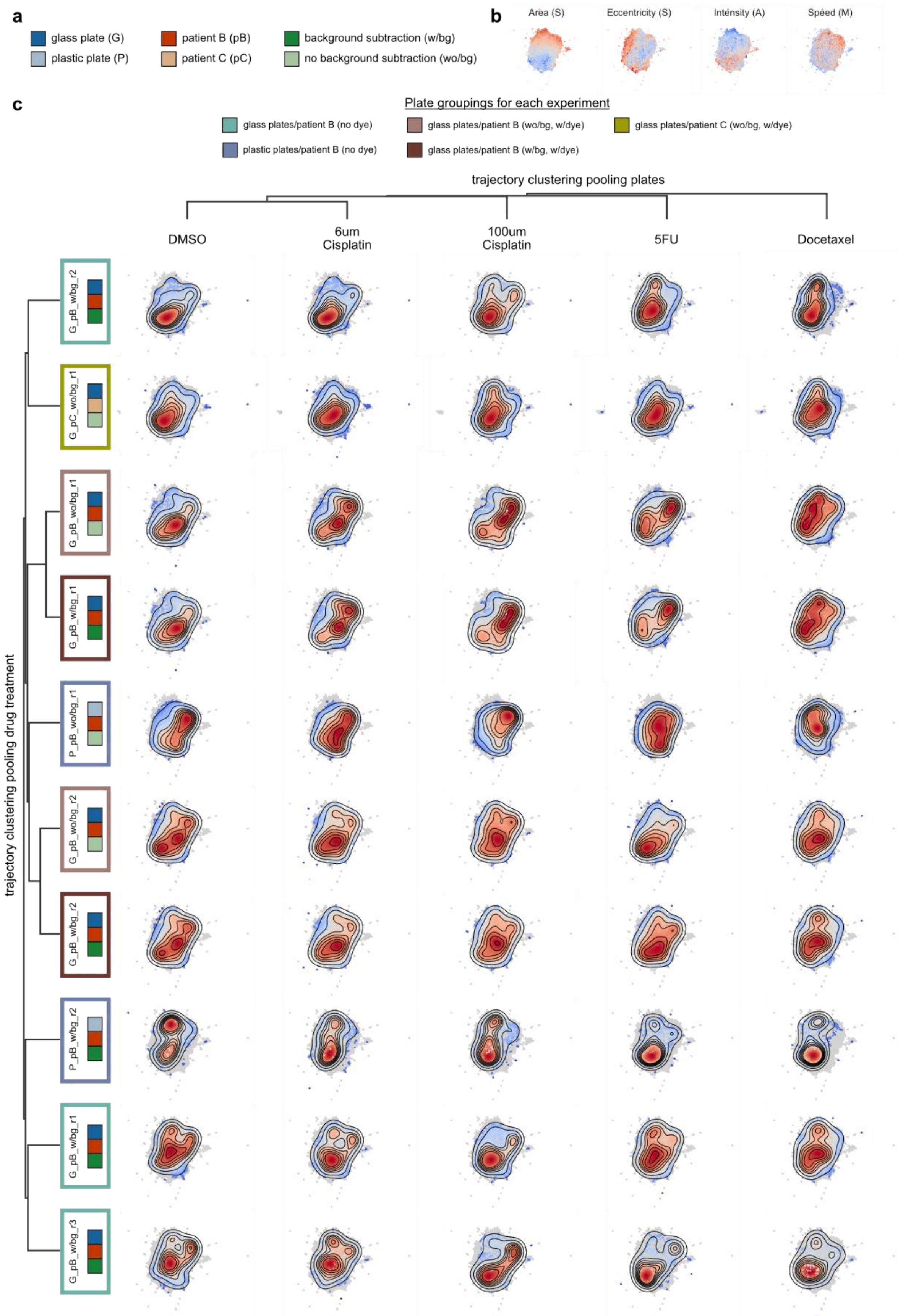
a
glass plate (G)
plastic plate (P)
patient B (pB)
patient C (pC)
background subtraction (w/bg)
no background subtraction (wo/bg)
b
Area (S)
Eccentricity (S)
Intensity (A)
Speed (M)
c
Plate groupings for each experiment
glass plates/patient B (no dye)
plastic plates/patient B (no dye)
glass plates/patient B (wo/bg, w/dye)
glass plates/patient B (w/bg, w/dye)
glass plates/patient C (wo/bg, w/dye)
trajectory clustering pooling plates
DMSO
6um Cisplatin
100um Cisplatin
5FU
Docetaxel
trajectory clustering pooling drug treatment
G_pB_w/bg_r2
G_pC_wo/bg_r1
G_pB_wo/bg_r1
G_pB_w/bg_r1
P_pB_wo/bg_r1
G_pB_wo/bg_r2
G_pB_w/bg_r2
P_pB_w/bg_r2
G_pB_w/bg_r1
G_pB_w/bg_r3

Reviewer Supplementary Figure 3r. Joint UMAP analysis of chemotherapy treated patient-derived organoids with batch effect correction by individual plate-based feature normalization of SAM phenomes. a) Color coding of plate type, patient, and whether video frames were subject to flatfield background correction. **b)** Joint UMAP colored by selected shape, appearance and motion features. **c)** Point density heatmaps showing how organoids subject to different drug conditions (columns) distribute in the UMAP phenomic landscape in each individual plate (rows).

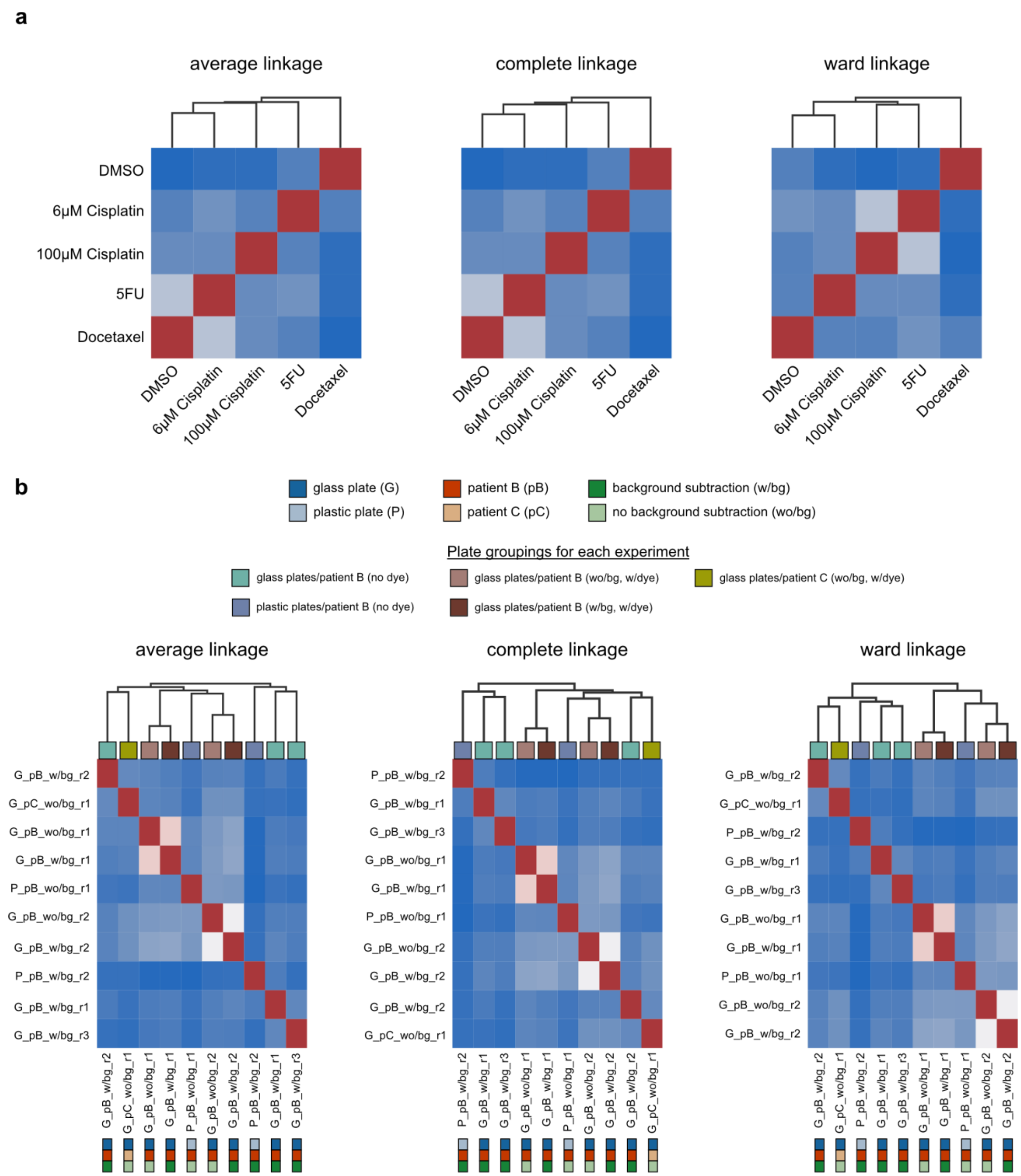

Reviewer Supplementary Figure 3s. Hierarchical clustering of chemotherapy drug treated patient-derived organoids with individual plate-based feature normalization of SAM phenomes. a) Hierarchical clustering of drug conditions of the affinity matrix constructed from concatenating the trajectories across individual plates and using the specified linkages and Euclidean metric. **b)** Hierarchical clustering of individual plates of the affinity matrix constructed

from concatenating the trajectories across drug conditions and using the specified linkages and Euclidean metric.
